# Supplementary material for: Identifying fibrogenic cells following salivary gland obstructive injury
Source: Front Cell Dev Biol. 2023 May 23;11:1190386. doi: 10.3389/fcell.2023.1190386 (PMC10242138; doi:10.3389/fcell.2023.1190386)
Supplement: Supplementary file 1 [file DataSheet1.pdf]

Supplementary Data

Identifying Fibrogenic Cells Following Salivary Gland Obstructive Injury

Amber L. Altrieth[1,2], Kevin J. O'Keefe[1,2,3], Victoria A. Gellatly[1,2], Joey R. Tavarez[1,2], Sage M. Feminella[1,6], Nicholas L. Moskwa[1,2,4], Carmalena V. Cordi[1,5], Judy C. Turrieta[1], Deirdre A. Nelson[2], and Melinda Larsen[1,2*]

[1]Department of Biological Sciences and The RNA Institute, University at Albany, State University of New York, Albany, New York, USA
[2]Molecular, Cellular, Developmental, and Neural Biology Graduate Program, Department of Biological Sciences, University at Albany, State University of New York Albany, New York, USA
[3]Current Location: Carl Zeiss Microscopy, LLC, White Plains, New York, USA
[4]Current Location: The Jackson Laboratory, Farmington, Connecticut, USA
[5]Current Location: Department of Biological Sciences, Rensselaer Polytechnic Institute, Troy, New York, USA
[6]Current Location: Albany Medical College, Albany, New York, USA

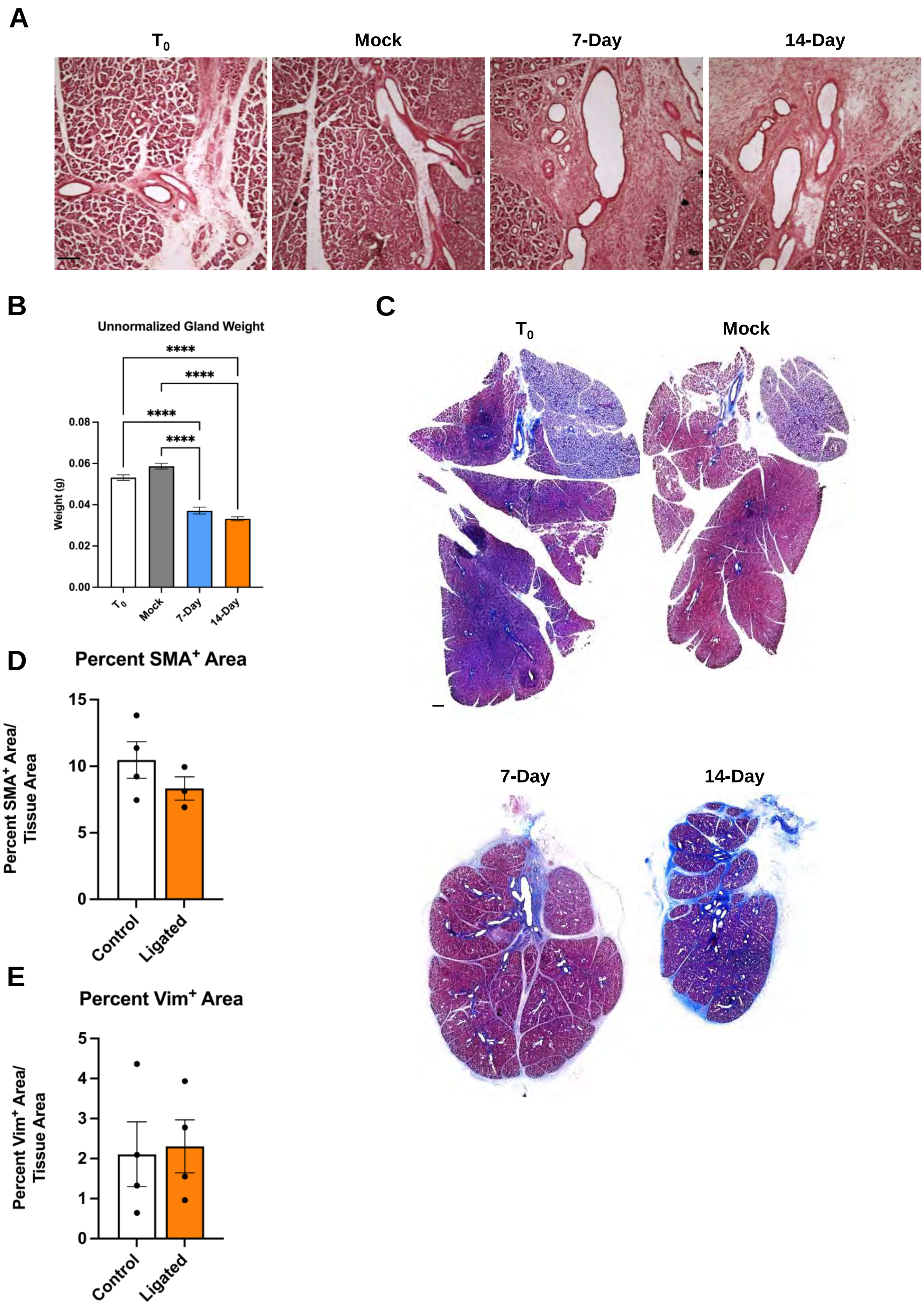
A
T0
Mock
7-Day
14-Day
B
Unnormalized Gland Weight
****
****
****
****
Weight (g)
0.00
0.02
0.04
0.06
0.08
T0
Mock
7-Day
14-Day
C
T0
Mock
7-Day
14-Day
D
Percent SMA+ Area
Percent SMA+ Area/
Tissue Area
0
5
10
15
Control
Ligated
E
Percent Vim+ Area
Percent Vim+ Area/
Tissue Area
0
1
2
3
4
5
Control
Ligated

Supplementary Figure 1. The stromal and ECM response to ductal ligation surgery

(**A**) 10X magnification images of H&E-stained glands from time zero (T_0),14-day mock (Mock), 7-day ligated (7-Day), and 14-day ligated (14-Day) mice. Scale bar 100 μm. (**B**) Raw gland weights in T_0, Mock, 7-Day, and 14-Day glands. N = 10, 25, 6, 34 respectively. (**C**) Masson's trichrome-stained whole gland images of T_0, Mock, 7-Day, and 14-Day mice. Scale bar 250 μm. Quantification of percent stain area normalized to total tissue area in $Gli1^{tm3(cre/ERT2)Alj}$/J;(CAG)$ROSA26^{tdTomato}$ (Gli1; R26tdT) 3-week induced control (Control) and 14-day ligated (Ligated) mice for (**D**) SMA (N = 4 and 3 respectively) and (**E**) vimentin (N = 4). Error bars: S.E.M. Statistical Test: Unpaired two-tailed t-test was performed using GraphPad Prism version 9.4.1. ****$p \leq 0.0001$.

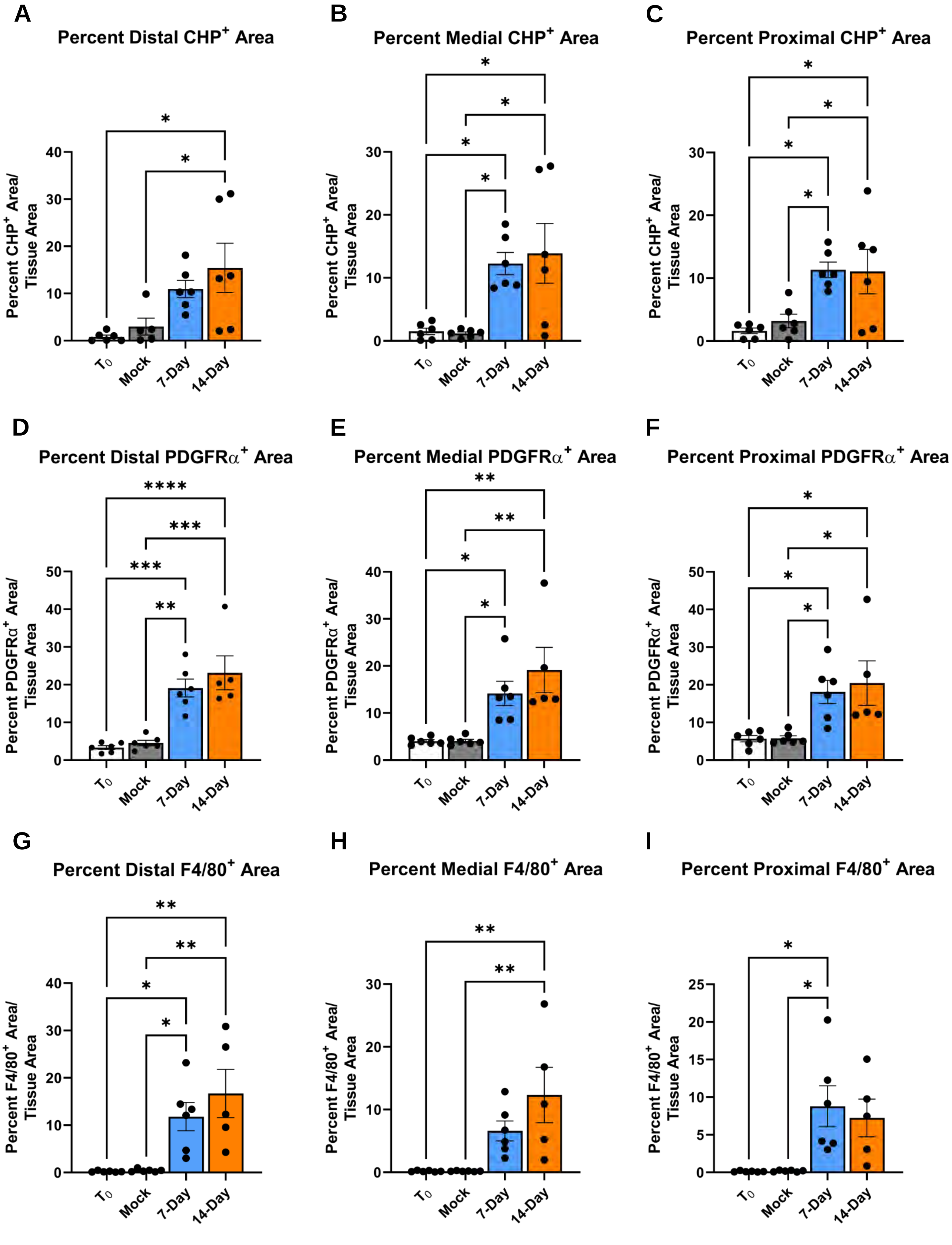

A
Percent Distal CHP+ Area
Percent CHP+ Area/ Tissue Area
T0
Mock
7-Day
14-Day
B
Percent Medial CHP+ Area
C
Percent Proximal CHP+ Area
D
Percent Distal PDGFRα+ Area
Percent PDGFRα+ Area/ Tissue Area
E
Percent Medial PDGFRα+ Area
F
Percent Proximal PDGFRα+ Area
G
Percent Distal F4/80+ Area
Percent F4/80+ Area/ Tissue Area
H
Percent Medial F4/80+ Area
I
Percent Proximal F4/80+ Area

Supplementary Figure 2. Regional stromal and ECM response to ductal ligation surgery

Quantification of percent stain area normalized to total tissue area for distal, medial, and proximal regions in the submandibular salivary gland for (**A, B, C**) CHP, (**D, E, F**) PDGFRα, and (**G, H, I**) F4/80. N = 6, 6, 6, and 5 respectively from 2 to 3 images per region. Error bars: S.E.M. Statistics: One-way ANOVA followed by Tukey's multiple comparisons test was performed using GraphPad Prism version 9.4.1. *$p \leq 0.05$, **$p \leq 0.01$, ***$p \leq 0.001$, ****$p \leq 0.0001$.

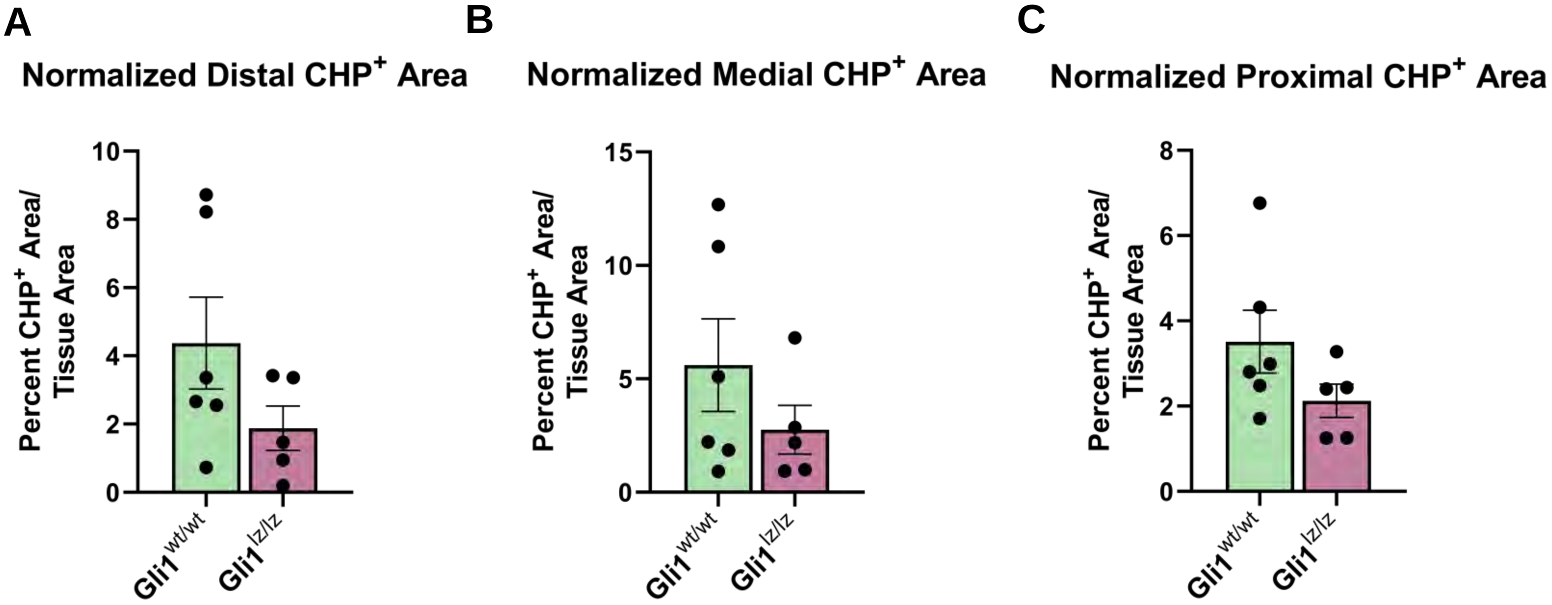
A
Normalized Distal CHP+ Area
Percent CHP+ Area/ Tissue Area
10
8
6
4
2
0
Gli1wt/wt
Gli1lz/lz
B
Normalized Medial CHP+ Area
Percent CHP+ Area/ Tissue Area
15
10
5
0
Gli1wt/wt
Gli1lz/lz
C
Normalized Proximal CHP+ Area
Percent CHP+ Area/ Tissue Area
8
6
4
2
0
Gli1wt/wt
Gli1lz/lz

Supplementary Figure 3. Regional CHP in 14-day ligated Gli1 null and wildtype mice

Quantification of percent stain area normalized to total tissue area for (**A**) distal, (**B**) medial, and (**C**) proximal regions in the submandibular salivary gland for CHP in 14-day ligated Gli1 wildtype ($Gli1^{wt/wt}$) and Gli1 null ($Gli1^{lz/lz}$) mice from 1 image per region. Statistical Test: Unpaired two-tailed t-test was performed using GraphPad Prism version 9.4.1. $Gli1^{wt/wt}$ N=6 and $Gli1^{lz/lz}$ N=5.

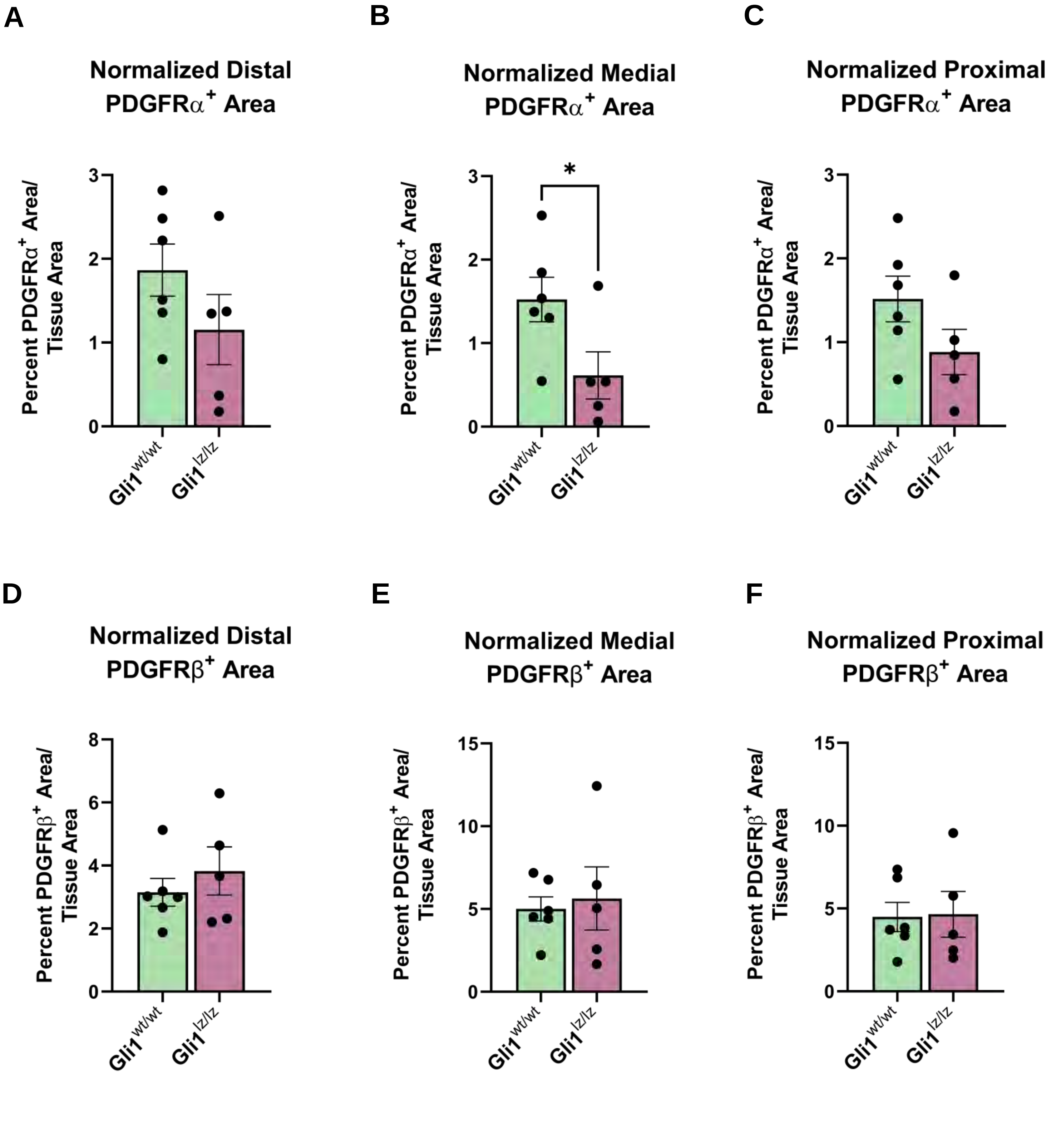
A
Normalized Distal
PDGFRα+ Area
Percent PDGFRα+ Area/
Tissue Area
0
1
2
3
Gli1wt/wt
Gli1lz/lz
B
Normalized Medial
PDGFRα+ Area
*
Percent PDGFRα+ Area/
Tissue Area
0
1
2
3
Gli1wt/wt
Gli1lz/lz
C
Normalized Proximal
PDGFRα+ Area
Percent PDGFRα+ Area/
Tissue Area
0
1
2
3
Gli1wt/wt
Gli1lz/lz
D
Normalized Distal
PDGFRβ+ Area
Percent PDGFRβ+ Area/
Tissue Area
0
2
4
6
8
Gli1wt/wt
Gli1lz/lz
E
Normalized Medial
PDGFRβ+ Area
Percent PDGFRβ+ Area/
Tissue Area
0
5
10
15
Gli1wt/wt
Gli1lz/lz
F
Normalized Proximal
PDGFRβ+ Area
Percent PDGFRβ+ Area/
Tissue Area
0
5
10
15
Gli1wt/wt
Gli1lz/lz

Supplementary Figure 4. Regional changes in PDGFRα and PDGFRβ in 14-day ligated Gli1 null and wildtype mice

Quantification of percent stain area normalized to total tissue area for distal, medial, and proximal regions in the submandibular salivary gland for (**A, B, C**) PDGFRα and (**D, E, F**) PDGFRβ in 14-day ligated Gli1 wildtype ($Gli1^{wt/wt}$) and Gli1 null ($Gli1^{lz/lz}$) mice from 2-3 images per region. Statistical Test: Unpaired two-tailed t-test was performed using GraphPad Prism version 9.4.1. *$p \leq 0.05$. $Gli1^{wt/wt}$ N=6 and $Gli1^{lz/lz}$ N=5.

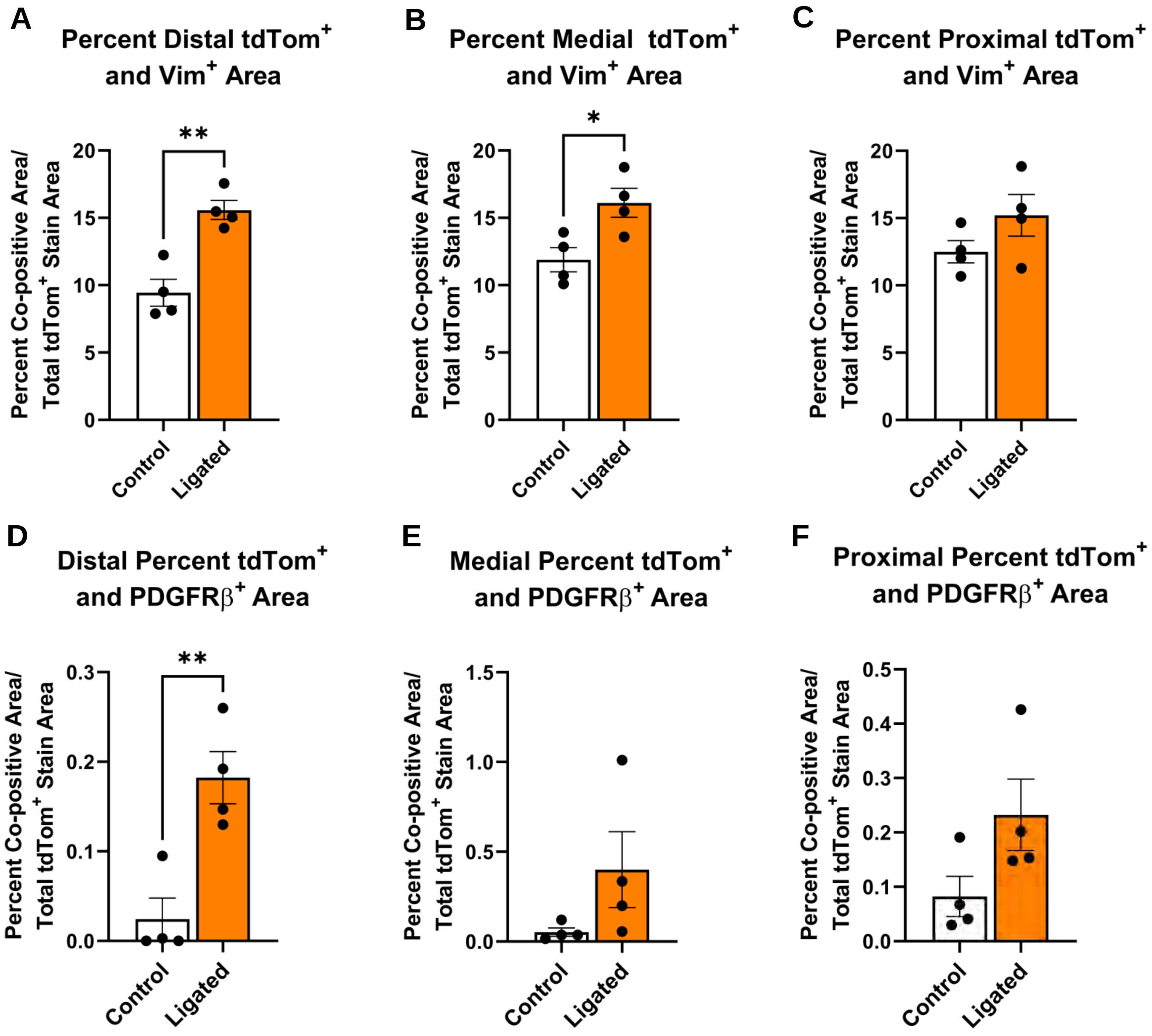
A
Percent Distal tdTom+ and Vim+ Area
B
Percent Medial tdTom+ and Vim+ Area
C
Percent Proximal tdTom+ and Vim+ Area
D
Distal Percent tdTom+ and PDGFRβ+ Area
E
Medial Percent tdTom+ and PDGFRβ+ Area
F
Proximal Percent tdTom+ and PDGFRβ+ Area
Percent Co-positive Area/ Total tdTom+ Stain Area
Control
Ligated
**
*

Supplementary Figure 5. Gli1-lineage derived cells show regional differences in vimentin and PDGFRβ colocalization

Quantification of percent colocalized stain area normalized to total Gli1;R26tdT$^+$ stain area for distal, medial, and proximal regions in the submandibular salivary gland for (**A, B, C**) vimentin (Vim), and (**D, E, F**) PDGFRβ in 3-week induced control (Control) or 14-day ligated (Ligated) Gli1;R26tdT mice from 2-3 images per region. Statistical Test: Unpaired two-tailed t-test was performed using GraphPad Prism version 9.4.1. *$p \leq 0.05$ and ** $p \leq 0.01$. N = 4

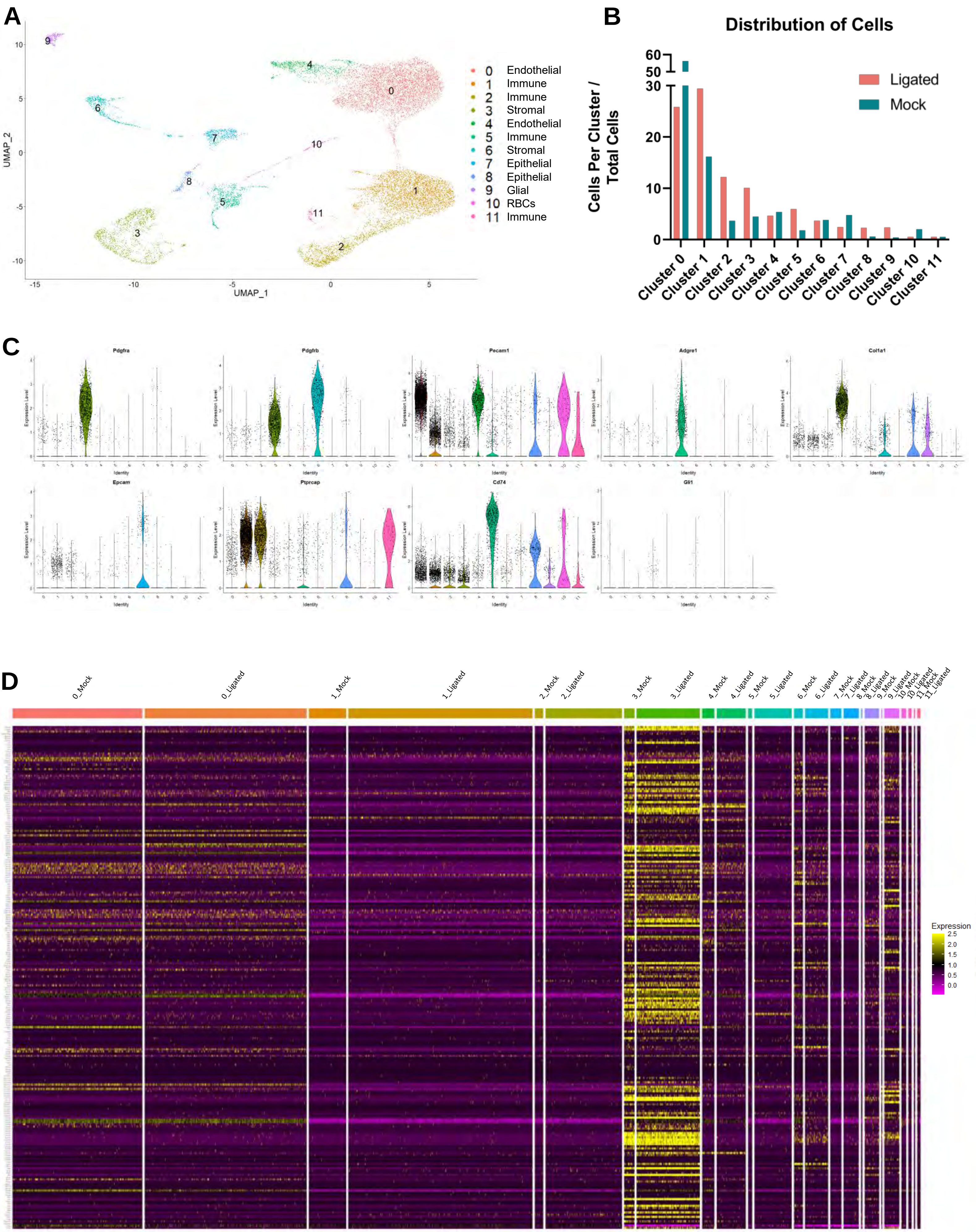
A
UMAP_1
UMAP_2
0 Endothelial
1 Immune
2 Immune
3 Stromal
4 Endothelial
5 Immune
6 Stromal
7 Epithelial
8 Epithelial
9 Glial
10 RBCs
11 Immune
B
Distribution of Cells
Cells Per Cluster / Total Cells
Ligated
Mock
Cluster 0
Cluster 1
Cluster 2
Cluster 3
Cluster 4
Cluster 5
Cluster 6
Cluster 7
Cluster 8
Cluster 9
Cluster 10
Cluster 11
C
Pdgfra
Pdgfrb
Pecam1
Adgre1
Col1a1
Epcam
Ptprcap
Cd74
Gli1
Identity
Expression Level
D
0_Mock
0_Ligated
1_Mock
1_Ligated
2_Mock
2_Ligated
3_Mock
3_Ligated
4_Mock
4_Ligated
5_Mock
5_Ligated
6_Mock
6_Ligated
7_Mock
7_Ligated
8_Mock
8_Ligated
9_Mock
9_Ligated
10_Mock
10_Ligated
11_Mock
11_Ligated
Expression

Supplemental Figure 6. scRNAseq reveals cluster 3 expresses high levels of stromal identity markers and ECM-associated genes

(**A**) UMAP of merged 14-day ligated and 14-day mock datasets showing 12 different clusters of cells sequenced. (**B**) Percent distribution of cells was calculated by dividing the number of cells in each cluster by the total number of cells in their sample of origin multiplied by 100. (**C**) Violin plots showing the expression of *Pdgfra, Pdgfrb, Pecam1, Adgre1, Col1a1, Epcam, Ptprcap, Cd74,* and *Gli1* which were used for identification of clusters. (**D**) A heatmap was generated using “matrisome-associated” genes that encode for collagens, proteoglycans and glycoproteins, with cluster 3, the stromal population, expressing the highest levels in both mock and ligated conditions.

Supplementary Table 1. Antibodies and dilutions used

Table showing the primary antibodies, direct conjugates, secondary antibodies, and other fluorescent peptides used for staining including the antibody name, the company name, the catalog number, the RRID number and the dilution used for staining, where applicable.

	Antibody	Company	Catalog Number	RRID Number	Dilution Used
Primary Antibodies	PDGFRα goat	R & D Systems	AF1062	AB_2236897	1:100
	Living Colors DSRed polyclonal antibody	Takara Biosciences	632496	AB_10013483	1:1000
	Anti-mouse CD140b (PDGFRb)	Invitrogen	14-1402-82	AB_467493	1:200
	CD31 (clone MEC13.3)	BD Pharmingen	553370	AB_394816	1:200
	F4/80 clone BM8 rat	Biolegend	123101	AB_893504	1:200
	RFP	Rockland	200-101-379	AB_2744552	1:1000
Direct Conjugates	A488 vimentin	Cell Signaling	9854	AB_10829352	1:100
	A488 smooth muscle actin (clone 1A4)	Invitrogen	53-9760-80	AB_2574460	1:200
	Cy3 smooth muscle actin (clone 1A4)	Sigma	C6198	AB_476856	1:600
	Alexa Fluor 647 anti-Tubulin β 3 (TUBB3)	Biolegend	801210	AB_2686931	1:200
Secondary Antibodies	Anti-rabbit cy 2	Jackson ImmunoResearch	711-546-152		1:500
	anti-goat 488	Jackson ImmunoResearch	705-546-147		1:500
	Anti-rat no mouse cy 3	Jackson ImmunoResearch	712-166-153		1:500

	Anti-rabbit cy 3	Jackson ImmunoResearch	711-166-152		1:500
	Anti-goat cy 5	Jackson ImmunoResearch	705-606-147		1:500
	anti-rat no mouse Cy5	Jackson ImmunoResearch	712-606-153		1:500
Other	5-FAM-CHP	Advanced Biomatrix	5264-60ug		40μL of 20 μM stock

Supplementary Table 2. Positively upregulated genes in *Gli1*$^{+}$ cells

The FindAllMarkers command from the Seurat package in Rstudio was used to examine all differentially expressed genes between 14-day ligated and 14-day mock *Gli1*$^{+}$ cells. Genes shown have an adjusted p value < 0.05.

	p_val	avg_log2FC	pct.1	pct.2	p_val_adj	cluster	gene
Nme2.1	1.15E-08	2.969054	0.912	0.059	0.000232	Ligated_Gli+	Nme2
AY036118	8.09E-07	2.959726	0.941	0.561	0.016296	Mock_Gli+	AY036118
Rpl13a.1	2.44E-08	2.557193	0.947	0.588	0.000492	Ligated_Gli+	Rpl13a
Gas5.1	8.62E-08	2.437051	0.842	0	0.001737	Ligated_Gli+	Gas5
Atp6v0c.1	2.32E-08	2.250302	0.895	0.059	0.000467	Ligated_Gli+	Atp6v0c
Tle5.1	8.62E-08	2.215576	0.842	0	0.001737	Ligated_Gli+	Tle5
Micos10.1	2.48E-07	1.90341	0.807	0	0.004989	Ligated_Gli+	Micos10
mt-Atp8	3.37E-08	1.805314	1	0.614	0.000678	Mock_Gli+	mt-Atp8
Rnasek.1	1.47E-07	1.784067	0.825	0	0.002962	Ligated_Gli+	Rnasek
Atp5md.1	4.12E-07	1.78051	0.789	0	0.008302	Ligated_Gli+	Atp5md
Grcc10.1	1.92E-06	1.721904	0.754	0.059	0.038709	Ligated_Gli+	Grcc10
Aes	6.87E-09	1.677924	0.529	0	0.000138	Mock_Gli+	Aes
Minos1	8.07E-12	1.670877	0.706	0	1.62E-07	Mock_Gli+	Minos1
Atp5o.2	8.62E-08	1.669184	0.842	0	0.001737	Ligated_Gli+	Atp5o
Atp5o.1	6.73E-14	1.651892	0.824	0	1.35E-09	Mock_Gli+	Atp5o.1
Fam198b	6.87E-09	1.646321	0.529	0	0.000138	Mock_Gli+	Fam198b
Atp5mpl.1	1.77E-06	1.642173	0.737	0	0.035645	Ligated_Gli+	Atp5mpl
Ndufb1-ps.1	1.77E-06	1.607464	0.737	0	0.035645	Ligated_Gli+	Ndufb1-ps
Usmg5	8.07E-12	1.598941	0.706	0	1.62E-07	Mock_Gli+	Usmg5
1110008F13Rik	8.07E-12	1.346015	0.706	0	1.62E-07	Mock_Gli+	1110008F13Rik
2010107E04Rik	7.65E-10	1.334062	0.588	0	1.54E-05	Mock_Gli+	2010107E04Rik
1700020I14Rik	6.87E-09	1.222484	0.529	0	0.000138	Mock_Gli+	1700020I14Rik
1810022K09Rik	7.59E-13	1.212908	0.765	0	1.53E-08	Mock_Gli+	1810022K09Rik
Zcchc6	8.08E-11	1.205116	0.647	0	1.63E-06	Mock_Gli+	Zcchc6
Trove2	5.85E-08	1.158301	0.471	0	0.001178	Mock_Gli+	Trove2
2010111I01Rik	7.65E-10	1.137548	0.588	0	1.54E-05	Mock_Gli+	2010111I01Rik
Fam96b	7.65E-10	1.119438	0.588	0	1.54E-05	Mock_Gli+	Fam96b
Fam208a	5.85E-08	0.931175	0.471	0	0.001178	Mock_Gli+	Fam208a
BC003331	6.87E-09	0.909603	0.529	0	0.000138	Mock_Gli+	BC003331
2410015M20Rik	7.65E-10	0.89785	0.588	0	1.54E-05	Mock_Gli+	2410015M20Rik
Tmem5	6.87E-09	0.848406	0.529	0	0.000138	Mock_Gli+	Tmem5
Coro1b	6.10E-09	0.841451	0.588	0.018	0.000123	Mock_Gli+	Coro1b
Taf6l	3.64E-07	0.812025	0.529	0.035	0.007321	Mock_Gli+	Taf6l
Zcchc11	4.75E-07	0.750525	0.412	0	0.009567	Mock_Gli+	Zcchc11
Skiv2l2	4.75E-07	0.721403	0.412	0	0.009567	Mock_Gli+	Skiv2l2
Dirc2	4.75E-07	0.66283	0.412	0	0.009567	Mock_Gli+	Dirc2
0610037L13Rik	4.75E-07	0.634829	0.412	0	0.009567	Mock_Gli+	0610037L13Rik
Fbxo18	4.75E-07	0.54309	0.412	0	0.009567	Mock_Gli+	Fbxo18
Fbxo18.1	4.75E-07	-0.54309	0	0.412	0.009567	Ligated_Gli+	Fbxo18
0610037L13Rik.1	4.75E-07	-0.63483	0	0.412	0.009567	Ligated_Gli+	0610037L13Rik

Dirc2.1	4.75E-07	-0.66283	0	0.412	0.009567	Ligated_Gli+	Dirc2
Skiv2l2.1	4.75E-07	-0.7214	0	0.412	0.009567	Ligated_Gli+	Skiv2l2
Zcchc11.1	4.75E-07	-0.75053	0	0.412	0.009567	Ligated_Gli+	Zcchc11
Taf6l.1	3.64E-07	-0.81203	0.035	0.529	0.007321	Ligated_Gli+	Taf6l
Coro1b.1	6.10E-09	-0.84145	0.018	0.588	0.000123	Ligated_Gli+	Coro1b
Tmem5.1	6.87E-09	-0.84841	0	0.529	0.000138	Ligated_Gli+	Tmem5
2410015M20Rik.1	7.65E-10	-0.89785	0	0.588	1.54E-05	Ligated_Gli+	2410015M20Rik
BC003331.1	6.87E-09	-0.9096	0	0.529	0.000138	Ligated_Gli+	BC003331
Fam208a.1	5.85E-08	-0.93118	0	0.471	0.001178	Ligated_Gli+	Fam208a
Fam96b.1	7.65E-10	-1.11944	0	0.588	1.54E-05	Ligated_Gli+	Fam96b
2010111I01Rik.1	7.65E-10	-1.13755	0	0.588	1.54E-05	Ligated_Gli+	2010111I01Rik
Trove2.1	5.85E-08	-1.1583	0	0.471	0.001178	Ligated_Gli+	Trove2
Zcchc6.1	8.08E-11	-1.20512	0	0.647	1.63E-06	Ligated_Gli+	Zcchc6
1810022K09Rik.1	7.59E-13	-1.21291	0	0.765	1.53E-08	Ligated_Gli+	1810022K09Rik
1700020I14Rik.1	6.87E-09	-1.22248	0	0.529	0.000138	Ligated_Gli+	1700020I14Rik
2010107E04Rik.1	7.65E-10	-1.33406	0	0.588	1.54E-05	Ligated_Gli+	2010107E04Rik
1110008F13Rik.1	8.07E-12	-1.34601	0	0.706	1.62E-07	Ligated_Gli+	1110008F13Rik
Usmg5.1	8.07E-12	-1.59894	0	0.706	1.62E-07	Ligated_Gli+	Usmg5
Ndufb1-ps	1.77E-06	-1.60746	0	0.737	0.035645	Mock_Gli+	Ndufb1-ps
Atp5mpl	1.77E-06	-1.64217	0	0.737	0.035645	Mock_Gli+	Atp5mpl
Fam198b.1	6.87E-09	-1.64632	0	0.529	0.000138	Ligated_Gli+	Fam198b
Atp5o.1.1	6.73E-14	-1.65189	0	0.824	1.35E-09	Ligated_Gli+	Atp5o.1
Atp5o	8.62E-08	-1.66918	0	0.842	0.001737	Mock_Gli+	Atp5o
Minos1.1	8.07E-12	-1.67088	0	0.706	1.62E-07	Ligated_Gli+	Minos1
Aes.1	6.87E-09	-1.67792	0	0.529	0.000138	Ligated_Gli+	Aes
Grcc10	1.92E-06	-1.7219	0.059	0.754	0.038709	Mock_Gli+	Grcc10
Atp5md	4.12E-07	-1.78051	0	0.789	0.008302	Mock_Gli+	Atp5md
Rnasek	1.47E-07	-1.78407	0	0.825	0.002962	Mock_Gli+	Rnasek
mt-Atp8.1	3.37E-08	-1.80531	0.614	1	0.000678	Ligated_Gli+	mt-Atp8
Micos10	2.48E-07	-1.90341	0	0.807	0.004989	Mock_Gli+	Micos10
Tle5	8.62E-08	-2.21558	0	0.842	0.001737	Mock_Gli+	Tle5
Atp6v0c	2.32E-08	-2.2503	0.059	0.895	0.000467	Mock_Gli+	Atp6v0c
Gas5	8.62E-08	-2.43705	0	0.842	0.001737	Mock_Gli+	Gas5
Rpl13a	2.44E-08	-2.55719	0.588	0.947	0.000492	Mock_Gli+	Rpl13a
AY036118.1	8.09E-07	-2.95973	0.561	0.941	0.016296	Ligated_Gli+	AY036118
Nme2	1.15E-08	-2.96905	0.059	0.912	0.000232	Mock_Gli+	Nme2

Supplementary Table 3. Positively upregulated genes in stromal subsetted cells

The FindAllMarkers command from the Seurat package in Rstudio was used to determine the positively upregulated genes in the stromal subsetted cells expressing *Pdgfra, Pdgfrb,* or *Gli1*. Clusters are divided into 14-day ligated cells or mock ligated cells denoted by the “L” or “M” respectively, followed by their cluster number. Genes shown have an adjusted p value of $p < 0.05$.

	p_val	avg_log2FC	pct.1	pct.2	p_val_adj	cluster	gene
Mucl2	7.38E-08	0.622332	0.74	0.738	0.001487	0 L1 _	Mucl2
Iigp11	1.27E-08	0.452436	0.509	0.368	0.000257	0 L1 _	Iigp1
Sh3glb11	1.12E-09	0.252942	0.878	0.77	2.26E-05	0 L1 _	Sh3glb1
Hlf	1.56E-12	0.255726	0.25	0.136	3.15E-08	0 L1 _	Hlf
Smdt1	6.22E-13	0.255473	0.883	0.748	1.25E-08	0 L1 _	Smdt1
F830016B08Rik	7.21E-15	0.261458	0.164	0.063	1.45E-10	0 L1 _	F830016B08Rik
Pid1	6.66E-15	0.253918	0.423	0.257	1.34E-10	0 L1 _	Pid1
Abca11	4.25E-15	0.273139	0.467	0.304	8.55E-11	0 L1 _	Abca1
Hcfc1r1	1.86E-15	0.254016	0.738	0.559	3.74E-11	0 L1 _	Hcfc1r1
Ier3ip11	1.60E-15	0.265152	0.761	0.592	3.23E-11	0 L1 _	Ier3ip1
Imp3	2.46E-16	0.255102	0.796	0.606	4.95E-12	0 L1 _	Imp3
Tm9sf31	2.26E-16	0.25314	0.847	0.685	4.55E-12	0 L1 _	Tm9sf3
Col4a4	2.32E-17	0.254517	0.249	0.118	4.67E-13	0 L1 _	Col4a4
Arcn11	1.23E-17	0.258555	0.647	0.448	2.47E-13	0 L1 _	Arcn1
Ddb1	1.18E-17	0.265922	0.623	0.427	2.38E-13	0 L1 _	Ddb1
Tmem64	7.30E-18	0.271098	0.332	0.182	1.47E-13	0 L1 _	Tmem64
Adh11	6.83E-18	0.329914	0.252	0.116	1.38E-13	0 L1 _	Adh1
Serpine2	6.28E-18	0.345982	0.669	0.455	1.26E-13	0 L1 _	Serpine2
Resf1	2.20E-18	0.267046	0.585	0.366	4.43E-14	0 L1 _	Resf1
Dnajc1	1.84E-18	0.275029	0.737	0.528	3.71E-14	0 L1 _	Dnajc1
Nrip1	1.54E-18	0.292644	0.733	0.532	3.11E-14	0 L1 _	Nrip1
Grina1	1.50E-18	0.256986	0.71	0.472	3.01E-14	0 L1 _	Grina
Sec61b1	1.03E-18	0.29495	0.902	0.723	2.08E-14	0 L1 _	Sec61b
Adamts151	7.24E-19	0.312005	0.342	0.182	1.46E-14	0 L1 _	Adamts15
C1d	6.90E-19	0.259023	0.582	0.389	1.39E-14	0 L1 _	C1d
Uap1l1	6.18E-19	0.251401	0.347	0.189	1.25E-14	0 L1 _	Uap1l1
Itpr11	5.44E-19	0.253808	0.602	0.395	1.09E-14	0 L1 _	Itpr1
Dnajb11	5.01E-19	0.270581	0.631	0.441	1.01E-14	0 L1 _	Dnajb11
Aprt	3.41E-19	0.253091	0.683	0.477	6.86E-15	0 L1 _	Aprt
Acbd3	2.97E-19	0.251901	0.592	0.395	5.98E-15	0 L1 _	Acbd3
Sfr1	1.52E-19	0.312919	0.882	0.684	3.07E-15	0 L1 _	Sfr1
Ccl81	4.30E-20	0.398268	0.191	0.068	8.67E-16	0 L1 _	Ccl8
Glg11	3.68E-20	0.286973	0.798	0.561	7.41E-16	0 L1 _	Glg1
Prrg31	3.37E-20	0.266923	0.247	0.109	6.79E-16	0 L1 _	Prrg3
Vegfb	3.00E-20	0.272464	0.377	0.206	6.05E-16	0 L1 _	Vegfb
Ssh21	1.92E-20	0.258583	0.61	0.391	3.87E-16	0 L1 _	Ssh2
Irs21	1.87E-20	0.274087	0.297	0.142	3.78E-16	0 L1 _	Irs2
Svbp	1.46E-20	0.268379	0.625	0.422	2.94E-16	0 L1 _	Svbp
1700025G04Rik	1.38E-20	0.301204	0.603	0.393	2.77E-16	0 L1 _	1700025G04Rik
Trf1	1.32E-20	0.295855	0.372	0.195	2.66E-16	0 L1 _	Trf

Cebpb1	1.06E-20	0.311476	0.555	0.346	2.13E-16	0 L1 _	Cebpb
Selenok	8.02E-21	0.261249	0.929	0.782	1.62E-16	0 L1 _	Selenok
Cdc16	7.63E-21	0.255957	0.399	0.221	1.54E-16	0 L1 _	Cdc16
Cxxc5	5.52E-21	0.259985	0.469	0.281	1.11E-16	0 L1 _	Cxxc5
Kdelr11	3.77E-21	0.289052	0.77	0.554	7.60E-17	0 L1 _	Kdelr1
Runx1t11	3.54E-21	0.268239	0.426	0.239	7.14E-17	0 L1 _	Runx1t1
Tril1	2.50E-21	0.262195	0.585	0.363	5.03E-17	0 L1 _	Tril
Hmgcs1	2.40E-21	0.355503	0.375	0.207	4.84E-17	0 L1 _	Hmgcs1
Smad31	1.54E-21	0.275853	0.319	0.158	3.10E-17	0 L1 _	Smad3
Olfm1	1.22E-21	0.252538	0.444	0.251	2.47E-17	0 L1 _	Olfm1
Pnp1	1.01E-21	0.261646	0.73	0.484	2.03E-17	0 L1 _	Pnp
Tcf42	7.17E-22	0.339371	0.975	0.83	1.44E-17	0 L1 _	Tcf4
Fnbp11	4.15E-22	0.288331	0.665	0.44	8.36E-18	0 L1 _	Fnbp1
Tex264	3.72E-22	0.251855	0.577	0.36	7.48E-18	0 L1 _	Tex264
Sult5a11	3.44E-22	0.259445	0.243	0.099	6.92E-18	0 L1 _	Sult5a1
Slc38a101	3.04E-22	0.282089	0.538	0.334	6.12E-18	0 L1 _	Slc38a10
Yipf3	2.91E-22	0.257142	0.663	0.431	5.86E-18	0 L1 _	Yipf3
Ufc1	2.55E-22	0.265587	0.712	0.482	5.14E-18	0 L1 _	Ufc1
Ptn	2.39E-22	0.392363	0.174	0.051	4.80E-18	0 L1 _	Ptn
Rnf10	2.24E-22	0.251477	0.671	0.443	4.51E-18	0 L1 _	Rnf10
Iah1	2.03E-22	0.278792	0.511	0.309	4.09E-18	0 L1 _	Iah1
Dpy19l11	1.94E-22	0.28224	0.426	0.237	3.92E-18	0 L1 _	Dpy19l1
Clptm1l	1.69E-22	0.252353	0.553	0.353	3.40E-18	0 L1 _	Clptm1l
Sgcb1	1.61E-22	0.250534	0.452	0.26	3.25E-18	0 L1 _	Sgcb
Plod3	1.27E-22	0.256651	0.493	0.293	2.56E-18	0 L1 _	Plod3
Fign	1.23E-22	0.275755	0.262	0.11	2.48E-18	0 L1 _	Fign
Ccn1	1.10E-22	0.286291	0.227	0.084	2.22E-18	0 L1 _	Ccn1
Gpc4	1.02E-22	0.291207	0.263	0.111	2.05E-18	0 L1 _	Gpc4
Asah1	1.01E-22	0.26483	0.63	0.407	2.03E-18	0 L1 _	Asah1
Trim471	9.17E-23	0.272084	0.533	0.321	1.85E-18	0 L1 _	Trim47
Acvr1	8.84E-23	0.265286	0.303	0.141	1.78E-18	0 L1 _	Acvr1
Xbp11	8.16E-23	0.268084	0.748	0.506	1.64E-18	0 L1 _	Xbp1
Ccl19	7.04E-23	0.382558	0.173	0.049	1.42E-18	0 L1 _	Ccl19
Casp12	5.64E-23	0.281813	0.299	0.136	1.14E-18	0 L1 _	Casp12
Smarcb1	5.45E-23	0.259156	0.65	0.43	1.10E-18	0 L1 _	Smarcb1
Dio2	5.37E-23	0.321242	0.204	0.069	1.08E-18	0 L1 _	Dio2
Ang	5.11E-23	0.266325	0.28	0.12	1.03E-18	0 L1 _	Ang
C1qtnf6	4.14E-23	0.259474	0.442	0.248	8.34E-19	0 L1 _	C1qtnf6
Copg11	3.41E-23	0.304437	0.513	0.308	6.87E-19	0 L1 _	Copg1
Creld2	3.35E-23	0.274587	0.429	0.242	6.75E-19	0 L1 _	Creld2
Pcsk5	3.34E-23	0.325153	0.301	0.134	6.72E-19	0 L1 _	Pcsk5

Col4a3	3.15E-23	0.264496	0.218	0.079	6.34E-19	0 L1 _	Col4a3
Fkbp21	1.97E-23	0.316328	0.709	0.475	3.97E-19	0 L1 _	Fkbp2
Mcrip1	1.73E-23	0.314454	0.721	0.51	3.48E-19	0 L1 _	Mcrip1
Man2a11	1.42E-23	0.270258	0.715	0.458	2.86E-19	0 L1 _	Man2a1
Spred1	1.38E-23	0.281439	0.733	0.474	2.78E-19	0 L1 _	Spred1
Lrp61	1.34E-23	0.308409	0.589	0.365	2.70E-19	0 L1 _	Lrp6
Loxl21	1.33E-23	0.265551	0.457	0.244	2.69E-19	0 L1 _	Loxl2
St131	1.00E-23	0.321324	0.868	0.66	2.02E-19	0 L1 _	St13
Zeb1	7.45E-24	0.34569	0.742	0.516	1.50E-19	0 L1 _	Zeb1
Copa1	6.83E-24	0.299162	0.756	0.526	1.38E-19	0 L1 _	Copa
Asap11	6.19E-24	0.316423	0.76	0.522	1.25E-19	0 L1 _	Asap1
Mitf	4.55E-24	0.297219	0.302	0.136	9.16E-20	0 L1 _	Mitf
Tspan311	3.72E-24	0.280628	0.594	0.368	7.50E-20	0 L1 _	Tspan31
Ssc5d	2.95E-24	0.25459	0.236	0.088	5.94E-20	0 L1 _	Ssc5d
Mettl9	2.49E-24	0.259824	0.533	0.314	5.01E-20	0 L1 _	Mettl9
Fdps	2.28E-24	0.439952	0.378	0.197	4.59E-20	0 L1 _	Fdps
Serf2	2.09E-24	0.310459	0.972	0.932	4.20E-20	0 L1 _	Serf2
Atxn10	2.07E-24	0.254685	0.677	0.428	4.17E-20	0 L1 _	Atxn10
Ctsa	2.05E-24	0.285459	0.65	0.419	4.14E-20	0 L1 _	Ctsa
Cdkn2b	1.22E-24	0.251604	0.164	0.043	2.45E-20	0 L1 _	Cdkn2b
Lsp11	1.02E-24	0.334709	0.669	0.401	2.05E-20	0 L1 _	Lsp1
Rhoq1	1.00E-24	0.277989	0.543	0.32	2.02E-20	0 L1 _	Rhoq
Lamc31	9.54E-25	0.301445	0.444	0.228	1.92E-20	0 L1 _	Lamc3
Maf1	8.10E-25	0.265071	0.693	0.426	1.63E-20	0 L1 _	Maf
Myo101	7.37E-25	0.269899	0.601	0.353	1.49E-20	0 L1 _	Myo10
Synpo	7.32E-25	0.282973	0.472	0.26	1.47E-20	0 L1 _	Synpo
Pink1	7.14E-25	0.298326	0.638	0.415	1.44E-20	0 L1 _	Pink1
Naglu	7.01E-25	0.276239	0.301	0.134	1.41E-20	0 L1 _	Naglu
Rtn41	6.64E-25	0.325925	0.855	0.671	1.34E-20	0 L1 _	Rtn4
Sox4	6.16E-25	0.289551	0.584	0.343	1.24E-20	0 L1 _	Sox4
Bet1l	6.07E-25	0.323767	0.63	0.405	1.22E-20	0 L1 _	Bet1l
Mme1	5.77E-25	0.253092	0.253	0.093	1.16E-20	0 L1 _	Mme
Bmpr1a1	5.52E-25	0.292488	0.514	0.288	1.11E-20	0 L1 _	Bmpr1a
Pard6g	5.48E-25	0.281867	0.346	0.161	1.10E-20	0 L1 _	Pard6g
Gcsh	4.48E-25	0.263694	0.345	0.161	9.03E-21	0 L1 _	Gcsh
Nrxn11	4.13E-25	0.402682	0.208	0.066	8.31E-21	0 L1 _	Nrxn1
Pam1	4.02E-25	0.259884	0.674	0.423	8.10E-21	0 L1 _	Pam
1110065P20Rik	3.81E-25	0.250709	0.466	0.255	7.67E-21	0 L1 _	1110065P20Rik
Dnm11	2.67E-25	0.251967	0.329	0.148	5.37E-21	0 L1 _	Dnm1
Smad5	2.46E-25	0.357792	0.488	0.278	4.96E-21	0 L1 _	Smad5
Cdk8	2.15E-25	0.357108	0.497	0.281	4.34E-21	0 L1 _	Cdk8

Siva1	1.99E-25	0.294877	0.555	0.332	4.02E-21	0 L1 _	Siva1
Rab7b	1.81E-25	0.27483	0.298	0.129	3.64E-21	0 L1 _	Rab7b
Ssr4	1.74E-25	0.36512	0.883	0.714	3.51E-21	0 L1 _	Ssr4
Vmp1	1.08E-25	0.299458	0.565	0.34	2.18E-21	0 L1 _	Vmp1
App2	1.05E-25	0.282016	0.972	0.75	2.11E-21	0 L1 _	App
Rhod	9.76E-26	0.261997	0.269	0.103	1.97E-21	0 L1 _	Rhod
Il15	8.54E-26	0.277283	0.285	0.116	1.72E-21	0 L1 _	Il15
Gm300751	7.93E-26	0.327801	0.259	0.099	1.60E-21	0 L1 _	Gm30075
Map1lc3b	6.89E-26	0.309047	0.923	0.804	1.39E-21	0 L1 _	Map1lc3b
Sqle1	6.48E-26	0.315338	0.263	0.102	1.30E-21	0 L1 _	Sqle
Lefty1	6.25E-26	0.250901	0.247	0.092	1.26E-21	0 L1 _	Lefty1
Tslp1	5.86E-26	0.276434	0.238	0.085	1.18E-21	0 L1 _	Tslp
Serpinb6a2	4.96E-26	0.27088	0.861	0.613	9.98E-22	0 L1 _	Serpinb6a
Atp1a21	4.19E-26	0.301899	0.361	0.166	8.43E-22	0 L1 _	Atp1a2
Smo	4.18E-26	0.272541	0.438	0.227	8.42E-22	0 L1 _	Smo
Tnfsf12	4.00E-26	0.310075	0.788	0.559	8.06E-22	0 L1 _	Tnfsf12
Atp5o1	3.55E-26	0.279946	0.806	0.492	7.16E-22	0 L1 _	Atp5o
Tmem97	3.10E-26	0.255978	0.317	0.138	6.24E-22	0 L1 _	Tmem97
Tceal9	2.74E-26	0.288085	0.849	0.625	5.52E-22	0 L1 _	Tceal9
Srpr	2.64E-26	0.347801	0.764	0.541	5.33E-22	0 L1 _	Srpr
Susd2	2.53E-26	0.276367	0.249	0.089	5.09E-22	0 L1 _	Susd2
Tmem9	2.41E-26	0.252284	0.383	0.186	4.86E-22	0 L1 _	Tmem9
Tmed9	2.38E-26	0.321306	0.886	0.707	4.80E-22	0 L1 _	Tmed9
Usp9x1	2.19E-26	0.326513	0.673	0.434	4.40E-22	0 L1 _	Usp9x
Creb3l2	2.12E-26	0.358585	0.564	0.341	4.27E-22	0 L1 _	Creb3l2
Herpud11	2.09E-26	0.299825	0.708	0.442	4.21E-22	0 L1 _	Herpud1
Tomm6	1.98E-26	0.260664	0.77	0.492	3.98E-22	0 L1 _	Tomm6
Tmem59	1.80E-26	0.295274	0.935	0.834	3.62E-22	0 L1 _	Tmem59
Magi2	1.70E-26	0.258134	0.347	0.154	3.43E-22	0 L1 _	Magi2
Sigmar1	1.61E-26	0.345736	0.464	0.258	3.25E-22	0 L1 _	Sigmar1
Oasl2	1.61E-26	0.368031	0.476	0.257	3.24E-22	0 L1 _	Oasl2
Layn1	1.04E-26	0.295179	0.374	0.181	2.10E-22	0 L1 _	Layn
1500011B03Rik	9.93E-27	0.251454	0.321	0.138	2.00E-22	0 L1 _	1500011B03Rik
Sra1	5.76E-27	0.335271	0.688	0.46	1.16E-22	0 L1 _	Sra1
Parp3	4.87E-27	0.302002	0.581	0.349	9.81E-23	0 L1 _	Parp3
Gulp11	4.19E-27	0.299604	0.501	0.275	8.43E-23	0 L1 _	Gulp1
Spcs1	2.65E-27	0.318497	0.844	0.658	5.34E-23	0 L1 _	Spcs1
Hist1h2bc1	2.62E-27	0.330413	0.635	0.397	5.27E-23	0 L1 _	Hist1h2bc
Bace2	2.37E-27	0.281911	0.339	0.149	4.78E-23	0 L1 _	Bace2
Ubtd1	2.06E-27	0.2694	0.325	0.138	4.16E-23	0 L1 _	Ubtd1
Gadd45b	1.56E-27	0.450121	0.462	0.245	3.14E-23	0 L1 _	Gadd45b

Glmp	1.39E-27	0.291119	0.672	0.421	2.79E-23	0 L1 _	Glmp
Rnd31	1.31E-27	0.380772	0.515	0.29	2.63E-23	0 L1 _	Rnd3
Atraid	1.30E-27	0.311202	0.802	0.59	2.61E-23	0 L1 _	Atraid
Gpnmb	9.30E-28	0.28622	0.175	0.042	1.87E-23	0 L1 _	Gpnmb
Ptov1	8.55E-28	0.257406	0.645	0.389	1.72E-23	0 L1 _	Ptov1
Tubb2b	7.97E-28	0.299681	0.216	0.065	1.60E-23	0 L1 _	Tubb2b
Dock71	7.13E-28	0.294577	0.453	0.238	1.44E-23	0 L1 _	Dock7
Rhoj2	3.74E-28	0.352309	0.89	0.642	7.53E-24	0 L1 _	Rhoj
Rab2a	2.84E-28	0.348772	0.906	0.733	5.73E-24	0 L1 _	Rab2a
Itga81	2.54E-28	0.352501	0.31	0.13	5.11E-24	0 L1 _	Itga8
Adprh	2.19E-28	0.337465	0.695	0.466	4.41E-24	0 L1 _	Adprh
Sar1a1	1.59E-28	0.384565	0.729	0.494	3.20E-24	0 L1 _	Sar1a
Ror1	1.56E-28	0.259321	0.313	0.125	3.15E-24	0 L1 _	Ror1
Pbx11	1.42E-28	0.368083	0.685	0.416	2.86E-24	0 L1 _	Pbx1
Kctd11	1.40E-28	0.25456	0.299	0.115	2.81E-24	0 L1 _	Kctd11
Tram11	6.90E-29	0.350835	0.748	0.504	1.39E-24	0 L1 _	Tram1
Naxd	6.08E-29	0.277608	0.62	0.381	1.23E-24	0 L1 _	Naxd
Ttc3	5.33E-29	0.369448	0.721	0.475	1.07E-24	0 L1 _	Ttc3
Nid21	5.02E-29	0.263262	0.517	0.264	1.01E-24	0 L1 _	Nid2
Fgf2	4.69E-29	0.280301	0.322	0.134	9.44E-25	0 L1 _	Fgf2
Tmed1	4.24E-29	0.293251	0.487	0.257	8.54E-25	0 L1 _	Tmed1
Sec24d1	3.42E-29	0.327657	0.45	0.236	6.89E-25	0 L1 _	Sec24d
Ggcx	2.89E-29	0.269503	0.362	0.162	5.82E-25	0 L1 _	Ggcx
Stt3a1	2.51E-29	0.326671	0.628	0.366	5.05E-25	0 L1 _	Stt3a
Hmox1	2.21E-29	0.268725	0.255	0.089	4.46E-25	0 L1 _	Hmox1
Rgma1	2.15E-29	0.274422	0.528	0.277	4.32E-25	0 L1 _	Rgma
Piezo21	1.76E-29	0.328931	0.51	0.258	3.54E-25	0 L1 _	Piezo2
Ro60	1.39E-29	0.312108	0.425	0.202	2.80E-25	0 L1 _	Ro60
Rab3il1	8.57E-30	0.323038	0.38	0.183	1.73E-25	0 L1 _	Rab3il1
Ahnak1	7.76E-30	0.360577	0.975	0.841	1.56E-25	0 L1 _	Ahnak
Icam1	6.48E-30	0.349442	0.41	0.198	1.31E-25	0 L1 _	Icam1
Ift20	5.77E-30	0.326757	0.808	0.574	1.16E-25	0 L1 _	Ift20
Ddost	5.09E-30	0.37825	0.79	0.539	1.03E-25	0 L1 _	Ddost
Sertad4	4.94E-30	0.292628	0.258	0.087	9.94E-26	0 L1 _	Sertad4
Tmem167	3.52E-30	0.361852	0.786	0.565	7.08E-26	0 L1 _	Tmem167
Pofut21	3.11E-30	0.317925	0.611	0.356	6.27E-26	0 L1 _	Pofut2
Col28a1	3.03E-30	0.379532	0.176	0.038	6.10E-26	0 L1 _	Col28a1
Anxa52	2.99E-30	0.326196	0.961	0.837	6.03E-26	0 L1 _	Anxa5
Ifi2071	2.64E-30	0.319604	0.481	0.238	5.31E-26	0 L1 _	Ifi207
Cbr31	2.37E-30	0.30862	0.504	0.262	4.78E-26	0 L1 _	Cbr3
S1pr21	2.30E-30	0.306245	0.373	0.167	4.63E-26	0 L1 _	S1pr2

Cyb5a1	2.02E-30	0.384345	0.9	0.684	4.07E-26	0 L1 _	Cyb5a
Lrrc581	1.68E-30	0.386142	0.827	0.567	3.38E-26	0 L1 _	Lrrc58
Igsf31	1.60E-30	0.327991	0.357	0.149	3.23E-26	0 L1 _	Igsf3
Tm7sf3	1.28E-30	0.347559	0.481	0.254	2.58E-26	0 L1 _	Tm7sf3
Dse1	9.20E-31	0.312441	0.412	0.198	1.85E-26	0 L1 _	Dse
Art4	6.58E-31	0.345157	0.22	0.061	1.32E-26	0 L1 _	Art4
Atp6ap1	6.12E-31	0.373285	0.726	0.462	1.23E-26	0 L1 _	Atp6ap1
Sec61g1	5.17E-31	0.405536	0.927	0.814	1.04E-26	0 L1 _	Sec61g
Tnfrsf19	5.06E-31	0.263608	0.164	0.03	1.02E-26	0 L1 _	Tnfrsf19
Ltbp11	5.00E-31	0.431057	0.566	0.314	1.01E-26	0 L1 _	Ltbp1
Rnf150	4.52E-31	0.308283	0.314	0.121	9.10E-27	0 L1 _	Rnf150
Tubb2a	4.36E-31	0.467919	0.581	0.338	8.78E-27	0 L1 _	Tubb2a
Tspan4	2.94E-31	0.35287	0.605	0.352	5.92E-27	0 L1 _	Tspan4
Tnfrsf12a	2.75E-31	0.472882	0.252	0.081	5.54E-27	0 L1 _	Tnfrsf12a
Tm2d21	2.72E-31	0.318905	0.642	0.391	5.47E-27	0 L1 _	Tm2d2
Ldlr	2.16E-31	0.336384	0.356	0.15	4.36E-27	0 L1 _	Ldlr
Dsel1	2.13E-31	0.354192	0.455	0.226	4.29E-27	0 L1 _	Dsel
Fscn1	1.76E-31	0.277231	0.481	0.239	3.55E-27	0 L1 _	Fscn1
Sh3pxd2b1	1.62E-31	0.318067	0.474	0.229	3.26E-27	0 L1 _	Sh3pxd2b
Itih2	1.62E-31	0.256243	0.182	0.039	3.25E-27	0 L1 _	Itih2
Slc1a5	1.33E-31	0.365237	0.658	0.399	2.67E-27	0 L1 _	Slc1a5
Fgl21	1.30E-31	0.493045	0.557	0.31	2.62E-27	0 L1 _	Fgl2
Cul1	8.69E-32	0.400327	0.739	0.495	1.75E-27	0 L1 _	Cul1
Mocs2	7.60E-32	0.312141	0.619	0.366	1.53E-27	0 L1 _	Mocs2
Surf41	6.47E-32	0.371374	0.709	0.441	1.30E-27	0 L1 _	Surf4
Rcn2	6.11E-32	0.296443	0.658	0.39	1.23E-27	0 L1 _	Rcn2
Shox21	3.42E-32	0.326858	0.386	0.166	6.90E-28	0 L1 _	Shox2
Sgce1	3.29E-32	0.293746	0.586	0.314	6.62E-28	0 L1 _	Sgce
Nr2f12	3.16E-32	0.274628	0.8	0.465	6.37E-28	0 L1 _	Nr2f1
2900097C17Rik1	3.08E-32	0.360997	0.717	0.446	6.20E-28	0 L1 _	2900097C17Rik
Rgmb1	3.04E-32	0.386333	0.438	0.215	6.13E-28	0 L1 _	Rgmb
Sec22b	2.89E-32	0.300287	0.566	0.322	5.83E-28	0 L1 _	Sec22b
Pthlh	2.65E-32	0.404841	0.249	0.078	5.34E-28	0 L1 _	Pthlh
Scml4	2.49E-32	0.321786	0.294	0.107	5.02E-28	0 L1 _	Scml4
Fndc3b1	2.26E-32	0.312099	0.71	0.413	4.55E-28	0 L1 _	Fndc3b
Mettl11	2.11E-32	0.369095	0.421	0.202	4.24E-28	0 L1 _	Mettl1
Ndfip11	2.09E-32	0.326261	0.88	0.621	4.21E-28	0 L1 _	Ndfip1
Zfyve21	1.99E-32	0.275414	0.462	0.219	4.02E-28	0 L1 _	Zfyve21
Bnc2	1.69E-32	0.314359	0.264	0.087	3.40E-28	0 L1 _	Bnc2
Ext11	1.33E-32	0.358087	0.515	0.272	2.68E-28	0 L1 _	Ext1
P2rx4	1.11E-32	0.317812	0.473	0.233	2.23E-28	0 L1 _	P2rx4

Plbd2	1.07E-32	0.353692	0.49	0.252	2.15E-28	0 L1 _	Plbd2
Fkbp101	1.02E-32	0.297084	0.443	0.214	2.06E-28	0 L1 _	Fkbp10
Itga2b	9.89E-33	0.298974	0.269	0.089	1.99E-28	0 L1 _	Itga2b
Colgalt1	7.77E-33	0.388645	0.702	0.445	1.57E-28	0 L1 _	Colgalt1
Tcea3	6.05E-33	0.296314	0.258	0.08	1.22E-28	0 L1 _	Tcea3
Myh101	5.70E-33	0.36138	0.361	0.151	1.15E-28	0 L1 _	Myh10
Swi5	5.51E-33	0.375544	0.887	0.689	1.11E-28	0 L1 _	Swi5
Apbb1ip1	4.28E-33	0.365905	0.61	0.335	8.63E-29	0 L1 _	Apbb1ip
Rbis	3.86E-33	0.272803	0.622	0.329	7.77E-29	0 L1 _	Rbis
Csf11	2.95E-33	0.384718	0.549	0.298	5.94E-29	0 L1 _	Csf1
Boc1	2.93E-33	0.337575	0.332	0.132	5.91E-29	0 L1 _	Boc
Calu1	2.18E-33	0.37926	0.809	0.513	4.39E-29	0 L1 _	Calu
Gpx31	2.16E-33	0.707527	0.551	0.284	4.36E-29	0 L1 _	Gpx3
Ap3s1	1.85E-33	0.34379	0.54	0.297	3.73E-29	0 L1 _	Ap3s1
Pkig1	1.78E-33	0.384395	0.777	0.514	3.58E-29	0 L1 _	Pkig
Serpine1	1.59E-33	0.490481	0.223	0.058	3.20E-29	0 L1 _	Serpine1
Czib	1.25E-33	0.272375	0.442	0.199	2.53E-29	0 L1 _	Czib
Bsg	1.13E-33	0.358594	0.933	0.775	2.28E-29	0 L1 _	Bsg
Gab2	1.12E-33	0.330537	0.37	0.155	2.25E-29	0 L1 _	Gab2
Dclk11	1.00E-33	0.388478	0.344	0.136	2.01E-29	0 L1 _	Dclk1
Eif1a	8.73E-34	0.353354	0.617	0.366	1.76E-29	0 L1 _	Eif1a
Ghr	7.38E-34	0.340721	0.429	0.2	1.49E-29	0 L1 _	Ghr
Lman11	5.91E-34	0.369935	0.781	0.525	1.19E-29	0 L1 _	Lman1
Antxr11	5.81E-34	0.31791	0.45	0.21	1.17E-29	0 L1 _	Antxr1
Tgfbr1	4.93E-34	0.326	0.551	0.302	9.93E-30	0 L1 _	Tgfbr1
Cst3	4.82E-34	0.738882	0.983	0.933	9.71E-30	0 L1 _	Cst3
Dnajc31	2.40E-34	0.407233	0.86	0.615	4.83E-30	0 L1 _	Dnajc3
Sema3a1	2.27E-34	0.377966	0.307	0.108	4.56E-30	0 L1 _	Sema3a
Chic2	2.25E-34	0.375521	0.718	0.452	4.54E-30	0 L1 _	Chic2
Bace11	2.20E-34	0.334104	0.525	0.266	4.42E-30	0 L1 _	Bace1
Emp31	1.61E-34	0.407318	0.926	0.725	3.25E-30	0 L1 _	Emp3
Hsp90b11	1.35E-34	0.368895	0.965	0.845	2.71E-30	0 L1 _	Hsp90b1
Gm2a	4.97E-35	0.351974	0.529	0.273	1.00E-30	0 L1 _	Gm2a
Yipf51	4.41E-35	0.368495	0.65	0.386	8.88E-31	0 L1 _	Yipf5
Slc7a2	3.72E-35	0.329619	0.368	0.141	7.49E-31	0 L1 _	Slc7a2
Abat	3.08E-35	0.328223	0.314	0.114	6.21E-31	0 L1 _	Abat
Igfbp71	2.92E-35	0.339385	0.992	0.912	5.87E-31	0 L1 _	Igfbp7
Twsg11	2.70E-35	0.371901	0.535	0.274	5.44E-31	0 L1 _	Twsg1
Odr4	1.97E-35	0.320081	0.435	0.188	3.97E-31	0 L1 _	Odr4
Slc39a13	1.94E-35	0.327492	0.501	0.25	3.90E-31	0 L1 _	Slc39a13
Wipi11	1.54E-35	0.343704	0.561	0.291	3.09E-31	0 L1 _	Wipi1

Ap2a2	1.28E-35	0.415372	0.695	0.433	2.58E-31	0 L1 _	Ap2a2
Gm26682	1.14E-35	0.31179	0.224	0.053	2.30E-31	0 L1 _	Gm26682
Nnmt	1.11E-35	0.307821	0.449	0.2	2.24E-31	0 L1 _	Nnmt
Plpp5	1.02E-35	0.342683	0.39	0.17	2.06E-31	0 L1 _	Plpp5
Enho	9.72E-36	0.362781	0.345	0.13	1.96E-31	0 L1 _	Enho
Csad	8.31E-36	0.330284	0.438	0.205	1.67E-31	0 L1 _	Csad
Arf5	6.88E-36	0.428006	0.893	0.72	1.38E-31	0 L1 _	Arf5
Pld3	6.57E-36	0.356913	0.593	0.329	1.32E-31	0 L1 _	Pld3
Erp29	5.63E-36	0.380619	0.815	0.572	1.13E-31	0 L1 _	Erp29
Myo7a	4.10E-36	0.29052	0.224	0.054	8.26E-32	0 L1 _	Myo7a
Mir22hg	2.78E-36	0.304102	0.274	0.08	5.60E-32	0 L1 _	Mir22hg
Tmed10	2.67E-36	0.38526	0.934	0.776	5.37E-32	0 L1 _	Tmed10
Tmem2631	2.52E-36	0.345058	0.472	0.227	5.07E-32	0 L1 _	Tmem263
Robo11	1.77E-36	0.337397	0.361	0.141	3.56E-32	0 L1 _	Robo1
Cnn3	1.60E-36	0.403368	0.777	0.512	3.23E-32	0 L1 _	Cnn3
Ctsd1	1.18E-36	0.363363	0.947	0.765	2.37E-32	0 L1 _	Ctsd
Fam102b1	1.17E-36	0.379626	0.47	0.212	2.35E-32	0 L1 _	Fam102b
Atp6v0c	1.05E-36	0.370607	0.885	0.541	2.12E-32	0 L1 _	Atp6v0c
Lmna1	8.64E-37	0.443007	0.847	0.593	1.74E-32	0 L1 _	Lmna
Sned11	8.58E-37	0.494788	0.511	0.249	1.73E-32	0 L1 _	Sned1
Yif1a	7.74E-37	0.359983	0.543	0.285	1.56E-32	0 L1 _	Yif1a
Dap	7.60E-37	0.433961	0.663	0.401	1.53E-32	0 L1 _	Dap
Tmem223	6.80E-37	0.313339	0.542	0.267	1.37E-32	0 L1 _	Tmem223
Ifitm3	6.26E-37	0.423562	0.991	0.905	1.26E-32	0 L1 _	Ifitm3
Clcn51	6.13E-37	0.352832	0.384	0.153	1.24E-32	0 L1 _	Clcn5
Anxa21	5.81E-37	0.451283	0.929	0.794	1.17E-32	0 L1 _	Anxa2
Ifngr11	5.71E-37	0.424423	0.888	0.629	1.15E-32	0 L1 _	Ifngr1
Tiam1	5.51E-37	0.306455	0.243	0.063	1.11E-32	0 L1 _	Tiam1
Ryk	4.32E-37	0.408056	0.678	0.41	8.71E-33	0 L1 _	Ryk
Aldh7a11	3.57E-37	0.343624	0.508	0.242	7.18E-33	0 L1 _	Aldh7a1
Npc2	2.43E-37	0.432293	0.882	0.668	4.89E-33	0 L1 _	Npc2
Dipk1b	2.42E-37	0.273078	0.257	0.069	4.88E-33	0 L1 _	Dipk1b
Echdc2	2.16E-37	0.286134	0.236	0.059	4.36E-33	0 L1 _	Echdc2
Slc8a1	1.54E-37	0.331766	0.264	0.073	3.11E-33	0 L1 _	Slc8a1
Axl2	1.51E-37	0.322925	0.781	0.443	3.03E-33	0 L1 _	Axl
Desi2	1.43E-37	0.367016	0.614	0.342	2.88E-33	0 L1 _	Desi2
Tnfaip2	1.43E-37	0.503769	0.59	0.321	2.88E-33	0 L1 _	Tnfaip2
Nrxn21	1.31E-37	0.445505	0.43	0.18	2.64E-33	0 L1 _	Nrxn2
Dapk11	1.11E-37	0.363171	0.534	0.249	2.24E-33	0 L1 _	Dapk1
Lgmn	1.01E-37	0.321089	0.563	0.283	2.04E-33	0 L1 _	Lgmn
Slc39a7	9.29E-38	0.363748	0.611	0.345	1.87E-33	0 L1 _	Slc39a7

Cdk141	8.79E-38	0.444507	0.581	0.312	1.77E-33	0 L1 _	Cdk14
Cks1b	8.19E-38	0.305026	0.337	0.118	1.65E-33	0 L1 _	Cks1b
Golim41	4.80E-38	0.406181	0.745	0.466	9.67E-34	0 L1 _	Golim4
Celf21	3.37E-38	0.381368	0.784	0.438	6.79E-34	0 L1 _	Celf2
Dact3	3.20E-38	0.338404	0.346	0.125	6.44E-34	0 L1 _	Dact3
Krtcap2	2.17E-38	0.378432	0.919	0.756	4.36E-34	0 L1 _	Krtcap2
Nr1d1	2.12E-38	0.476857	0.468	0.22	4.28E-34	0 L1 _	Nr1d1
Egflam	9.44E-39	0.314591	0.607	0.309	1.90E-34	0 L1 _	Egflam
Tmem176a	8.18E-39	0.470658	0.94	0.73	1.65E-34	0 L1 _	Tmem176a
Lix1l	7.30E-39	0.376286	0.577	0.312	1.47E-34	0 L1 _	Lix1l
Nrep	6.88E-39	0.562221	0.438	0.201	1.39E-34	0 L1 _	Nrep
Arhgap6	6.75E-39	0.402554	0.589	0.305	1.36E-34	0 L1 _	Arhgap6
Lamc11	6.65E-39	0.374548	0.807	0.482	1.34E-34	0 L1 _	Lamc1
Cyp51	6.48E-39	0.457675	0.351	0.13	1.31E-34	0 L1 _	Cyp51
Prrc1	4.66E-39	0.416478	0.549	0.286	9.38E-35	0 L1 _	Prrc1
Zbtb201	4.61E-39	0.452824	0.973	0.851	9.29E-35	0 L1 _	Zbtb20
Cacna1g	3.98E-39	0.349742	0.285	0.085	8.02E-35	0 L1 _	Cacna1g
Postn1	3.41E-39	0.799799	0.632	0.36	6.86E-35	0 L1 _	Postn
Pcdh181	1.73E-39	0.393057	0.623	0.328	3.48E-35	0 L1 _	Pcdh18
Olfm2	1.69E-39	0.3483	0.201	0.035	3.41E-35	0 L1 _	Olfm2
Actn1	1.61E-39	0.347164	0.675	0.375	3.24E-35	0 L1 _	Actn1
Adgra1	1.37E-39	0.425652	0.197	0.032	2.76E-35	0 L1 _	Adgra1
Idh11	1.32E-39	0.380009	0.511	0.244	2.66E-35	0 L1 _	Idh1
Slc39a11	5.13E-40	0.440676	0.613	0.337	1.03E-35	0 L1 _	Slc39a1
Phyhd1	4.48E-40	0.293759	0.257	0.063	9.02E-36	0 L1 _	Phyhd1
Ptges1	3.29E-40	0.386806	0.41	0.165	6.63E-36	0 L1 _	Ptges
Eri3	3.14E-40	0.33489	0.479	0.218	6.33E-36	0 L1 _	Eri3
Itgav1	3.07E-40	0.464908	0.652	0.368	6.19E-36	0 L1 _	Itgav
H6pd1	2.34E-40	0.41147	0.455	0.201	4.72E-36	0 L1 _	H6pd
Scd21	2.14E-40	0.3689	0.711	0.392	4.30E-36	0 L1 _	Scd2
Runx2	1.83E-40	0.376752	0.369	0.141	3.69E-36	0 L1 _	Runx2
Morf4l21	1.78E-40	0.424583	0.758	0.471	3.58E-36	0 L1 _	Morf4l2
Naalad21	1.39E-40	0.372571	0.666	0.35	2.79E-36	0 L1 _	Naalad2
Rftn1	1.07E-40	0.453884	0.643	0.37	2.15E-36	0 L1 _	Rftn1
Wnt9a	9.95E-41	0.391846	0.226	0.048	2.00E-36	0 L1 _	Wnt9a
Mmp191	6.53E-41	0.301026	0.472	0.197	1.32E-36	0 L1 _	Mmp19
Calhm5	5.47E-41	0.330681	0.251	0.059	1.10E-36	0 L1 _	Calhm5
Plod11	5.31E-41	0.412097	0.736	0.42	1.07E-36	0 L1 _	Plod1
Pkd21	4.64E-41	0.442724	0.712	0.393	9.35E-37	0 L1 _	Pkd2
Micos10	4.13E-41	0.382826	0.834	0.482	8.32E-37	0 L1 _	Micos10
Robo2	2.47E-41	0.431018	0.302	0.093	4.96E-37	0 L1 _	Robo2

Laptm4a1	2.39E-41	0.33044	0.983	0.887	4.82E-37	0 L1 _	Laptm4a
Fxyd11	1.49E-41	0.342776	0.884	0.539	3.00E-37	0 L1 _	Fxyd1
Rspo31	7.31E-42	0.486007	0.433	0.167	1.47E-37	0 L1 _	Rspo3
Akr1b8	6.17E-42	0.357812	0.371	0.133	1.24E-37	0 L1 _	Akr1b8
P3h1	4.64E-42	0.378762	0.395	0.151	9.35E-38	0 L1 _	P3h1
Mpg	3.93E-42	0.390685	0.499	0.234	7.91E-38	0 L1 _	Mpg
Plod21	3.19E-42	0.448834	0.471	0.21	6.42E-38	0 L1 _	Plod2
Manf	2.55E-42	0.612428	0.81	0.595	5.14E-38	0 L1 _	Manf
Gng10	1.81E-42	0.407547	0.535	0.256	3.65E-38	0 L1 _	Gng10
Sesn3	1.80E-42	0.451399	0.658	0.377	3.63E-38	0 L1 _	Sesn3
Stc2	1.63E-42	0.374943	0.242	0.051	3.28E-38	0 L1 _	Stc2
Adamts22	1.41E-42	0.363511	0.474	0.197	2.84E-38	0 L1 _	Adamts2
Lgals3bp	1.34E-42	0.488085	0.8	0.499	2.70E-38	0 L1 _	Lgals3bp
Rxylt1	1.33E-42	0.317821	0.536	0.231	2.68E-38	0 L1 _	Rxylt1
Cpz	1.04E-42	0.389758	0.198	0.029	2.09E-38	0 L1 _	Cpz
Dpp7	7.87E-43	0.332773	0.348	0.115	1.59E-38	0 L1 _	Dpp7
Fam20c	6.85E-43	0.348723	0.315	0.097	1.38E-38	0 L1 _	Fam20c
Tmem176b	5.77E-43	0.526158	0.96	0.768	1.16E-38	0 L1 _	Tmem176b
Arl11	4.05E-43	0.46719	0.818	0.559	8.15E-39	0 L1 _	Arl1
Tspan33	4.03E-43	0.384009	0.845	0.547	8.11E-39	0 L1 _	Tspan3
Sspn1	3.80E-43	0.406127	0.523	0.241	7.65E-39	0 L1 _	Sspn
Antxr22	2.27E-43	0.292113	0.589	0.264	4.57E-39	0 L1 _	Antxr2
Dnajc251	2.26E-43	0.429266	0.492	0.224	4.55E-39	0 L1 _	Dnajc25
Serpina3n	2.16E-43	0.565649	0.306	0.088	4.34E-39	0 L1 _	Serpina3n
Adamts10	1.98E-43	0.486734	0.6	0.311	3.98E-39	0 L1 _	Adamts10
P4ha11	1.95E-43	0.437917	0.669	0.368	3.93E-39	0 L1 _	P4ha1
Fads11	1.90E-43	0.420001	0.473	0.205	3.82E-39	0 L1 _	Fads1
Plekhf11	1.38E-43	0.380971	0.404	0.154	2.78E-39	0 L1 _	Plekhf1
Col11a1	1.13E-43	0.411844	0.223	0.04	2.28E-39	0 L1 _	Col11a1
Ostc	1.09E-43	0.500587	0.816	0.585	2.19E-39	0 L1 _	Ostc
Tafa5	9.81E-44	0.277909	0.207	0.032	1.98E-39	0 L1 _	Tafa5
Timm23	8.59E-44	0.397747	0.587	0.282	1.73E-39	0 L1 _	Timm23
Sdc41	5.72E-44	0.51043	0.532	0.251	1.15E-39	0 L1 _	Sdc4
Ccpg1	3.37E-44	0.421135	0.612	0.311	6.79E-40	0 L1 _	Ccpg1
Tmem150a	3.31E-44	0.361482	0.387	0.141	6.66E-40	0 L1 _	Tmem150a
Prxl2c	2.38E-44	0.373297	0.49	0.205	4.80E-40	0 L1 _	Prxl2c
Thbs11	1.71E-44	0.570495	0.405	0.151	3.45E-40	0 L1 _	Thbs1
Fkbp11	1.32E-44	0.375738	0.325	0.099	2.65E-40	0 L1 _	Fkbp11
Slc29a11	1.27E-44	0.488532	0.803	0.486	2.55E-40	0 L1 _	Slc29a1
Slc36a2	1.01E-44	0.335272	0.241	0.047	2.03E-40	0 L1 _	Slc36a2
Msx1	7.27E-45	0.478827	0.419	0.171	1.46E-40	0 L1 _	Msx1

Slit31	5.07E-45	0.445498	0.555	0.262	1.02E-40	0 L1 _	Slit3
Fbln51	2.20E-45	0.297222	0.577	0.262	4.43E-41	0 L1 _	Fbln5
P2ry11	2.13E-45	0.440015	0.426	0.159	4.29E-41	0 L1 _	P2ry1
Hmgn3	1.43E-45	0.475058	0.689	0.39	2.87E-41	0 L1 _	Hmgn3
Scarb21	1.41E-45	0.438167	0.734	0.415	2.85E-41	0 L1 _	Scarb2
Mmp231	1.07E-45	0.388527	0.351	0.112	2.15E-41	0 L1 _	Mmp23
Myo1d	9.67E-46	0.456817	0.443	0.178	1.95E-41	0 L1 _	Myo1d
Ifi27l2a	7.11E-46	0.62753	0.793	0.479	1.43E-41	0 L1 _	Ifi27l2a
Penk1	5.13E-46	0.848753	0.401	0.148	1.03E-41	0 L1 _	Penk
Rcn1	4.13E-46	0.47059	0.732	0.432	8.32E-42	0 L1 _	Rcn1
Tmem98	4.13E-46	0.297289	0.272	0.062	8.32E-42	0 L1 _	Tmem98
Arhgef401	3.77E-46	0.496255	0.63	0.333	7.60E-42	0 L1 _	Arhgef40
Gata6	2.90E-46	0.375132	0.346	0.107	5.84E-42	0 L1 _	Gata6
Tmem200a	2.15E-46	0.328866	0.22	0.034	4.33E-42	0 L1 _	Tmem200a
Casc4	1.47E-46	0.405641	0.563	0.262	2.95E-42	0 L1 _	Casc4
Poglut3	1.24E-46	0.380757	0.407	0.142	2.50E-42	0 L1 _	Poglut3
Arf4	1.05E-46	0.482856	0.899	0.679	2.11E-42	0 L1 _	Arf4
Lamp1	8.50E-47	0.444617	0.959	0.804	1.71E-42	0 L1 _	Lamp1
Ifi2041	7.83E-47	0.475572	0.535	0.239	1.58E-42	0 L1 _	Ifi204
Wwtr1	7.22E-47	0.482602	0.772	0.472	1.45E-42	0 L1 _	Wwtr1
Rin2	4.50E-47	0.548286	0.597	0.306	9.05E-43	0 L1 _	Rin2
Ikbip1	4.32E-47	0.413051	0.672	0.345	8.70E-43	0 L1 _	Ikbip
Cgnl1	3.80E-47	0.49747	0.421	0.164	7.65E-43	0 L1 _	Cgnl1
Tmem158	3.64E-47	0.487017	0.423	0.161	7.33E-43	0 L1 _	Tmem158
Fuca1	1.48E-47	0.458091	0.767	0.462	2.97E-43	0 L1 _	Fuca1
Tcf7l21	1.36E-47	0.532046	0.65	0.342	2.73E-43	0 L1 _	Tcf7l2
Sh3d191	1.27E-47	0.457119	0.6	0.284	2.56E-43	0 L1 _	Sh3d19
Ak1	1.08E-47	0.426743	0.315	0.087	2.18E-43	0 L1 _	Ak1
Fam20a	1.00E-47	0.422065	0.444	0.174	2.02E-43	0 L1 _	Fam20a
Ecm11	7.94E-48	0.400653	0.677	0.352	1.60E-43	0 L1 _	Ecm1
Gabarap	5.32E-48	0.464473	0.942	0.847	1.07E-43	0 L1 _	Gabarap
Grn	3.92E-48	0.505742	0.861	0.562	7.89E-44	0 L1 _	Grn
Tmem2041	3.64E-48	0.545822	0.773	0.448	7.34E-44	0 L1 _	Tmem204
Nfic1	1.22E-48	0.491767	0.895	0.596	2.47E-44	0 L1 _	Nfic
Gm10115	9.85E-49	0.355361	0.236	0.038	1.98E-44	0 L1 _	Gm10115
Ptprd	9.07E-49	0.469522	0.349	0.104	1.83E-44	0 L1 _	Ptprd
Frem1	4.67E-49	0.395761	0.287	0.067	9.41E-45	0 L1 _	Frem1
Igfbp51	3.49E-49	0.815748	0.709	0.388	7.02E-45	0 L1 _	Igfbp5
Chp2	1.04E-49	0.564202	0.325	0.092	2.09E-45	0 L1 _	Chp2
Nucb21	1.02E-49	0.487981	0.594	0.285	2.05E-45	0 L1 _	Nucb2
Gng12	9.45E-50	0.499341	0.727	0.413	1.90E-45	0 L1 _	Gng12

Twist11	8.05E-50	0.437152	0.682	0.343	1.62E-45	0 L1 _	Twist1
Gm17167	4.89E-50	0.483071	0.272	0.055	9.84E-46	0 L1 _	Gm17167
Fat1	4.89E-50	0.452986	0.427	0.152	9.84E-46	0 L1 _	Fat1
Kdelr21	6.92E-51	0.520452	0.748	0.455	1.39E-46	0 L1 _	Kdelr2
Medag2	5.41E-51	0.454756	0.518	0.207	1.09E-46	0 L1 _	Medag
Vcan1	5.08E-51	0.661805	0.481	0.19	1.02E-46	0 L1 _	Vcan
Selenom1	4.60E-51	0.521262	0.952	0.735	9.27E-47	0 L1 _	Selenom
Runx1	4.28E-51	0.56586	0.496	0.205	8.62E-47	0 L1 _	Runx1
Tpbg	2.04E-51	0.418354	0.322	0.087	4.11E-47	0 L1 _	Tpbg
P4hb1	1.43E-51	0.517861	0.869	0.577	2.89E-47	0 L1 _	P4hb
Hmgn1	3.55E-52	0.543578	0.885	0.64	7.15E-48	0 L1 _	Hmgn1
Fkbp91	3.18E-52	0.48793	0.732	0.396	6.41E-48	0 L1 _	Fkbp9
Cdc42ep5	3.04E-52	0.482304	0.554	0.249	6.12E-48	0 L1 _	Cdc42ep5
Lima11	2.86E-52	0.523835	0.82	0.466	5.75E-48	0 L1 _	Lima1
Il341	2.43E-52	0.589682	0.697	0.362	4.89E-48	0 L1 _	Il34
Bpgm	1.80E-52	0.828904	0.403	0.143	3.62E-48	0 L1 _	Bpgm
Ar1	1.09E-52	0.579885	0.773	0.438	2.20E-48	0 L1 _	Ar
Tmsb101	6.49E-53	0.433645	0.98	0.885	1.31E-48	0 L1 _	Tmsb10
mt-Cytb	5.95E-53	0.332434	0.987	0.991	1.20E-48	0 L1 _	mt-Cytb
Man1c1	3.03E-53	0.443535	0.437	0.161	6.10E-49	0 L1 _	Man1c1
Hspg22	3.01E-53	0.485016	0.93	0.541	6.06E-49	0 L1 _	Hspg2
Cstb	1.41E-53	0.597694	0.761	0.463	2.85E-49	0 L1 _	Cstb
Rhbdf1	1.24E-53	0.473296	0.536	0.231	2.51E-49	0 L1 _	Rhbdf1
Mmp11	1.21E-53	0.622683	0.519	0.216	2.44E-49	0 L1 _	Mmp11
Col12a1	1.10E-53	0.427737	0.333	0.083	2.21E-49	0 L1 _	Col12a1
Col8a2	8.68E-54	0.403236	0.317	0.074	1.75E-49	0 L1 _	Col8a2
Rasl11b	5.11E-54	0.523711	0.367	0.105	1.03E-49	0 L1 _	Rasl11b
Clmp1	2.52E-54	0.473427	0.486	0.18	5.07E-50	0 L1 _	Clmp
Fap1	2.20E-54	0.462526	0.682	0.332	4.43E-50	0 L1 _	Fap
Oat1	1.33E-54	0.518437	0.84	0.511	2.69E-50	0 L1 _	Oat
Fmo11	8.37E-55	0.49606	0.704	0.348	1.69E-50	0 L1 _	Fmo1
Hspa5	6.99E-55	0.65556	0.956	0.831	1.41E-50	0 L1 _	Hspa5
Cxcl16	5.43E-55	0.38333	0.509	0.197	1.09E-50	0 L1 _	Cxcl16
Clec11a1	5.14E-55	0.601881	0.408	0.133	1.04E-50	0 L1 _	Clec11a
Scd11	4.44E-55	0.403307	0.505	0.192	8.94E-51	0 L1 _	Scd1
Abca91	3.16E-55	0.508778	0.51	0.201	6.35E-51	0 L1 _	Abca9
Egfr2	2.44E-55	0.475827	0.498	0.187	4.92E-51	0 L1 _	Egfr
Erlec1	1.60E-55	0.517357	0.689	0.36	3.21E-51	0 L1 _	Erlec1
Setbp1	1.29E-55	0.541783	0.516	0.211	2.59E-51	0 L1 _	Setbp1
Gria3	1.01E-55	0.44296	0.372	0.107	2.04E-51	0 L1 _	Gria3
Pdia61	9.43E-56	0.580587	0.88	0.641	1.90E-51	0 L1 _	Pdia6

Ssr2	9.41E-56	0.537197	0.811	0.53	1.90E-51	0 L1 _	Ssr2
Fgf71	5.86E-56	0.522212	0.408	0.133	1.18E-51	0 L1 _	Fgf7
Tgfbr22	4.70E-56	0.563055	0.837	0.528	9.47E-52	0 L1 _	Tgfbr2
Ptgis1	3.63E-56	0.53406	0.426	0.141	7.31E-52	0 L1 _	Ptgis
Msc	2.82E-56	0.448791	0.331	0.08	5.69E-52	0 L1 _	Msc
Gm15867	2.00E-56	0.371049	0.313	0.068	4.03E-52	0 L1 _	Gm15867
Prss12	1.36E-56	0.448344	0.329	0.078	2.73E-52	0 L1 _	Prss12
Lrrc32	1.22E-56	0.610782	0.576	0.258	2.45E-52	0 L1 _	Lrrc32
Cyth31	9.69E-57	0.592454	0.864	0.54	1.95E-52	0 L1 _	Cyth3
Rora1	4.44E-57	0.592926	0.731	0.389	8.95E-53	0 L1 _	Rora
Pdzrn3	4.34E-57	0.468839	0.429	0.142	8.74E-53	0 L1 _	Pdzrn3
Tceal8	4.08E-57	0.494587	0.642	0.314	8.22E-53	0 L1 _	Tceal8
Wdr66	2.85E-57	0.571711	0.404	0.128	5.75E-53	0 L1 _	Wdr66
Nenf	2.20E-57	0.539449	0.926	0.671	4.44E-53	0 L1 _	Nenf
Sec31a	1.62E-57	0.550871	0.793	0.476	3.26E-53	0 L1 _	Sec31a
Ckap41	1.33E-57	0.585683	0.727	0.386	2.68E-53	0 L1 _	Ckap4
Cx3cl1	1.29E-57	0.552269	0.582	0.258	2.60E-53	0 L1 _	Cx3cl1
Socs21	8.93E-58	0.529257	0.698	0.36	1.80E-53	0 L1 _	Socs2
Fam114a11	5.53E-58	0.518787	0.668	0.327	1.11E-53	0 L1 _	Fam114a1
Selenos1	5.50E-58	0.609309	0.83	0.53	1.11E-53	0 L1 _	Selenos
Rbms31	4.56E-58	0.439394	0.815	0.408	9.18E-54	0 L1 _	Rbms3
Bhlhe41	4.15E-58	0.560627	0.357	0.093	8.35E-54	0 L1 _	Bhlhe41
F2r	3.08E-58	0.742139	0.551	0.249	6.20E-54	0 L1 _	F2r
Basp12	2.84E-58	0.411069	0.586	0.235	5.72E-54	0 L1 _	Basp1
Col27a11	2.35E-58	0.553833	0.53	0.206	4.72E-54	0 L1 _	Col27a1
Pdlim4	8.46E-59	0.47175	0.44	0.147	1.70E-54	0 L1 _	Pdlim4
Ctsz	5.34E-59	0.489619	0.909	0.658	1.08E-54	0 L1 _	Ctsz
Ccn5	4.21E-59	1.34691	0.257	0.033	8.48E-55	0 L1 _	Ccn5
Crtap	3.98E-59	0.504133	0.699	0.351	8.01E-55	0 L1 _	Crtap
Htra11	9.14E-60	0.604275	0.875	0.503	1.84E-55	0 L1 _	Htra1
Pi161	4.19E-60	0.49353	0.45	0.144	8.43E-56	0 L1 _	Pi16
Fam180a	4.17E-60	0.630666	0.291	0.05	8.40E-56	0 L1 _	Fam180a
Ctsb1	2.74E-60	0.457834	0.933	0.667	5.52E-56	0 L1 _	Ctsb
Lrrk1	2.27E-60	0.599504	0.624	0.297	4.58E-56	0 L1 _	Lrrk1
Plac81	3.69E-61	0.861323	0.567	0.229	7.43E-57	0 L1 _	Plac8
Rap2b	2.64E-61	0.531105	0.563	0.24	5.31E-57	0 L1 _	Rap2b
Stimate	1.61E-61	0.378006	0.377	0.094	3.24E-57	0 L1 _	Stimate
Ank21	1.35E-61	0.58673	0.53	0.199	2.72E-57	0 L1 _	Ank2
Svil1	8.90E-62	0.635492	0.713	0.359	1.79E-57	0 L1 _	Svil
Plau1	7.23E-62	0.569043	0.521	0.194	1.46E-57	0 L1 _	Plau
Tgfb3	7.00E-62	0.532183	0.487	0.168	1.41E-57	0 L1 _	Tgfb3

Ergic3	3.60E-62	0.595992	0.809	0.488	7.25E-58	0 L1 _	Ergic3
Snhg18	1.50E-62	0.553893	0.831	0.484	3.02E-58	0 L1 _	Snhg18
Hdlbp1	1.48E-62	0.63946	0.865	0.588	2.98E-58	0 L1 _	Hdlbp
Col4a5	1.47E-62	0.642708	0.454	0.151	2.97E-58	0 L1 _	Col4a5
Cp1	1.44E-62	0.667959	0.845	0.436	2.89E-58	0 L1 _	Cp
Pros1	1.39E-62	0.569805	0.769	0.423	2.80E-58	0 L1 _	Pros1
Shc4	1.23E-62	0.508597	0.336	0.072	2.48E-58	0 L1 _	Shc4
Copz2	1.07E-62	0.546996	0.668	0.319	2.15E-58	0 L1 _	Copz2
Cnpy2	8.39E-63	0.559167	0.854	0.542	1.69E-58	0 L1 _	Cnpy2
Lrpap1	6.34E-63	0.577199	0.818	0.484	1.28E-58	0 L1 _	Lrpap1
Gas61	6.34E-63	0.637658	0.795	0.417	1.28E-58	0 L1 _	Gas6
Cmklr11	5.93E-63	0.524459	0.496	0.174	1.20E-58	0 L1 _	Cmklr1
Efemp12	3.72E-63	0.4159	0.577	0.207	7.50E-59	0 L1 _	Efemp1
Dnm3os	2.16E-63	0.688621	0.491	0.172	4.35E-59	0 L1 _	Dnm3os
S100a161	1.96E-63	0.660339	0.806	0.455	3.94E-59	0 L1 _	S100a16
Eva1b	1.62E-63	0.601085	0.893	0.602	3.26E-59	0 L1 _	Eva1b
Rhobtb31	1.27E-63	0.539326	0.584	0.239	2.56E-59	0 L1 _	Rhobtb3
Prrx11	9.39E-64	0.634169	0.934	0.572	1.89E-59	0 L1 _	Prrx1
Gsto1	4.83E-64	0.547508	0.463	0.155	9.73E-60	0 L1 _	Gsto1
Creb3l11	2.21E-64	0.551878	0.56	0.215	4.44E-60	0 L1 _	Creb3l1
Gadd45a1	1.68E-64	0.655646	0.494	0.177	3.38E-60	0 L1 _	Gadd45a
Npdc1	1.29E-64	0.604716	0.803	0.45	2.60E-60	0 L1 _	Npdc1
Snap47	2.21E-65	0.566403	0.528	0.2	4.44E-61	0 L1 _	Snap47
Gpx81	1.43E-65	0.460618	0.869	0.505	2.89E-61	0 L1 _	Gpx8
Gfpt22	8.60E-66	0.525974	0.523	0.185	1.73E-61	0 L1 _	Gfpt2
Mgst12	7.75E-66	0.515019	0.742	0.338	1.56E-61	0 L1 _	Mgst1
Gpx7	6.27E-66	0.547088	0.543	0.207	1.26E-61	0 L1 _	Gpx7
Mfap2	5.34E-66	0.654532	0.693	0.332	1.07E-61	0 L1 _	Mfap2
Adamtsl11	3.75E-66	0.639448	0.547	0.209	7.55E-62	0 L1 _	Adamtsl1
Fmo21	3.56E-66	0.863983	0.684	0.318	7.17E-62	0 L1 _	Fmo2
Rab34	3.32E-66	0.532986	0.597	0.246	6.68E-62	0 L1 _	Rab34
Capg	2.58E-66	0.729235	0.768	0.446	5.20E-62	0 L1 _	Capg
Atf51	5.37E-67	0.651218	0.718	0.345	1.08E-62	0 L1 _	Atf5
Ifi2111	3.73E-67	0.577348	0.571	0.216	7.51E-63	0 L1 _	Ifi211
Hacd4	3.46E-67	0.683432	0.546	0.211	6.97E-63	0 L1 _	Hacd4
Gstt11	2.58E-67	0.669783	0.615	0.259	5.21E-63	0 L1 _	Gstt1
Ppib	1.64E-68	0.508968	0.964	0.882	3.30E-64	0 L1 _	Ppib
C32	1.18E-68	0.578528	0.629	0.242	2.38E-64	0 L1 _	C3
Gas11	7.88E-69	0.599676	0.879	0.441	1.59E-64	0 L1 _	Gas1
Slc40a1	3.74E-69	0.585512	0.378	0.083	7.53E-65	0 L1 _	Slc40a1
Adra2c	1.97E-69	0.919473	0.332	0.061	3.96E-65	0 L1 _	Adra2c

Ubtd2	1.30E-69	0.541248	0.446	0.131	2.63E-65	0 L1 _	Ubtd2
Spry11	4.21E-70	0.691256	0.765	0.383	8.48E-66	0 L1 _	Spry1
Ndn1	1.91E-70	0.556773	0.709	0.308	3.85E-66	0 L1 _	Ndn
Gas51	1.65E-70	0.678147	0.926	0.571	3.32E-66	0 L1 _	Gas5
Ctsf	1.48E-70	0.554836	0.548	0.202	2.98E-66	0 L1 _	Ctsf
C1s11	1.29E-70	0.616343	0.955	0.562	2.60E-66	0 L1 _	C1s1
Sulf1	4.44E-71	0.622966	0.558	0.208	8.93E-67	0 L1 _	Sulf1
Dchs2	2.91E-71	0.627288	0.412	0.104	5.86E-67	0 L1 _	Dchs2
Pdlim2	2.31E-71	0.702984	0.783	0.435	4.66E-67	0 L1 _	Pdlim2
Lbp1	1.36E-71	0.58729	0.473	0.146	2.74E-67	0 L1 _	Lbp
Inmt1	1.19E-71	1.050032	0.508	0.165	2.40E-67	0 L1 _	Inmt
Txndc51	9.06E-72	0.625512	0.662	0.309	1.82E-67	0 L1 _	Txndc5
Olfml1	5.37E-72	0.556406	0.436	0.117	1.08E-67	0 L1 _	Olfml1
Bmp12	1.91E-72	0.581753	0.675	0.284	3.86E-68	0 L1 _	Bmp1
Maged11	1.87E-72	0.644648	0.777	0.405	3.76E-68	0 L1 _	Maged1
Plekha61	3.38E-73	0.625277	0.582	0.216	6.80E-69	0 L1 _	Plekha6
Mt22	2.02E-73	0.93679	0.557	0.2	4.07E-69	0 L1 _	Mt2
Vim1	1.80E-73	0.629265	0.967	0.828	3.62E-69	0 L1 _	Vim
F31	1.09E-73	0.645312	0.488	0.146	2.19E-69	0 L1 _	F3
Omd	4.37E-74	0.610391	0.38	0.081	8.80E-70	0 L1 _	Omd
Steap31	4.11E-74	0.552103	0.593	0.207	8.28E-70	0 L1 _	Steap3
Fmod	3.83E-74	0.640824	0.339	0.055	7.72E-70	0 L1 _	Fmod
B3gnt9	2.17E-74	0.558786	0.471	0.143	4.36E-70	0 L1 _	B3gnt9
Adamtsl21	5.82E-75	0.735505	0.511	0.16	1.17E-70	0 L1 _	Adamtsl2
Nav31	4.84E-75	0.664015	0.558	0.2	9.76E-71	0 L1 _	Nav3
1810058I24Rik	4.26E-75	0.741603	0.856	0.563	8.57E-71	0 L1 _	1810058I24Rik
Pdia3	3.82E-75	0.645103	0.961	0.799	7.69E-71	0 L1 _	Pdia3
Auts21	1.24E-75	0.650864	0.725	0.327	2.50E-71	0 L1 _	Auts2
Zfhx41	1.19E-75	0.645501	0.647	0.253	2.40E-71	0 L1 _	Zfhx4
Oaf1	2.75E-76	0.685813	0.75	0.336	5.55E-72	0 L1 _	Oaf
Adcy7	6.12E-77	0.67849	0.662	0.28	1.23E-72	0 L1 _	Adcy7
Ctsh1	4.54E-77	0.706061	0.832	0.394	9.14E-73	0 L1 _	Ctsh
Cyp1b11	3.10E-77	0.68319	0.567	0.191	6.23E-73	0 L1 _	Cyp1b1
Creg1	2.36E-77	0.696508	0.782	0.428	4.75E-73	0 L1 _	Creg1
Mrc21	1.19E-77	0.598944	0.668	0.26	2.40E-73	0 L1 _	Mrc2
Calr1	6.45E-78	0.643308	0.966	0.812	1.30E-73	0 L1 _	Calr
Arap11	1.49E-78	0.674895	0.636	0.267	3.00E-74	0 L1 _	Arap1
Scara52	1.27E-78	0.608516	0.665	0.235	2.55E-74	0 L1 _	Scara5
Lbhd2	6.52E-79	0.771685	0.336	0.047	1.31E-74	0 L1 _	Lbhd2
Scarf21	6.03E-79	0.624274	0.689	0.288	1.21E-74	0 L1 _	Scarf2
Lrrn2	4.80E-79	0.602445	0.473	0.131	9.66E-75	0 L1 _	Lrrn2

Barx1	3.16E-79	0.672303	0.829	0.414	6.37E-75	0 L1 _	Barx1
Fcgrt1	2.20E-79	0.711178	0.914	0.584	4.44E-75	0 L1 _	Fcgrt
Gaa1	4.80E-80	0.624923	0.743	0.349	9.67E-76	0 L1 _	Gaa
Chpf1	3.73E-80	0.67428	0.714	0.315	7.50E-76	0 L1 _	Chpf
Ftl11	1.08E-80	0.538984	0.982	0.95	2.17E-76	0 L1 _	Ftl1
P3h31	5.00E-81	0.664944	0.739	0.34	1.01E-76	0 L1 _	P3h3
Slit2	2.29E-81	0.593326	0.438	0.097	4.61E-77	0 L1 _	Slit2
Lamp21	1.05E-81	0.702922	0.934	0.632	2.11E-77	0 L1 _	Lamp2
Ppic	1.43E-82	0.704719	0.808	0.438	2.89E-78	0 L1 _	Ppic
Kdelr31	8.72E-83	0.644593	0.632	0.238	1.76E-78	0 L1 _	Kdelr3
Col6a61	3.17E-83	0.759847	0.547	0.169	6.38E-79	0 L1 _	Col6a6
Tenm3	1.46E-83	0.647462	0.492	0.134	2.93E-79	0 L1 _	Tenm3
Gstm21	1.41E-83	0.661399	0.738	0.317	2.85E-79	0 L1 _	Gstm2
Fzd2	9.00E-84	0.70641	0.516	0.154	1.81E-79	0 L1 _	Fzd2
Efemp2	3.14E-84	0.677274	0.717	0.314	6.33E-80	0 L1 _	Efemp2
Eid1	1.15E-84	0.77603	0.924	0.691	2.32E-80	0 L1 _	Eid1
Gpr1531	9.56E-85	0.673637	0.623	0.223	1.93E-80	0 L1 _	Gpr153
Nme2	6.05E-85	0.775946	0.952	0.661	1.22E-80	0 L1 _	Nme2
Vgll3	1.31E-85	0.690389	0.54	0.159	2.65E-81	0 L1 _	Vgll3
Tmem1191	8.88E-86	0.643282	0.63	0.218	1.79E-81	0 L1 _	Tmem119
Serf1	6.69E-86	0.717612	0.585	0.208	1.35E-81	0 L1 _	Serf1
Phlda31	1.44E-86	0.82148	0.789	0.397	2.90E-82	0 L1 _	Phlda3
Marcks1	1.23E-86	0.786908	0.975	0.721	2.48E-82	0 L1 _	Marcks
Lamb11	1.07E-86	0.74018	0.918	0.48	2.16E-82	0 L1 _	Lamb1
Snx9	4.06E-87	0.727385	0.741	0.348	8.18E-83	0 L1 _	Snx9
Ccbe1	2.78E-87	0.655706	0.434	0.094	5.59E-83	0 L1 _	Ccbe1
Ltbp2	4.12E-88	1.036489	0.419	0.076	8.29E-84	0 L1 _	Ltbp2
Fhl2	9.57E-89	0.775438	0.629	0.232	1.93E-84	0 L1 _	Fhl2
Adk	1.07E-89	0.689955	0.612	0.218	2.16E-85	0 L1 _	Adk
Vkorc1	4.33E-90	0.75413	0.835	0.451	8.72E-86	0 L1 _	Vkorc1
Prrx21	2.02E-91	0.676642	0.554	0.164	4.07E-87	0 L1 _	Prrx2
Thbs4	1.95E-91	2.246591	0.464	0.102	3.93E-87	0 L1 _	Thbs4
Aldh1a11	2.62E-92	0.901601	0.582	0.188	5.27E-88	0 L1 _	Aldh1a1
Ltbp31	2.53E-93	0.736957	0.687	0.275	5.10E-89	0 L1 _	Ltbp3
Nbl1	1.28E-93	1.148849	0.878	0.498	2.57E-89	0 L1 _	Nbl1
Islr1	6.45E-94	0.717973	0.747	0.285	1.30E-89	0 L1 _	Islr
Col5a12	2.58E-94	0.81985	0.797	0.323	5.21E-90	0 L1 _	Col5a1
Lpar11	1.19E-94	0.683374	0.684	0.234	2.40E-90	0 L1 _	Lpar1
Col16a1	9.16E-95	0.744286	0.636	0.225	1.84E-90	0 L1 _	Col16a1
Tspan111	1.08E-95	0.718336	0.647	0.213	2.18E-91	0 L1 _	Tspan11
Ptgfrn1	3.72E-96	0.69828	0.658	0.234	7.49E-92	0 L1 _	Ptgfrn

Igsf101	3.34E-96	0.793971	0.761	0.296	6.73E-92	0 L1 _	Igsf10
Ank	2.57E-96	0.801368	0.574	0.184	5.18E-92	0 L1 _	Ank
Mt11	1.77E-96	1.125912	0.868	0.459	3.57E-92	0 L1 _	Mt1
Nav11	1.21E-96	0.864728	0.891	0.435	2.44E-92	0 L1 _	Nav1
Hexa	4.26E-97	0.790928	0.84	0.451	8.59E-93	0 L1 _	Hexa
Clec3b2	2.11E-97	0.663031	0.727	0.239	4.25E-93	0 L1 _	Clec3b
Sdc21	2.07E-97	0.809182	0.827	0.382	4.17E-93	0 L1 _	Sdc2
Col14a12	7.38E-99	0.709951	0.752	0.26	1.49E-94	0 L1 _	Col14a1
Fgfr11	5.35E-99	0.727153	0.764	0.304	1.08E-94	0 L1 _	Fgfr1
Cpxm2	2.68E-99	1.35466	0.541	0.147	5.40E-95	0 L1 _	Cpxm2
Flrt21	1.71E-99	0.914739	0.626	0.213	3.45E-95	0 L1 _	Flrt2
Lsamp1	1.05E-99	0.761365	0.793	0.306	2.12E-95	0 L1 _	Lsamp
Pla1a	8.93E-100	0.871566	0.684	0.236	1.80E-95	0 L1 _	Pla1a
Thbs2	8.14E-101	0.795729	0.63	0.206	1.64E-96	0 L1 _	Thbs2
Selenop1	5.24E-101	0.918764	0.961	0.632	1.06E-96	0 L1 _	Selenop
Tent5a	4.18E-101	0.88937	0.672	0.229	8.42E-97	0 L1 _	Tent5a
Ckb	1.63E-101	1.114994	0.852	0.49	3.28E-97	0 L1 _	Ckb
Tuba1a1	4.33E-102	0.870055	0.964	0.738	8.71E-98	0 L1 _	Tuba1a
Mmp141	1.43E-102	0.829303	0.824	0.348	2.89E-98	0 L1 _	Mmp14
Vcam1	1.42E-102	0.834692	0.573	0.159	2.86E-98	0 L1 _	Vcam1
Ifitm2	2.78E-103	0.756229	0.976	0.833	5.60E-99	0 L1 _	Ifitm2
Pcdh71	1.87E-103	0.808696	0.633	0.2	3.76E-99	0 L1 _	Pcdh7
Nid12	1.10E-103	0.768597	0.957	0.583	2.21E-99	0 L1 _	Nid1
Fbn12	3.40E-104	0.786002	0.888	0.441	6.84E-100	0 L1 _	Fbn1
Ahnak2	1.42E-104	0.824469	0.611	0.177	2.85E-100	0 L1 _	Ahnak2
Tmed31	1.30E-104	0.867406	0.88	0.531	2.61E-100	0 L1 _	Tmed3
Ms4a4d1	9.59E-105	0.809893	0.736	0.253	1.93E-100	0 L1 _	Ms4a4d
Fbn2	8.02E-105	0.885648	0.51	0.11	1.62E-100	0 L1 _	Fbn2
Emilin1	4.28E-105	0.831476	0.76	0.311	8.62E-101	0 L1 _	Emilin1
Prss23	2.38E-105	1.054602	0.933	0.578	4.80E-101	0 L1 _	Prss23
Mmp31	7.62E-106	1.755535	0.609	0.176	1.53E-101	0 L1 _	Mmp3
Fkbp71	6.04E-106	0.765686	0.842	0.381	1.22E-101	0 L1 _	Fkbp7
Col5a31	5.28E-106	0.796803	0.859	0.365	1.06E-101	0 L1 _	Col5a3
Wls	6.96E-107	0.889509	0.884	0.5	1.40E-102	0 L1 _	Wls
Sfrp1	4.80E-108	1.041802	0.617	0.175	9.66E-104	0 L1 _	Sfrp1
Nfix1	1.57E-108	0.84602	0.947	0.513	3.16E-104	0 L1 _	Nfix
Lhfp	5.97E-109	0.85442	0.926	0.508	1.20E-104	0 L1 _	Lhfp
Cd91	2.75E-109	0.879345	0.937	0.58	5.54E-105	0 L1 _	Cd9
Rrbp11	1.01E-111	0.828402	0.974	0.823	2.04E-107	0 L1 _	Rrbp1
Pdpn1	4.58E-114	0.834582	0.652	0.195	9.22E-110	0 L1 _	Pdpn
Nkd2	3.98E-114	0.930401	0.589	0.143	8.01E-110	0 L1 _	Nkd2

Scpep11	2.71E-114	0.839711	0.738	0.278	5.45E-110	0 L1 _	Scpep1
Cped11	1.59E-115	1.004832	0.946	0.53	3.21E-111	0 L1 _	Cped1
Srpx21	5.65E-117	0.889295	0.64	0.189	1.14E-112	0 L1 _	Srpx2
Enpp1	1.33E-117	0.989238	0.646	0.193	2.68E-113	0 L1 _	Enpp1
Srpx1	1.37E-118	0.671195	0.792	0.256	2.76E-114	0 L1 _	Srpx
Igf11	2.44E-119	0.937027	0.82	0.297	4.91E-115	0 L1 _	Igf1
Abca8a1	1.64E-119	0.898219	0.85	0.313	3.30E-115	0 L1 _	Abca8a
Fth11	3.93E-120	0.832326	0.994	0.992	7.92E-116	0 L1 _	Fth1
Tgfbi1	6.01E-121	1.100849	0.729	0.237	1.21E-116	0 L1 _	Tgfbi
Pla2g4a	1.26E-121	0.862246	0.655	0.19	2.53E-117	0 L1 _	Pla2g4a
Ccn4	3.39E-122	0.930507	0.518	0.093	6.83E-118	0 L1 _	Ccn4
C1ra1	3.79E-125	0.96479	0.86	0.342	7.64E-121	0 L1 _	C1ra
Il331	9.78E-126	0.9606	0.757	0.229	1.97E-121	0 L1 _	Il33
Plat	3.39E-126	0.917989	0.823	0.324	6.83E-122	0 L1 _	Plat
Cpq1	3.38E-126	0.878146	0.75	0.264	6.80E-122	0 L1 _	Cpq
Col15a11	2.17E-126	1.085166	0.938	0.428	4.38E-122	0 L1 _	Col15a1
Dpep11	1.43E-127	0.948814	0.849	0.271	2.87E-123	0 L1 _	Dpep1
Mir100hg	1.32E-127	0.890357	0.72	0.2	2.66E-123	0 L1 _	Mir100hg
Fzd1	8.16E-129	1.03609	0.691	0.216	1.64E-124	0 L1 _	Fzd1
Sparc1	4.19E-129	1.081525	0.991	0.877	8.44E-125	0 L1 _	Sparc
Crispld1	1.87E-130	1.161827	0.768	0.272	3.77E-126	0 L1 _	Crispld1
Mafb1	1.51E-130	1.164671	0.883	0.389	3.03E-126	0 L1 _	Mafb
Timp22	4.37E-131	0.79585	0.962	0.531	8.80E-127	0 L1 _	Timp2
Ifi30	1.24E-131	0.966826	0.699	0.196	2.50E-127	0 L1 _	Ifi30
Kcnk2	1.08E-131	0.970619	0.653	0.167	2.18E-127	0 L1 _	Kcnk2
Spats2l1	5.82E-132	1.026578	0.785	0.294	1.17E-127	0 L1 _	Spats2l
Ccn2	5.33E-132	1.515107	0.655	0.159	1.07E-127	0 L1 _	Ccn2
Cygb	3.85E-132	1.048926	0.979	0.614	7.75E-128	0 L1 _	Cygb
Mtch1	1.38E-132	1.086124	0.923	0.63	2.78E-128	0 L1 _	Mtch1
Rnase42	2.80E-134	0.904407	0.938	0.373	5.63E-130	0 L1 _	Rnase4
Cd3021	1.89E-135	1.046942	0.925	0.417	3.80E-131	0 L1 _	Cd302
Mmp21	6.59E-136	0.898987	0.926	0.314	1.33E-131	0 L1 _	Mmp2
Lgals1	9.69E-138	1.217545	0.974	0.708	1.95E-133	0 L1 _	Lgals1
Itih51	7.06E-138	1.12874	0.95	0.492	1.42E-133	0 L1 _	Itih5
S100a61	4.73E-138	1.062297	0.989	0.887	9.53E-134	0 L1 _	S100a6
Crlf11	1.42E-138	1.236727	0.725	0.213	2.87E-134	0 L1 _	Crlf1
C1qtnf2	3.61E-140	1.02657	0.869	0.371	7.27E-136	0 L1 _	C1qtnf2
Serpinh11	3.35E-140	1.026806	0.966	0.706	6.75E-136	0 L1 _	Serpinh1
C2	2.04E-140	1.086392	0.693	0.191	4.12E-136	0 L1 _	C2
Scn7a1	2.16E-143	0.989791	0.844	0.296	4.36E-139	0 L1 _	Scn7a
Eln	3.18E-145	1.42667	0.891	0.428	6.41E-141	0 L1 _	Eln

Itgb5	2.14E-146	1.180656	0.904	0.489	4.31E-142	0 L1 _	Itgb5
Hsd11b11	1.33E-149	1.647699	0.821	0.314	2.67E-145	0 L1 _	Hsd11b1
Gpm6b1	1.83E-150	1.124157	0.875	0.285	3.68E-146	0 L1 _	Gpm6b
Cfb	1.76E-151	1.355532	0.655	0.146	3.54E-147	0 L1 _	Cfb
Timp1	5.07E-153	2.053152	0.71	0.183	1.02E-148	0 L1 _	Timp1
Lama21	7.58E-154	1.174344	0.948	0.406	1.53E-149	0 L1 _	Lama2
Mir99ahg	2.10E-154	1.033698	0.686	0.149	4.24E-150	0 L1 _	Mir99ahg
Col8a1	4.13E-155	1.783269	0.689	0.169	8.31E-151	0 L1 _	Col8a1
Pdgfrl	2.19E-156	1.220712	0.66	0.13	4.41E-152	0 L1 _	Pdgfrl
Mxra7	1.75E-156	1.128999	0.926	0.503	3.53E-152	0 L1 _	Mxra7
Epha3	1.57E-156	1.156966	0.712	0.182	3.16E-152	0 L1 _	Epha3
Adamts52	1.39E-157	1.389822	0.875	0.288	2.80E-153	0 L1 _	Adamts5
Fstl11	7.46E-158	1.175573	0.96	0.522	1.50E-153	0 L1 _	Fstl1
Fibin	3.08E-158	1.295021	0.751	0.212	6.21E-154	0 L1 _	Fibin
Crispld21	7.74E-159	1.24837	0.932	0.352	1.56E-154	0 L1 _	Crispld2
Lrp11	9.90E-162	1.20577	0.973	0.512	1.99E-157	0 L1 _	Lrp1
Itm2a1	1.27E-162	1.305694	0.935	0.426	2.56E-158	0 L1 _	Itm2a
Cd63	1.01E-163	1.106349	0.981	0.691	2.02E-159	0 L1 _	Cd63
Gsn1	3.11E-165	1.084509	0.989	0.675	6.26E-161	0 L1 _	Gsn
Pmepa1	5.99E-169	1.477315	0.95	0.566	1.21E-164	0 L1 _	Pmepa1
Dpysl31	5.88E-169	1.201258	0.865	0.283	1.18E-164	0 L1 _	Dpysl3
Mxra81	4.41E-169	1.233364	0.939	0.41	8.89E-165	0 L1 _	Mxra8
Htra31	3.76E-169	1.34169	0.924	0.324	7.58E-165	0 L1 _	Htra3
Ogn1	9.40E-170	1.551174	0.891	0.312	1.89E-165	0 L1 _	Ogn
Col6a21	1.92E-170	1.318897	0.958	0.468	3.87E-166	0 L1 _	Col6a2
Pdgfra1	1.95E-171	1.370209	0.978	0.469	3.93E-167	0 L1 _	Pdgfra
Igfbp41	3.77E-172	1.348912	0.975	0.57	7.60E-168	0 L1 _	Igfbp4
Il11ra11	1.27E-172	1.30427	0.932	0.396	2.56E-168	0 L1 _	Il11ra1
Igfbp61	3.61E-173	1.35674	0.848	0.244	7.28E-169	0 L1 _	Igfbp6
Rcn31	5.34E-177	1.225402	0.932	0.43	1.08E-172	0 L1 _	Rcn3
Col6a11	9.28E-179	1.331116	0.973	0.537	1.87E-174	0 L1 _	Col6a1
Serping11	2.76E-179	1.366648	0.982	0.604	5.56E-175	0 L1 _	Serping1
Spon11	2.21E-179	1.378563	0.849	0.254	4.45E-175	0 L1 _	Spon1
Podn1	1.88E-179	1.505721	0.818	0.23	3.78E-175	0 L1 _	Podn
Pcolce1	1.61E-180	1.325548	0.973	0.604	3.24E-176	0 L1 _	Pcolce
Col6a31	1.11E-181	1.538754	0.95	0.355	2.23E-177	0 L1 _	Col6a3
Col5a21	8.02E-183	1.466613	0.96	0.442	1.62E-178	0 L1 _	Col5a2
Plxdc21	8.85E-185	1.373988	0.953	0.337	1.78E-180	0 L1 _	Plxdc2
Entpd21	4.70E-185	1.308035	0.91	0.298	9.47E-181	0 L1 _	Entpd2
Ccdc801	6.06E-186	1.440915	0.941	0.401	1.22E-181	0 L1 _	Ccdc80
Prelp1	1.10E-186	1.505862	0.882	0.292	2.22E-182	0 L1 _	Prelp

Mfap51	2.60E-189	1.746988	0.888	0.298	5.23E-185	0 L1 _	Mfap5
Dcn1	9.02E-191	1.619709	0.992	0.461	1.82E-186	0 L1 _	Dcn
Ctsl1	1.94E-191	1.32455	0.975	0.701	3.91E-187	0 L1 _	Ctsl
Abi3bp	1.12E-191	1.591996	0.876	0.276	2.25E-187	0 L1 _	Abi3bp
Gask1b	2.08E-192	1.371254	0.785	0.177	4.18E-188	0 L1 _	Gask1b
Lox	1.53E-194	1.587288	0.797	0.195	3.09E-190	0 L1 _	Lox
Col3a11	4.69E-198	1.710564	0.987	0.601	9.45E-194	0 L1 _	Col3a1
Mdk	1.15E-205	1.524995	0.907	0.309	2.32E-201	0 L1 _	Mdk
Lum1	9.84E-208	1.641907	0.98	0.355	1.98E-203	0 L1 _	Lum
Col1a21	1.20E-208	1.736493	0.984	0.592	2.42E-204	0 L1 _	Col1a2
Colec121	4.09E-210	1.501374	0.94	0.322	8.24E-206	0 L1 _	Colec12
Emp11	1.92E-211	1.604539	0.961	0.453	3.86E-207	0 L1 _	Emp1
Smoc21	8.02E-212	1.725647	0.908	0.264	1.61E-207	0 L1 _	Smoc2
Mfap4	1.14E-212	1.923695	0.802	0.163	2.29E-208	0 L1 _	Mfap4
Fbln71	4.30E-213	1.448524	0.88	0.243	8.66E-209	0 L1 _	Fbln7
Spp1	7.09E-214	2.938211	0.842	0.224	1.43E-209	0 L1 _	Spp1
Ctsk	4.18E-216	1.548095	0.92	0.323	8.41E-212	0 L1 _	Ctsk
Fxyd6	2.01E-217	1.81976	0.854	0.224	4.05E-213	0 L1 _	Fxyd6
Bicc11	9.34E-218	1.576652	0.956	0.354	1.88E-213	0 L1 _	Bicc1
Col1a11	2.10E-218	1.853802	0.98	0.465	4.23E-214	0 L1 _	Col1a1
Lpl1	2.05E-218	1.848937	0.979	0.484	4.12E-214	0 L1 _	Lpl
Cxcl141	6.98E-220	2.089792	0.965	0.379	1.41E-215	0 L1 _	Cxcl14
Fbln11	6.20E-220	1.589965	0.923	0.27	1.25E-215	0 L1 _	Fbln1
Olfml3	4.80E-220	1.609825	0.898	0.286	9.66E-216	0 L1 _	Olfml3
Svep1	2.02E-224	1.573931	0.891	0.246	4.08E-220	0 L1 _	Svep1
Cpxm11	3.57E-225	1.671395	0.924	0.271	7.18E-221	0 L1 _	Cpxm1
Cilp	3.66E-230	2.479287	0.878	0.218	7.38E-226	0 L1 _	Cilp
Nupr11	7.05E-232	1.928896	0.955	0.373	1.42E-227	0 L1 _	Nupr1
Apod1	3.32E-238	2.350431	0.986	0.421	6.68E-234	0 L1 _	Apod
Cfh	2.01E-238	1.853023	0.969	0.399	4.06E-234	0 L1 _	Cfh
Cdh111	5.00E-242	1.801704	0.955	0.27	1.01E-237	0 L1 _	Cdh11
Aebp1	1.19E-243	1.782552	0.972	0.399	2.40E-239	0 L1 _	Aebp1
Itgbl1	5.21E-245	1.808423	0.893	0.215	1.05E-240	0 L1 _	Itgbl1
Loxl1	3.63E-247	1.787859	0.962	0.306	7.32E-243	0 L1 _	Loxl1
Rbp1	2.32E-265	2.130634	0.96	0.396	4.67E-261	0 L1 _	Rbp1
Serpinf11	2.84E-266	2.052953	0.98	0.336	5.72E-262	0 L1 _	Serpinf1
Bgn	1.40E-279	2.173885	0.988	0.645	2.82E-275	0 L1 _	Bgn
Mgp	8.11E-292	2.765209	0.993	0.736	1.63E-287	0 L1 _	Mgp
Ccdc88a	1.86E-06	0.282521	0.599	0.412	0.037451	0 M1 _	Ccdc88a
Xbp1	1.83E-06	0.267449	0.784	0.591	0.036758	0 M1 _	Xbp1
Snx6	1.75E-06	0.319531	0.737	0.539	0.035201	0 M1 _	Snx6

Sfrp2	1.53E-06	0.557054	0.204	0.088	0.030768	0 M1 _	Sfrp2
C4b	1.38E-06	0.473834	0.192	0.081	0.027788	0 M1 _	C4b
Lamc3	1.26E-06	0.311435	0.485	0.302	0.025298	0 M1 _	Lamc3
Ncbp2	1.17E-06	0.33131	0.587	0.382	0.023591	0 M1 _	Ncbp2
Irgm2	1.16E-06	0.260315	0.365	0.203	0.023379	0 M1 _	Irgm2
Ptgis	1.03E-06	0.321014	0.413	0.244	0.02081	0 M1 _	Ptgis
Sec63	9.58E-07	0.276401	0.772	0.55	0.019287	0 M1 _	Sec63
Slc25a39	9.01E-07	0.266498	0.563	0.369	0.018143	0 M1 _	Slc25a39
Pdap1	8.65E-07	0.264643	0.904	0.711	0.017428	0 M1 _	Pdap1
Nucb2	8.01E-07	0.254867	0.593	0.396	0.016127	0 M1 _	Nucb2
Matr3	7.96E-07	0.250398	0.749	0.517	0.016031	0 M1 _	Matr3
Spon1	7.23E-07	0.465567	0.683	0.481	0.014553	0 M1 _	Spon1
Errfi1	6.59E-07	0.37925	0.814	0.615	0.013269	0 M1 _	Errfi1
Lmna	3.86E-07	0.365582	0.91	0.68	0.00777	0 M1 _	Lmna
Hist1h2bc	3.80E-07	0.353975	0.701	0.478	0.007657	0 M1 _	Hist1h2bc
Rex1bd	3.32E-07	0.289285	0.808	0.58	0.006692	0 M1 _	Rex1bd
Ace	3.13E-07	0.312419	0.593	0.384	0.006314	0 M1 _	Ace
Cpxm1	2.80E-07	0.291627	0.802	0.516	0.005647	0 M1 _	Cpxm1
Lhfpl2	2.77E-07	0.251662	0.431	0.247	0.005589	0 M1 _	Lhfpl2
Ikbip	1.91E-07	0.345953	0.671	0.463	0.003854	0 M1 _	Ikbip
Tuba1b	1.85E-07	0.312288	0.952	0.813	0.003736	0 M1 _	Tuba1b
Fam120a	1.75E-07	0.256668	0.641	0.429	0.00353	0 M1 _	Fam120a
Rpl4	1.73E-07	0.314828	0.976	0.83	0.003492	0 M1 _	Rpl4
Nupr1	1.67E-07	0.314685	0.964	0.582	0.003369	0 M1 _	Nupr1
Lbp	1.59E-07	0.26101	0.461	0.265	0.003195	0 M1 _	Lbp
Golim4	1.54E-07	0.349714	0.79	0.563	0.003102	0 M1 _	Golim4
Capn1	1.42E-07	0.261089	0.443	0.261	0.002853	0 M1 _	Capn1
Sec62	1.38E-07	0.295436	0.964	0.819	0.002784	0 M1 _	Sec62
Pofut2	1.33E-07	0.30597	0.671	0.444	0.00267	0 M1 _	Pofut2
Pds5b	1.32E-07	0.279246	0.587	0.367	0.002653	0 M1 _	Pds5b
Kdelr3	1.31E-07	0.253068	0.629	0.38	0.002633	0 M1 _	Kdelr3
Hdlbp	1.27E-07	0.294505	0.934	0.682	0.002564	0 M1 _	Hdlbp
Sspn	1.25E-07	0.262326	0.551	0.34	0.002522	0 M1 _	Sspn
Tm2d2	1.24E-07	0.276199	0.713	0.476	0.002494	0 M1 _	Tm2d2
Tspan31	1.22E-07	0.264161	0.659	0.445	0.00246	0 M1 _	Tspan31
Ogn	1.16E-07	0.433338	0.772	0.53	0.002331	0 M1 _	Ogn
Anxa2	1.04E-07	0.288292	0.976	0.839	0.002085	0 M1 _	Anxa2
Dhrs3	9.84E-08	0.266609	0.635	0.405	0.001983	0 M1 _	Dhrs3
Tm9sf3	9.84E-08	0.281227	0.922	0.738	0.001982	0 M1 _	Tm9sf3
Rcn3	9.37E-08	0.273383	0.928	0.611	0.001886	0 M1 _	Rcn3
Yipf5	9.36E-08	0.263762	0.707	0.477	0.001884	0 M1 _	Yipf5

Eif4g1	9.10E-08	0.282231	0.784	0.574	0.001834	0 M1 _	Eif4g1
Ier3ip1	8.80E-08	0.263036	0.88	0.644	0.001772	0 M1 _	Ier3ip1
Uba1	7.15E-08	0.264951	0.653	0.438	0.001439	0 M1 _	Uba1
Mgat2	6.55E-08	0.298038	0.671	0.43	0.001319	0 M1 _	Mgat2
Sec61g	6.49E-08	0.330024	0.97	0.852	0.001308	0 M1 _	Sec61g
Shox2	5.86E-08	0.272303	0.437	0.241	0.00118	0 M1 _	Shox2
Socs4	4.79E-08	0.26334	0.407	0.209	0.000964	0 M1 _	Socs4
Ccdc80	4.41E-08	0.305476	0.904	0.598	0.000889	0 M1 _	Ccdc80
Wipf1	4.39E-08	0.283841	0.647	0.438	0.000884	0 M1 _	Wipf1
Dync1h1	3.98E-08	0.29105	0.832	0.618	0.000801	0 M1 _	Dync1h1
Rab6a	3.66E-08	0.260849	0.737	0.504	0.000738	0 M1 _	Rab6a
Fxyd1	3.52E-08	0.258569	0.946	0.658	0.000709	0 M1 _	Fxyd1
Nrxn1	3.43E-08	0.46357	0.257	0.113	0.000692	0 M1 _	Nrxn1
Vcp	3.22E-08	0.320936	0.94	0.752	0.000649	0 M1 _	Vcp
Anxa5	3.18E-08	0.289226	0.994	0.879	0.00064	0 M1 _	Anxa5
Dusp1	3.04E-08	0.281266	0.557	0.322	0.000611	0 M1 _	Dusp1
Twf1	2.93E-08	0.291049	0.617	0.401	0.00059	0 M1 _	Twf1
Gabarapl1	2.62E-08	0.269372	0.707	0.469	0.000527	0 M1 _	Gabarapl1
Klf6	2.55E-08	0.339043	0.778	0.543	0.000514	0 M1 _	Klf6
Chpt1	2.40E-08	0.298438	0.587	0.36	0.000484	0 M1 _	Chpt1
Cebpb	1.92E-08	0.311682	0.647	0.414	0.000387	0 M1 _	Cebpb
Svil	1.84E-08	0.413352	0.701	0.488	0.000371	0 M1 _	Svil
Cdh11	1.81E-08	0.317812	0.85	0.525	0.000366	0 M1 _	Cdh11
Tor1aip1	1.72E-08	0.387919	0.79	0.576	0.000347	0 M1 _	Tor1aip1
Lman1	1.58E-08	0.321131	0.844	0.612	0.000319	0 M1 _	Lman1
Copa	1.55E-08	0.27564	0.838	0.602	0.000313	0 M1 _	Copa
Dram1	1.43E-08	0.250177	0.335	0.165	0.000289	0 M1 _	Dram1
Sp3	1.43E-08	0.275016	0.671	0.436	0.000287	0 M1 _	Sp3
Aldh1a2	1.42E-08	0.274226	0.162	0.053	0.000286	0 M1 _	Aldh1a2
Mettl1	1.27E-08	0.252904	0.491	0.276	0.000256	0 M1 _	Mettl1
Phactr2	1.20E-08	0.257365	0.665	0.42	0.000242	0 M1 _	Phactr2
Fn1	1.16E-08	0.443684	0.317	0.154	0.000234	0 M1 _	Fn1
Amotl1	1.11E-08	0.30247	0.856	0.622	0.000224	0 M1 _	Amotl1
Adamts4	1.09E-08	0.25811	0.329	0.158	0.000219	0 M1 _	Adamts4
Phtf1	9.40E-09	0.303957	0.341	0.161	0.000189	0 M1 _	Phtf1
Rab1a	9.28E-09	0.342343	0.802	0.558	0.000187	0 M1 _	Rab1a
Cdkn1c	7.74E-09	0.582819	0.605	0.397	0.000156	0 M1 _	Cdkn1c
Derl1	6.87E-09	0.252808	0.671	0.428	0.000138	0 M1 _	Derl1
Layn	6.60E-09	0.305305	0.449	0.245	0.000133	0 M1 _	Layn
Lrrc8a	6.32E-09	0.289384	0.587	0.353	0.000127	0 M1 _	Lrrc8a
Sntb2	6.21E-09	0.325288	0.802	0.533	0.000125	0 M1 _	Sntb2

Nucb1	5.45E-09	0.367833	0.826	0.596	0.00011	0 M1 _	Nucb1
Tmed3	5.36E-09	0.328585	0.922	0.654	0.000108	0 M1 _	Tmed3
Sqle	4.81E-09	0.274008	0.329	0.155	9.69E-05	0 M1 _	Sqle
5730455P16Rik	4.72E-09	0.259898	0.359	0.175	9.51E-05	0 M1 _	5730455P16Rik
Mia3	4.52E-09	0.311111	0.743	0.525	9.10E-05	0 M1 _	Mia3
Scarf2	4.39E-09	0.324912	0.689	0.433	8.85E-05	0 M1 _	Scarf2
Scn1b	4.38E-09	0.265022	0.79	0.518	8.82E-05	0 M1 _	Scn1b
Egr1	4.37E-09	0.549175	0.563	0.349	8.81E-05	0 M1 _	Egr1
Arhgef40	4.15E-09	0.259064	0.713	0.434	8.35E-05	0 M1 _	Arhgef40
Phldb1	3.95E-09	0.270957	0.557	0.328	7.96E-05	0 M1 _	Phldb1
Trip12	3.80E-09	0.286211	0.719	0.473	7.66E-05	0 M1 _	Trip12
Atp6v0d1	3.78E-09	0.254554	0.665	0.408	7.60E-05	0 M1 _	Atp6v0d1
Crlf1	3.45E-09	0.397817	0.671	0.402	6.95E-05	0 M1 _	Crlf1
Mcfd2	3.41E-09	0.291884	0.485	0.276	6.86E-05	0 M1 _	Mcfd2
Tmem159	3.26E-09	0.280782	0.545	0.316	6.57E-05	0 M1 _	Tmem159
Grsf1	3.19E-09	0.257205	0.371	0.18	6.42E-05	0 M1 _	Grsf1
Gaa	3.18E-09	0.326776	0.766	0.489	6.41E-05	0 M1 _	Gaa
Furin	3.17E-09	0.267647	0.539	0.314	6.38E-05	0 M1 _	Furin
Nudcd2	3.11E-09	0.28652	0.461	0.256	6.25E-05	0 M1 _	Nudcd2
Cand1	2.89E-09	0.276805	0.419	0.223	5.82E-05	0 M1 _	Cand1
Emp1	2.88E-09	0.287438	0.97	0.636	5.80E-05	0 M1 _	Emp1
Cblb	2.84E-09	0.314972	0.497	0.283	5.72E-05	0 M1 _	Cblb
Herpud1	2.72E-09	0.310871	0.778	0.533	5.48E-05	0 M1 _	Herpud1
Ssbp3	2.54E-09	0.299615	0.575	0.343	5.12E-05	0 M1 _	Ssbp3
Golga2	2.39E-09	0.280112	0.539	0.304	4.81E-05	0 M1 _	Golga2
Lnpep	2.15E-09	0.300458	0.737	0.476	4.33E-05	0 M1 _	Lnpep
mt-Nd3	2.04E-09	0.348547	0.988	0.915	4.10E-05	0 M1 _	mt-Nd3
Caprin1	2.04E-09	0.293698	0.784	0.53	4.10E-05	0 M1 _	Caprin1
Sdcbp	2.01E-09	0.361468	0.838	0.603	4.04E-05	0 M1 _	Sdcbp
Tmed2	1.99E-09	0.342122	0.94	0.727	4.01E-05	0 M1 _	Tmed2
App	1.97E-09	0.360029	0.994	0.828	3.96E-05	0 M1 _	App
Rarres2	1.95E-09	0.334096	0.916	0.674	3.92E-05	0 M1 _	Rarres2
Tcf7l2	1.82E-09	0.300391	0.719	0.448	3.66E-05	0 M1 _	Tcf7l2
Copg1	1.81E-09	0.251813	0.623	0.373	3.65E-05	0 M1 _	Copg1
Wdr26	1.71E-09	0.270393	0.719	0.45	3.45E-05	0 M1 _	Wdr26
Esyt2	1.62E-09	0.285146	0.772	0.523	3.27E-05	0 M1 _	Esyt2
Aida	1.55E-09	0.347042	0.641	0.389	3.12E-05	0 M1 _	Aida
Clec11a	1.33E-09	0.266834	0.455	0.229	2.68E-05	0 M1 _	Clec11a
Ttc28	1.28E-09	0.32566	0.82	0.553	2.57E-05	0 M1 _	Ttc28
S100a16	1.09E-09	0.315308	0.904	0.574	2.20E-05	0 M1 _	S100a16
Tmed7	1.08E-09	0.312843	0.569	0.343	2.17E-05	0 M1 _	Tmed7

Bace1	9.24E-10	0.318497	0.593	0.354	1.86E-05	0 M1 _	Bace1
Mtmr6	9.23E-10	0.255548	0.461	0.245	1.86E-05	0 M1 _	Mtmr6
Rnf144a	8.84E-10	0.343289	0.551	0.323	1.78E-05	0 M1 _	Rnf144a
Selenos	8.70E-10	0.326486	0.91	0.632	1.75E-05	0 M1 _	Selenos
Csde1	8.26E-10	0.333544	0.91	0.686	1.66E-05	0 M1 _	Csde1
Osbpl1a	8.01E-10	0.270233	0.437	0.225	1.61E-05	0 M1 _	Osbpl1a
Srpx2	7.88E-10	0.314041	0.605	0.354	1.59E-05	0 M1 _	Srpx2
Scpep1	7.33E-10	0.304406	0.731	0.444	1.48E-05	0 M1 _	Scpep1
Pbx1	6.25E-10	0.351571	0.743	0.509	1.26E-05	0 M1 _	Pbx1
P4ha1	6.16E-10	0.318887	0.76	0.469	1.24E-05	0 M1 _	P4ha1
Nckap1	6.09E-10	0.28308	0.76	0.5	1.23E-05	0 M1 _	Nckap1
Spry2	6.00E-10	0.258477	0.431	0.218	1.21E-05	0 M1 _	Spry2
Kdelr1	5.79E-10	0.348862	0.868	0.624	1.17E-05	0 M1 _	Kdelr1
Rtn4	5.45E-10	0.391999	0.964	0.728	1.10E-05	0 M1 _	Rtn4
Vps35	5.40E-10	0.3031	0.707	0.441	1.09E-05	0 M1 _	Vps35
Prrc2a	5.32E-10	0.279182	0.641	0.391	1.07E-05	0 M1 _	Prrc2a
Trf	5.22E-10	0.84732	0.443	0.253	1.05E-05	0 M1 _	Trf
Pea15a	5.17E-10	0.321935	0.826	0.549	1.04E-05	0 M1 _	Pea15a
Grina	4.95E-10	0.37259	0.808	0.55	9.97E-06	0 M1 _	Grina
Thbs3	4.74E-10	0.280516	0.323	0.145	9.55E-06	0 M1 _	Thbs3
Smarca2	4.58E-10	0.38136	0.838	0.618	9.22E-06	0 M1 _	Smarca2
Ppp3r1	4.57E-10	0.258975	0.509	0.274	9.20E-06	0 M1 _	Ppp3r1
Ncor2	4.55E-10	0.291778	0.665	0.388	9.15E-06	0 M1 _	Ncor2
Cpd	4.51E-10	0.313278	0.725	0.466	9.08E-06	0 M1 _	Cpd
Naalad2	4.32E-10	0.29957	0.737	0.459	8.70E-06	0 M1 _	Naalad2
Ppp3cb	3.74E-10	0.292812	0.677	0.403	7.53E-06	0 M1 _	Ppp3cb
St13	3.65E-10	0.345527	0.928	0.73	7.35E-06	0 M1 _	St13
Slc25a24	3.33E-10	0.29444	0.551	0.307	6.71E-06	0 M1 _	Slc25a24
Scoc	3.27E-10	0.257236	0.605	0.331	6.58E-06	0 M1 _	Scoc
Camkk2	3.22E-10	0.265997	0.491	0.255	6.49E-06	0 M1 _	Camkk2
Pik3r1	3.11E-10	0.341714	0.85	0.652	6.26E-06	0 M1 _	Pik3r1
Nmb	3.08E-10	0.298247	0.24	0.089	6.19E-06	0 M1 _	Nmb
Fcgrt	2.93E-10	0.35477	0.952	0.7	5.90E-06	0 M1 _	Fcgrt
Tmem100	2.76E-10	0.276869	0.515	0.269	5.56E-06	0 M1 _	Tmem100
Amfr	2.75E-10	0.272772	0.509	0.283	5.55E-06	0 M1 _	Amfr
P3h3	2.71E-10	0.320907	0.796	0.479	5.45E-06	0 M1 _	P3h3
Xdh	2.69E-10	0.33637	0.683	0.414	5.42E-06	0 M1 _	Xdh
Srebf1	2.63E-10	0.277699	0.461	0.243	5.29E-06	0 M1 _	Srebf1
Rnd3	2.54E-10	0.300583	0.641	0.361	5.12E-06	0 M1 _	Rnd3
Arl1	2.50E-10	0.378406	0.88	0.648	5.04E-06	0 M1 _	Arl1
Ppp1r2	2.44E-10	0.376769	0.898	0.672	4.92E-06	0 M1 _	Ppp1r2

Gadd45a	2.43E-10	0.266596	0.539	0.288	4.90E-06	0 M1 _	Gadd45a
Mtpn	2.36E-10	0.29518	0.76	0.498	4.76E-06	0 M1 _	Mtpn
Gfpt1	2.34E-10	0.325382	0.521	0.288	4.71E-06	0 M1 _	Gfpt1
Ext1	2.28E-10	0.371614	0.599	0.353	4.58E-06	0 M1 _	Ext1
Dnajc25	2.26E-10	0.295884	0.563	0.315	4.56E-06	0 M1 _	Dnajc25
Eif4g2	2.21E-10	0.331426	0.922	0.769	4.44E-06	0 M1 _	Eif4g2
Zfp503	2.16E-10	0.408121	0.653	0.41	4.36E-06	0 M1 _	Zfp503
Srxn1	2.12E-10	0.292786	0.287	0.118	4.27E-06	0 M1 _	Srxn1
Itsn1	1.99E-10	0.271604	0.413	0.204	4.00E-06	0 M1 _	Itsn1
Carm1	1.82E-10	0.258576	0.377	0.174	3.67E-06	0 M1 _	Carm1
Mafb	1.63E-10	0.403294	0.868	0.568	3.28E-06	0 M1 _	Mafb
Kdm7a	1.61E-10	0.267735	0.473	0.236	3.24E-06	0 M1 _	Kdm7a
Smoc2	1.46E-10	0.697342	0.826	0.503	2.94E-06	0 M1 _	Smoc2
Spats2l	1.45E-10	0.31157	0.778	0.472	2.92E-06	0 M1 _	Spats2l
Tgfbr2	1.35E-10	0.407704	0.892	0.635	2.72E-06	0 M1 _	Tgfbr2
Wipi1	1.17E-10	0.366046	0.629	0.383	2.35E-06	0 M1 _	Wipi1
Usp9x	1.09E-10	0.406087	0.766	0.513	2.19E-06	0 M1 _	Usp9x
Cyp26b1	1.06E-10	0.462133	0.263	0.099	2.13E-06	0 M1 _	Cyp26b1
Slc38a10	1.04E-10	0.355253	0.653	0.399	2.09E-06	0 M1 _	Slc38a10
Col4a1	1.01E-10	0.399724	0.982	0.865	2.04E-06	0 M1 _	Col4a1
Reep3	1.00E-10	0.393701	0.91	0.681	2.02E-06	0 M1 _	Reep3
Rnf6	9.97E-11	0.33119	0.623	0.373	2.01E-06	0 M1 _	Rnf6
Map3k7	9.70E-11	0.275826	0.401	0.192	1.95E-06	0 M1 _	Map3k7
Fam91a1	8.83E-11	0.271628	0.425	0.21	1.78E-06	0 M1 _	Fam91a1
Taok1	8.41E-11	0.32921	0.772	0.503	1.69E-06	0 M1 _	Taok1
Csf1	8.26E-11	0.313487	0.659	0.38	1.66E-06	0 M1 _	Csf1
Zbtb4	7.93E-11	0.320854	0.617	0.353	1.60E-06	0 M1 _	Zbtb4
Slc48a1	7.88E-11	0.340898	0.587	0.335	1.59E-06	0 M1 _	Slc48a1
Arap1	7.68E-11	0.344929	0.683	0.396	1.55E-06	0 M1 _	Arap1
Plscr4	7.22E-11	0.325099	0.677	0.402	1.45E-06	0 M1 _	Plscr4
Dnajc3	7.18E-11	0.404332	0.922	0.698	1.45E-06	0 M1 _	Dnajc3
Negr1	7.16E-11	0.256539	0.246	0.091	1.44E-06	0 M1 _	Negr1
Rhoq	6.88E-11	0.302509	0.647	0.392	1.39E-06	0 M1 _	Rhoq
Myadm	6.78E-11	0.295994	0.575	0.32	1.36E-06	0 M1 _	Myadm
BC005537	6.62E-11	0.323535	0.707	0.432	1.33E-06	0 M1 _	BC005537
Acadsb	6.59E-11	0.35656	0.617	0.368	1.33E-06	0 M1 _	Acadsb
Smim1	6.26E-11	0.289525	0.425	0.211	1.26E-06	0 M1 _	Smim1
Wdfy2	6.20E-11	0.255429	0.341	0.15	1.25E-06	0 M1 _	Wdfy2
Snx18	5.98E-11	0.299659	0.665	0.398	1.20E-06	0 M1 _	Snx18
Ugdh	5.45E-11	0.377646	0.449	0.23	1.10E-06	0 M1 _	Ugdh
Tmem53	5.42E-11	0.270954	0.24	0.087	1.09E-06	0 M1 _	Tmem53

Ddah2	4.93E-11	0.391618	0.772	0.505	9.94E-07	0 M1 _	Ddah2
Col1a1	4.75E-11	0.389034	0.988	0.65	9.56E-07	0 M1 _	Col1a1
Mob1a	4.74E-11	0.275927	0.629	0.356	9.54E-07	0 M1 _	Mob1a
Adamtsl2	4.24E-11	0.720112	0.509	0.287	8.54E-07	0 M1 _	Adamtsl2
Vma21	4.03E-11	0.308109	0.587	0.334	8.13E-07	0 M1 _	Vma21
Plxnc1	3.96E-11	0.308207	0.293	0.122	7.98E-07	0 M1 _	Plxnc1
Fasn	3.82E-11	0.328854	0.395	0.182	7.69E-07	0 M1 _	Fasn
Il4ra	3.52E-11	0.287566	0.461	0.231	7.09E-07	0 M1 _	Il4ra
Mmp3	3.40E-11	0.729089	0.599	0.333	6.85E-07	0 M1 _	Mmp3
Rora	3.19E-11	0.394031	0.814	0.506	6.42E-07	0 M1 _	Rora
Tnrc18	2.97E-11	0.359869	0.581	0.325	5.98E-07	0 M1 _	Tnrc18
Epb41l2	2.81E-11	0.375077	0.904	0.663	5.66E-07	0 M1 _	Epb41l2
Dag1	2.45E-11	0.331228	0.838	0.573	4.92E-07	0 M1 _	Dag1
Lpp	2.38E-11	0.324101	0.874	0.612	4.79E-07	0 M1 _	Lpp
Igf1r	2.36E-11	0.353543	0.593	0.338	4.76E-07	0 M1 _	Igf1r
Lmo4	2.34E-11	0.35695	0.796	0.557	4.71E-07	0 M1 _	Lmo4
Peg3	2.23E-11	0.755577	0.371	0.178	4.48E-07	0 M1 _	Peg3
Podn	2.22E-11	0.304435	0.778	0.445	4.48E-07	0 M1 _	Podn
Gm30075	1.63E-11	0.289401	0.353	0.15	3.29E-07	0 M1 _	Gm30075
Fbln7	1.50E-11	0.331748	0.862	0.474	3.02E-07	0 M1 _	Fbln7
Arcn1	1.47E-11	0.371364	0.772	0.51	2.97E-07	0 M1 _	Arcn1
Emp2	1.43E-11	0.35754	0.82	0.543	2.87E-07	0 M1 _	Emp2
Acvr2a	1.41E-11	0.272214	0.365	0.16	2.83E-07	0 M1 _	Acvr2a
Plod2	1.29E-11	0.395691	0.551	0.298	2.60E-07	0 M1 _	Plod2
Acp2	1.27E-11	0.334514	0.467	0.238	2.55E-07	0 M1 _	Acp2
Clcn4	1.18E-11	0.317068	0.725	0.463	2.38E-07	0 M1 _	Clcn4
Agtrap	1.00E-11	0.358189	0.617	0.343	2.02E-07	0 M1 _	Agtrap
Magt1	1.00E-11	0.396568	0.713	0.436	2.02E-07	0 M1 _	Magt1
Hsd11b1	9.99E-12	0.520129	0.796	0.498	2.01E-07	0 M1 _	Hsd11b1
C1qtnf7	9.86E-12	0.27988	0.407	0.181	1.99E-07	0 M1 _	C1qtnf7
Pdia6	9.82E-12	0.482237	0.934	0.723	1.98E-07	0 M1 _	Pdia6
Prrg3	9.49E-12	0.262677	0.359	0.15	1.91E-07	0 M1 _	Prrg3
Mbtps1	9.34E-12	0.324986	0.701	0.419	1.88E-07	0 M1 _	Mbtps1
Nr3c1	8.56E-12	0.388893	0.952	0.721	1.72E-07	0 M1 _	Nr3c1
Tgfb2	8.36E-12	0.367176	0.605	0.331	1.68E-07	0 M1 _	Tgfb2
Rpn2	8.20E-12	0.365354	0.886	0.659	1.65E-07	0 M1 _	Rpn2
Tcf4	7.80E-12	0.443271	0.988	0.881	1.57E-07	0 M1 _	Tcf4
Rrbp1	7.79E-12	0.38054	0.988	0.876	1.57E-07	0 M1 _	Rrbp1
Trps1	7.71E-12	0.309637	0.551	0.296	1.55E-07	0 M1 _	Trps1
Pdpn	7.52E-12	0.385465	0.641	0.361	1.51E-07	0 M1 _	Pdpn
Gnaq	7.30E-12	0.27587	0.731	0.425	1.47E-07	0 M1 _	Gnaq

Atp11a	6.58E-12	0.253412	0.443	0.207	1.33E-07	0 M1 _	Atp11a
Pxdn	6.25E-12	0.308987	0.515	0.266	1.26E-07	0 M1 _	Pxdn
Sobp	6.16E-12	0.304913	0.491	0.242	1.24E-07	0 M1 _	Sobp
Ube4b	5.97E-12	0.288518	0.497	0.253	1.20E-07	0 M1 _	Ube4b
Sod1	5.76E-12	0.320209	0.796	0.514	1.16E-07	0 M1 _	Sod1
Sqor	5.75E-12	0.282899	0.431	0.203	1.16E-07	0 M1 _	Sqor
Rgma	5.63E-12	0.386839	0.617	0.361	1.13E-07	0 M1 _	Rgma
Acap2	5.46E-12	0.380091	0.808	0.542	1.10E-07	0 M1 _	Acap2
Cdk14	5.44E-12	0.365532	0.683	0.401	1.10E-07	0 M1 _	Cdk14
Tmem263	5.31E-12	0.367657	0.581	0.307	1.07E-07	0 M1 _	Tmem263
Smarcd2	4.81E-12	0.272776	0.467	0.227	9.68E-08	0 M1 _	Smarcd2
Rpn1	4.50E-12	0.386729	0.844	0.609	9.06E-08	0 M1 _	Rpn1
Chpf	4.08E-12	0.392625	0.754	0.456	8.23E-08	0 M1 _	Chpf
Tiparp	3.93E-12	0.276135	0.401	0.18	7.92E-08	0 M1 _	Tiparp
Tmem106b	3.88E-12	0.337263	0.701	0.418	7.82E-08	0 M1 _	Tmem106b
Cd81	3.86E-12	0.417246	0.994	0.889	7.78E-08	0 M1 _	Cd81
Gulp1	3.72E-12	0.294679	0.629	0.347	7.49E-08	0 M1 _	Gulp1
Sgpl1	2.85E-12	0.283117	0.461	0.216	5.74E-08	0 M1 _	Sgpl1
Cyth3	2.74E-12	0.385909	0.922	0.652	5.51E-08	0 M1 _	Cyth3
Ank3	2.60E-12	0.374337	0.527	0.271	5.23E-08	0 M1 _	Ank3
Fkbp10	2.53E-12	0.258411	0.569	0.287	5.09E-08	0 M1 _	Fkbp10
Tmem184c	2.40E-12	0.263904	0.377	0.16	4.83E-08	0 M1 _	Tmem184c
Irs2	2.27E-12	0.282177	0.419	0.189	4.58E-08	0 M1 _	Irs2
Selenot	2.12E-12	0.305731	0.671	0.371	4.28E-08	0 M1 _	Selenot
Sgce	2.01E-12	0.336368	0.689	0.404	4.05E-08	0 M1 _	Sgce
Emp3	2.01E-12	0.437544	0.964	0.794	4.04E-08	0 M1 _	Emp3
Ccdc8	1.97E-12	0.264549	0.335	0.136	3.98E-08	0 M1 _	Ccdc8
Itgav	1.84E-12	0.331048	0.766	0.462	3.71E-08	0 M1 _	Itgav
Map1a	1.51E-12	0.303866	0.503	0.245	3.04E-08	0 M1 _	Map1a
Arl8b	1.49E-12	0.32389	0.593	0.311	3.01E-08	0 M1 _	Arl8b
Aff1	1.49E-12	0.316616	0.449	0.206	2.99E-08	0 M1 _	Aff1
Notch2	1.48E-12	0.26938	0.437	0.196	2.99E-08	0 M1 _	Notch2
Ifi211	1.37E-12	0.428869	0.605	0.342	2.76E-08	0 M1 _	Ifi211
Plekhf1	1.20E-12	0.309941	0.485	0.237	2.42E-08	0 M1 _	Plekhf1
Rgs7bp	1.15E-12	0.282562	0.365	0.148	2.33E-08	0 M1 _	Rgs7bp
Arhgap24	1.13E-12	0.350727	0.425	0.195	2.27E-08	0 M1 _	Arhgap24
Ccl8	1.03E-12	0.383702	0.287	0.105	2.07E-08	0 M1 _	Ccl8
Fkbp7	9.63E-13	0.447613	0.85	0.546	1.94E-08	0 M1 _	Fkbp7
Mn1	9.20E-13	0.279354	0.287	0.103	1.85E-08	0 M1 _	Mn1
Cbx3	9.07E-13	0.409879	0.934	0.716	1.83E-08	0 M1 _	Cbx3
Slc43a3	8.32E-13	0.307814	0.784	0.488	1.68E-08	0 M1 _	Slc43a3

Ptp4a2	8.20E-13	0.439022	0.88	0.703	1.65E-08	0 M1 _	Ptp4a2
Copb2	8.10E-13	0.468214	0.79	0.545	1.63E-08	0 M1 _	Copb2
Kdelr2	6.70E-13	0.427796	0.874	0.551	1.35E-08	0 M1 _	Kdelr2
Zadh2	6.51E-13	0.259905	0.467	0.216	1.31E-08	0 M1 _	Zadh2
Spry1	5.80E-13	0.430363	0.808	0.517	1.17E-08	0 M1 _	Spry1
Man1a	5.77E-13	0.485865	0.581	0.32	1.16E-08	0 M1 _	Man1a
Fgd4	5.50E-13	0.286645	0.365	0.148	1.11E-08	0 M1 _	Fgd4
Sgcb	5.42E-13	0.325985	0.605	0.317	1.09E-08	0 M1 _	Sgcb
Rev3l	5.28E-13	0.261538	0.479	0.219	1.06E-08	0 M1 _	Rev3l
Tlr4	4.44E-13	0.252348	0.293	0.105	8.95E-09	0 M1 _	Tlr4
S1pr2	4.32E-13	0.271943	0.491	0.232	8.70E-09	0 M1 _	S1pr2
Sult5a1	4.24E-13	0.35806	0.353	0.142	8.54E-09	0 M1 _	Sult5a1
Thbd	4.10E-13	0.703324	0.641	0.399	8.25E-09	0 M1 _	Thbd
Lama4	4.08E-13	0.49543	0.79	0.5	8.22E-09	0 M1 _	Lama4
Fam114a1	4.08E-13	0.41626	0.737	0.444	8.21E-09	0 M1 _	Fam114a1
Klhl13	3.95E-13	0.262897	0.347	0.137	7.96E-09	0 M1 _	Klhl13
Lrrc58	3.93E-13	0.449355	0.904	0.655	7.91E-09	0 M1 _	Lrrc58
Glis2	2.70E-13	0.279854	0.431	0.189	5.43E-09	0 M1 _	Glis2
Tmem63a	2.64E-13	0.293424	0.515	0.24	5.32E-09	0 M1 _	Tmem63a
Slfn5	2.32E-13	0.397785	0.916	0.701	4.68E-09	0 M1 _	Slfn5
Ltbp3	2.03E-13	0.365602	0.754	0.419	4.09E-09	0 M1 _	Ltbp3
Selenon	1.96E-13	0.329482	0.539	0.265	3.95E-09	0 M1 _	Selenon
Tgoln1	1.89E-13	0.395542	0.82	0.548	3.81E-09	0 M1 _	Tgoln1
Setd7	1.89E-13	0.348049	0.569	0.294	3.81E-09	0 M1 _	Setd7
Chp1	1.85E-13	0.393323	0.491	0.249	3.72E-09	0 M1 _	Chp1
Zfp592	1.74E-13	0.286678	0.419	0.184	3.51E-09	0 M1 _	Zfp592
Serping1	1.66E-13	0.484398	0.988	0.74	3.35E-09	0 M1 _	Serping1
Vim	1.62E-13	0.562593	0.988	0.877	3.27E-09	0 M1 _	Vim
Pank3	1.59E-13	0.289248	0.491	0.224	3.20E-09	0 M1 _	Pank3
Lamp2	1.41E-13	0.412471	0.97	0.738	2.85E-09	0 M1 _	Lamp2
Mab21l2	1.41E-13	0.288772	0.275	0.095	2.85E-09	0 M1 _	Mab21l2
Pttg1ip	1.32E-13	0.396416	0.898	0.634	2.66E-09	0 M1 _	Pttg1ip
Runx1t1	1.29E-13	0.39732	0.569	0.295	2.60E-09	0 M1 _	Runx1t1
Col23a1	1.28E-13	0.313127	0.317	0.118	2.57E-09	0 M1 _	Col23a1
Ywhag	1.21E-13	0.325917	0.635	0.346	2.43E-09	0 M1 _	Ywhag
Cped1	1.03E-13	0.435527	0.952	0.68	2.07E-09	0 M1 _	Cped1
Itpripl2	8.80E-14	0.417359	0.802	0.545	1.77E-09	0 M1 _	Itpripl2
Fmnl2	8.78E-14	0.359235	0.539	0.271	1.77E-09	0 M1 _	Fmnl2
Itga8	8.76E-14	0.276825	0.443	0.185	1.76E-09	0 M1 _	Itga8
Stt3a	8.72E-14	0.382967	0.743	0.451	1.76E-09	0 M1 _	Stt3a
Mlec	8.59E-14	0.408515	0.808	0.518	1.73E-09	0 M1 _	Mlec

Hunk	8.27E-14	0.289456	0.222	0.064	1.67E-09	0 M1 _	Hunk
Serpinf1	7.60E-14	0.584958	0.988	0.568	1.53E-09	0 M1 _	Serpinf1
Dsel	7.38E-14	0.360749	0.581	0.299	1.49E-09	0 M1 _	Dsel
Dpy19l1	7.02E-14	0.356736	0.569	0.294	1.41E-09	0 M1 _	Dpy19l1
Prelp	6.28E-14	0.423839	0.862	0.506	1.26E-09	0 M1 _	Prelp
Olfml2b	5.97E-14	0.269004	0.329	0.116	1.20E-09	0 M1 _	Olfml2b
Cdh19	5.82E-14	0.250078	0.353	0.133	1.17E-09	0 M1 _	Cdh19
S100a6	4.80E-14	0.486608	0.994	0.923	9.67E-10	0 M1 _	S100a6
Morf4l2	4.62E-14	0.427786	0.844	0.568	9.30E-10	0 M1 _	Morf4l2
S100a10	4.39E-14	0.555504	0.94	0.674	8.84E-10	0 M1 _	S100a10
Adcy5	3.55E-14	0.277437	0.329	0.121	7.16E-10	0 M1 _	Adcy5
Tpcn1	3.40E-14	0.279887	0.461	0.202	6.84E-10	0 M1 _	Tpcn1
Sh3pxd2a	3.38E-14	0.411843	0.683	0.387	6.81E-10	0 M1 _	Sh3pxd2a
Abl1	3.37E-14	0.269887	0.521	0.242	6.79E-10	0 M1 _	Abl1
C1qtnf1	3.11E-14	0.252259	0.389	0.15	6.26E-10	0 M1 _	C1qtnf1
Tslp	2.98E-14	0.307799	0.347	0.132	6.01E-10	0 M1 _	Tslp
Txndc5	2.86E-14	0.370994	0.76	0.428	5.76E-10	0 M1 _	Txndc5
Zfhx4	2.58E-14	0.415778	0.701	0.391	5.20E-10	0 M1 _	Zfhx4
Extl3	2.51E-14	0.350406	0.587	0.301	5.05E-10	0 M1 _	Extl3
Tmem123	2.48E-14	0.3303	0.635	0.338	4.99E-10	0 M1 _	Tmem123
Aldh7a1	2.20E-14	0.459823	0.593	0.331	4.42E-10	0 M1 _	Aldh7a1
Adamtsl1	2.06E-14	0.515243	0.617	0.325	4.15E-10	0 M1 _	Adamtsl1
Lrp3	2.04E-14	0.287521	0.443	0.183	4.11E-10	0 M1 _	Lrp3
Asph	1.92E-14	0.350842	0.701	0.379	3.88E-10	0 M1 _	Asph
Samd4b	1.89E-14	0.2846	0.599	0.286	3.81E-10	0 M1 _	Samd4b
Cbr3	1.78E-14	0.452273	0.629	0.34	3.59E-10	0 M1 _	Cbr3
Obsl1	1.77E-14	0.264889	0.353	0.135	3.56E-10	0 M1 _	Obsl1
Basp1	1.57E-14	0.461535	0.665	0.355	3.17E-10	0 M1 _	Basp1
Fstl1	1.35E-14	0.524186	0.982	0.678	2.72E-10	0 M1 _	Fstl1
Tshz1	1.30E-14	0.338861	0.497	0.23	2.61E-10	0 M1 _	Tshz1
Vangl1	1.23E-14	0.262382	0.515	0.234	2.48E-10	0 M1 _	Vangl1
Flrt2	1.22E-14	0.264131	0.725	0.354	2.45E-10	0 M1 _	Flrt2
Idh1	1.21E-14	0.432294	0.617	0.332	2.44E-10	0 M1 _	Idh1
Ckap4	8.96E-15	0.445442	0.832	0.501	1.80E-10	0 M1 _	Ckap4
Bicc1	8.88E-15	0.454865	0.952	0.571	1.79E-10	0 M1 _	Bicc1
8-Sep	7.69E-15	0.388572	0.689	0.37	1.55E-10	0 M1 _	8-Sep
Ifi27	7.32E-15	0.498041	0.946	0.785	1.47E-10	0 M1 _	Ifi27
Rgmb	6.98E-15	0.368858	0.575	0.285	1.41E-10	0 M1 _	Rgmb
Dock7	6.98E-15	0.327254	0.617	0.303	1.40E-10	0 M1 _	Dock7
Myh10	6.73E-15	0.424193	0.479	0.218	1.36E-10	0 M1 _	Myh10
Efs	5.99E-15	0.270888	0.293	0.098	1.21E-10	0 M1 _	Efs

Tmem106a	5.58E-15	0.311465	0.365	0.14	1.12E-10	0 M1 _	Tmem106a
Jade1	4.62E-15	0.425283	0.617	0.331	9.30E-11	0 M1 _	Jade1
Lima1	4.40E-15	0.552748	0.868	0.59	8.86E-11	0 M1 _	Lima1
Fads1	3.09E-15	0.441855	0.569	0.294	6.23E-11	0 M1 _	Fads1
Lpgat1	2.45E-15	0.37922	0.611	0.303	4.94E-11	0 M1 _	Lpgat1
Ergic1	2.13E-15	0.375452	0.581	0.291	4.28E-11	0 M1 _	Ergic1
Impad1	2.02E-15	0.396051	0.665	0.35	4.06E-11	0 M1 _	Impad1
Fkbp9	1.97E-15	0.416163	0.856	0.508	3.96E-11	0 M1 _	Fkbp9
Ski	1.96E-15	0.442536	0.838	0.539	3.94E-11	0 M1 _	Ski
Rbfox2	1.96E-15	0.390652	0.689	0.377	3.94E-11	0 M1 _	Rbfox2
Rnf145	1.82E-15	0.295973	0.449	0.186	3.67E-11	0 M1 _	Rnf145
Pcolce	1.75E-15	0.543779	0.988	0.736	3.52E-11	0 M1 _	Pcolce
Serinc1	1.57E-15	0.502372	0.922	0.709	3.16E-11	0 M1 _	Serinc1
Apod	1.55E-15	0.39124	0.982	0.625	3.12E-11	0 M1 _	Apod
Fkbp14	1.55E-15	0.312177	0.455	0.193	3.12E-11	0 M1 _	Fkbp14
Dnajc10	1.28E-15	0.40979	0.808	0.471	2.58E-11	0 M1 _	Dnajc10
Rhobtb1	1.17E-15	0.259422	0.275	0.085	2.35E-11	0 M1 _	Rhobtb1
Prrx2	1.11E-15	0.397816	0.617	0.3	2.23E-11	0 M1 _	Prrx2
Nectin1	1.04E-15	0.317282	0.365	0.136	2.09E-11	0 M1 _	Nectin1
Irf2bp2	9.41E-16	0.54036	0.856	0.568	1.90E-11	0 M1 _	Irf2bp2
Reep5	8.33E-16	0.525141	0.97	0.727	1.68E-11	0 M1 _	Reep5
Abca1	7.32E-16	0.45805	0.665	0.348	1.47E-11	0 M1 _	Abca1
Irak1	6.35E-16	0.446384	0.683	0.385	1.28E-11	0 M1 _	Irak1
Nav3	5.36E-16	0.411328	0.647	0.323	1.08E-11	0 M1 _	Nav3
Col27a1	5.02E-16	0.477552	0.617	0.316	1.01E-11	0 M1 _	Col27a1
Prrx1	4.64E-16	0.522448	0.958	0.7	9.36E-12	0 M1 _	Prrx1
Pik3r2	3.91E-16	0.334824	0.521	0.232	7.88E-12	0 M1 _	Pik3r2
Gfpt2	3.85E-16	0.48297	0.599	0.301	7.75E-12	0 M1 _	Gfpt2
Twist1	3.40E-16	0.474043	0.784	0.457	6.86E-12	0 M1 _	Twist1
Aoc3	2.81E-16	0.254876	0.473	0.183	5.67E-12	0 M1 _	Aoc3
Arhgap20	2.73E-16	0.44008	0.509	0.225	5.50E-12	0 M1 _	Arhgap20
Boc	2.70E-16	0.385015	0.461	0.194	5.44E-12	0 M1 _	Boc
Oat	2.33E-16	0.453761	0.952	0.621	4.70E-12	0 M1 _	Oat
Ppp3ca	2.06E-16	0.520259	0.784	0.497	4.14E-12	0 M1 _	Ppp3ca
Naaa	1.78E-16	0.347354	0.335	0.116	3.59E-12	0 M1 _	Naaa
Olfml2a	1.77E-16	0.397567	0.425	0.172	3.57E-12	0 M1 _	Olfml2a
Tuba1a	1.74E-16	0.508306	0.976	0.819	3.50E-12	0 M1 _	Tuba1a
Slc29a1	1.58E-16	0.582896	0.862	0.596	3.19E-12	0 M1 _	Slc29a1
Vldlr	1.52E-16	0.31019	0.228	0.058	3.05E-12	0 M1 _	Vldlr
Mt1	1.45E-16	0.820666	0.952	0.6	2.91E-12	0 M1 _	Mt1
Slc16a2	1.26E-16	0.458466	0.599	0.297	2.53E-12	0 M1 _	Slc16a2

Sec61a1	1.17E-16	0.481242	0.778	0.476	2.36E-12	0 M1 _	Sec61a1
Eps15	1.13E-16	0.444044	0.653	0.343	2.28E-12	0 M1 _	Eps15
Por	1.13E-16	0.431895	0.653	0.329	2.27E-12	0 M1 _	Por
Sec24d	9.99E-17	0.375398	0.611	0.301	2.01E-12	0 M1 _	Sec24d
Fbln1	9.89E-17	0.593274	0.916	0.506	1.99E-12	0 M1 _	Fbln1
Igf2r	8.37E-17	0.614263	0.695	0.405	1.68E-12	0 M1 _	Igf2r
Trabd2b	7.33E-17	0.263189	0.323	0.101	1.48E-12	0 M1 _	Trabd2b
Cd44	6.60E-17	0.382242	0.455	0.187	1.33E-12	0 M1 _	Cd44
Stat5b	6.49E-17	0.3112	0.479	0.195	1.31E-12	0 M1 _	Stat5b
Ptgir	6.40E-17	0.399219	0.467	0.194	1.29E-12	0 M1 _	Ptgir
Cdr2l	5.05E-17	0.264384	0.431	0.162	1.02E-12	0 M1 _	Cdr2l
Arhgap21	3.65E-17	0.420579	0.491	0.211	7.35E-13	0 M1 _	Arhgap21
Dmpk	3.65E-17	0.275804	0.323	0.1	7.35E-13	0 M1 _	Dmpk
Ssr3	2.82E-17	0.486196	0.874	0.559	5.69E-13	0 M1 _	Ssr3
Tppp3	2.77E-17	1.211634	0.413	0.176	5.58E-13	0 M1 _	Tppp3
Ahnak	2.48E-17	0.580946	0.988	0.888	5.00E-13	0 M1 _	Ahnak
Sar1a	2.43E-17	0.509941	0.88	0.567	4.90E-13	0 M1 _	Sar1a
Col6a6	2.40E-17	0.731964	0.605	0.301	4.82E-13	0 M1 _	Col6a6
Tram1	2.16E-17	0.514602	0.88	0.582	4.34E-13	0 M1 _	Tram1
Fkbp2	2.08E-17	0.531936	0.856	0.548	4.20E-13	0 M1 _	Fkbp2
Tubb6	2.00E-17	0.451903	0.617	0.298	4.03E-13	0 M1 _	Tubb6
Fam111a	1.71E-17	0.361044	0.539	0.234	3.44E-13	0 M1 _	Fam111a
Vwa5a	1.62E-17	0.372517	0.653	0.33	3.26E-13	0 M1 _	Vwa5a
Ftl1	1.48E-17	0.448882	1	0.96	2.99E-13	0 M1 _	Ftl1
Arrdc3	1.39E-17	0.376407	0.509	0.21	2.80E-13	0 M1 _	Arrdc3
Hook3	1.31E-17	0.499406	0.916	0.633	2.64E-13	0 M1 _	Hook3
Pam	1.27E-17	0.650834	0.808	0.503	2.56E-13	0 M1 _	Pam
Golph3	1.22E-17	0.407698	0.659	0.322	2.45E-13	0 M1 _	Golph3
Pdia4	9.29E-18	0.513024	0.713	0.399	1.87E-13	0 M1 _	Pdia4
Il1r1	9.22E-18	0.507709	0.79	0.434	1.86E-13	0 M1 _	Il1r1
Krt10	9.14E-18	0.287841	0.401	0.144	1.84E-13	0 M1 _	Krt10
Slc39a1	8.83E-18	0.509465	0.743	0.426	1.78E-13	0 M1 _	Slc39a1
Aldh1a1	8.54E-18	0.35464	0.683	0.322	1.72E-13	0 M1 _	Aldh1a1
Psap	7.66E-18	0.460347	0.982	0.848	1.54E-13	0 M1 _	Psap
Eps8	6.71E-18	0.43364	0.743	0.403	1.35E-13	0 M1 _	Eps8
Pmp22	5.42E-18	0.523955	0.94	0.69	1.09E-13	0 M1 _	Pmp22
Tgfbi	4.07E-18	0.818401	0.754	0.413	8.21E-14	0 M1 _	Tgfbi
Canx	3.40E-18	0.538714	0.934	0.721	6.85E-14	0 M1 _	Canx
mt-Nd4l	3.19E-18	0.588582	0.97	0.817	6.43E-14	0 M1 _	mt-Nd4l
Abca6	2.91E-18	0.298158	0.341	0.108	5.86E-14	0 M1 _	Abca6
Cd9	2.50E-18	0.60522	0.946	0.708	5.03E-14	0 M1 _	Cd9

Foxo3	2.05E-18	0.309733	0.509	0.207	4.12E-14	0 M1 _	Foxo3
Txn1	1.91E-18	0.560895	0.958	0.824	3.85E-14	0 M1 _	Txn1
Surf4	1.75E-18	0.553466	0.832	0.528	3.52E-14	0 M1 _	Surf4
Dpysl2	1.61E-18	0.476748	0.778	0.456	3.25E-14	0 M1 _	Dpysl2
Plod1	1.60E-18	0.546245	0.844	0.525	3.22E-14	0 M1 _	Plod1
Atp2a2	1.54E-18	0.499217	0.934	0.665	3.11E-14	0 M1 _	Atp2a2
Mt2	1.34E-18	0.943851	0.677	0.32	2.69E-14	0 M1 _	Mt2
Dse	9.38E-19	0.432197	0.581	0.262	1.89E-14	0 M1 _	Dse
Adamts1	7.57E-19	0.645582	0.659	0.319	1.52E-14	0 M1 _	Adamts1
Armcx2	6.74E-19	0.347099	0.491	0.195	1.36E-14	0 M1 _	Armcx2
Bmpr1a	6.64E-19	0.518404	0.695	0.355	1.34E-14	0 M1 _	Bmpr1a
Cdon	6.63E-19	0.347749	0.317	0.094	1.34E-14	0 M1 _	Cdon
Pnp	6.32E-19	0.612799	0.862	0.562	1.27E-14	0 M1 _	Pnp
Marcks	6.15E-19	0.613721	0.988	0.811	1.24E-14	0 M1 _	Marcks
Tmem119	6.10E-19	0.595329	0.707	0.361	1.23E-14	0 M1 _	Tmem119
Zhx3	5.54E-19	0.468094	0.635	0.308	1.12E-14	0 M1 _	Zhx3
Colec12	5.33E-19	0.581703	0.952	0.544	1.07E-14	0 M1 _	Colec12
Col5a2	5.06E-19	0.617845	0.97	0.628	1.02E-14	0 M1 _	Col5a2
Hikeshi	4.77E-19	0.443394	0.689	0.362	9.60E-15	0 M1 _	Hikeshi
Nxf1	4.76E-19	0.402334	0.575	0.253	9.58E-15	0 M1 _	Nxf1
Il6st	3.53E-19	0.528713	0.856	0.558	7.12E-15	0 M1 _	Il6st
Nr2f1	3.41E-19	0.669129	0.886	0.579	6.87E-15	0 M1 _	Nr2f1
Tbc1d2b	2.96E-19	0.417185	0.689	0.337	5.96E-15	0 M1 _	Tbc1d2b
Lrp6	2.62E-19	0.536027	0.754	0.433	5.29E-15	0 M1 _	Lrp6
Glg1	2.57E-19	0.534134	0.916	0.637	5.18E-15	0 M1 _	Glg1
Dclk1	2.51E-19	0.60264	0.491	0.2	5.06E-15	0 M1 _	Dclk1
Col3a1	2.44E-19	0.673313	0.988	0.74	4.91E-15	0 M1 _	Col3a1
Npas2	2.31E-19	0.255196	0.341	0.104	4.65E-15	0 M1 _	Npas2
Sash1	2.29E-19	0.559735	0.844	0.559	4.60E-15	0 M1 _	Sash1
Dcun1d3	2.24E-19	0.394106	0.347	0.112	4.52E-15	0 M1 _	Dcun1d3
Cd47	1.95E-19	0.504275	0.982	0.854	3.92E-15	0 M1 _	Cd47
Fgf7	1.52E-19	0.346523	0.551	0.221	3.05E-15	0 M1 _	Fgf7
Fap	1.41E-19	0.501526	0.826	0.447	2.84E-15	0 M1 _	Fap
Ssr1	1.11E-19	0.574286	0.814	0.505	2.23E-15	0 M1 _	Ssr1
Uap1	1.08E-19	0.456371	0.563	0.245	2.18E-15	0 M1 _	Uap1
Maged2	8.84E-20	0.546627	0.76	0.412	1.78E-15	0 M1 _	Maged2
Akap12	8.71E-20	0.744988	0.838	0.523	1.75E-15	0 M1 _	Akap12
Ssh2	7.17E-20	0.653575	0.766	0.458	1.44E-15	0 M1 _	Ssh2
Ncoa1	5.99E-20	0.422618	0.545	0.228	1.21E-15	0 M1 _	Ncoa1
Smad3	5.99E-20	0.389671	0.503	0.202	1.21E-15	0 M1 _	Smad3
Srgap1	5.64E-20	0.26431	0.251	0.06	1.14E-15	0 M1 _	Srgap1

Ecm1	4.24E-20	0.739364	0.784	0.461	8.53E-16	0 M1 _	Ecm1
Gpc3	3.12E-20	0.376386	0.281	0.07	6.29E-16	0 M1 _	Gpc3
Scarb2	2.41E-20	0.590698	0.856	0.521	4.86E-16	0 M1 _	Scarb2
Gnas	2.25E-20	0.415036	1	0.959	4.53E-16	0 M1 _	Gnas
Fam129a	1.91E-20	0.529736	0.707	0.373	3.85E-16	0 M1 _	Fam129a
Angptl4	1.57E-20	0.322589	0.275	0.068	3.16E-16	0 M1 _	Angptl4
Gdf10	1.32E-20	0.643176	0.341	0.103	2.67E-16	0 M1 _	Gdf10
Adgra2	1.00E-20	0.456788	0.563	0.237	2.02E-16	0 M1 _	Adgra2
Steap4	8.55E-21	0.27817	0.832	0.422	1.72E-16	0 M1 _	Steap4
Cat	7.04E-21	0.522869	0.605	0.274	1.42E-16	0 M1 _	Cat
Gja1	5.68E-21	0.640662	0.671	0.33	1.14E-16	0 M1 _	Gja1
Flnc	5.36E-21	0.339696	0.347	0.103	1.08E-16	0 M1 _	Flnc
Adamts5	3.65E-21	0.688397	0.904	0.497	7.35E-17	0 M1 _	Adamts5
Tmem198b	3.05E-21	0.268886	0.335	0.096	6.15E-17	0 M1 _	Tmem198b
Thbs1	3.01E-21	0.651933	0.557	0.231	6.07E-17	0 M1 _	Thbs1
Col1a2	2.35E-21	0.753453	0.994	0.733	4.73E-17	0 M1 _	Col1a2
Lifr	2.27E-21	0.741901	0.707	0.389	4.58E-17	0 M1 _	Lifr
G6pdx	1.73E-21	0.335294	0.401	0.128	3.48E-17	0 M1 _	G6pdx
Fth1	1.55E-21	0.550057	0.994	0.993	3.13E-17	0 M1 _	Fth1
Abca9	1.49E-21	0.561376	0.653	0.302	3.00E-17	0 M1 _	Abca9
Bmp1	1.44E-21	0.564674	0.766	0.418	2.91E-17	0 M1 _	Bmp1
Fgf18	1.25E-21	0.334461	0.305	0.081	2.52E-17	0 M1 _	Fgf18
Cpq	1.23E-21	0.650541	0.82	0.434	2.47E-17	0 M1 _	Cpq
Grb10	1.23E-21	0.631652	0.671	0.333	2.47E-17	0 M1 _	Grb10
Fam171b	1.20E-21	0.466961	0.371	0.116	2.41E-17	0 M1 _	Fam171b
Reck	1.06E-21	0.426034	0.521	0.202	2.14E-17	0 M1 _	Reck
Gstm2	7.38E-22	0.674125	0.796	0.464	1.49E-17	0 M1 _	Gstm2
Nfic	7.14E-22	0.675465	0.958	0.699	1.44E-17	0 M1 _	Nfic
Atrnl1	5.78E-22	0.395625	0.497	0.185	1.16E-17	0 M1 _	Atrnl1
Slc16a9	5.71E-22	0.340124	0.491	0.169	1.15E-17	0 M1 _	Slc16a9
Eif4g3	4.74E-22	0.679851	0.886	0.602	9.55E-18	0 M1 _	Eif4g3
Calr	4.73E-22	0.638024	0.982	0.866	9.52E-18	0 M1 _	Calr
Dennd2a	4.64E-22	0.39944	0.581	0.235	9.34E-18	0 M1 _	Dennd2a
H6pd	3.83E-22	0.467997	0.641	0.278	7.72E-18	0 M1 _	H6pd
Cacna1a	3.58E-22	0.478431	0.497	0.189	7.21E-18	0 M1 _	Cacna1a
Arhgap32	3.41E-22	0.463225	0.479	0.178	6.87E-18	0 M1 _	Arhgap32
Hgsnat	3.32E-22	0.48698	0.683	0.327	6.69E-18	0 M1 _	Hgsnat
Sdc2	3.03E-22	0.650443	0.91	0.536	6.11E-18	0 M1 _	Sdc2
Gstm1	1.63E-22	0.734664	0.91	0.647	3.29E-18	0 M1 _	Gstm1
P4hb	1.54E-22	0.635424	0.952	0.676	3.10E-18	0 M1 _	P4hb
Maged1	1.44E-22	0.604048	0.904	0.529	2.90E-18	0 M1 _	Maged1

Cyb5a	1.33E-22	0.57813	0.982	0.756	2.68E-18	0 M1 _	Cyb5a
Sh3d19	1.23E-22	0.621614	0.743	0.387	2.48E-18	0 M1 _	Sh3d19
Col6a1	9.27E-23	0.6889	0.982	0.693	1.87E-18	0 M1 _	Col6a1
Abcc4	9.22E-23	0.308773	0.323	0.084	1.86E-18	0 M1 _	Abcc4
Bmper	9.20E-23	0.321729	0.269	0.061	1.85E-18	0 M1 _	Bmper
Slit3	8.28E-23	0.538639	0.725	0.355	1.67E-18	0 M1 _	Slit3
Rhobtb3	3.80E-23	0.599995	0.719	0.353	7.66E-19	0 M1 _	Rhobtb3
Cxcl14	3.29E-23	1.108858	0.886	0.596	6.63E-19	0 M1 _	Cxcl14
9330159F19Rik	2.65E-23	0.29297	0.192	0.031	5.33E-19	0 M1 _	9330159F19Rik
Lgi2	1.77E-23	0.445149	0.371	0.106	3.57E-19	0 M1 _	Lgi2
Spag7	1.45E-23	0.35293	0.479	0.161	2.92E-19	0 M1 _	Spag7
Sema3a	9.19E-24	0.495632	0.479	0.166	1.85E-19	0 M1 _	Sema3a
Mxra8	7.85E-24	0.721046	0.946	0.6	1.58E-19	0 M1 _	Mxra8
Dpysl3	5.95E-24	0.682463	0.91	0.49	1.20E-19	0 M1 _	Dpysl3
Lgi4	5.08E-24	0.416336	0.413	0.133	1.02E-19	0 M1 _	Lgi4
Bcl6	4.54E-24	0.397417	0.437	0.14	9.14E-20	0 M1 _	Bcl6
Adamtsl3	3.91E-24	0.507769	0.371	0.108	7.88E-20	0 M1 _	Adamtsl3
Nfib	3.85E-24	0.606699	0.958	0.746	7.76E-20	0 M1 _	Nfib
Ifngr1	3.27E-24	0.772974	0.94	0.718	6.59E-20	0 M1 _	Ifngr1
Reps2	2.94E-24	0.355325	0.371	0.104	5.93E-20	0 M1 _	Reps2
Mrap	2.46E-24	0.414314	0.311	0.076	4.95E-20	0 M1 _	Mrap
Tshz3	2.36E-24	0.504554	0.605	0.246	4.75E-20	0 M1 _	Tshz3
Maob	2.25E-24	0.270108	0.216	0.038	4.52E-20	0 M1 _	Maob
Ank2	1.97E-24	0.529205	0.689	0.306	3.96E-20	0 M1 _	Ank2
Ccdc71l	1.76E-24	0.509146	0.575	0.231	3.54E-20	0 M1 _	Ccdc71l
Nav1	1.13E-24	0.750722	0.934	0.597	2.27E-20	0 M1 _	Nav1
Lpl	1.01E-24	0.891074	0.982	0.662	2.03E-20	0 M1 _	Lpl
Pcdh7	7.51E-25	0.655289	0.725	0.349	1.51E-20	0 M1 _	Pcdh7
Itm2a	4.69E-25	0.846233	0.94	0.609	9.45E-21	0 M1 _	Itm2a
Auts2	4.53E-25	0.716802	0.844	0.461	9.12E-21	0 M1 _	Auts2
AY036118	4.35E-25	1.356401	0.88	0.543	8.76E-21	0 M1 _	AY036118
G0s2	4.27E-25	0.689154	0.886	0.495	8.61E-21	0 M1 _	G0s2
Arl5a	3.94E-25	0.609838	0.713	0.346	7.94E-21	0 M1 _	Arl5a
Pdxk	3.74E-25	0.357442	0.353	0.092	7.54E-21	0 M1 _	Pdxk
Vcan	2.96E-25	0.75262	0.659	0.281	5.95E-21	0 M1 _	Vcan
St3gal1	2.57E-25	0.597696	0.551	0.211	5.19E-21	0 M1 _	St3gal1
Stt3b	2.26E-25	0.633897	0.689	0.329	4.55E-21	0 M1 _	Stt3b
Ifi204	2.05E-25	0.717766	0.707	0.332	4.13E-21	0 M1 _	Ifi204
Adcyap1r1	1.95E-25	0.489608	0.635	0.258	3.92E-21	0 M1 _	Adcyap1r1
Cnmd	1.89E-25	0.628366	0.138	0.014	3.80E-21	0 M1 _	Cnmd
Gm42418	1.66E-25	0.99898	1	0.999	3.35E-21	0 M1 _	Gm42418

Cyp2f2	1.59E-25	0.630694	0.18	0.025	3.19E-21	0 M1 _	Cyp2f2
Glipr2	1.41E-25	0.494936	0.437	0.137	2.85E-21	0 M1 _	Glipr2
F3	1.40E-25	0.643631	0.647	0.257	2.81E-21	0 M1 _	F3
Fbln2	1.39E-25	1.198837	0.838	0.524	2.80E-21	0 M1 _	Fbln2
Nfasc	7.27E-26	0.351495	0.311	0.072	1.46E-21	0 M1 _	Nfasc
Fcgr2b	5.11E-26	0.797713	0.437	0.143	1.03E-21	0 M1 _	Fcgr2b
Ctsh	4.88E-26	0.7373	0.916	0.545	9.82E-22	0 M1 _	Ctsh
Ramp2	3.78E-26	0.569597	0.874	0.469	7.61E-22	0 M1 _	Ramp2
Ltbp4	3.74E-26	0.862388	0.928	0.652	7.54E-22	0 M1 _	Ltbp4
Zim1	1.69E-26	0.341704	0.18	0.024	3.41E-22	0 M1 _	Zim1
Ptgfrn	1.54E-26	0.720568	0.766	0.378	3.10E-22	0 M1 _	Ptgfrn
Fez1	1.27E-26	0.331511	0.269	0.053	2.57E-22	0 M1 _	Fez1
Serpinh1	1.01E-26	0.76085	0.994	0.798	2.03E-22	0 M1 _	Serpinh1
Col6a2	5.34E-27	0.817646	0.976	0.643	1.07E-22	0 M1 _	Col6a2
Serpinb6a	5.04E-27	0.750541	0.952	0.695	1.01E-22	0 M1 _	Serpinb6a
Ggt5	4.89E-27	0.761635	0.713	0.337	9.84E-23	0 M1 _	Ggt5
Ctsl	4.22E-27	0.745146	0.994	0.798	8.50E-23	0 M1 _	Ctsl
Fkbp5	6.84E-28	0.622991	0.599	0.236	1.38E-23	0 M1 _	Fkbp5
Twsg1	6.64E-28	0.637399	0.743	0.352	1.34E-23	0 M1 _	Twsg1
Fhl1	5.35E-28	0.464144	0.293	0.059	1.08E-23	0 M1 _	Fhl1
Penk	3.98E-28	0.477748	0.605	0.224	8.01E-24	0 M1 _	Penk
Pde10a	3.78E-28	0.555115	0.443	0.132	7.61E-24	0 M1 _	Pde10a
Adam12	3.57E-28	0.275691	0.257	0.046	7.19E-24	0 M1 _	Adam12
Evc	3.38E-28	0.40928	0.425	0.118	6.80E-24	0 M1 _	Evc
Mme	2.89E-28	0.593096	0.443	0.136	5.83E-24	0 M1 _	Mme
Gpx3	2.54E-28	1.096251	0.76	0.364	5.11E-24	0 M1 _	Gpx3
Entpd2	1.88E-28	0.779327	0.934	0.517	3.80E-24	0 M1 _	Entpd2
Add3	1.61E-28	0.778424	0.844	0.483	3.24E-24	0 M1 _	Add3
Nedd4	1.42E-28	0.627193	0.988	0.882	2.86E-24	0 M1 _	Nedd4
Cd248	1.37E-28	0.715653	0.808	0.396	2.77E-24	0 M1 _	Cd248
Fbn1	1.32E-28	0.709245	0.97	0.596	2.66E-24	0 M1 _	Fbn1
Robo1	1.31E-28	0.51059	0.575	0.204	2.65E-24	0 M1 _	Robo1
Plekha6	8.07E-29	0.729359	0.731	0.336	1.62E-24	0 M1 _	Plekha6
Adam9	5.78E-29	0.315521	0.461	0.128	1.16E-24	0 M1 _	Adam9
Atf5	3.79E-29	0.927442	0.856	0.468	7.63E-25	0 M1 _	Atf5
Cp	2.11E-29	0.872611	0.922	0.578	4.24E-25	0 M1 _	Cp
Ccdc50	1.44E-29	0.803453	0.88	0.556	2.91E-25	0 M1 _	Ccdc50
Scn7a	1.38E-29	0.639288	0.934	0.487	2.78E-25	0 M1 _	Scn7a
Gstt1	1.15E-29	0.769922	0.778	0.375	2.32E-25	0 M1 _	Gstt1
Prg4	1.15E-29	0.541306	0.228	0.034	2.31E-25	0 M1 _	Prg4
Sned1	9.40E-30	1.037595	0.707	0.328	1.89E-25	0 M1 _	Sned1

Vit	9.36E-30	0.464826	0.132	0.01	1.89E-25	0 M1 _	Vit
Synm	8.67E-30	0.44159	0.407	0.107	1.75E-25	0 M1 _	Synm
Csrp2	6.22E-30	1.019866	0.659	0.263	1.25E-25	0 M1 _	Csrp2
Klf9	5.87E-30	0.874899	0.97	0.763	1.18E-25	0 M1 _	Klf9
Adm	3.70E-30	0.497645	0.353	0.081	7.46E-26	0 M1 _	Adm
Gpr153	3.19E-30	0.673641	0.772	0.355	6.42E-26	0 M1 _	Gpr153
Igfbp5	3.07E-30	1.460679	0.856	0.492	6.19E-26	0 M1 _	Igfbp5
Oaf	1.84E-30	0.81903	0.88	0.475	3.71E-26	0 M1 _	Oaf
Col6a3	1.26E-30	0.833353	0.982	0.568	2.54E-26	0 M1 _	Col6a3
Nsg1	1.12E-30	0.446901	0.455	0.122	2.26E-26	0 M1 _	Nsg1
Adamts2	1.03E-30	0.723868	0.689	0.28	2.08E-26	0 M1 _	Adamts2
Adamts12	7.28E-31	0.960935	0.856	0.46	1.47E-26	0 M1 _	Adamts12
Calml4	7.11E-31	0.450338	0.347	0.077	1.43E-26	0 M1 _	Calml4
Sdc4	6.76E-31	0.648909	0.784	0.333	1.36E-26	0 M1 _	Sdc4
Lars2	6.22E-31	1.046437	0.976	0.802	1.25E-26	0 M1 _	Lars2
Ctsb	3.09E-31	0.712529	0.982	0.759	6.22E-27	0 M1 _	Ctsb
Tpst1	2.81E-31	0.643616	0.707	0.294	5.67E-27	0 M1 _	Tpst1
Sox9	2.60E-31	0.381331	0.257	0.042	5.23E-27	0 M1 _	Sox9
Scd2	1.81E-31	0.842255	0.88	0.494	3.65E-27	0 M1 _	Scd2
2900097C17Rik	1.65E-31	0.712966	0.904	0.529	3.33E-27	0 M1 _	2900097C17Rik
Selenop	1.03E-31	0.840676	0.97	0.75	2.08E-27	0 M1 _	Selenop
Man2a1	3.24E-32	0.961973	0.898	0.537	6.52E-28	0 M1 _	Man2a1
Tpbgl	2.14E-32	0.39458	0.353	0.074	4.31E-28	0 M1 _	Tpbgl
Htra1	2.00E-32	1.014743	0.934	0.632	4.04E-28	0 M1 _	Htra1
Loxl2	1.97E-32	0.764064	0.707	0.302	3.96E-28	0 M1 _	Loxl2
Steap3	1.27E-32	0.916319	0.737	0.335	2.57E-28	0 M1 _	Steap3
Osr1	1.20E-32	0.658553	0.533	0.167	2.42E-28	0 M1 _	Osr1
Antxr1	1.09E-32	0.66661	0.701	0.277	2.19E-28	0 M1 _	Antxr1
Slc7a8	8.64E-33	0.380315	0.311	0.059	1.74E-28	0 M1 _	Slc7a8
Creb3l1	7.49E-33	0.749571	0.749	0.325	1.51E-28	0 M1 _	Creb3l1
Calu	7.16E-33	0.805362	0.946	0.609	1.44E-28	0 M1 _	Calu
Fndc3b	5.90E-33	0.860676	0.874	0.508	1.19E-28	0 M1 _	Fndc3b
Neo1	2.56E-33	0.609846	0.635	0.228	5.15E-29	0 M1 _	Neo1
Cebpd	2.51E-33	1.181512	0.85	0.491	5.06E-29	0 M1 _	Cebpd
Ar	2.20E-33	0.904535	0.922	0.548	4.44E-29	0 M1 _	Ar
Tnfaip6	2.19E-33	0.540259	0.467	0.128	4.42E-29	0 M1 _	Tnfaip6
Pkd2	1.77E-33	0.809372	0.904	0.493	3.56E-29	0 M1 _	Pkd2
Egfr	1.68E-33	0.741999	0.701	0.284	3.38E-29	0 M1 _	Egfr
Cd302	6.08E-34	0.98267	0.946	0.599	1.22E-29	0 M1 _	Cd302
Il17d	2.70E-34	0.443295	0.419	0.098	5.44E-30	0 M1 _	Il17d
Astn2	2.67E-34	0.403292	0.377	0.082	5.38E-30	0 M1 _	Astn2

Ndn	2.22E-34	0.752395	0.892	0.438	4.47E-30	0 M1 _	Ndn
S1pr3	1.18E-34	0.68103	0.689	0.261	2.37E-30	0 M1 _	S1pr3
Tril	9.91E-35	0.95269	0.82	0.425	2.00E-30	0 M1 _	Tril
Eda	7.46E-35	0.48937	0.479	0.127	1.50E-30	0 M1 _	Eda
Lpar1	5.23E-35	0.825444	0.814	0.386	1.05E-30	0 M1 _	Lpar1
Ltbp1	2.77E-35	0.955688	0.82	0.385	5.57E-31	0 M1 _	Ltbp1
Clcn5	1.86E-35	0.654222	0.623	0.218	3.74E-31	0 M1 _	Clcn5
Col6a5	7.63E-36	0.518182	0.281	0.044	1.54E-31	0 M1 _	Col6a5
Axl	6.19E-36	0.905567	0.928	0.553	1.25E-31	0 M1 _	Axl
Cd55	5.68E-36	0.536839	0.419	0.098	1.14E-31	0 M1 _	Cd55
Plau	4.41E-36	0.787867	0.743	0.295	8.88E-32	0 M1 _	Plau
Igsf3	4.35E-36	0.663078	0.599	0.206	8.75E-32	0 M1 _	Igsf3
Rhoj	2.37E-36	0.941042	0.97	0.725	4.77E-32	0 M1 _	Rhoj
Epb41l3	1.58E-36	0.549666	0.521	0.143	3.19E-32	0 M1 _	Epb41l3
Fbln5	1.25E-36	0.769356	0.814	0.358	2.52E-32	0 M1 _	Fbln5
Adamts15	9.46E-37	0.752956	0.641	0.217	1.90E-32	0 M1 _	Adamts15
Fam102b	8.33E-37	0.802468	0.713	0.286	1.68E-32	0 M1 _	Fam102b
C1ra	4.72E-37	0.889062	0.946	0.522	9.50E-33	0 M1 _	C1ra
Il33	4.12E-37	1.131653	0.826	0.414	8.30E-33	0 M1 _	Il33
Celf2	3.87E-37	0.950837	0.916	0.552	7.80E-33	0 M1 _	Celf2
Rhob	3.84E-37	1.060755	0.934	0.593	7.73E-33	0 M1 _	Rhob
Nid2	2.84E-37	0.945916	0.754	0.337	5.72E-33	0 M1 _	Nid2
Il11ra1	2.66E-37	0.996814	0.97	0.586	5.35E-33	0 M1 _	Il11ra1
Tgfbr3	2.39E-37	0.782486	0.862	0.432	4.81E-33	0 M1 _	Tgfbr3
Tsc22d3	8.73E-38	0.942228	0.886	0.448	1.76E-33	0 M1 _	Tsc22d3
Nrxn2	6.63E-38	0.98284	0.677	0.251	1.34E-33	0 M1 _	Nrxn2
Islr	5.68E-38	1.005321	0.886	0.441	1.14E-33	0 M1 _	Islr
Fndc5	4.26E-38	0.493571	0.389	0.08	8.59E-34	0 M1 _	Fndc5
Inmt	3.93E-38	1.051001	0.743	0.271	7.91E-34	0 M1 _	Inmt
Xpnpep2	3.52E-38	0.449708	0.311	0.051	7.09E-34	0 M1 _	Xpnpep2
Lpar4	1.57E-38	0.759976	0.527	0.151	3.15E-34	0 M1 _	Lpar4
Lsamp	7.81E-39	1.022105	0.892	0.474	1.57E-34	0 M1 _	Lsamp
Zcchc24	2.43E-39	0.908213	0.838	0.397	4.90E-35	0 M1 _	Zcchc24
Ddr2	1.54E-39	0.884815	0.85	0.413	3.09E-35	0 M1 _	Ddr2
Igsf10	1.44E-39	0.940663	0.91	0.452	2.89E-35	0 M1 _	Igsf10
Plac8	1.32E-39	0.887952	0.826	0.331	2.66E-35	0 M1 _	Plac8
Fmo1	1.19E-39	1.072256	0.868	0.463	2.41E-35	0 M1 _	Fmo1
Ppp1r3c	5.71E-40	0.322824	0.305	0.045	1.15E-35	0 M1 _	Ppp1r3c
Cmklr1	5.30E-40	0.754403	0.731	0.272	1.07E-35	0 M1 _	Cmklr1
Adam23	4.38E-40	0.373955	0.317	0.049	8.82E-36	0 M1 _	Adam23
Cyp1b1	1.17E-40	1.061242	0.754	0.312	2.36E-36	0 M1 _	Cyp1b1

Hspg2	1.11E-40	1.106963	0.976	0.678	2.24E-36	0 M1 _	Hspg2
Rassf2	7.21E-41	0.727416	0.593	0.183	1.45E-36	0 M1 _	Rassf2
Col5a3	4.67E-41	1.179126	0.946	0.536	9.41E-37	0 M1 _	Col5a3
Plpp3	3.31E-41	0.967307	0.994	0.711	6.66E-37	0 M1 _	Plpp3
B4galt1	2.60E-41	0.759026	0.808	0.331	5.24E-37	0 M1 _	B4galt1
AU019823	2.00E-41	0.288899	0.246	0.026	4.02E-37	0 M1 _	AU019823
Ddit4	1.02E-41	0.721458	0.527	0.133	2.05E-37	0 M1 _	Ddit4
4632415L05Rik	9.34E-42	0.255607	0.222	0.02	1.88E-37	0 M1 _	4632415L05Rik
Col5a1	5.26E-42	0.972596	0.94	0.483	1.06E-37	0 M1 _	Col5a1
Dopey2	4.02E-42	0.291747	0.18	0.012	8.10E-38	0 M1 _	Dopey2
Lrig1	3.48E-42	0.614114	0.605	0.179	7.01E-38	0 M1 _	Lrig1
Dnm1	2.58E-42	0.639364	0.629	0.19	5.19E-38	0 M1 _	Dnm1
Lama2	2.35E-42	1.08589	0.982	0.599	4.73E-38	0 M1 _	Lama2
Lamc1	1.79E-42	1.143563	0.928	0.59	3.60E-38	0 M1 _	Lamc1
Htra3	1.33E-42	1.216033	0.964	0.538	2.67E-38	0 M1 _	Htra3
Fetub	1.31E-42	0.315828	0.192	0.014	2.63E-38	0 M1 _	Fetub
Fam208b	9.10E-43	0.279117	0.234	0.023	1.83E-38	0 M1 _	Fam208b
Zbtb16	8.33E-43	0.461637	0.413	0.076	1.68E-38	0 M1 _	Zbtb16
Scd1	5.73E-43	1.195369	0.731	0.287	1.15E-38	0 M1 _	Scd1
Pdgfra	5.72E-43	1.100675	0.982	0.652	1.15E-38	0 M1 _	Pdgfra
Taf6l	4.44E-43	0.405308	0.389	0.069	8.95E-39	0 M1 _	Taf6l
Fgfr1	3.08E-43	0.926459	0.946	0.456	6.19E-39	0 M1 _	Fgfr1
Sphkap	2.18E-43	0.409935	0.293	0.038	4.39E-39	0 M1 _	Sphkap
P2ry1	1.83E-43	0.945033	0.695	0.235	3.69E-39	0 M1 _	P2ry1
Cyr61	1.39E-43	0.627245	0.234	0.022	2.80E-39	0 M1 _	Cyr61
Igfbp4	1.02E-43	1.153269	0.994	0.715	2.05E-39	0 M1 _	Igfbp4
Igf1	9.10E-44	1.454797	0.91	0.479	1.83E-39	0 M1 _	Igf1
Lamb1	5.15E-44	1.120808	0.958	0.635	1.04E-39	0 M1 _	Lamb1
Mmp14	4.60E-44	1.117548	0.946	0.51	9.26E-40	0 M1 _	Mmp14
Fam49a	2.25E-44	0.587022	0.611	0.165	4.54E-40	0 M1 _	Fam49a
Aldh2	2.06E-44	0.964148	0.982	0.741	4.14E-40	0 M1 _	Aldh2
Atp1a2	5.42E-45	0.811577	0.671	0.213	1.09E-40	0 M1 _	Atp1a2
Zfp395	3.31E-45	0.459225	0.377	0.064	6.67E-41	0 M1 _	Zfp395
Chtf8	2.96E-45	0.412298	0.401	0.069	5.97E-41	0 M1 _	Chtf8
Plxdc2	2.68E-45	1.158985	0.982	0.557	5.39E-41	0 M1 _	Plxdc2
Hsp90b1	1.97E-45	0.90123	0.994	0.886	3.96E-41	0 M1 _	Hsp90b1
Col14a1	1.93E-45	1.240033	0.88	0.428	3.88E-41	0 M1 _	Col14a1
Medag	1.75E-45	1.080347	0.778	0.299	3.53E-41	0 M1 _	Medag
Kcna2	4.59E-46	0.470752	0.287	0.034	9.25E-42	0 M1 _	Kcna2
1500011K16Rik	3.60E-46	0.349784	0.287	0.032	7.25E-42	0 M1 _	1500011K16Rik
Emilin2	2.14E-46	0.713539	0.563	0.145	4.32E-42	0 M1 _	Emilin2

Ifi207	2.05E-46	1.136341	0.778	0.302	4.13E-42	0 M1 _	Ifi207
Itih5	1.58E-46	1.3831	0.982	0.655	3.19E-42	0 M1 _	Itih5
Fam96b	9.22E-47	0.364629	0.359	0.05	1.86E-42	0 M1 _	Fam96b
0610009O20Rik	6.26E-47	0.347035	0.246	0.023	1.26E-42	0 M1 _	0610009O20Rik
Lrp1	5.86E-47	1.120168	0.976	0.678	1.18E-42	0 M1 _	Lrp1
Ret	5.34E-47	0.282573	0.21	0.015	1.07E-42	0 M1 _	Ret
Ptges	5.04E-47	0.783178	0.719	0.23	1.01E-42	0 M1 _	Ptges
Pyurf	3.67E-47	0.294464	0.281	0.03	7.39E-43	0 M1 _	Pyurf
Camk2n1	3.49E-47	1.177396	0.922	0.516	7.02E-43	0 M1 _	Camk2n1
Nid1	1.75E-47	1.145733	0.982	0.716	3.53E-43	0 M1 _	Nid1
Masp1	9.50E-48	0.642787	0.269	0.029	1.91E-43	0 M1 _	Masp1
Scara3	5.88E-48	0.866266	0.671	0.204	1.18E-43	0 M1 _	Scara3
Nfix	3.93E-48	1.092298	0.976	0.668	7.92E-44	0 M1 _	Nfix
Crispld2	3.13E-48	1.390828	0.946	0.56	6.31E-44	0 M1 _	Crispld2
C1s1	2.31E-48	1.137403	0.982	0.702	4.66E-44	0 M1 _	C1s1
Sh3pxd2b	1.62E-48	0.84527	0.784	0.293	3.27E-44	0 M1 _	Sh3pxd2b
Frmd6	9.91E-49	0.998146	0.886	0.354	2.00E-44	0 M1 _	Frmd6
Tspan11	7.50E-49	0.986915	0.868	0.353	1.51E-44	0 M1 _	Tspan11
Mrc2	6.98E-49	0.949865	0.892	0.39	1.41E-44	0 M1 _	Mrc2
Ms4a4d	4.36E-49	1.190745	0.892	0.415	8.78E-45	0 M1 _	Ms4a4d
Cd34	3.17E-49	1.42013	0.892	0.415	6.39E-45	0 M1 _	Cd34
Dapk1	1.84E-49	1.008476	0.826	0.33	3.71E-45	0 M1 _	Dapk1
Fam212a	1.81E-49	0.285018	0.24	0.019	3.64E-45	0 M1 _	Fam212a
Lrrtm3	7.65E-50	0.323548	0.198	0.012	1.54E-45	0 M1 _	Lrrtm3
C3	4.30E-50	2.083386	0.85	0.365	8.65E-46	0 M1 _	C3
Thsd7a	3.88E-50	0.623905	0.419	0.071	7.82E-46	0 M1 _	Thsd7a
Sssca1	3.41E-50	0.289439	0.24	0.019	6.86E-46	0 M1 _	Sssca1
Zbtb20	2.51E-50	1.181689	0.988	0.893	5.05E-46	0 M1 _	Zbtb20
Rspo1	1.25E-50	0.71843	0.413	0.071	2.52E-46	0 M1 _	Rspo1
Nfia	9.71E-51	1.197289	0.928	0.534	1.96E-46	0 M1 _	Nfia
Gxylt2	6.59E-51	0.80029	0.581	0.146	1.33E-46	0 M1 _	Gxylt2
Lum	1.49E-51	1.332408	0.982	0.58	3.00E-47	0 M1 _	Lum
Anpep	1.27E-51	0.96266	0.725	0.238	2.56E-47	0 M1 _	Anpep
Ppl	8.82E-53	0.406916	0.269	0.024	1.78E-48	0 M1 _	Ppl
Ces2g	8.77E-53	0.836855	0.521	0.112	1.77E-48	0 M1 _	Ces2g
Adam19	5.02E-53	1.142004	0.725	0.242	1.01E-48	0 M1 _	Adam19
Maf	4.76E-53	1.44239	0.922	0.505	9.58E-49	0 M1 _	Maf
Pcsk6	2.33E-53	0.502099	0.347	0.043	4.68E-49	0 M1 _	Pcsk6
Gpm6b	1.35E-53	1.292049	0.952	0.491	2.71E-49	0 M1 _	Gpm6b
Gcn1l1	1.34E-53	0.339542	0.293	0.028	2.69E-49	0 M1 _	Gcn1l1
Zufsp	5.34E-54	0.347412	0.257	0.02	1.08E-49	0 M1 _	Zufsp

Abca8b	3.39E-54	1.0542	0.629	0.165	6.83E-50	0 M1 _	Abca8b
Lrrc17	7.35E-55	0.647891	0.365	0.047	1.48E-50	0 M1 _	Lrrc17
Col15a1	4.99E-55	1.493697	0.97	0.609	1.00E-50	0 M1 _	Col15a1
mt-Atp8	1.19E-55	1.301682	0.946	0.581	2.40E-51	0 M1 _	mt-Atp8
Fam58b	1.17E-55	0.326755	0.275	0.023	2.36E-51	0 M1 _	Fam58b
Crabp1	6.28E-56	1.829339	0.623	0.164	1.26E-51	0 M1 _	Crabp1
Mgst1	1.89E-56	1.226461	0.934	0.469	3.81E-52	0 M1 _	Mgst1
Rbms3	5.13E-57	1.394069	0.964	0.543	1.03E-52	0 M1 _	Rbms3
Timp2	3.99E-57	1.240202	0.988	0.685	8.04E-53	0 M1 _	Timp2
Clmp	1.40E-57	0.98522	0.79	0.267	2.81E-53	0 M1 _	Clmp
Clec3b	1.34E-57	1.56311	0.916	0.401	2.70E-53	0 M1 _	Clec3b
Efemp1	6.53E-58	1.527102	0.838	0.32	1.31E-53	0 M1 _	Efemp1
Abca8a	1.25E-58	1.559474	0.958	0.498	2.52E-54	0 M1 _	Abca8a
Papd7	2.07E-59	0.292941	0.222	0.012	4.18E-55	0 M1 _	Papd7
D3Ertd254e	1.86E-59	0.258995	0.204	0.009	3.74E-55	0 M1 _	D3Ertd254e
Gsta3	1.37E-59	0.583267	0.347	0.039	2.76E-55	0 M1 _	Gsta3
D10Jhu81e	1.80E-60	0.353479	0.311	0.027	3.63E-56	0 M1 _	D10Jhu81e
Lppos	1.63E-60	0.265254	0.216	0.01	3.28E-56	0 M1 _	Lppos
Rspo3	9.26E-61	1.283672	0.766	0.237	1.87E-56	0 M1 _	Rspo3
Aox1	7.82E-61	0.6219	0.497	0.084	1.58E-56	0 M1 _	Aox1
Tnxb	3.89E-61	1.317981	0.725	0.204	7.84E-57	0 M1 _	Tnxb
Ikbkap	4.49E-62	0.267338	0.204	0.008	9.03E-58	0 M1 _	Ikbkap
A230050P20Rik	1.07E-62	0.314755	0.246	0.014	2.16E-58	0 M1 _	A230050P20Rik
Mest	5.83E-64	0.570149	0.299	0.024	1.17E-59	0 M1 _	Mest
Bmp4	2.25E-64	1.314265	0.731	0.207	4.53E-60	0 M1 _	Bmp4
Tmem57	5.11E-65	0.347911	0.305	0.024	1.03E-60	0 M1 _	Tmem57
Scara5	4.82E-65	1.497965	0.916	0.371	9.71E-61	0 M1 _	Scara5
Adh1	3.30E-65	1.037855	0.641	0.135	6.66E-61	0 M1 _	Adh1
Mmp2	2.72E-65	1.715286	0.982	0.531	5.48E-61	0 M1 _	Mmp2
9030624J02Rik	1.16E-65	0.343812	0.317	0.025	2.33E-61	0 M1 _	9030624J02Rik
Gas1	1.10E-65	1.789474	0.976	0.592	2.21E-61	0 M1 _	Gas1
2410089E03Rik	9.75E-66	0.301423	0.287	0.019	1.96E-61	0 M1 _	2410089E03Rik
Mum1	2.51E-66	0.460373	0.365	0.036	5.06E-62	0 M1 _	Mum1
Coro1b	1.90E-66	0.495047	0.503	0.074	3.83E-62	0 M1 _	Coro1b
Fam96a	8.86E-67	0.364455	0.317	0.025	1.78E-62	0 M1 _	Fam96a
Rbm12	1.40E-67	0.2836	0.24	0.011	2.81E-63	0 M1 _	Rbm12
Fam26e	9.59E-68	0.318151	0.234	0.01	1.93E-63	0 M1 _	Fam26e
Ptgfr	7.07E-68	0.671536	0.359	0.035	1.42E-63	0 M1 _	Ptgfr
Spock2	2.26E-68	0.958393	0.665	0.144	4.54E-64	0 M1 _	Spock2
Capn6	2.07E-68	0.876311	0.593	0.116	4.17E-64	0 M1 _	Capn6
2010111I01Rik	5.01E-69	0.448359	0.449	0.052	1.01E-64	0 M1 _	2010111I01Rik

Ccl11	6.15E-70	1.677639	0.946	0.402	1.24E-65	0 M1 _	Ccl11
Antxr2	1.46E-70	1.49731	0.892	0.358	2.94E-66	0 M1 _	Antxr2
Dcn	3.25E-71	1.614986	0.994	0.652	6.55E-67	0 M1 _	Dcn
D17Wsu92e	3.08E-71	0.41894	0.389	0.037	6.19E-67	0 M1 _	D17Wsu92e
Pla2g16	2.84E-71	0.419435	0.323	0.024	5.71E-67	0 M1 _	Pla2g16
2310035C23Rik	2.53E-71	0.338901	0.299	0.019	5.09E-67	0 M1 _	2310035C23Rik
Fam69b	2.23E-71	0.30907	0.222	0.007	4.49E-67	0 M1 _	Fam69b
Angptl1	8.22E-72	1.055621	0.281	0.017	1.66E-67	0 M1 _	Angptl1
Srpx	4.25E-72	1.810232	0.94	0.438	8.55E-68	0 M1 _	Srpx
BC017643	3.09E-72	0.367075	0.263	0.013	6.23E-68	0 M1 _	BC017643
Rnase4	2.04E-72	1.632341	0.97	0.574	4.11E-68	0 M1 _	Rnase4
Spon2	5.97E-73	1.673353	0.743	0.179	1.20E-68	0 M1 _	Spon2
Col18a1	2.00E-73	1.285684	0.754	0.187	4.03E-69	0 M1 _	Col18a1
Tmem55b	3.01E-74	0.423016	0.353	0.028	6.06E-70	0 M1 _	Tmem55b
Fst	9.71E-75	2.120007	0.826	0.259	1.96E-70	0 M1 _	Fst
Papd4	3.37E-75	0.362856	0.323	0.022	6.79E-71	0 M1 _	Papd4
Ifi205	7.01E-77	1.821281	0.838	0.257	1.41E-72	0 M1 _	Ifi205
Gsn	3.59E-77	2.243742	0.994	0.788	7.24E-73	0 M1 _	Gsn
Dpep1	1.39E-77	2.009672	0.976	0.47	2.79E-73	0 M1 _	Dpep1
Fam208a	3.73E-79	0.682268	0.491	0.056	7.51E-75	0 M1 _	Fam208a
Mmp19	1.30E-79	1.381187	0.868	0.266	2.62E-75	0 M1 _	Mmp19
Ntrk2	5.76E-80	1.582131	0.814	0.226	1.16E-75	0 M1 _	Ntrk2
Fam103a1	1.71E-81	0.581345	0.431	0.04	3.44E-77	0 M1 _	Fam103a1
1190002N15Rik	9.65E-82	0.345144	0.269	0.011	1.94E-77	0 M1 _	1190002N15Rik
Dirc2	3.80E-82	0.447247	0.395	0.031	7.65E-78	0 M1 _	Dirc2
Gatsl2	2.74E-82	0.378024	0.293	0.014	5.51E-78	0 M1 _	Gatsl2
0610037L13Rik	1.70E-82	0.481	0.383	0.029	3.43E-78	0 M1 _	0610037L13Rik
Fbxo18	1.06E-82	0.415286	0.371	0.027	2.14E-78	0 M1 _	Fbxo18
Sdccag3	5.75E-83	0.452865	0.359	0.024	1.16E-78	0 M1 _	Sdccag3
Fam220a	7.39E-84	0.461644	0.407	0.033	1.49E-79	0 M1 _	Fam220a
Wisp1	9.98E-85	0.341919	0.192	0.001	2.01E-80	0 M1 _	Wisp1
1110008F13Rik	6.14E-85	0.594663	0.545	0.062	1.24E-80	0 M1 _	1110008F13Rik
Kdelc1	7.16E-86	0.35777	0.287	0.012	1.44E-81	0 M1 _	Kdelc1
Enpp2	2.76E-88	2.601172	0.958	0.43	5.57E-84	0 M1 _	Enpp2
Pappa	1.73E-89	1.72344	0.778	0.177	3.48E-85	0 M1 _	Pappa
Skiv2l2	1.15E-89	0.546309	0.425	0.033	2.31E-85	0 M1 _	Skiv2l2
Ptp4a1	7.70E-91	0.661302	0.467	0.043	1.55E-86	0 M1 _	Ptp4a1
Myoc	1.10E-91	1.028965	0.335	0.017	2.21E-87	0 M1 _	Myoc
Aox3	1.88E-92	1.479643	0.599	0.084	3.78E-88	0 M1 _	Aox3
Fgf10	1.00E-93	2.022837	0.85	0.229	2.02E-89	0 M1 _	Fgf10
Fam19a5	2.93E-94	0.37864	0.228	0.003	5.91E-90	0 M1 _	Fam19a5

CT025619.1	7.05E-98	0.446878	0.311	0.011	1.42E-93	0 M1 _	CT025619.1
Zcchc11	8.57E-99	0.681321	0.491	0.042	1.73E-94	0 M1 _	Zcchc11
Dopey1	2.70E-100	0.525355	0.419	0.027	5.44E-96	0 M1 _	Dopey1
Tmem110	1.39E-105	0.448563	0.353	0.014	2.80E-101	0 M1 _	Tmem110
Mut	1.83E-107	0.516489	0.413	0.023	3.70E-103	0 M1 _	Mut
Zcchc6	1.63E-109	0.85791	0.611	0.061	3.28E-105	0 M1 _	Zcchc6
1700020I14Rik	8.79E-113	0.797352	0.653	0.067	1.77E-108	0 M1 _	1700020I14Rik
2810474O19Rik	6.23E-115	0.787035	0.551	0.044	1.25E-110	0 M1 _	2810474O19Rik
Mgea5	2.84E-115	0.587365	0.461	0.027	5.72E-111	0 M1 _	Mgea5
BC003331	3.20E-119	0.705796	0.509	0.035	6.44E-115	0 M1 _	BC003331
Tmem5	1.36E-125	0.762448	0.605	0.049	2.74E-121	0 M1 _	Tmem5
1810022K09Rik	1.03E-125	0.872009	0.659	0.062	2.08E-121	0 M1 _	1810022K09Rik
Trove2	3.26E-129	0.844335	0.599	0.046	6.56E-125	0 M1 _	Trove2
Ctgf	3.07E-134	1.800601	0.449	0.019	6.19E-130	0 M1 _	Ctgf
Fam69a	1.79E-134	0.701563	0.527	0.031	3.61E-130	0 M1 _	Fam69a
2410015M20Rik	6.22E-142	0.977578	0.743	0.071	1.25E-137	0 M1 _	2410015M20Rik
Usmg5	2.43E-142	1.066346	0.808	0.082	4.89E-138	0 M1 _	Usmg5
Aaed1	1.63E-145	0.80122	0.581	0.036	3.29E-141	0 M1 _	Aaed1
Dpt	1.26E-145	2.342407	0.85	0.118	2.53E-141	0 M1 _	Dpt
AI314180	5.96E-146	0.794313	0.581	0.035	1.20E-141	0 M1 _	AI314180
2010107E04Rik	3.77E-162	1.184718	0.832	0.077	7.59E-158	0 M1 _	2010107E04Rik
Atp5o.1	9.90E-166	1.123091	0.82	0.072	1.99E-161	0 M1 _	Atp5o.1
Minos1	3.62E-167	1.269847	0.832	0.075	7.29E-163	0 M1 _	Minos1
Kdelc2	1.04E-181	0.95725	0.641	0.031	2.09E-177	0 M1 _	Kdelc2
Aes	1.99E-185	1.491024	0.904	0.079	4.02E-181	0 M1 _	Aes
Fam46a	4.20E-230	1.386155	0.617	0.014	8.45E-226	0 M1 _	Fam46a
Fam198b	0	2.324246	0.94	0.034	0	0 M1 _	Fam198b
Anp32e	2.48E-06	0.262143	0.43	0.313	0.049851	1 L1 _	Anp32e
Uqcrc1	2.33E-06	0.258574	0.602	0.483	0.046835	1 L1 _	Uqcrc1
Idnk	2.21E-06	0.269327	0.508	0.387	0.044421	1 L1 _	Idnk
Cycs1	2.02E-06	0.251392	0.621	0.518	0.040619	1 L1 _	Cycs
Jak1	1.82E-06	0.287048	0.793	0.765	0.036557	1 L1 _	Jak1
Kif13b	1.72E-06	0.275396	0.501	0.391	0.034666	1 L1 _	Kif13b
Rbm25	1.67E-06	0.269886	0.726	0.679	0.033584	1 L1 _	Rbm25
Ccdc12	1.60E-06	0.321056	0.694	0.617	0.032243	1 L1 _	Ccdc12
Pgs1	1.57E-06	0.259785	0.248	0.152	0.031627	1 L1 _	Pgs1
Lst1	1.52E-06	0.779046	0.248	0.162	0.030713	1 L1 _	Lst1
Eif4a1	7.99E-07	0.31805	0.786	0.764	0.016084	1 L1 _	Eif4a1
Wipf11	6.68E-07	0.27241	0.552	0.431	0.013456	1 L1 _	Wipf1
Hist1h4d	6.44E-07	0.287601	0.529	0.407	0.012975	1 L1 _	Hist1h4d
Snrpd2	5.80E-07	0.265798	0.759	0.679	0.011679	1 L1 _	Snrpd2

Srpk1	5.76E-07	0.261274	0.476	0.358	0.011604	1 L1 _	Srpk1
Nfkb1	3.43E-07	0.253469	0.517	0.402	0.006898	1 L1 _	Nfkb1
Ifnar1	3.28E-07	0.269214	0.423	0.297	0.006608	1 L1 _	Ifnar1
Rgs10	2.64E-07	0.495063	0.322	0.224	0.005313	1 L1 _	Rgs10
Ggnbp2	2.50E-07	0.287439	0.589	0.459	0.005033	1 L1 _	Ggnbp2
Phb2	2.27E-07	0.270872	0.644	0.53	0.004567	1 L1 _	Phb2
Pitpna	2.15E-07	0.323533	0.609	0.485	0.004328	1 L1 _	Pitpna
Os9	9.31E-08	0.308398	0.648	0.523	0.001875	1 L1 _	Os9
Uqcc2	9.17E-08	0.318413	0.683	0.572	0.001847	1 L1 _	Uqcc2
Gas71	8.46E-08	0.320139	0.462	0.332	0.001703	1 L1 _	Gas7
Man2b1	7.40E-08	0.457365	0.444	0.328	0.00149	1 L1 _	Man2b1
Dennd1b	7.04E-08	0.252121	0.343	0.217	0.001419	1 L1 _	Dennd1b
Mrpl58	6.72E-08	0.257375	0.517	0.378	0.001353	1 L1 _	Mrpl58
Hpcal11	6.55E-08	0.286881	0.478	0.341	0.00132	1 L1 _	Hpcal1
Fam173a	6.49E-08	0.298468	0.556	0.43	0.001307	1 L1 _	Fam173a
Atp5o	6.03E-08	0.254228	0.761	0.587	0.001214	1 L1 _	Atp5o
Grb2	5.07E-08	0.303337	0.51	0.385	0.001022	1 L1 _	Grb2
Cnot6l	4.81E-08	0.274904	0.492	0.363	0.000969	1 L1 _	Cnot6l
Sema6d1	4.80E-08	0.258154	0.48	0.331	0.000966	1 L1 _	Sema6d
Ldlrad4	4.15E-08	0.273771	0.278	0.163	0.000836	1 L1 _	Ldlrad4
Srsf51	4.03E-08	0.296609	0.807	0.752	0.000811	1 L1 _	Srsf5
Crot	4.00E-08	0.258593	0.407	0.278	0.000806	1 L1 _	Crot
Tle2	3.99E-08	0.250019	0.179	0.089	0.000803	1 L1 _	Tle2
Actr2	3.95E-08	0.276889	0.641	0.505	0.000795	1 L1 _	Actr2
Akr1b3	3.54E-08	0.285558	0.552	0.422	0.000712	1 L1 _	Akr1b3
Klf61	3.04E-08	0.320938	0.662	0.536	0.000612	1 L1 _	Klf6
Trp53	3.00E-08	0.291972	0.529	0.398	0.000604	1 L1 _	Trp53
Pole4	2.95E-08	0.254609	0.421	0.29	0.000595	1 L1 _	Pole4
Borcs8	2.84E-08	0.291234	0.322	0.201	0.000571	1 L1 _	Borcs8
Izumo4	2.63E-08	0.274054	0.361	0.234	0.000529	1 L1 _	Izumo4
Mark2	1.88E-08	0.315262	0.446	0.304	0.00038	1 L1 _	Mark2
Swap701	1.37E-08	0.340935	0.492	0.359	0.000276	1 L1 _	Swap70
Spcs2	1.04E-08	0.276228	0.818	0.736	0.000209	1 L1 _	Spcs2
Mrpl54	6.94E-09	0.270727	0.533	0.404	0.00014	1 L1 _	Mrpl54
Odc1	6.17E-09	0.302378	0.379	0.245	0.000124	1 L1 _	Odc1
Ndufb7	6.16E-09	0.269224	0.768	0.692	0.000124	1 L1 _	Ndufb7
Sin3b	5.28E-09	0.287521	0.579	0.441	0.000106	1 L1 _	Sin3b
Cdc37	5.09E-09	0.271782	0.754	0.67	0.000103	1 L1 _	Cdc37
Gm10076	4.79E-09	0.253129	0.391	0.249	9.64E-05	1 L1 _	Gm10076
Pabpn1	4.66E-09	0.279395	0.687	0.538	9.39E-05	1 L1 _	Pabpn1
1110008P14Rik	4.31E-09	0.310512	0.405	0.27	8.69E-05	1 L1 _	1110008P14Rik

Tln11	3.89E-09	0.274693	0.837	0.81	7.84E-05	1 L1 _	Tln1
Ran	3.24E-09	0.363852	0.775	0.682	6.53E-05	1 L1 _	Ran
H2-Eb1	3.20E-09	1.747341	0.313	0.2	6.44E-05	1 L1 _	H2-Eb1
Arl6ip5	2.97E-09	0.335864	0.625	0.481	5.98E-05	1 L1 _	Arl6ip5
Med10	2.69E-09	0.262206	0.501	0.358	5.42E-05	1 L1 _	Med10
Cnot3	2.45E-09	0.29317	0.591	0.446	4.94E-05	1 L1 _	Cnot3
Hspe1	2.17E-09	0.376937	0.791	0.713	4.36E-05	1 L1 _	Hspe1
Cdk2ap2	2.08E-09	0.326948	0.621	0.477	4.18E-05	1 L1 _	Cdk2ap2
Cbl	2.02E-09	0.272052	0.462	0.308	4.07E-05	1 L1 _	Cbl
Tkt	1.46E-09	0.336148	0.57	0.441	2.94E-05	1 L1 _	Tkt
Use1	1.44E-09	0.364781	0.611	0.491	2.90E-05	1 L1 _	Use1
Xrn2	1.28E-09	0.318579	0.602	0.462	2.58E-05	1 L1 _	Xrn2
Mitd1	1.13E-09	0.312253	0.377	0.24	2.27E-05	1 L1 _	Mitd1
Ndfip1	1.10E-09	0.356698	0.825	0.703	2.21E-05	1 L1 _	Ndfip1
Znrf1	8.76E-10	0.266752	0.384	0.235	1.76E-05	1 L1 _	Znrf1
Ccnd3	8.33E-10	0.357524	0.63	0.51	1.68E-05	1 L1 _	Ccnd3
Serbp1	5.93E-10	0.305894	0.906	0.897	1.19E-05	1 L1 _	Serbp1
Rpa2	5.93E-10	0.250805	0.205	0.098	1.19E-05	1 L1 _	Rpa2
Eif5a	4.61E-10	0.34861	0.871	0.841	9.29E-06	1 L1 _	Eif5a
Cetn2	3.64E-10	0.367837	0.559	0.39	7.34E-06	1 L1 _	Cetn2
Ppm1g	3.09E-10	0.267984	0.543	0.389	6.23E-06	1 L1 _	Ppm1g
Cct5	2.86E-10	0.313012	0.713	0.59	5.77E-06	1 L1 _	Cct5
Sat1	2.72E-10	0.542933	0.752	0.679	5.47E-06	1 L1 _	Sat1
Vgll4	2.65E-10	0.304905	0.586	0.443	5.33E-06	1 L1 _	Vgll4
Tcirg1	2.58E-10	0.295293	0.274	0.146	5.21E-06	1 L1 _	Tcirg1
Myo1e	2.55E-10	0.253529	0.432	0.279	5.13E-06	1 L1 _	Myo1e
Nt5c	1.71E-10	0.334423	0.522	0.361	3.44E-06	1 L1 _	Nt5c
Aif1	1.30E-10	0.976766	0.159	0.069	2.62E-06	1 L1 _	Aif1
Cct7	1.25E-10	0.328334	0.692	0.555	2.53E-06	1 L1 _	Cct7
Npc1	1.16E-10	0.252288	0.326	0.187	2.34E-06	1 L1 _	Npc1
Morc3	1.16E-10	0.284964	0.393	0.235	2.33E-06	1 L1 _	Morc3
Rhog	1.14E-10	0.371561	0.469	0.312	2.30E-06	1 L1 _	Rhog
Sec61b	1.13E-10	0.300252	0.851	0.783	2.27E-06	1 L1 _	Sec61b
H2-Aa	1.09E-10	1.730697	0.322	0.196	2.19E-06	1 L1 _	H2-Aa
Mfsd10	9.20E-11	0.281163	0.437	0.28	1.85E-06	1 L1 _	Mfsd10
Mef2a2	8.88E-11	0.294574	0.754	0.64	1.79E-06	1 L1 _	Mef2a
Ptbp31	8.77E-11	0.296919	0.697	0.566	1.77E-06	1 L1 _	Ptbp3
Vps13a	8.64E-11	0.292738	0.425	0.258	1.74E-06	1 L1 _	Vps13a
Mndal1	8.53E-11	0.308049	0.754	0.649	1.72E-06	1 L1 _	Mndal
Mrps23	8.12E-11	0.26649	0.389	0.236	1.64E-06	1 L1 _	Mrps23
Tmem189	7.89E-11	0.257176	0.251	0.125	1.59E-06	1 L1 _	Tmem189

Ncl	7.37E-11	0.350326	0.876	0.843	1.48E-06	1 L1 _	Ncl
Tmem160	7.24E-11	0.373942	0.651	0.513	1.46E-06	1 L1 _	Tmem160
Mcl11	6.64E-11	0.370126	0.738	0.605	1.34E-06	1 L1 _	Mcl1
Cap1	6.25E-11	0.302057	0.478	0.316	1.26E-06	1 L1 _	Cap1
Chd3	6.08E-11	0.350577	0.595	0.423	1.22E-06	1 L1 _	Chd3
Pet100	4.97E-11	0.293334	0.536	0.376	1.00E-06	1 L1 _	Pet100
Lbr	4.45E-11	0.252358	0.34	0.191	8.95E-07	1 L1 _	Lbr
Eif4b	3.91E-11	0.322623	0.598	0.438	7.87E-07	1 L1 _	Eif4b
H2-Ke6	3.86E-11	0.315282	0.359	0.21	7.78E-07	1 L1 _	H2-Ke6
2510002D24Rik	3.68E-11	0.257758	0.271	0.139	7.42E-07	1 L1 _	2510002D24Rik
Pcna	3.35E-11	0.256016	0.425	0.261	6.74E-07	1 L1 _	Pcna
Cirbp	2.81E-11	0.363536	0.595	0.438	5.66E-07	1 L1 _	Cirbp
Itpr2	2.72E-11	0.287668	0.501	0.323	5.47E-07	1 L1 _	Itpr2
Zfas1	2.47E-11	0.282206	0.338	0.19	4.97E-07	1 L1 _	Zfas1
Dnajc9	2.42E-11	0.258015	0.278	0.143	4.88E-07	1 L1 _	Dnajc9
Fkbp31	2.35E-11	0.362056	0.699	0.573	4.73E-07	1 L1 _	Fkbp3
Ptpn2	2.22E-11	0.297324	0.331	0.189	4.48E-07	1 L1 _	Ptpn2
Tpp2	1.77E-11	0.356947	0.52	0.367	3.57E-07	1 L1 _	Tpp2
Tpst21	1.68E-11	0.268931	0.446	0.275	3.37E-07	1 L1 _	Tpst2
Igbp1	1.36E-11	0.332276	0.441	0.279	2.73E-07	1 L1 _	Igbp1
Stk24	1.32E-11	0.341411	0.559	0.388	2.66E-07	1 L1 _	Stk24
1700017B05Rik	1.11E-11	0.348736	0.267	0.132	2.23E-07	1 L1 _	1700017B05Rik
Fbl	1.06E-11	0.350187	0.457	0.301	2.14E-07	1 L1 _	Fbl
Rbl2	1.01E-11	0.328823	0.407	0.255	2.04E-07	1 L1 _	Rbl2
Clta	9.90E-12	0.340412	0.862	0.791	1.99E-07	1 L1 _	Clta
Nans	9.21E-12	0.304076	0.395	0.245	1.85E-07	1 L1 _	Nans
Tap1	6.94E-12	0.306895	0.464	0.291	1.40E-07	1 L1 _	Tap1
Ankrd11	6.89E-12	0.329981	0.807	0.717	1.39E-07	1 L1 _	Ankrd11
Atp5l	6.88E-12	0.282524	0.91	0.897	1.39E-07	1 L1 _	Atp5l
Plgrkt	6.27E-12	0.340381	0.611	0.457	1.26E-07	1 L1 _	Plgrkt
Cx3cr1	5.16E-12	0.704471	0.115	0.037	1.04E-07	1 L1 _	Cx3cr1
Arhgef31	4.84E-12	0.297536	0.209	0.092	9.75E-08	1 L1 _	Arhgef3
Ntan1	4.49E-12	0.425055	0.662	0.543	9.04E-08	1 L1 _	Ntan1
Slbp	4.13E-12	0.306766	0.474	0.303	8.32E-08	1 L1 _	Slbp
Capza1	4.08E-12	0.349382	0.545	0.38	8.21E-08	1 L1 _	Capza1
Klk1	4.02E-12	0.440498	0.529	0.349	8.10E-08	1 L1 _	Klk1
Hdgfl2	3.93E-12	0.375915	0.4	0.247	7.91E-08	1 L1 _	Hdgfl2
Ikbkb	3.85E-12	0.276846	0.448	0.281	7.75E-08	1 L1 _	Ikbkb
Ndufa3	3.44E-12	0.33818	0.779	0.682	6.94E-08	1 L1 _	Ndufa3
Skap2	3.39E-12	0.309916	0.262	0.132	6.83E-08	1 L1 _	Skap2
Cox7c1	2.81E-12	0.262024	0.913	0.91	5.66E-08	1 L1 _	Cox7c

Mrpl57	2.59E-12	0.41094	0.616	0.444	5.21E-08	1 L1 _	Mrpl57
Higd1a	2.43E-12	0.352871	0.602	0.437	4.89E-08	1 L1 _	Higd1a
Eif3e1	2.13E-12	0.378405	0.749	0.641	4.28E-08	1 L1 _	Eif3e
Fnbp1	2.06E-12	0.371825	0.671	0.499	4.15E-08	1 L1 _	Fnbp1
H2-Ab1	2.05E-12	1.762007	0.306	0.176	4.13E-08	1 L1 _	H2-Ab1
Exosc8	1.96E-12	0.295297	0.411	0.245	3.95E-08	1 L1 _	Exosc8
Tpm31	1.85E-12	0.345642	0.823	0.736	3.72E-08	1 L1 _	Tpm3
Arpc3	9.47E-13	0.304403	0.867	0.785	1.91E-08	1 L1 _	Arpc3
Clcn3	8.36E-13	0.261597	0.306	0.155	1.68E-08	1 L1 _	Clcn3
Ndufa41	8.21E-13	0.334336	0.837	0.714	1.65E-08	1 L1 _	Ndufa4
Psmb1	6.98E-13	0.37428	0.832	0.742	1.41E-08	1 L1 _	Psmb1
Reep51	6.74E-13	0.369305	0.814	0.729	1.36E-08	1 L1 _	Reep5
Ddx39	6.52E-13	0.252485	0.308	0.159	1.31E-08	1 L1 _	Ddx39
Oaz1	6.28E-13	0.265713	0.954	0.936	1.26E-08	1 L1 _	Oaz1
Gtf3a	5.39E-13	0.281221	0.421	0.248	1.09E-08	1 L1 _	Gtf3a
Ntpcr	5.03E-13	0.406859	0.218	0.097	1.01E-08	1 L1 _	Ntpcr
Trmt112	3.87E-13	0.389478	0.687	0.544	7.80E-09	1 L1 _	Trmt112
Lamtor4	3.53E-13	0.415051	0.69	0.554	7.10E-09	1 L1 _	Lamtor4
Map3k8	3.08E-13	0.258796	0.239	0.108	6.19E-09	1 L1 _	Map3k8
Hist1h1c	2.76E-13	0.345303	0.559	0.37	5.56E-09	1 L1 _	Hist1h1c
Psmb3	2.72E-13	0.38364	0.8	0.735	5.48E-09	1 L1 _	Psmb3
Gm17018	2.65E-13	0.262823	0.317	0.164	5.34E-09	1 L1 _	Gm17018
Snrpf	2.43E-13	0.362171	0.708	0.557	4.89E-09	1 L1 _	Snrpf
Fam214a	1.76E-13	0.297257	0.274	0.132	3.55E-09	1 L1 _	Fam214a
Rpl9-ps6	1.76E-13	0.310801	0.313	0.163	3.54E-09	1 L1 _	Rpl9-ps6
Rsbn1l1	1.56E-13	0.34102	0.648	0.465	3.14E-09	1 L1 _	Rsbn1l
Nme1	1.50E-13	0.509091	0.701	0.571	3.03E-09	1 L1 _	Nme1
Nedd91	1.20E-13	0.342322	0.506	0.317	2.42E-09	1 L1 _	Nedd9
Hist1h1e	1.19E-13	0.364404	0.554	0.365	2.39E-09	1 L1 _	Hist1h1e
Eef1g	7.82E-14	0.367633	0.814	0.752	1.57E-09	1 L1 _	Eef1g
Tuba1c	7.40E-14	0.374439	0.308	0.155	1.49E-09	1 L1 _	Tuba1c
Birc2	7.27E-14	0.413065	0.462	0.279	1.46E-09	1 L1 _	Birc2
Magohb	6.96E-14	0.267264	0.271	0.128	1.40E-09	1 L1 _	Magohb
Zmiz2	6.32E-14	0.337611	0.292	0.14	1.27E-09	1 L1 _	Zmiz2
Arpc5	5.81E-14	0.372725	0.775	0.665	1.17E-09	1 L1 _	Arpc5
Tgfb1	5.40E-14	0.329054	0.639	0.441	1.09E-09	1 L1 _	Tgfb1
1-Jun	4.36E-14	0.34885	0.832	0.666	8.78E-10	1 L1 _	Jun
Ier5	3.56E-14	0.423418	0.662	0.474	7.16E-10	1 L1 _	Ier5
Flt3l	3.50E-14	0.309265	0.349	0.18	7.05E-10	1 L1 _	Flt3l
Fryl	3.13E-14	0.330293	0.407	0.224	6.31E-10	1 L1 _	Fryl
Phgdh	3.10E-14	0.335432	0.287	0.138	6.24E-10	1 L1 _	Phgdh

Mien1	2.85E-14	0.367964	0.572	0.399	5.73E-10	1 L1 _	Mien1
Commd4	2.39E-14	0.429972	0.46	0.286	4.81E-10	1 L1 _	Commd4
Wdr1	2.17E-14	0.445167	0.522	0.348	4.36E-10	1 L1 _	Wdr1
Kxd1	1.92E-14	0.299227	0.499	0.307	3.87E-10	1 L1 _	Kxd1
Hnrnpa2b1	1.77E-14	0.391892	0.899	0.876	3.56E-10	1 L1 _	Hnrnpa2b1
Ostf12	1.70E-14	0.344226	0.736	0.583	3.43E-10	1 L1 _	Ostf1
Atp5d	1.62E-14	0.35706	0.851	0.794	3.25E-10	1 L1 _	Atp5d
Dut	1.19E-14	0.307769	0.375	0.201	2.39E-10	1 L1 _	Dut
Ubl5	1.18E-14	0.375891	0.846	0.77	2.38E-10	1 L1 _	Ubl5
Gapdh2	1.15E-14	0.392107	0.92	0.905	2.31E-10	1 L1 _	Gapdh
Mpeg1	1.13E-14	0.518139	0.14	0.044	2.28E-10	1 L1 _	Mpeg1
Snhg1	8.20E-15	0.303279	0.428	0.237	1.65E-10	1 L1 _	Snhg1
Rab8b1	6.25E-15	0.29321	0.531	0.322	1.26E-10	1 L1 _	Rab8b
Card19	6.01E-15	0.291556	0.448	0.262	1.21E-10	1 L1 _	Card19
Etv3	5.63E-15	0.356732	0.301	0.144	1.13E-10	1 L1 _	Etv3
Nosip	5.32E-15	0.341373	0.411	0.231	1.07E-10	1 L1 _	Nosip
Sun2	4.99E-15	0.266057	0.384	0.202	1.00E-10	1 L1 _	Sun2
Ube2d3	4.73E-15	0.413708	0.841	0.751	9.52E-11	1 L1 _	Ube2d3
Crlf2	4.71E-15	0.412279	0.559	0.369	9.50E-11	1 L1 _	Crlf2
Mpp7	3.89E-15	0.321839	0.276	0.126	7.84E-11	1 L1 _	Mpp7
Slc25a5	3.74E-15	0.407138	0.816	0.679	7.53E-11	1 L1 _	Slc25a5
Ciapin1	3.59E-15	0.263383	0.329	0.159	7.24E-11	1 L1 _	Ciapin1
Tut7	3.13E-15	0.262403	0.607	0.385	6.31E-11	1 L1 _	Tut7
C1qa	2.93E-15	2.456217	0.338	0.182	5.91E-11	1 L1 _	C1qa
Irf11	2.66E-15	0.3541	0.529	0.319	5.36E-11	1 L1 _	Irf1
Ubac2	2.65E-15	0.342046	0.411	0.23	5.34E-11	1 L1 _	Ubac2
Zfp318	2.44E-15	0.405137	0.409	0.237	4.91E-11	1 L1 _	Zfp318
Prkcb	2.41E-15	0.286516	0.267	0.117	4.86E-11	1 L1 _	Prkcb
Arhgap17	2.09E-15	0.361212	0.506	0.308	4.21E-11	1 L1 _	Arhgap17
Rpl10-ps3	2.07E-15	0.271794	0.322	0.156	4.17E-11	1 L1 _	Rpl10-ps3
Sdf2l1	1.76E-15	0.370411	0.368	0.197	3.54E-11	1 L1 _	Sdf2l1
Asxl2	1.53E-15	0.313901	0.503	0.304	3.08E-11	1 L1 _	Asxl2
Rpap2	1.52E-15	0.259577	0.257	0.11	3.06E-11	1 L1 _	Rpap2
Abr	1.49E-15	0.288905	0.345	0.169	3.00E-11	1 L1 _	Abr
Tpd52	1.38E-15	0.260058	0.186	0.067	2.78E-11	1 L1 _	Tpd52
Arl6ip1	1.27E-15	0.499136	0.821	0.741	2.55E-11	1 L1 _	Arl6ip1
Commd7	1.24E-15	0.469681	0.559	0.371	2.49E-11	1 L1 _	Commd7
Ptger4	1.19E-15	0.264549	0.29	0.133	2.40E-11	1 L1 _	Ptger4
Cox7b	1.19E-15	0.362863	0.839	0.768	2.39E-11	1 L1 _	Cox7b
Lsm5	1.12E-15	0.334069	0.492	0.301	2.25E-11	1 L1 _	Lsm5
Rpgrip1	1.07E-15	0.266693	0.255	0.109	2.16E-11	1 L1 _	Rpgrip1

Snhg8	1.05E-15	0.351151	0.623	0.418	2.11E-11	1 L1 _	Snhg8
Crlf3	9.30E-16	0.320728	0.423	0.229	1.87E-11	1 L1 _	Crlf3
Cd74	8.97E-16	2.678858	0.497	0.33	1.81E-11	1 L1 _	Cd74
Smc4	8.51E-16	0.331242	0.398	0.215	1.71E-11	1 L1 _	Smc4
Nhp2	8.20E-16	0.352307	0.49	0.307	1.65E-11	1 L1 _	Nhp2
Ak2	8.13E-16	0.409402	0.437	0.26	1.64E-11	1 L1 _	Ak2
Coq10b	7.60E-16	0.38602	0.476	0.281	1.53E-11	1 L1 _	Coq10b
Mettl23	6.05E-16	0.44679	0.522	0.328	1.22E-11	1 L1 _	Mettl23
Zfp512	4.68E-16	0.328305	0.405	0.22	9.43E-12	1 L1 _	Zfp512
Supt4a	4.53E-16	0.434125	0.68	0.513	9.12E-12	1 L1 _	Supt4a
Git2	3.98E-16	0.395974	0.575	0.371	8.01E-12	1 L1 _	Git2
Hnrnpf1	3.66E-16	0.466514	0.807	0.715	7.38E-12	1 L1 _	Hnrnpf
Cox5b	3.44E-16	0.441414	0.862	0.807	6.93E-12	1 L1 _	Cox5b
Taldo1	3.43E-16	0.420559	0.71	0.563	6.90E-12	1 L1 _	Taldo1
Tapbpl	3.36E-16	0.350143	0.347	0.169	6.78E-12	1 L1 _	Tapbpl
Gas5	3.14E-16	0.410498	0.89	0.675	6.33E-12	1 L1 _	Gas5
Spi1	2.37E-16	0.625844	0.14	0.04	4.77E-12	1 L1 _	Spi1
Scly	2.31E-16	0.278107	0.246	0.099	4.66E-12	1 L1 _	Scly
Csnk2b	2.22E-16	0.408465	0.674	0.503	4.48E-12	1 L1 _	Csnk2b
Sipa11	2.11E-16	0.329059	0.607	0.391	4.24E-12	1 L1 _	Sipa1
Tex9	2.02E-16	0.289954	0.202	0.074	4.06E-12	1 L1 _	Tex9
Lyz2	1.94E-16	1.402654	0.262	0.117	3.90E-12	1 L1 _	Lyz2
Sp100	1.91E-16	0.474622	0.703	0.515	3.85E-12	1 L1 _	Sp100
Fgl2	1.61E-16	0.352428	0.579	0.372	3.23E-12	1 L1 _	Fgl2
Sipa1l1	1.32E-16	0.295604	0.317	0.147	2.65E-12	1 L1 _	Sipa1l1
Srrm2	1.05E-16	0.396239	0.906	0.889	2.11E-12	1 L1 _	Srrm2
Rab8a	8.45E-17	0.425938	0.598	0.389	1.70E-12	1 L1 _	Rab8a
Bola2	7.66E-17	0.446257	0.669	0.49	1.54E-12	1 L1 _	Bola2
Zfp869	7.58E-17	0.356218	0.402	0.214	1.53E-12	1 L1 _	Zfp869
Tnik	7.49E-17	0.29089	0.338	0.163	1.51E-12	1 L1 _	Tnik
Atp6v1f	7.46E-17	0.474761	0.814	0.725	1.50E-12	1 L1 _	Atp6v1f
Tmem173	5.56E-17	0.307501	0.393	0.207	1.12E-12	1 L1 _	Tmem173
Selenoh	4.22E-17	0.386866	0.425	0.226	8.49E-13	1 L1 _	Selenoh
Ppp6r1	4.12E-17	0.358509	0.382	0.196	8.30E-13	1 L1 _	Ppp6r1
Btf3	2.85E-17	0.385275	0.887	0.871	5.74E-13	1 L1 _	Btf3
Dcps	2.70E-17	0.270286	0.324	0.146	5.43E-13	1 L1 _	Dcps
Taf15	2.46E-17	0.331278	0.577	0.362	4.95E-13	1 L1 _	Taf15
Gbp41	2.38E-17	0.344321	0.336	0.156	4.80E-13	1 L1 _	Gbp4
Tecpr1	2.00E-17	0.415933	0.448	0.245	4.03E-13	1 L1 _	Tecpr1
Fosb	1.74E-17	0.483401	0.584	0.372	3.51E-13	1 L1 _	Fosb
Tmem134	1.72E-17	0.380608	0.586	0.373	3.46E-13	1 L1 _	Tmem134

Psmb10	1.49E-17	0.39173	0.745	0.546	3.00E-13	1 L1 _	Psmb10
Rnf114	1.41E-17	0.250401	0.331	0.15	2.84E-13	1 L1 _	Rnf114
Lypla2	1.14E-17	0.326565	0.379	0.191	2.29E-13	1 L1 _	Lypla2
Tut4	1.11E-17	0.388037	0.549	0.332	2.24E-13	1 L1 _	Tut4
Nol7	1.08E-17	0.389525	0.726	0.539	2.17E-13	1 L1 _	Nol7
Cox7a2l	1.07E-17	0.440329	0.814	0.736	2.16E-13	1 L1 _	Cox7a2l
Vamp8	9.44E-18	0.43601	0.676	0.493	1.90E-13	1 L1 _	Vamp8
Ppil2	7.81E-18	0.4707	0.517	0.318	1.57E-13	1 L1 _	Ppil2
Ciao2a	7.27E-18	0.331567	0.407	0.213	1.46E-13	1 L1 _	Ciao2a
Arpc1b	6.00E-18	0.441936	0.89	0.806	1.21E-13	1 L1 _	Arpc1b
Vars	5.80E-18	0.33722	0.363	0.178	1.17E-13	1 L1 _	Vars
Dnajc2	5.28E-18	0.339807	0.428	0.228	1.06E-13	1 L1 _	Dnajc2
Snrnp70	4.59E-18	0.401324	0.848	0.725	9.24E-14	1 L1 _	Snrnp70
Sigirr1	2.80E-18	0.323116	0.207	0.07	5.64E-14	1 L1 _	Sigirr
Bin1	2.64E-18	0.396256	0.503	0.295	5.31E-14	1 L1 _	Bin1
Rnf166	2.02E-18	0.444889	0.476	0.27	4.07E-14	1 L1 _	Rnf166
Plekhj1	1.53E-18	0.457857	0.586	0.385	3.07E-14	1 L1 _	Plekhj1
Ramp1	1.48E-18	0.329556	0.255	0.097	2.97E-14	1 L1 _	Ramp1
1810026B05Rik	1.23E-18	0.478072	0.513	0.292	2.48E-14	1 L1 _	1810026B05Rik
Snx5	1.14E-18	0.518008	0.503	0.318	2.31E-14	1 L1 _	Snx5
Tomm7	1.05E-18	0.418059	0.867	0.815	2.12E-14	1 L1 _	Tomm7
Nfkbiz	8.36E-19	0.378244	0.253	0.098	1.68E-14	1 L1 _	Nfkbiz
Alox5ap	7.71E-19	0.466559	0.131	0.031	1.55E-14	1 L1 _	Alox5ap
Mllt3	6.10E-19	0.335696	0.329	0.146	1.23E-14	1 L1 _	Mllt3
Cnn22	4.91E-19	0.454645	0.625	0.403	9.88E-15	1 L1 _	Cnn2
Rp9	4.61E-19	0.481696	0.743	0.562	9.29E-15	1 L1 _	Rp9
Snrpe	4.13E-19	0.478813	0.784	0.676	8.31E-15	1 L1 _	Snrpe
Syngr2	4.06E-19	0.501687	0.538	0.327	8.18E-15	1 L1 _	Syngr2
Zfpm11	3.63E-19	0.357443	0.336	0.152	7.32E-15	1 L1 _	Zfpm1
Eif3m	3.49E-19	0.508579	0.713	0.544	7.03E-15	1 L1 _	Eif3m
Gatm	3.25E-19	0.274588	0.108	0.019	6.55E-15	1 L1 _	Gatm
Cbx4	3.24E-19	0.312557	0.301	0.126	6.53E-15	1 L1 _	Cbx4
Traf2	2.69E-19	0.324346	0.313	0.136	5.42E-15	1 L1 _	Traf2
Haus3	1.99E-19	0.366586	0.382	0.186	4.02E-15	1 L1 _	Haus3
Arpc4	1.92E-19	0.465094	0.717	0.55	3.86E-15	1 L1 _	Arpc4
Cox17	1.11E-19	0.433497	0.685	0.488	2.24E-15	1 L1 _	Cox17
Grap1	1.00E-19	0.39675	0.29	0.116	2.02E-15	1 L1 _	Grap
Rnaseh2c	8.44E-20	0.469078	0.625	0.43	1.70E-15	1 L1 _	Rnaseh2c
Rpl42	8.08E-20	0.407779	0.88	0.832	1.63E-15	1 L1 _	Rpl4
Zbp1	6.73E-20	0.442923	0.271	0.107	1.36E-15	1 L1 _	Zbp1
Lsm4	6.58E-20	0.47316	0.68	0.466	1.33E-15	1 L1 _	Lsm4

Cep250	6.50E-20	0.314427	0.292	0.119	1.31E-15	1 L1 _	Cep250
Rab4b	5.37E-20	0.436882	0.513	0.29	1.08E-15	1 L1 _	Rab4b
Smap2	5.37E-20	0.495362	0.469	0.261	1.08E-15	1 L1 _	Smap2
Cdkn2aipnl	5.28E-20	0.26446	0.257	0.094	1.06E-15	1 L1 _	Cdkn2aipnl
Mast3	4.60E-20	0.329726	0.239	0.085	9.27E-16	1 L1 _	Mast3
Rnaset2a	3.68E-20	0.492322	0.628	0.426	7.41E-16	1 L1 _	Rnaset2a
Cyld	3.42E-20	0.432887	0.451	0.24	6.90E-16	1 L1 _	Cyld
Dock5	1.89E-20	0.315141	0.306	0.126	3.81E-16	1 L1 _	Dock5
Rnasek	1.78E-20	0.421507	0.779	0.554	3.58E-16	1 L1 _	Rnasek
Ndufa6	1.35E-20	0.495384	0.839	0.706	2.72E-16	1 L1 _	Ndufa6
Cmc1	1.31E-20	0.346558	0.372	0.173	2.65E-16	1 L1 _	Cmc1
Bhlhe40	1.18E-20	0.444141	0.423	0.216	2.37E-16	1 L1 _	Bhlhe40
Elf11	9.08E-21	0.424246	0.657	0.423	1.83E-16	1 L1 _	Elf1
Card6	8.49E-21	0.260896	0.257	0.093	1.71E-16	1 L1 _	Card6
Cks2	6.52E-21	0.30433	0.154	0.037	1.31E-16	1 L1 _	Cks2
Gm10138	6.42E-21	0.283644	0.177	0.048	1.29E-16	1 L1 _	Gm10138
Csk	6.22E-21	0.45036	0.478	0.267	1.25E-16	1 L1 _	Csk
Exoc6	4.59E-21	0.27082	0.223	0.071	9.25E-17	1 L1 _	Exoc6
Slfn8	3.98E-21	0.328513	0.29	0.113	8.01E-17	1 L1 _	Slfn8
C1qc	3.73E-21	1.945643	0.269	0.108	7.52E-17	1 L1 _	C1qc
A630072M18Rik	3.49E-21	0.421081	0.336	0.145	7.03E-17	1 L1 _	A630072M18Rik
Dgkd	2.48E-21	0.412172	0.416	0.2	4.99E-17	1 L1 _	Dgkd
Ubash3b	2.08E-21	0.316512	0.322	0.133	4.19E-17	1 L1 _	Ubash3b
Csf1r	1.94E-21	0.629152	0.129	0.026	3.90E-17	1 L1 _	Csf1r
Wfdc17	1.52E-21	0.460004	0.117	0.021	3.07E-17	1 L1 _	Wfdc17
Ms4a7	1.18E-21	1.464015	0.193	0.058	2.37E-17	1 L1 _	Ms4a7
Me2	1.14E-21	0.307083	0.28	0.105	2.29E-17	1 L1 _	Me2
C1qb	8.85E-22	2.333123	0.372	0.183	1.78E-17	1 L1 _	C1qb
Ctsc	8.59E-22	0.713078	0.382	0.183	1.73E-17	1 L1 _	Ctsc
Dcxr	7.97E-22	0.318617	0.26	0.093	1.60E-17	1 L1 _	Dcxr
Ppp1r10	6.83E-22	0.445506	0.572	0.332	1.38E-17	1 L1 _	Ppp1r10
Cdk6	6.13E-22	0.388214	0.366	0.16	1.24E-17	1 L1 _	Cdk6
Vsir	5.21E-22	0.42019	0.359	0.161	1.05E-17	1 L1 _	Vsir
B930036N10Rik1	4.78E-22	0.357903	0.234	0.077	9.62E-18	1 L1 _	B930036N10Rik
Sh2d3c1	4.32E-22	0.267641	0.308	0.116	8.70E-18	1 L1 _	Sh2d3c
Irf8	3.98E-22	0.556147	0.382	0.179	8.02E-18	1 L1 _	Irf8
Vasp1	3.70E-22	0.461435	0.639	0.414	7.46E-18	1 L1 _	Vasp
D16Ertd472e	3.65E-22	0.378306	0.331	0.137	7.35E-18	1 L1 _	D16Ertd472e
Gm47664	3.55E-22	0.25628	0.154	0.035	7.16E-18	1 L1 _	Gm47664
Fuca2	3.09E-22	0.2732	0.244	0.081	6.22E-18	1 L1 _	Fuca2
Hvcn11	2.89E-22	0.329403	0.31	0.125	5.82E-18	1 L1 _	Hvcn1

Rpl31	2.23E-22	0.424672	0.917	0.871	4.50E-18	1 L1 _	Rpl31
Capzb	1.66E-22	0.526611	0.777	0.632	3.34E-18	1 L1 _	Capzb
Ppp1r15a1	1.54E-22	0.490989	0.471	0.247	3.11E-18	1 L1 _	Ppp1r15a
Slc3a21	1.27E-22	0.518148	0.752	0.55	2.57E-18	1 L1 _	Slc3a2
Cep192	9.65E-23	0.343322	0.271	0.097	1.94E-18	1 L1 _	Cep192
Cd300c2	9.32E-23	0.413729	0.115	0.018	1.88E-18	1 L1 _	Cd300c2
Evi2a	9.21E-23	0.250628	0.14	0.028	1.85E-18	1 L1 _	Evi2a
Chsy1	8.74E-23	0.395756	0.366	0.164	1.76E-18	1 L1 _	Chsy1
Cdc42se2	8.08E-23	0.43933	0.448	0.23	1.63E-18	1 L1 _	Cdc42se2
Saraf1	7.01E-23	0.525262	0.759	0.594	1.41E-18	1 L1 _	Saraf
Atp5h	6.65E-23	0.472348	0.908	0.871	1.34E-18	1 L1 _	Atp5h
Rnaset2b	6.48E-23	0.522777	0.593	0.375	1.30E-18	1 L1 _	Rnaset2b
Nsa2	4.62E-23	0.513951	0.846	0.76	9.31E-19	1 L1 _	Nsa2
Gm2000	3.99E-23	0.406459	0.556	0.318	8.05E-19	1 L1 _	Gm2000
Nr4a2	3.20E-23	0.353374	0.251	0.083	6.44E-19	1 L1 _	Nr4a2
Hspa8	2.85E-23	0.4804	0.959	0.933	5.73E-19	1 L1 _	Hspa8
Dbnl	2.08E-23	0.441028	0.545	0.307	4.20E-19	1 L1 _	Dbnl
Lrrfip1	1.43E-23	0.615683	0.641	0.444	2.89E-19	1 L1 _	Lrrfip1
Phf11b	9.96E-24	0.337614	0.189	0.049	2.01E-19	1 L1 _	Phf11b
Il7r	9.24E-24	0.312919	0.101	0.012	1.86E-19	1 L1 _	Il7r
Elmo11	8.42E-24	0.313128	0.308	0.116	1.70E-19	1 L1 _	Elmo1
Gpi1	8.17E-24	0.571425	0.749	0.584	1.64E-19	1 L1 _	Gpi1
Atp5mpl	6.59E-24	0.499954	0.798	0.59	1.33E-19	1 L1 _	Atp5mpl
Tmem179b	6.35E-24	0.410594	0.485	0.246	1.28E-19	1 L1 _	Tmem179b
Tsc22d4	4.46E-24	0.584518	0.754	0.607	8.99E-20	1 L1 _	Tsc22d4
Nfatc1	4.36E-24	0.282454	0.276	0.095	8.78E-20	1 L1 _	Nfatc1
Mgat4a1	3.80E-24	0.251968	0.248	0.077	7.65E-20	1 L1 _	Mgat4a
Cox4i1	2.82E-24	0.415794	0.947	0.933	5.68E-20	1 L1 _	Cox4i1
Slc38a2	2.47E-24	0.708381	0.83	0.726	4.98E-20	1 L1 _	Slc38a2
Tra2b	2.12E-24	0.474506	0.782	0.59	4.26E-20	1 L1 _	Tra2b
Nlrc5	1.99E-24	0.260196	0.177	0.042	4.01E-20	1 L1 _	Nlrc5
Strbp	1.73E-24	0.298077	0.306	0.113	3.49E-20	1 L1 _	Strbp
Atp5md	1.20E-24	0.480817	0.805	0.555	2.43E-20	1 L1 _	Atp5md
Ppp1ca	1.19E-24	0.524431	0.834	0.715	2.40E-20	1 L1 _	Ppp1ca
Ube2i	1.07E-24	0.532587	0.775	0.619	2.15E-20	1 L1 _	Ube2i
Cln31	9.45E-25	0.374286	0.297	0.107	1.90E-20	1 L1 _	Cln3
Adgre1	9.15E-25	0.500539	0.108	0.014	1.84E-20	1 L1 _	Adgre1
Lta4h	5.52E-25	0.522069	0.517	0.284	1.11E-20	1 L1 _	Lta4h
Elf4	1.85E-25	0.263425	0.234	0.068	3.73E-21	1 L1 _	Elf4
Slamf9	1.80E-25	0.600882	0.133	0.023	3.63E-21	1 L1 _	Slamf9
Cntrl	1.32E-25	0.426812	0.402	0.176	2.66E-21	1 L1 _	Cntrl

Rabgap1l	9.48E-26	0.450868	0.474	0.234	1.91E-21	1 L1 _	Rabgap1l
Atp11b	6.81E-26	0.511184	0.501	0.26	1.37E-21	1 L1 _	Atp11b
Psme2	5.56E-26	0.592977	0.766	0.593	1.12E-21	1 L1 _	Psme2
Vps28	5.14E-26	0.541386	0.731	0.508	1.04E-21	1 L1 _	Vps28
Rfc2	4.50E-26	0.407827	0.393	0.165	9.06E-22	1 L1 _	Rfc2
Unc119	3.15E-26	0.421412	0.407	0.181	6.35E-22	1 L1 _	Unc119
Eif3k	2.84E-26	0.496808	0.853	0.759	5.71E-22	1 L1 _	Eif3k
Ctsd	2.64E-26	0.585935	0.922	0.82	5.32E-22	1 L1 _	Ctsd
Chd2	2.29E-26	0.58414	0.628	0.369	4.61E-22	1 L1 _	Chd2
Casp8	2.21E-26	0.349943	0.418	0.178	4.45E-22	1 L1 _	Casp8
Tle4	1.89E-26	0.471518	0.533	0.275	3.80E-22	1 L1 _	Tle4
Prdx6	1.03E-26	0.624222	0.724	0.527	2.08E-22	1 L1 _	Prdx6
2310001H17Rik	9.71E-27	0.339169	0.234	0.066	1.96E-22	1 L1 _	2310001H17Rik
Stk101	7.69E-27	0.397314	0.326	0.117	1.55E-22	1 L1 _	Stk10
Itpr31	4.32E-27	0.321619	0.225	0.06	8.69E-23	1 L1 _	Itpr3
Eef1d	4.04E-27	0.526725	0.878	0.809	8.14E-23	1 L1 _	Eef1d
4930581F22Rik	3.76E-27	0.379339	0.361	0.137	7.58E-23	1 L1 _	4930581F22Rik
Ly86	3.65E-27	0.55741	0.122	0.016	7.34E-23	1 L1 _	Ly86
Ifi47	3.20E-27	0.398881	0.497	0.234	6.45E-23	1 L1 _	Ifi47
Camk1d1	2.82E-27	0.263467	0.189	0.042	5.68E-23	1 L1 _	Camk1d
Leprotl1	2.65E-27	0.630542	0.662	0.435	5.33E-23	1 L1 _	Leprotl1
2410006H16Rik	1.96E-27	0.585687	0.731	0.477	3.95E-23	1 L1 _	2410006H16Rik
Sulf21	1.77E-27	0.475167	0.386	0.163	3.56E-23	1 L1 _	Sulf2
Ppm1j	8.65E-28	0.250691	0.182	0.038	1.74E-23	1 L1 _	Ppm1j
Iqgap2	8.49E-28	0.285034	0.156	0.028	1.71E-23	1 L1 _	Iqgap2
St3gal61	7.93E-28	0.37999	0.444	0.186	1.60E-23	1 L1 _	St3gal6
Cd68	6.98E-28	0.692973	0.149	0.026	1.40E-23	1 L1 _	Cd68
Cfl1	4.55E-28	0.529158	0.929	0.901	9.16E-24	1 L1 _	Cfl1
Rbm3	1.59E-28	0.622653	0.834	0.712	3.21E-24	1 L1 _	Rbm3
Lpcat2	1.30E-28	0.381113	0.117	0.013	2.63E-24	1 L1 _	Lpcat2
Apbb1ip	9.61E-29	0.557659	0.662	0.398	1.94E-24	1 L1 _	Apbb1ip
Map11	8.22E-29	0.30532	0.179	0.036	1.66E-24	1 L1 _	Map11
Cox6a2	5.64E-29	0.89923	0.108	0.01	1.14E-24	1 L1 _	Cox6a2
Arf6	5.51E-29	0.554304	0.556	0.302	1.11E-24	1 L1 _	Arf6
Micos13	4.77E-29	0.573569	0.726	0.486	9.60E-25	1 L1 _	Micos13
Gmnn	3.97E-29	0.260666	0.193	0.041	7.99E-25	1 L1 _	Gmnn
Dusp5	3.48E-29	0.312551	0.216	0.053	7.01E-25	1 L1 _	Dusp5
Tspan131	2.93E-29	0.259249	0.402	0.144	5.90E-25	1 L1 _	Tspan13
Ddx5	2.82E-29	0.436826	0.97	0.964	5.68E-25	1 L1 _	Ddx5
Ggct	2.57E-29	0.438075	0.287	0.092	5.18E-25	1 L1 _	Ggct
Ndufb1-ps	2.18E-29	0.569197	0.749	0.53	4.40E-25	1 L1 _	Ndufb1-ps

Satb1	1.76E-29	0.27826	0.211	0.049	3.53E-25	1 L1 _	Satb1
Serpinb6b1	8.56E-30	0.297053	0.191	0.039	1.72E-25	1 L1 _	Serpinb6b
Plbd1	6.11E-30	0.368499	0.101	0.007	1.23E-25	1 L1 _	Plbd1
Diaph1	5.75E-30	0.570301	0.575	0.308	1.16E-25	1 L1 _	Diaph1
Abracl	4.83E-30	0.572816	0.651	0.405	9.74E-26	1 L1 _	Abracl
Rps17	4.48E-30	0.487352	0.936	0.906	9.03E-26	1 L1 _	Rps17
Tma7	3.96E-30	0.556744	0.848	0.722	7.97E-26	1 L1 _	Tma7
Cnp	2.14E-30	0.479391	0.343	0.123	4.32E-26	1 L1 _	Cnp
Pkn1	1.89E-30	0.497682	0.517	0.245	3.81E-26	1 L1 _	Pkn1
Atad2	1.59E-30	0.508317	0.389	0.151	3.20E-26	1 L1 _	Atad2
C730034F03Rik	1.33E-30	0.292457	0.172	0.031	2.68E-26	1 L1 _	C730034F03Rik
Hivep3	1.01E-30	0.553882	0.492	0.234	2.04E-26	1 L1 _	Hivep3
Ablim11	6.58E-31	0.609825	0.618	0.349	1.33E-26	1 L1 _	Ablim1
Rps27rt	4.50E-31	0.650476	0.533	0.274	9.07E-27	1 L1 _	Rps27rt
Glrx	3.06E-31	0.457627	0.347	0.122	6.15E-27	1 L1 _	Glrx
Trdc1	1.90E-31	0.272069	0.115	0.01	3.82E-27	1 L1 _	Trdc
Socs1	1.87E-31	0.498205	0.349	0.126	3.76E-27	1 L1 _	Socs1
Irf5	1.11E-31	0.296285	0.161	0.025	2.24E-27	1 L1 _	Irf5
Tab2	1.11E-31	0.663968	0.623	0.36	2.23E-27	1 L1 _	Tab2
Pld4	9.03E-32	0.449687	0.129	0.014	1.82E-27	1 L1 _	Pld4
Mvb12b	8.12E-32	0.35853	0.34	0.113	1.64E-27	1 L1 _	Mvb12b
Sub1	5.36E-32	0.65463	0.874	0.777	1.08E-27	1 L1 _	Sub1
Uqcrh	3.03E-32	0.526996	0.915	0.882	6.10E-28	1 L1 _	Uqcrh
Gpsm3	2.47E-32	0.774509	0.687	0.423	4.98E-28	1 L1 _	Gpsm3
Cybb	2.29E-32	0.561421	0.113	0.009	4.60E-28	1 L1 _	Cybb
Gzma	2.23E-32	2.273683	0.218	0.051	4.49E-28	1 L1 _	Gzma
Rapgef6	8.25E-33	0.669212	0.651	0.39	1.66E-28	1 L1 _	Rapgef6
Lsp1	5.10E-33	0.688815	0.72	0.462	1.03E-28	1 L1 _	Lsp1
Spata131	4.72E-33	0.369643	0.271	0.072	9.51E-29	1 L1 _	Spata13
Slfn2	4.11E-33	0.691905	0.552	0.272	8.27E-29	1 L1 _	Slfn2
Zdhhc181	2.90E-33	0.482728	0.423	0.163	5.84E-29	1 L1 _	Zdhhc18
Chl1	1.92E-33	0.486876	0.149	0.019	3.86E-29	1 L1 _	Chl1
Hpgds	1.58E-33	0.372982	0.136	0.015	3.18E-29	1 L1 _	Hpgds
Rbpj	1.03E-33	0.649462	0.655	0.398	2.08E-29	1 L1 _	Rbpj
Grk2	1.02E-33	0.488193	0.462	0.193	2.04E-29	1 L1 _	Grk2
Eif3h	3.09E-34	0.648832	0.839	0.714	6.23E-30	1 L1 _	Eif3h
Jund	2.94E-34	0.653139	0.908	0.833	5.92E-30	1 L1 _	Jund
Ripor2	2.28E-34	0.281444	0.211	0.041	4.59E-30	1 L1 _	Ripor2
Rel	2.10E-34	0.407994	0.271	0.07	4.23E-30	1 L1 _	Rel
Ppp1r16b1	1.72E-34	0.358797	0.285	0.075	3.46E-30	1 L1 _	Ppp1r16b
Nbeal2	1.64E-34	0.267454	0.202	0.038	3.31E-30	1 L1 _	Nbeal2

Zfp36l2	1.63E-34	0.755736	0.809	0.622	3.28E-30	1 L1 _	Zfp36l2
Pou2f2	1.19E-34	0.440915	0.154	0.02	2.40E-30	1 L1 _	Pou2f2
Akap13	1.19E-34	0.684801	0.844	0.662	2.39E-30	1 L1 _	Akap13
Klhl61	1.06E-34	0.35541	0.225	0.048	2.14E-30	1 L1 _	Klhl6
Peli1	9.92E-35	0.591966	0.563	0.281	2.00E-30	1 L1 _	Peli1
Ttc7	9.29E-35	0.431638	0.306	0.089	1.87E-30	1 L1 _	Ttc7
Dleu2	4.26E-35	0.443114	0.515	0.212	8.59E-31	1 L1 _	Dleu2
Taf10	4.11E-35	0.714494	0.738	0.518	8.28E-31	1 L1 _	Taf10
Gpr183	3.95E-35	0.310933	0.124	0.01	7.96E-31	1 L1 _	Gpr183
6-Sep	1.99E-35	0.522705	0.439	0.167	4.01E-31	1 L1 _	6-Sep
Rin32	1.32E-35	0.384644	0.329	0.1	2.65E-31	1 L1 _	Rin3
Cox8a1	1.26E-35	0.442961	0.949	0.945	2.53E-31	1 L1 _	Cox8a
H3f3b1	8.68E-36	0.490349	0.984	0.983	1.75E-31	1 L1 _	H3f3b
Tnfaip3	5.79E-36	0.368629	0.223	0.045	1.17E-31	1 L1 _	Tnfaip3
Sirt7	4.81E-36	0.539248	0.497	0.209	9.68E-32	1 L1 _	Sirt7
Lair1	4.38E-36	0.463437	0.126	0.01	8.83E-32	1 L1 _	Lair1
H2-DMa	4.09E-36	0.439058	0.205	0.037	8.23E-32	1 L1 _	H2-DMa
Rab19	3.72E-36	0.304436	0.17	0.024	7.49E-32	1 L1 _	Rab19
Baz1a1	3.35E-36	0.420492	0.372	0.123	6.75E-32	1 L1 _	Baz1a
Gzmk	3.05E-36	0.366849	0.101	0.004	6.13E-32	1 L1 _	Gzmk
Ms4a6c	3.04E-36	0.524781	0.163	0.022	6.12E-32	1 L1 _	Ms4a6c
Otulin	2.16E-36	0.666725	0.563	0.279	4.35E-32	1 L1 _	Otulin
AI504432	1.67E-36	0.346543	0.198	0.033	3.36E-32	1 L1 _	AI504432
H2afj	1.55E-36	0.700068	0.832	0.65	3.12E-32	1 L1 _	H2afj
H2afz	1.54E-36	0.731347	0.862	0.716	3.10E-32	1 L1 _	H2afz
Actg11	8.56E-37	0.577374	0.966	0.941	1.72E-32	1 L1 _	Actg1
Armc7	6.84E-37	0.284469	0.177	0.025	1.38E-32	1 L1 _	Armc7
Rpl35	5.67E-37	0.594603	0.894	0.851	1.14E-32	1 L1 _	Rpl35
Arpc2	4.81E-37	0.601339	0.894	0.825	9.68E-33	1 L1 _	Arpc2
Cox5a	3.29E-37	0.687394	0.897	0.74	6.64E-33	1 L1 _	Cox5a
Znrf2	1.22E-37	0.459189	0.391	0.133	2.46E-33	1 L1 _	Znrf2
Rpl22l1	7.28E-38	0.645253	0.903	0.839	1.47E-33	1 L1 _	Rpl22l1
1700028K03Rik	6.72E-38	0.276694	0.11	0.005	1.35E-33	1 L1 _	1700028K03Rik
C330011M18Rik	5.72E-38	0.326814	0.166	0.021	1.15E-33	1 L1 _	C330011M18Rik
Cd86	5.72E-38	0.253863	0.113	0.005	1.15E-33	1 L1 _	Cd86
B2m1	5.25E-38	0.47959	0.989	0.942	1.06E-33	1 L1 _	B2m
Cd28	4.31E-38	0.332555	0.106	0.004	8.68E-34	1 L1 _	Cd28
Smad71	3.12E-38	0.764063	0.667	0.355	6.29E-34	1 L1 _	Smad7
Fam102a1	1.43E-38	0.463976	0.368	0.117	2.88E-34	1 L1 _	Fam102a
Atp1b11	8.05E-39	0.312763	0.32	0.082	1.62E-34	1 L1 _	Atp1b1
St8sia41	7.20E-39	0.457381	0.474	0.165	1.45E-34	1 L1 _	St8sia4

Fam107b1	4.90E-39	0.330791	0.29	0.067	9.88E-35	1 L1 _	Fam107b
Def6	4.25E-39	0.395495	0.294	0.076	8.56E-35	1 L1 _	Def6
Ppia	2.36E-39	0.433282	0.984	0.984	4.74E-35	1 L1 _	Ppia
Afmid	2.06E-39	0.340578	0.211	0.036	4.15E-35	1 L1 _	Afmid
Acot7	1.75E-39	0.595731	0.462	0.187	3.53E-35	1 L1 _	Acot7
Ifi206	1.56E-39	0.404562	0.191	0.028	3.15E-35	1 L1 _	Ifi206
Zyx1	1.21E-39	0.762389	0.674	0.383	2.44E-35	1 L1 _	Zyx
Isg20	1.14E-39	0.532277	0.363	0.113	2.29E-35	1 L1 _	Isg20
Atp5g2	2.32E-40	0.657777	0.897	0.813	4.68E-36	1 L1 _	Atp5g2
Cd8b1	2.05E-40	1.194014	0.17	0.021	4.13E-36	1 L1 _	Cd8b1
Lgals3	1.40E-40	0.99961	0.63	0.344	2.83E-36	1 L1 _	Lgals3
Abi31	4.64E-41	0.546599	0.432	0.142	9.34E-37	1 L1 _	Abi3
Scimp	4.56E-41	0.331406	0.143	0.011	9.18E-37	1 L1 _	Scimp
Mfng	3.36E-41	0.38575	0.276	0.059	6.76E-37	1 L1 _	Mfng
Sytl22	3.13E-41	0.358615	0.237	0.043	6.30E-37	1 L1 _	Sytl2
Gmip	1.99E-41	0.456166	0.264	0.055	4.01E-37	1 L1 _	Gmip
Spin2c	1.86E-41	0.274065	0.156	0.015	3.75E-37	1 L1 _	Spin2c
Prkcz	1.41E-41	0.283741	0.207	0.031	2.85E-37	1 L1 _	Prkcz
4930520O04Rik	1.36E-41	0.273716	0.136	0.008	2.74E-37	1 L1 _	4930520O04Rik
Ly6c2	9.19E-42	1.18532	0.189	0.025	1.85E-37	1 L1 _	Ly6c2
Vps37b	6.35E-42	0.583879	0.405	0.132	1.28E-37	1 L1 _	Vps37b
Itgam	4.95E-42	0.255644	0.131	0.007	9.97E-38	1 L1 _	Itgam
Pim1	3.84E-42	0.765631	0.467	0.18	7.73E-38	1 L1 _	Pim1
Eef2	3.16E-42	0.593767	0.954	0.886	6.35E-38	1 L1 _	Eef2
Arhgef1	2.38E-42	0.721015	0.745	0.462	4.79E-38	1 L1 _	Arhgef1
Gm4070	2.32E-42	0.358014	0.29	0.064	4.68E-38	1 L1 _	Gm4070
Npm3	1.23E-42	0.612765	0.561	0.252	2.47E-38	1 L1 _	Npm3
Npm1	4.89E-43	0.674485	0.91	0.868	9.84E-39	1 L1 _	Npm1
Smpd51	9.42E-44	0.354576	0.202	0.027	1.90E-39	1 L1 _	Smpd5
Tec	2.82E-44	0.327552	0.223	0.035	5.67E-40	1 L1 _	Tec
Atp5e	1.62E-44	0.605059	0.94	0.903	3.26E-40	1 L1 _	Atp5e
Tpt1	1.17E-44	0.446098	0.991	0.986	2.36E-40	1 L1 _	Tpt1
Smyd3	7.28E-45	0.437637	0.331	0.082	1.47E-40	1 L1 _	Smyd3
Lilr4b	6.06E-45	0.348353	0.138	0.007	1.22E-40	1 L1 _	Lilr4b
Gem	3.16E-45	0.666371	0.366	0.103	6.36E-41	1 L1 _	Gem
H2afy	1.95E-45	0.751773	0.784	0.563	3.94E-41	1 L1 _	H2afy
Rpl36a	1.56E-45	0.611838	0.938	0.915	3.14E-41	1 L1 _	Rpl36a
Prr13	1.06E-45	0.781474	0.733	0.446	2.13E-41	1 L1 _	Prr13
Naca	8.93E-46	0.594037	0.94	0.922	1.80E-41	1 L1 _	Naca
Bcl2l11	8.50E-46	0.686947	0.395	0.118	1.71E-41	1 L1 _	Bcl2l11
Prkch1	4.82E-46	0.528178	0.428	0.127	9.71E-42	1 L1 _	Prkch

Casp1	4.07E-46	0.386151	0.267	0.05	8.20E-42	1 L1 _	Casp1
Plcg2	1.67E-46	0.266636	0.175	0.016	3.36E-42	1 L1 _	Plcg2
Pabpc1	7.45E-47	0.754099	0.876	0.731	1.50E-42	1 L1 _	Pabpc1
Pdcd4	6.00E-47	0.873094	0.766	0.496	1.21E-42	1 L1 _	Pdcd4
Twf2	5.04E-47	0.588762	0.453	0.155	1.02E-42	1 L1 _	Twf2
AB1246111	5.01E-47	0.32926	0.154	0.01	1.01E-42	1 L1 _	AB124611
Apobr	4.85E-47	0.413143	0.292	0.059	9.77E-43	1 L1 _	Apobr
Pfdn5	4.50E-47	0.662112	0.938	0.893	9.07E-43	1 L1 _	Pfdn5
Plaat3	4.39E-47	0.729555	0.676	0.327	8.83E-43	1 L1 _	Plaat3
Retreg1	1.88E-47	0.322612	0.191	0.02	3.79E-43	1 L1 _	Retreg1
Tnfsf101	8.65E-48	0.592418	0.503	0.167	1.74E-43	1 L1 _	Tnfsf10
Ppp1r18	5.35E-48	0.782458	0.625	0.304	1.08E-43	1 L1 _	Ppp1r18
Trac	2.25E-48	0.868141	0.241	0.038	4.52E-44	1 L1 _	Trac
Cysltr2	1.53E-48	0.259288	0.145	0.007	3.08E-44	1 L1 _	Cysltr2
Cd8a	1.47E-48	0.761658	0.161	0.011	2.96E-44	1 L1 _	Cd8a
Mxd1	1.28E-48	0.523242	0.343	0.083	2.59E-44	1 L1 _	Mxd1
Mapkapk3	8.42E-49	0.41635	0.241	0.037	1.70E-44	1 L1 _	Mapkapk3
Arid5a	7.73E-49	0.407823	0.329	0.072	1.56E-44	1 L1 _	Arid5a
Gimap9	6.71E-49	0.697421	0.499	0.18	1.35E-44	1 L1 _	Gimap9
Syk	6.52E-49	0.267362	0.172	0.014	1.31E-44	1 L1 _	Syk
Dapp11	6.23E-49	0.428762	0.26	0.045	1.26E-44	1 L1 _	Dapp1
Stk4	5.98E-49	0.62141	0.471	0.156	1.21E-44	1 L1 _	Stk4
Sh3bp1	4.49E-49	0.458193	0.329	0.075	9.03E-45	1 L1 _	Sh3bp1
Sms	2.57E-49	0.739688	0.52	0.197	5.18E-45	1 L1 _	Sms
Psmb91	1.58E-49	0.756869	0.667	0.319	3.19E-45	1 L1 _	Psmb9
Snrpg	1.18E-49	0.731574	0.901	0.813	2.38E-45	1 L1 _	Snrpg
Sp110	1.05E-49	0.734909	0.568	0.235	2.12E-45	1 L1 _	Sp110
Nt5e	8.10E-50	0.351771	0.159	0.009	1.63E-45	1 L1 _	Nt5e
AI467606	4.89E-50	0.336503	0.189	0.017	9.84E-46	1 L1 _	AI467606
Gsap	4.16E-50	0.298676	0.221	0.027	8.38E-46	1 L1 _	Gsap
P2ry10	1.69E-50	0.281121	0.149	0.007	3.39E-46	1 L1 _	P2ry10
D830025C05Rik	2.44E-51	0.475755	0.317	0.065	4.92E-47	1 L1 _	D830025C05Rik
Glcci1	2.05E-51	0.478406	0.274	0.046	4.13E-47	1 L1 _	Glcci1
Cd72	1.55E-51	0.778398	0.278	0.05	3.11E-47	1 L1 _	Cd72
N4bp2l1	7.25E-52	0.455809	0.257	0.039	1.46E-47	1 L1 _	N4bp2l1
Il10ra1	6.85E-52	0.287451	0.168	0.011	1.38E-47	1 L1 _	Il10ra
Eif3f	5.08E-52	0.762122	0.926	0.8	1.02E-47	1 L1 _	Eif3f
Grcc10	1.19E-52	0.860623	0.869	0.659	2.40E-48	1 L1 _	Grcc10
Arhgap251	5.85E-53	0.345009	0.232	0.029	1.18E-48	1 L1 _	Arhgap25
Rpl7a	5.13E-53	0.622656	0.938	0.914	1.03E-48	1 L1 _	Rpl7a
2810013P06Rik	3.29E-53	0.654104	0.503	0.168	6.62E-49	1 L1 _	2810013P06Rik

Sh3bp2	2.71E-53	0.352341	0.182	0.013	5.45E-49	1 L1 _	Sh3bp2
Jaml1	6.77E-54	0.647149	0.338	0.074	1.36E-49	1 L1 _	Jaml
Inpp4b1	6.02E-54	0.635196	0.614	0.213	1.21E-49	1 L1 _	Inpp4b
Vipr21	3.98E-54	0.382734	0.179	0.012	8.01E-50	1 L1 _	Vipr2
Lamb3	2.16E-54	0.259682	0.168	0.009	4.35E-50	1 L1 _	Lamb3
Dennd2d	8.71E-55	0.252404	0.145	0.004	1.75E-50	1 L1 _	Dennd2d
Srgap3	5.45E-55	0.417821	0.274	0.043	1.10E-50	1 L1 _	Srgap3
Zcchc18	3.57E-55	0.258332	0.168	0.008	7.19E-51	1 L1 _	Zcchc18
Tnfaip8l2	1.22E-55	0.421506	0.216	0.022	2.45E-51	1 L1 _	Tnfaip8l2
Gramd1a	5.71E-56	0.782263	0.513	0.181	1.15E-51	1 L1 _	Gramd1a
Pde3b	5.80E-57	0.438607	0.269	0.039	1.17E-52	1 L1 _	Pde3b
Fam189b	3.10E-57	0.701217	0.418	0.112	6.25E-53	1 L1 _	Fam189b
Il16	2.75E-57	0.485865	0.336	0.066	5.54E-53	1 L1 _	Il16
5830428M24Rik	2.66E-57	0.326514	0.166	0.007	5.37E-53	1 L1 _	5830428M24Rik
Junb	2.37E-57	1.003709	0.897	0.61	4.78E-53	1 L1 _	Junb
Ifng	2.33E-57	0.418873	0.166	0.007	4.68E-53	1 L1 _	Ifng
Gm36738	6.55E-58	0.410944	0.287	0.044	1.32E-53	1 L1 _	Gm36738
Ppp3cc	6.31E-58	0.531194	0.315	0.057	1.27E-53	1 L1 _	Ppp3cc
Plcxd2	3.53E-58	0.342422	0.184	0.011	7.10E-54	1 L1 _	Plcxd2
Gimap81	2.53E-58	0.514529	0.366	0.076	5.09E-54	1 L1 _	Gimap8
Asb2	7.85E-59	0.355731	0.232	0.023	1.58E-54	1 L1 _	Asb2
H2-D1	6.02E-59	0.650883	0.982	0.948	1.21E-54	1 L1 _	H2-D1
Ankrd44	3.93E-59	0.824143	0.641	0.266	7.92E-55	1 L1 _	Ankrd44
Dock10	2.44E-59	0.890773	0.715	0.348	4.91E-55	1 L1 _	Dock10
S100a101	2.37E-59	0.996973	0.86	0.654	4.77E-55	1 L1 _	S100a10
Itgad	8.50E-60	0.305811	0.156	0.004	1.71E-55	1 L1 _	Itgad
Rpl24	3.87E-60	0.594283	0.954	0.974	7.80E-56	1 L1 _	Rpl24
Lyrm9	3.55E-60	0.345415	0.205	0.015	7.16E-56	1 L1 _	Lyrm9
Bcl11b	2.83E-60	0.502456	0.186	0.01	5.70E-56	1 L1 _	Bcl11b
Faah	7.01E-61	0.426038	0.209	0.016	1.41E-56	1 L1 _	Faah
Cd247	4.97E-61	0.307474	0.184	0.009	1.00E-56	1 L1 _	Cd247
Actr3	1.25E-61	0.975029	0.83	0.54	2.53E-57	1 L1 _	Actr3
Rps6	9.37E-62	0.728568	0.959	0.888	1.89E-57	1 L1 _	Rps6
Cish	7.25E-62	0.563692	0.31	0.049	1.46E-57	1 L1 _	Cish
Mbnl1	5.81E-62	0.952815	0.897	0.791	1.17E-57	1 L1 _	Mbnl1
Jakmip11	4.75E-62	0.309285	0.182	0.008	9.56E-58	1 L1 _	Jakmip1
Ptpre1	3.52E-62	0.65627	0.462	0.124	7.09E-58	1 L1 _	Ptpre
Mob3a	3.41E-62	0.70781	0.423	0.104	6.86E-58	1 L1 _	Mob3a
Mbp	1.24E-62	0.532592	0.241	0.024	2.50E-58	1 L1 _	Mbp
Ugcg	8.03E-63	1.053049	0.621	0.267	1.62E-58	1 L1 _	Ugcg
Gm43065	7.56E-63	0.278298	0.149	0.001	1.52E-58	1 L1 _	Gm43065

Map4k1	4.24E-63	0.292902	0.182	0.007	8.55E-59	1 L1 _	Map4k1
Psme11	3.38E-63	1.000318	0.906	0.69	6.81E-59	1 L1 _	Psme1
Gata3	2.83E-63	0.38123	0.2	0.012	5.71E-59	1 L1 _	Gata3
Pag1	9.96E-64	0.537041	0.352	0.063	2.00E-59	1 L1 _	Pag1
Susd3	5.25E-64	0.340283	0.182	0.007	1.06E-59	1 L1 _	Susd3
Tnf	5.07E-64	0.393463	0.184	0.007	1.02E-59	1 L1 _	Tnf
Tmsb15b2	1.07E-64	0.26282	0.189	0.008	2.15E-60	1 L1 _	Tmsb15b2
Rbm38	7.80E-65	0.338533	0.241	0.022	1.57E-60	1 L1 _	Rbm38
Cd82	7.18E-65	0.80261	0.494	0.142	1.45E-60	1 L1 _	Cd82
Rpl28	3.57E-65	0.633461	0.977	0.973	7.19E-61	1 L1 _	Rpl28
Klrb1b1	1.94E-65	0.303733	0.191	0.008	3.90E-61	1 L1 _	Klrb1b
Gbp81	9.20E-66	0.398386	0.26	0.027	1.85E-61	1 L1 _	Gbp8
Rps8	6.17E-66	0.57867	0.995	0.992	1.24E-61	1 L1 _	Rps8
AC149090.11	5.44E-66	0.985536	0.793	0.401	1.10E-61	1 L1 _	AC149090.1
Rpl5	4.09E-66	0.830887	0.945	0.892	8.24E-62	1 L1 _	Rpl5
Klrc1	3.32E-66	0.546336	0.189	0.007	6.68E-62	1 L1 _	Klrc1
Parvg1	7.22E-67	0.297172	0.191	0.007	1.45E-62	1 L1 _	Parvg
Ctss	7.02E-67	2.256347	0.46	0.121	1.41E-62	1 L1 _	Ctss
Stk26	6.66E-67	0.350628	0.2	0.009	1.34E-62	1 L1 _	Stk26
Cd160	5.23E-67	0.552874	0.214	0.013	1.05E-62	1 L1 _	Cd160
H2-Q6	8.99E-68	0.826896	0.625	0.217	1.81E-63	1 L1 _	H2-Q6
Efhd21	5.63E-68	0.849316	0.66	0.252	1.13E-63	1 L1 _	Efhd2
Fmnl11	4.88E-68	0.492543	0.315	0.045	9.83E-64	1 L1 _	Fmnl1
Mir142hg	3.97E-68	0.394296	0.228	0.016	8.00E-64	1 L1 _	Mir142hg
Gm11808	2.80E-68	0.896527	0.722	0.356	5.64E-64	1 L1 _	Gm11808
Cyba	2.65E-68	1.036445	0.92	0.806	5.34E-64	1 L1 _	Cyba
Arhgap151	2.37E-68	0.518206	0.407	0.079	4.77E-64	1 L1 _	Arhgap15
Tspan32	5.87E-69	0.433083	0.218	0.013	1.18E-64	1 L1 _	Tspan32
Sytl3	3.04E-69	0.35732	0.228	0.015	6.12E-65	1 L1 _	Sytl3
Klra1	2.03E-69	0.867978	0.223	0.014	4.09E-65	1 L1 _	Klra1
Rab37	1.04E-69	0.38982	0.274	0.027	2.09E-65	1 L1 _	Rab37
Tmsb10	8.26E-70	1.030199	0.97	0.913	1.66E-65	1 L1 _	Tmsb10
Ezr	4.05E-71	0.646909	0.411	0.083	8.16E-67	1 L1 _	Ezr
Otulinl	3.86E-71	0.572921	0.331	0.047	7.77E-67	1 L1 _	Otulinl
Nckap1l	1.67E-71	0.422088	0.239	0.017	3.36E-67	1 L1 _	Nckap1l
Stxbp2	1.64E-71	0.388729	0.232	0.015	3.30E-67	1 L1 _	Stxbp2
Pde7a	1.42E-71	0.661004	0.428	0.092	2.86E-67	1 L1 _	Pde7a
Dnajc15	1.37E-71	1.034116	0.66	0.275	2.76E-67	1 L1 _	Dnajc15
Gng21	1.20E-71	0.864798	0.57	0.185	2.41E-67	1 L1 _	Gng2
Rnf43	9.35E-72	0.344504	0.184	0.004	1.88E-67	1 L1 _	Rnf43
Kif21b1	3.98E-72	0.399724	0.264	0.023	8.01E-68	1 L1 _	Kif21b

Sh2d1a	3.83E-72	0.388704	0.198	0.006	7.70E-68	1 L1 _	Sh2d1a
Dennd4a	2.41E-72	1.042252	0.607	0.224	4.85E-68	1 L1 _	Dennd4a
Hist1h1d1	8.02E-73	0.519019	0.239	0.016	1.61E-68	1 L1 _	Hist1h1d
Clec2i1	4.78E-73	0.380935	0.207	0.008	9.64E-69	1 L1 _	Clec2i
Hmgb21	2.14E-73	1.142611	0.717	0.319	4.30E-69	1 L1 _	Hmgb2
Rpl10	6.42E-74	0.801636	0.968	0.937	1.29E-69	1 L1 _	Rpl10
Cd27	4.54E-74	0.54288	0.32	0.04	9.14E-70	1 L1 _	Cd27
Eef1b2	2.96E-74	0.929088	0.933	0.857	5.96E-70	1 L1 _	Eef1b2
Sec11c	2.08E-74	1.043566	0.733	0.356	4.19E-70	1 L1 _	Sec11c
Bcl2a1b	1.03E-74	0.811022	0.271	0.024	2.08E-70	1 L1 _	Bcl2a1b
Fxyd5	5.69E-75	0.998308	0.917	0.71	1.15E-70	1 L1 _	Fxyd5
Chd7	5.63E-75	0.523647	0.363	0.055	1.13E-70	1 L1 _	Chd7
Mctp2	2.39E-75	0.392823	0.202	0.006	4.81E-71	1 L1 _	Mctp2
Gprin3	1.99E-75	0.484339	0.244	0.016	4.00E-71	1 L1 _	Gprin3
Slc16a6	1.36E-75	0.36094	0.237	0.014	2.74E-71	1 L1 _	Slc16a6
Gpr1741	7.95E-76	0.396446	0.218	0.009	1.60E-71	1 L1 _	Gpr174
Rpl36al	6.99E-76	0.870616	0.929	0.853	1.41E-71	1 L1 _	Rpl36al
Pik3ap1	2.30E-76	0.431767	0.234	0.013	4.64E-72	1 L1 _	Pik3ap1
Card11	1.79E-76	0.436242	0.232	0.012	3.61E-72	1 L1 _	Card11
Was	2.20E-77	0.449767	0.308	0.032	4.44E-73	1 L1 _	Was
I730030J21Rik	1.33E-77	0.371773	0.186	0.002	2.68E-73	1 L1 _	I730030J21Rik
Rpl7	4.49E-78	0.712974	0.972	0.951	9.05E-74	1 L1 _	Rpl7
Gm2682	1.34E-78	0.523926	0.239	0.013	2.69E-74	1 L1 _	Gm2682
Pstpip1	1.26E-78	0.647007	0.395	0.067	2.54E-74	1 L1 _	Pstpip1
Tox	7.18E-79	0.693162	0.333	0.043	1.45E-74	1 L1 _	Tox
Arl4c	2.62E-79	0.710371	0.359	0.052	5.28E-75	1 L1 _	Arl4c
Rps2	2.21E-79	0.755711	0.977	0.973	4.46E-75	1 L1 _	Rps2
Actb2	1.79E-79	0.773124	0.995	0.992	3.61E-75	1 L1 _	Actb
Aim21	1.20E-79	0.49674	0.292	0.026	2.43E-75	1 L1 _	Aim2
Spint2	8.91E-80	0.401556	0.253	0.015	1.79E-75	1 L1 _	Spint2
Snx20	6.39E-80	0.447807	0.262	0.018	1.29E-75	1 L1 _	Snx20
Gpr34	2.50E-80	0.412324	0.225	0.008	5.03E-76	1 L1 _	Gpr34
Pfn12	1.88E-80	1.134612	0.885	0.774	3.79E-76	1 L1 _	Pfn1
Itgb72	1.57E-80	0.691973	0.315	0.034	3.17E-76	1 L1 _	Itgb7
Osbpl3	8.31E-81	0.701379	0.418	0.071	1.67E-76	1 L1 _	Osbpl3
Vav11	4.84E-81	0.539553	0.274	0.02	9.74E-77	1 L1 _	Vav1
Tle5	3.33E-81	1.147379	0.883	0.599	6.70E-77	1 L1 _	Tle5
Gimap61	2.37E-81	1.150011	0.768	0.324	4.77E-77	1 L1 _	Gimap6
Nabp1	2.28E-81	0.621441	0.31	0.032	4.60E-77	1 L1 _	Nabp1
Inpp5d	7.78E-82	0.596807	0.379	0.055	1.57E-77	1 L1 _	Inpp5d
Rpl14	6.96E-82	0.792566	0.952	0.938	1.40E-77	1 L1 _	Rpl14

Pik3cd	6.89E-82	0.671437	0.402	0.064	1.39E-77	1 L1 _	Pik3cd
Akna1	1.83E-82	0.688388	0.395	0.062	3.68E-78	1 L1 _	Akna
Fam78a	5.24E-83	0.401497	0.234	0.009	1.06E-78	1 L1 _	Fam78a
Lilrb4a	2.25E-83	0.561359	0.244	0.011	4.53E-79	1 L1 _	Lilrb4a
Lpxn1	1.58E-83	0.607305	0.285	0.022	3.17E-79	1 L1 _	Lpxn
Rasgrp1	1.67E-84	0.470284	0.255	0.013	3.37E-80	1 L1 _	Rasgrp1
Rpl15	5.05E-85	0.749555	0.975	0.964	1.02E-80	1 L1 _	Rpl15
Eomes	4.22E-85	0.540925	0.234	0.008	8.50E-81	1 L1 _	Eomes
Arhgap41	3.88E-85	0.435737	0.257	0.013	7.82E-81	1 L1 _	Arhgap4
Rpl23a	2.50E-85	0.877161	0.961	0.916	5.03E-81	1 L1 _	Rpl23a
Clic1	2.01E-85	1.067521	0.929	0.783	4.04E-81	1 L1 _	Clic1
Tmem273	1.01E-85	0.580018	0.382	0.048	2.04E-81	1 L1 _	Tmem273
Lrmp	6.07E-86	0.379331	0.262	0.014	1.22E-81	1 L1 _	Lrmp
Rps19	4.15E-86	0.818431	0.975	0.965	8.35E-82	1 L1 _	Rps19
Uba52	3.04E-86	1.198087	0.855	0.553	6.12E-82	1 L1 _	Uba52
Jpt1	2.45E-86	1.127072	0.722	0.305	4.93E-82	1 L1 _	Jpt1
Cd3d	2.31E-86	1.521391	0.287	0.022	4.65E-82	1 L1 _	Cd3d
Rps25	2.18E-86	0.80598	0.966	0.937	4.38E-82	1 L1 _	Rps25
Rack1	9.67E-87	0.949364	0.972	0.918	1.95E-82	1 L1 _	Rack1
Lax11	5.90E-87	0.482992	0.244	0.009	1.19E-82	1 L1 _	Lax1
Nlrc3	4.05E-87	0.561602	0.276	0.017	8.15E-83	1 L1 _	Nlrc3
Rassf51	2.97E-87	0.588605	0.375	0.049	5.97E-83	1 L1 _	Rassf5
Psmb81	1.07E-87	1.129752	0.906	0.571	2.15E-83	1 L1 _	Psmb8
Tm6sf11	8.85E-88	0.623853	0.402	0.058	1.78E-83	1 L1 _	Tm6sf1
Samsn11	8.36E-88	0.554364	0.297	0.023	1.68E-83	1 L1 _	Samsn1
Napsa1	4.94E-88	0.440917	0.246	0.009	9.94E-84	1 L1 _	Napsa
Dok2	2.22E-88	0.495509	0.253	0.011	4.46E-84	1 L1 _	Dok2
Cdh12	1.50E-88	0.488673	0.276	0.016	3.02E-84	1 L1 _	Cdh1
Ubash3a	2.32E-89	0.406281	0.237	0.006	4.67E-85	1 L1 _	Ubash3a
Fcgr3	1.52E-89	0.597989	0.294	0.021	3.05E-85	1 L1 _	Fcgr3
Sla2	8.47E-90	0.461468	0.26	0.011	1.71E-85	1 L1 _	Sla2
Gm19585	8.38E-90	0.55063	0.239	0.007	1.69E-85	1 L1 _	Gm19585
Nup2101	3.55E-90	0.50965	0.269	0.013	7.15E-86	1 L1 _	Nup210
Cd3e	2.34E-90	1.222298	0.292	0.02	4.71E-86	1 L1 _	Cd3e
Arrb2	1.92E-90	0.734469	0.448	0.076	3.86E-86	1 L1 _	Arrb2
Gpr68	7.51E-91	0.584994	0.315	0.026	1.51E-86	1 L1 _	Gpr68
Plek	3.31E-91	0.739433	0.363	0.042	6.66E-87	1 L1 _	Plek
Psd41	1.02E-91	0.485959	0.301	0.021	2.05E-87	1 L1 _	Psd4
Rplp0	4.75E-92	0.900855	0.989	0.955	9.56E-88	1 L1 _	Rplp0
AU0202062	4.26E-92	1.154987	0.582	0.146	8.57E-88	1 L1 _	AU020206
Apol7e	1.64E-92	0.461551	0.244	0.006	3.31E-88	1 L1 _	Apol7e

Sh2d1b11	3.76E-93	0.460968	0.29	0.017	7.57E-89	1 L1 _	Sh2d1b1
Tbx211	2.54E-93	0.457009	0.264	0.011	5.11E-89	1 L1 _	Tbx21
Rpl29	4.60E-94	0.935049	0.959	0.932	9.26E-90	1 L1 _	Rpl29
Gimap1os	1.81E-94	0.453393	0.232	0.003	3.64E-90	1 L1 _	Gimap1os
Tnni1	1.43E-94	0.689887	0.264	0.01	2.87E-90	1 L1 _	Tnni1
Rpl26	4.82E-95	0.75346	0.984	0.961	9.72E-91	1 L1 _	Rpl26
Klra31	3.16E-95	1.091387	0.317	0.024	6.37E-91	1 L1 _	Klra3
Prkcq	5.88E-96	0.505675	0.267	0.009	1.19E-91	1 L1 _	Prkcq
Matk	4.87E-96	0.582284	0.287	0.015	9.81E-92	1 L1 _	Matk
Klk8	2.25E-96	0.757194	0.37	0.039	4.52E-92	1 L1 _	Klk8
Rps281	3.52E-97	0.968115	0.966	0.962	7.09E-93	1 L1 _	Rps28
Gm14029	9.05E-98	0.595031	0.255	0.006	1.82E-93	1 L1 _	Gm14029
Rpl22	4.04E-98	0.908667	0.954	0.931	8.13E-94	1 L1 _	Rpl22
Rpl41	1.58E-98	0.841035	0.986	0.989	3.17E-94	1 L1 _	Rpl41
Ccdc88c	1.52E-98	0.673579	0.423	0.054	3.07E-94	1 L1 _	Ccdc88c
Atp8b4	1.10E-98	0.564061	0.285	0.013	2.22E-94	1 L1 _	Atp8b4
Bcl2	7.76E-99	1.621738	0.747	0.322	1.56E-94	1 L1 _	Bcl2
Tmem1631	3.68E-99	0.427595	0.257	0.006	7.42E-95	1 L1 _	Tmem163
Ifitm10	3.12E-99	0.722134	0.4	0.049	6.28E-95	1 L1 _	Ifitm10
H2-K1	2.52E-99	1.06585	0.986	0.923	5.08E-95	1 L1 _	H2-K1
Cotl1	1.73E-99	1.378443	0.795	0.37	3.48E-95	1 L1 _	Cotl1
Rps15	1.51E-99	0.958989	0.975	0.946	3.03E-95	1 L1 _	Rps15
Klrb1a	1.21E-99	0.693617	0.329	0.024	2.45E-95	1 L1 _	Klrb1a
Grap2	1.11E-99	0.691133	0.264	0.007	2.24E-95	1 L1 _	Grap2
Chn2	1.01E-99	0.760308	0.409	0.05	2.03E-95	1 L1 _	Chn2
Ctla2b	5.17E-100	0.713366	0.366	0.035	1.04E-95	1 L1 _	Ctla2b
Rps4x	2.55E-100	0.871975	0.979	0.973	5.14E-96	1 L1 _	Rps4x
Rpl21	1.45E-100	0.840749	0.986	0.978	2.92E-96	1 L1 _	Rpl21
Arhgdib1	2.20E-101	1.322253	0.901	0.518	4.43E-97	1 L1 _	Arhgdib
Glipr11	1.39E-101	0.595071	0.324	0.021	2.81E-97	1 L1 _	Glipr1
Ctla2a1	1.06E-101	1.182877	0.795	0.257	2.13E-97	1 L1 _	Ctla2a
Myo1g	5.19E-102	0.500602	0.278	0.009	1.05E-97	1 L1 _	Myo1g
Ifi209	8.86E-104	0.635237	0.308	0.016	1.78E-99	1 L1 _	Ifi209
S100a4	1.31E-105	2.094793	0.779	0.358	2.64E-101	1 L1 _	S100a4
Sema4a	1.20E-105	0.635547	0.361	0.028	2.42E-101	1 L1 _	Sema4a
Itk	2.98E-106	0.5756	0.292	0.01	6.00E-102	1 L1 _	Itk
Malt1	1.95E-106	1.049123	0.561	0.114	3.93E-102	1 L1 _	Malt1
Sla1	1.80E-106	0.585624	0.333	0.021	3.63E-102	1 L1 _	Sla
H2-Q7	1.15E-106	1.526932	0.869	0.439	2.31E-102	1 L1 _	H2-Q7
Rhof1	6.51E-107	0.597178	0.31	0.014	1.31E-102	1 L1 _	Rhof
Cd84	1.36E-108	0.634391	0.303	0.012	2.75E-104	1 L1 _	Cd84

Rps3a1	9.69E-109	1.054344	0.975	0.963	1.95E-104	1 L1 _	Rps3a1
Rps26	5.34E-109	1.085731	0.972	0.955	1.08E-104	1 L1 _	Rps26
Rps21	2.23E-110	0.996087	0.982	0.981	4.48E-106	1 L1 _	Rps21
Rps23	2.19E-110	0.958525	0.966	0.969	4.41E-106	1 L1 _	Rps23
Rpl8	1.15E-110	0.99764	0.982	0.967	2.31E-106	1 L1 _	Rpl8
Fasl	5.24E-111	0.535374	0.29	0.007	1.05E-106	1 L1 _	Fasl
Cited41	1.78E-111	0.806745	0.379	0.031	3.59E-107	1 L1 _	Cited4
AI662270	9.37E-112	0.598945	0.317	0.014	1.89E-107	1 L1 _	AI662270
Rps27	9.20E-112	1.100557	0.986	0.975	1.85E-107	1 L1 _	Rps27
Rpl9	1.95E-112	0.977021	0.982	0.965	3.92E-108	1 L1 _	Rpl9
Rplp1	1.33E-112	0.878248	0.995	0.983	2.69E-108	1 L1 _	Rplp1
Lcp2	4.24E-113	0.55858	0.34	0.018	8.54E-109	1 L1 _	Lcp2
Rpl6	2.54E-113	0.945694	0.982	0.983	5.12E-109	1 L1 _	Rpl6
Gmfg	5.94E-115	0.880187	0.411	0.039	1.20E-110	1 L1 _	Gmfg
Btg2	3.05E-115	1.758217	0.802	0.318	6.15E-111	1 L1 _	Btg2
Rps12	1.76E-115	1.039063	0.986	0.971	3.54E-111	1 L1 _	Rps12
Rpl35a	1.25E-115	0.877438	0.984	0.983	2.52E-111	1 L1 _	Rpl35a
Spn1	1.24E-115	0.603167	0.32	0.012	2.49E-111	1 L1 _	Spn
Rpl17	1.14E-115	0.997193	0.982	0.954	2.30E-111	1 L1 _	Rpl17
Cyfip21	4.70E-116	0.683286	0.37	0.025	9.46E-112	1 L1 _	Cyfip2
Ptpn7	4.06E-116	0.592498	0.326	0.013	8.18E-112	1 L1 _	Ptpn7
Rps291	1.80E-116	0.908731	0.986	0.986	3.63E-112	1 L1 _	Rps29
Clnk1	6.53E-118	0.660507	0.322	0.011	1.31E-113	1 L1 _	Clnk
Evl1	4.87E-118	1.230926	0.713	0.207	9.81E-114	1 L1 _	Evl
Tnfrsf9	4.58E-118	0.782853	0.343	0.016	9.22E-114	1 L1 _	Tnfrsf9
Gpr651	1.66E-118	0.759299	0.356	0.019	3.35E-114	1 L1 _	Gpr65
Rpl13	1.43E-118	1.000095	0.995	0.988	2.87E-114	1 L1 _	Rpl13
Igflr1	3.06E-119	0.770272	0.379	0.026	6.16E-115	1 L1 _	Igflr1
Gm36723	1.67E-119	0.79274	0.31	0.008	3.35E-115	1 L1 _	Gm36723
Eef1a1	9.74E-120	0.931909	0.998	0.987	1.96E-115	1 L1 _	Eef1a1
Shisa5	9.09E-120	1.627566	0.931	0.735	1.83E-115	1 L1 _	Shisa5
Gimap51	4.84E-120	1.120067	0.618	0.124	9.76E-116	1 L1 _	Gimap5
Fam49b	2.45E-120	1.060181	0.602	0.121	4.93E-116	1 L1 _	Fam49b
Rpl3	1.88E-120	1.104788	0.979	0.947	3.79E-116	1 L1 _	Rpl3
Coro2a	1.11E-120	0.683477	0.363	0.02	2.23E-116	1 L1 _	Coro2a
Pycard	4.39E-121	1.187209	0.667	0.172	8.83E-117	1 L1 _	Pycard
Pkp31	2.76E-121	0.704694	0.379	0.024	5.55E-117	1 L1 _	Pkp3
Itga4	6.88E-122	0.870217	0.361	0.018	1.39E-117	1 L1 _	Itga4
Rps6ka1	6.60E-123	0.825945	0.439	0.042	1.33E-118	1 L1 _	Rps6ka1
Rpl39	5.64E-123	1.123194	0.984	0.971	1.14E-118	1 L1 _	Rpl39
Rpl37a1	5.59E-123	1.073466	0.989	0.984	1.13E-118	1 L1 _	Rpl37a

Stk17b1	5.39E-123	1.422879	0.766	0.249	1.09E-118	1 L1 _	Stk17b
Gpr171	4.98E-123	0.622265	0.324	0.009	1.00E-118	1 L1 _	Gpr171
Cxcr3	4.76E-123	0.819494	0.356	0.017	9.59E-119	1 L1 _	Cxcr3
Rpl37	4.55E-123	1.14044	0.982	0.977	9.16E-119	1 L1 _	Rpl37
Rpl10a	3.13E-123	1.148375	0.979	0.931	6.30E-119	1 L1 _	Rpl10a
Limd2	2.36E-124	1.456918	0.752	0.243	4.75E-120	1 L1 _	Limd2
Apobec3	2.13E-124	1.243714	0.699	0.179	4.30E-120	1 L1 _	Apobec3
Klrb1f	1.28E-124	0.756304	0.338	0.012	2.58E-120	1 L1 _	Klrb1f
Sash3	5.10E-125	0.659137	0.354	0.015	1.03E-120	1 L1 _	Sash3
Pglyrp2	3.59E-125	0.644224	0.326	0.009	7.24E-121	1 L1 _	Pglyrp2
Ccr21	1.40E-125	0.989625	0.379	0.022	2.81E-121	1 L1 _	Ccr2
Rps16	1.19E-125	1.109895	0.979	0.982	2.39E-121	1 L1 _	Rps16
Klri21	2.72E-126	0.901519	0.375	0.02	5.47E-122	1 L1 _	Klri2
Slc9a3r1	1.03E-126	0.849082	0.48	0.052	2.07E-122	1 L1 _	Slc9a3r1
Rps27a	5.08E-127	1.009015	0.989	0.978	1.02E-122	1 L1 _	Rps27a
Il21r	3.88E-127	0.702902	0.361	0.016	7.82E-123	1 L1 _	Il21r
Srgn2	2.81E-127	1.38863	0.897	0.312	5.66E-123	1 L1 _	Srgn
Gzmc	1.50E-127	3.790966	0.692	0.204	3.01E-123	1 L1 _	Gzmc
Rps3	1.15E-127	1.174925	0.977	0.943	2.32E-123	1 L1 _	Rps3
Rpl30	9.09E-128	1.073236	0.984	0.974	1.83E-123	1 L1 _	Rpl30
Rpl361	8.65E-128	1.198454	0.975	0.971	1.74E-123	1 L1 _	Rpl36
Cytip	2.99E-128	0.774978	0.379	0.02	6.02E-124	1 L1 _	Cytip
Rps9	1.32E-128	1.11913	0.977	0.968	2.66E-124	1 L1 _	Rps9
Rpl11	1.06E-128	1.054848	0.972	0.963	2.13E-124	1 L1 _	Rpl11
Traf3ip3	9.96E-129	0.652911	0.377	0.019	2.00E-124	1 L1 _	Traf3ip3
Klra7	2.22E-129	1.532629	0.455	0.045	4.47E-125	1 L1 _	Klra7
Rpl18	2.05E-129	1.140711	0.975	0.967	4.13E-125	1 L1 _	Rpl18
12-Sep	6.95E-130	1.322089	0.68	0.163	1.40E-125	1 L1 _	1-Sep
Rpl13a	3.69E-130	1.359861	0.982	0.862	7.43E-126	1 L1 _	Rpl13a
Rps20	4.32E-131	1.088654	0.995	0.972	8.70E-127	1 L1 _	Rps20
Sh2d2a	6.82E-132	0.830326	0.405	0.025	1.37E-127	1 L1 _	Sh2d2a
Ccl3	2.86E-132	1.287426	0.409	0.026	5.77E-128	1 L1 _	Ccl3
Rpl34	2.30E-132	1.212443	0.984	0.968	4.63E-128	1 L1 _	Rpl34
Rgs2	1.86E-132	1.370728	0.575	0.096	3.74E-128	1 L1 _	Rgs2
Tes	7.35E-133	0.831811	0.487	0.05	1.48E-128	1 L1 _	Tes
B4galnt11	4.10E-133	1.175877	0.641	0.132	8.26E-129	1 L1 _	B4galnt1
Rplp2	1.81E-133	1.146083	0.961	0.955	3.64E-129	1 L1 _	Rplp2
Tnfrsf181	1.76E-133	0.841526	0.423	0.029	3.54E-129	1 L1 _	Tnfrsf18
Itgal1	2.42E-134	0.780133	0.414	0.026	4.88E-130	1 L1 _	Itgal
Rpsa	6.48E-135	1.290134	0.986	0.974	1.31E-130	1 L1 _	Rpsa
Btg1	3.59E-135	1.999736	0.949	0.728	7.24E-131	1 L1 _	Btg1

Samd31	2.15E-135	0.792872	0.389	0.018	4.34E-131	1 L1 _	Samd3
Rps7	1.86E-135	1.289639	0.977	0.963	3.75E-131	1 L1 _	Rps7
Rps5	4.69E-136	1.172259	0.982	0.964	9.45E-132	1 L1 _	Rps5
Id21	2.33E-136	1.91556	0.869	0.419	4.69E-132	1 L1 _	Id2
Hcls1	2.18E-136	1.035124	0.522	0.062	4.38E-132	1 L1 _	Hcls1
Rpl27	9.94E-137	1.39239	0.956	0.922	2.00E-132	1 L1 _	Rpl27
Gimap11	6.16E-137	1.476396	0.724	0.17	1.24E-132	1 L1 _	Gimap1
Rps10	3.54E-137	1.210156	0.979	0.974	7.12E-133	1 L1 _	Rps10
Rps14	2.04E-137	1.136779	0.989	0.97	4.10E-133	1 L1 _	Rps14
Padi2	1.38E-137	0.828853	0.414	0.024	2.79E-133	1 L1 _	Padi2
Rpl381	2.89E-138	1.319031	0.977	0.967	5.83E-134	1 L1 _	Rpl38
Cd3g	2.23E-138	1.873649	0.434	0.031	4.49E-134	1 L1 _	Cd3g
Dennd1c	1.65E-138	0.813076	0.414	0.023	3.33E-134	1 L1 _	Dennd1c
Car21	1.10E-138	1.325667	0.533	0.064	2.22E-134	1 L1 _	Car2
Klra41	2.13E-139	1.675176	0.444	0.033	4.29E-135	1 L1 _	Klra4
Rpl19	3.88E-140	1.13681	0.989	0.985	7.81E-136	1 L1 _	Rpl19
Rpl12	2.91E-140	1.311752	0.979	0.932	5.85E-136	1 L1 _	Rpl12
Gm15472	1.13E-140	0.708038	0.356	0.008	2.28E-136	1 L1 _	Gm15472
Acap11	6.13E-142	0.862013	0.439	0.028	1.23E-137	1 L1 _	Acap1
Prf11	1.99E-142	0.860533	0.411	0.02	4.01E-138	1 L1 _	Prf1
Klrc21	7.47E-143	1.012533	0.398	0.016	1.50E-138	1 L1 _	Klrc2
Rps18	2.41E-144	1.269967	0.989	0.958	4.86E-140	1 L1 _	Rps18
Rpl23	2.07E-144	1.158231	0.991	0.978	4.17E-140	1 L1 _	Rpl23
Ms4a6b1	8.41E-145	1.167393	0.43	0.024	1.69E-140	1 L1 _	Ms4a6b
Lat	1.80E-145	0.93813	0.405	0.017	3.63E-141	1 L1 _	Lat
Trbc21	5.58E-146	2.045105	0.513	0.053	1.12E-141	1 L1 _	Trbc2
Tespa11	4.79E-146	0.826397	0.393	0.014	9.65E-142	1 L1 _	Tespa1
Traf1	1.14E-146	0.98632	0.428	0.023	2.30E-142	1 L1 _	Traf1
Gimap41	1.08E-146	1.751386	0.772	0.201	2.17E-142	1 L1 _	Gimap4
Myo1f1	1.71E-147	0.73097	0.425	0.021	3.45E-143	1 L1 _	Myo1f
Stap11	1.13E-147	0.876977	0.444	0.026	2.28E-143	1 L1 _	Stap1
Rpl27a	5.37E-148	1.177531	0.995	0.982	1.08E-143	1 L1 _	Rpl27a
Slamf7	2.89E-148	0.857185	0.4	0.015	5.82E-144	1 L1 _	Slamf7
Rps24	1.33E-150	1.339749	0.998	0.976	2.68E-146	1 L1 _	Rps24
Prex11	2.93E-151	1.072593	0.577	0.071	5.91E-147	1 L1 _	Prex1
Rpl18a	2.77E-152	1.285438	0.991	0.976	5.57E-148	1 L1 _	Rpl18a
Cd96	1.02E-152	0.775077	0.418	0.016	2.06E-148	1 L1 _	Cd96
Sh3bgrl3	2.72E-156	1.849754	0.963	0.752	5.47E-152	1 L1 _	Sh3bgrl3
Rps13	5.23E-157	1.310696	0.991	0.964	1.05E-152	1 L1 _	Rps13
Klra8	3.91E-157	1.905978	0.526	0.05	7.88E-153	1 L1 _	Klra8
Tmsb4x2	2.15E-157	1.662345	0.998	0.994	4.34E-153	1 L1 _	Tmsb4x

Cd531	1.23E-157	0.871043	0.462	0.025	2.47E-153	1 L1 _	Cd53
Lat21	5.31E-158	1.087723	0.515	0.043	1.07E-153	1 L1 _	Lat2
AW112010	2.66E-158	2.472802	0.959	0.668	5.36E-154	1 L1 _	AW112010
Rps15a	9.77E-160	1.669017	0.989	0.975	1.97E-155	1 L1 _	Rps15a
Rasal31	5.74E-160	0.857574	0.448	0.02	1.16E-155	1 L1 _	Rasal3
Stat41	4.82E-160	0.919258	0.434	0.017	9.71E-156	1 L1 _	Stat4
Rpl32	3.45E-160	1.416499	0.991	0.967	6.94E-156	1 L1 _	Rpl32
Dock8	6.53E-161	0.946226	0.554	0.051	1.32E-156	1 L1 _	Dock8
Ikzf11	5.13E-161	0.82041	0.453	0.021	1.03E-156	1 L1 _	Ikzf1
Klra91	4.44E-161	1.917673	0.531	0.048	8.93E-157	1 L1 _	Klra9
Zap701	4.11E-161	0.910469	0.467	0.025	8.27E-157	1 L1 _	Zap70
Ptk2b1	7.79E-163	0.963291	0.51	0.037	1.57E-158	1 L1 _	Ptk2b
Arhgap9	5.76E-164	1.010375	0.533	0.043	1.16E-159	1 L1 _	Arhgap9
Fau	7.99E-165	1.405987	0.991	0.984	1.61E-160	1 L1 _	Fau
Rps11	4.22E-166	1.580156	0.989	0.97	8.50E-162	1 L1 _	Rps11
Ncf4	3.74E-167	0.890401	0.483	0.025	7.53E-163	1 L1 _	Ncf4
Ipcef11	2.39E-167	0.923468	0.474	0.023	4.82E-163	1 L1 _	Ipcef1
Sorl11	7.03E-169	0.963894	0.485	0.025	1.42E-164	1 L1 _	Sorl1
Sema4d	2.70E-169	0.849324	0.434	0.012	5.44E-165	1 L1 _	Sema4d
Ucp22	6.76E-170	1.691182	0.834	0.224	1.36E-165	1 L1 _	Ucp2
Ikzf31	1.78E-171	1.010533	0.467	0.019	3.58E-167	1 L1 _	Ikzf3
Itgae	1.60E-171	1.369375	0.48	0.024	3.23E-167	1 L1 _	Itgae
Cxcr61	1.88E-172	1.300298	0.483	0.023	3.79E-168	1 L1 _	Cxcr6
Styk11	3.45E-173	1.236668	0.492	0.025	6.94E-169	1 L1 _	Styk1
Ptpn6	1.71E-173	1.473456	0.586	0.059	3.44E-169	1 L1 _	Ptpn6
Slc38a12	1.28E-175	0.932774	0.48	0.02	2.58E-171	1 L1 _	Slc38a1
Ccl4	1.98E-176	2.050028	0.589	0.056	4.00E-172	1 L1 _	Ccl4
Gzmb	5.99E-178	3.071863	0.694	0.116	1.21E-173	1 L1 _	Gzmb
Itgax	1.27E-179	1.36112	0.586	0.053	2.56E-175	1 L1 _	Itgax
Bin21	1.07E-179	0.943083	0.501	0.024	2.15E-175	1 L1 _	Bin2
Pglyrp1	8.56E-180	1.226362	0.545	0.037	1.72E-175	1 L1 _	Pglyrp1
Cst71	7.60E-181	1.175427	0.515	0.027	1.53E-176	1 L1 _	Cst7
Cd481	3.86E-181	0.955474	0.494	0.021	7.77E-177	1 L1 _	Cd48
Cd21	9.45E-182	1.193684	0.531	0.032	1.90E-177	1 L1 _	Cd2
Serpina3g	9.48E-188	1.452779	0.536	0.029	1.91E-183	1 L1 _	Serpina3g
Ncr11	5.82E-188	1.367458	0.533	0.028	1.17E-183	1 L1 _	Ncr1
Itgb21	1.94E-188	1.022791	0.529	0.025	3.91E-184	1 L1 _	Itgb2
Gimap7	1.38E-190	1.260154	0.51	0.02	2.78E-186	1 L1 _	Gimap7
Ccl5	4.67E-191	4.414308	0.952	0.456	9.40E-187	1 L1 _	Ccl5
Klrk11	1.04E-191	1.759574	0.575	0.039	2.10E-187	1 L1 _	Klrk1
Tbc1d10c1	2.70E-192	1.185904	0.554	0.033	5.43E-188	1 L1 _	Tbc1d10c

Tyrobp	1.05E-196	3.61655	0.867	0.272	2.12E-192	1 L1 _	Tyrobp
Ccr51	6.30E-197	1.24715	0.556	0.029	1.27E-192	1 L1 _	Ccr5
Fcer1g	7.32E-198	3.712658	0.88	0.299	1.47E-193	1 L1 _	Fcer1g
Trbc1	1.03E-198	1.681974	0.591	0.039	2.07E-194	1 L1 _	Trbc1
Fermt31	1.37E-200	1.060488	0.572	0.032	2.76E-196	1 L1 _	Fermt3
Cd71	6.76E-203	3.160685	0.733	0.116	1.36E-198	1 L1 _	Cd7
Cyth4	1.33E-203	1.331989	0.607	0.043	2.68E-199	1 L1 _	Cyth4
Arhgap451	2.43E-204	1.207649	0.621	0.045	4.90E-200	1 L1 _	Arhgap45
Neurl31	1.73E-204	1.264835	0.593	0.035	3.48E-200	1 L1 _	Neurl3
Arhgap30	7.47E-205	1.071798	0.563	0.027	1.50E-200	1 L1 _	Arhgap30
Cd244a	7.21E-205	1.239233	0.522	0.016	1.45E-200	1 L1 _	Cd244a
Ptpn221	1.10E-206	1.193445	0.533	0.018	2.21E-202	1 L1 _	Ptpn22
Klre11	7.72E-207	1.469215	0.568	0.027	1.56E-202	1 L1 _	Klre1
Xcl11	4.89E-208	3.266548	0.77	0.129	9.84E-204	1 L1 _	Xcl1
Rhoh	1.03E-208	1.317037	0.568	0.027	2.07E-204	1 L1 _	Rhoh
Cd226	2.43E-210	1.526177	0.556	0.023	4.90E-206	1 L1 _	Cd226
Ighm	6.53E-211	1.457304	0.623	0.044	1.32E-206	1 L1 _	Ighm
Dusp21	5.52E-214	1.987951	0.683	0.066	1.11E-209	1 L1 _	Dusp2
Skap11	1.06E-214	1.557444	0.687	0.067	2.14E-210	1 L1 _	Skap1
Rinl1	3.64E-222	1.551875	0.697	0.062	7.34E-218	1 L1 _	Rinl
Klrb1c1	2.40E-231	1.92162	0.644	0.037	4.83E-227	1 L1 _	Klrb1c
Dock21	1.21E-232	1.288423	0.628	0.03	2.43E-228	1 L1 _	Dock2
Ctsw	2.55E-235	2.853686	0.867	0.173	5.14E-231	1 L1 _	Ctsw
Il2rg1	2.07E-236	1.737611	0.733	0.068	4.18E-232	1 L1 _	Il2rg
Ptpn181	9.86E-237	2.337889	0.876	0.168	1.98E-232	1 L1 _	Ptpn18
Epsti11	2.87E-237	1.501812	0.662	0.038	5.78E-233	1 L1 _	Epsti1
Fyb	7.78E-241	1.904926	0.74	0.069	1.57E-236	1 L1 _	Fyb
Cd521	2.97E-243	3.973661	0.963	0.332	5.98E-239	1 L1 _	Cd52
Txk1	4.65E-245	2.025372	0.667	0.036	9.36E-241	1 L1 _	Txk
Klrd11	9.10E-248	2.21851	0.722	0.056	1.83E-243	1 L1 _	Klrd1
Cd371	8.48E-249	1.619183	0.733	0.058	1.71E-244	1 L1 _	Cd37
Nkg71	1.29E-249	3.332095	0.846	0.138	2.60E-245	1 L1 _	Nkg7
Lck	7.51E-252	1.720016	0.706	0.046	1.51E-247	1 L1 _	Lck
Gimap31	1.78E-254	1.947685	0.761	0.066	3.59E-250	1 L1 _	Gimap3
Il2rb1	9.39E-260	2.104634	0.761	0.064	1.89E-255	1 L1 _	Il2rb
Runx31	9.65E-261	1.567855	0.685	0.03	1.94E-256	1 L1 _	Runx3
Hcst1	8.33E-262	2.337366	0.777	0.069	1.68E-257	1 L1 _	Hcst
Lcp11	1.24E-262	2.23028	0.86	0.115	2.50E-258	1 L1 _	Lcp1
Cd691	1.53E-264	2.155166	0.726	0.043	3.09E-260	1 L1 _	Cd69
Ms4a4b1	3.68E-268	2.55112	0.768	0.06	7.42E-264	1 L1 _	Ms4a4b
Coro1a1	1.45E-275	2.475978	0.883	0.122	2.91E-271	1 L1 _	Coro1a

Rgs11	1.36E-276	2.798272	0.83	0.084	2.73E-272	1 L1 _	Rgs1
Ltb1	2.21E-278	2.693025	0.8	0.066	4.44E-274	1 L1 _	Ltb
Ptprcap1	1.67E-289	2.250279	0.793	0.054	3.36E-285	1 L1 _	Ptprcap
Laptm51	1.70E-291	2.40607	0.871	0.095	3.43E-287	1 L1 _	Laptm5
Rac21	3.05E-294	2.736721	0.906	0.119	6.15E-290	1 L1 _	Rac2
Selplg1	2.01E-299	2.035129	0.821	0.057	4.04E-295	1 L1 _	Selplg
Ptprc1	0	2.922558	0.899	0.089	0	1 L1 _	Ptprc
Hist1h1d	2.34E-06	0.311672	0.357	0.056	0.047117	1 M1 _	Hist1h1d
Il10ra	2.31E-06	0.436435	0.286	0.038	0.046523	1 M1 _	Il10ra
Eif3e	2.16E-06	1.202077	0.929	0.66	0.043577	1 M1 _	Eif3e
Arhgap15	2.01E-06	0.64443	0.571	0.138	0.040528	1 M1 _	Arhgap15
Rassf5	2.00E-06	0.51132	0.5	0.107	0.040376	1 M1 _	Rassf5
Ablim1	1.91E-06	1.188526	0.929	0.396	0.038532	1 M1 _	Ablim1
Apol9b	1.83E-06	0.423694	0.357	0.057	0.036947	1 M1 _	Apol9b
Cd48	1.77E-06	0.795157	0.5	0.106	0.03573	1 M1 _	Cd48
Cd5	1.76E-06	0.368183	0.214	0.022	0.035497	1 M1 _	Cd5
Tbc1d10c	1.71E-06	0.513958	0.571	0.127	0.034369	1 M1 _	Tbc1d10c
Dusp10	1.70E-06	0.291957	0.214	0.022	0.034174	1 M1 _	Dusp10
Mgea52	1.63E-06	0.476683	0.357	0.056	0.032781	1 M1 _	Mgea5
Ucp2	1.57E-06	1.167827	0.857	0.335	0.031656	1 M1 _	Ucp2
Fam69b1	1.54E-06	0.314229	0.214	0.022	0.030925	1 M1 _	Fam69b
Gnpda1	1.48E-06	0.442552	0.5	0.101	0.029798	1 M1 _	Gnpda1
Gm17745	1.48E-06	0.389054	0.214	0.022	0.029792	1 M1 _	Gm17745
Trbc2	1.47E-06	1.209687	0.571	0.136	0.02951	1 M1 _	Trbc2
D3Ertd254e1	1.38E-06	0.268792	0.214	0.022	0.027757	1 M1 _	D3Ertd254e
Sla	1.34E-06	0.44743	0.429	0.077	0.026924	1 M1 _	Sla
Wdr95	1.33E-06	0.368418	0.143	0.01	0.026721	1 M1 _	Wdr95
Klra9	1.28E-06	1.286366	0.571	0.136	0.025834	1 M1 _	Klra9
Mum12	1.26E-06	0.767519	0.357	0.057	0.02528	1 M1 _	Mum1
Rpl37a	1.25E-06	1.182129	1	0.985	0.025203	1 M1 _	Rpl37a
Sytl1	1.25E-06	0.66831	0.214	0.022	0.025166	1 M1 _	Sytl1
Fam208b1	1.21E-06	0.49014	0.286	0.036	0.024402	1 M1 _	Fam208b
Zfp873	1.21E-06	0.33764	0.214	0.022	0.024329	1 M1 _	Zfp873
Zap70	1.15E-06	0.710975	0.5	0.105	0.023084	1 M1 _	Zap70
Saraf	1.10E-06	0.94988	1	0.623	0.022158	1 M1 _	Saraf
3110062M04Rik1	1.03E-06	0.336004	0.286	0.036	0.020795	1 M1 _	3110062M04Rik
Zufsp2	1.02E-06	0.328232	0.286	0.036	0.020541	1 M1 _	Zufsp
Rpl36	9.91E-07	1.053331	1	0.972	0.019953	1 M1 _	Rpl36
Rps28	9.51E-07	1.308448	1	0.963	0.019155	1 M1 _	Rps28
Cln3	8.97E-07	0.724166	0.571	0.14	0.018069	1 M1 _	Cln3
Klra3	8.92E-07	0.987027	0.429	0.077	0.017956	1 M1 _	Klra3

Cd53	8.27E-07	0.881132	0.5	0.104	0.016649	1 M1 _	Cd53
Xcl1	8.19E-07	0.962252	0.786	0.245	0.016495	1 M1 _	Xcl1
Gng2	8.02E-07	0.818679	0.786	0.254	0.016158	1 M1 _	Gng2
AB124611	7.98E-07	0.295108	0.286	0.035	0.016082	1 M1 _	AB124611
Jaml	7.96E-07	0.46065	0.571	0.12	0.016026	1 M1 _	Jaml
Atp5s	7.95E-07	0.287741	0.143	0.009	0.016008	1 M1 _	Atp5s
Foxm1	7.67E-07	0.25327	0.143	0.009	0.015457	1 M1 _	Foxm1
Tmsb4x	7.52E-07	1.610477	1	0.994	0.015142	1 M1 _	Tmsb4x
Lat2	7.31E-07	0.812017	0.571	0.128	0.014714	1 M1 _	Lat2
Prc1	6.91E-07	0.573809	0.143	0.009	0.013912	1 M1 _	Prc1
Slc38a1	6.29E-07	1.014246	0.5	0.103	0.012665	1 M1 _	Slc38a1
Rin3	6.17E-07	0.7755	0.571	0.14	0.012429	1 M1 _	Rin3
Grip2	5.92E-07	0.257994	0.214	0.02	0.011923	1 M1 _	Grip2
AU020206	5.76E-07	0.67142	0.786	0.224	0.011608	1 M1 _	AU020206
Samsn1	5.76E-07	0.348166	0.429	0.071	0.011599	1 M1 _	Samsn1
Id2	4.66E-07	1.552989	0.929	0.5	0.009381	1 M1 _	Id2
Narfl	4.48E-07	0.306182	0.214	0.02	0.009019	1 M1 _	Narfl
Rps29	4.43E-07	1.318314	1	0.986	0.008916	1 M1 _	Rps29
Cd40lg	4.19E-07	0.343894	0.143	0.009	0.00843	1 M1 _	Cd40lg
Ikzf1	4.02E-07	0.931127	0.5	0.099	0.008088	1 M1 _	Ikzf1
0610037L13Rik2	3.92E-07	0.89243	0.357	0.053	0.007886	1 M1 _	0610037L13Rik
AA467197	3.45E-07	0.809241	0.214	0.02	0.006948	1 M1 _	AA467197
Cd69	3.26E-07	1.181668	0.714	0.167	0.006568	1 M1 _	Cd69
Hvcn1	3.21E-07	0.694539	0.643	0.157	0.00647	1 M1 _	Hvcn1
Cd7	2.89E-07	1.367656	0.786	0.227	0.005812	1 M1 _	Cd7
Crybg1	2.80E-07	0.404075	0.286	0.033	0.005631	1 M1 _	Crybg1
Rapsn	2.68E-07	0.386336	0.214	0.019	0.005396	1 M1 _	Rapsn
Ptpn22	2.48E-07	0.47912	0.571	0.112	0.004993	1 M1 _	Ptpn22
Klra4	2.46E-07	2.352667	0.5	0.107	0.004962	1 M1 _	Klra4
Stat4	2.35E-07	0.743259	0.5	0.092	0.004726	1 M1 _	Stat4
Prex1	2.34E-07	1.036567	0.643	0.162	0.004707	1 M1 _	Prex1
Gfi1	2.31E-07	0.569924	0.214	0.019	0.004659	1 M1 _	Gfi1
Rpl38	2.25E-07	1.322965	1	0.969	0.004525	1 M1 _	Rpl38
Gm26882	2.22E-07	0.267723	0.143	0.009	0.004467	1 M1 _	Gm26882
Ms4a6b	2.02E-07	1.686675	0.5	0.097	0.004077	1 M1 _	Ms4a6b
Lax1	1.92E-07	0.580527	0.357	0.051	0.003869	1 M1 _	Lax1
Cenpq	1.87E-07	0.474886	0.357	0.05	0.003761	1 M1 _	Cenpq
Pfn11	1.74E-07	1.597674	1	0.794	0.003504	1 M1 _	Pfn1
Il2rb	1.66E-07	1.261233	0.714	0.191	0.003338	1 M1 _	Il2rb
Car2	1.61E-07	1.247872	0.643	0.149	0.00325	1 M1 _	Car2
Ncr1	1.61E-07	1.311824	0.571	0.12	0.003248	1 M1 _	Ncr1

Ikbkg	1.57E-07	0.427069	0.5	0.089	0.003163	1 M1 _	Ikbkg
Napsa	1.42E-07	1.146743	0.357	0.051	0.002868	1 M1 _	Napsa
Camk1d	1.41E-07	0.406279	0.429	0.067	0.002847	1 M1 _	Camk1d
Fgr	1.25E-07	0.277071	0.286	0.031	0.002522	1 M1 _	Fgr
Hmgb2	9.24E-08	1.950274	0.929	0.39	0.001861	1 M1 _	Hmgb2
Pyurf2	8.76E-08	0.414219	0.357	0.046	0.001765	1 M1 _	Pyurf
Dock2	8.61E-08	0.767083	0.643	0.138	0.001734	1 M1 _	Dock2
Fam103a12	8.34E-08	0.53999	0.429	0.065	0.001679	1 M1 _	Fam103a1
Acsbg1	5.98E-08	0.376427	0.214	0.018	0.001204	1 M1 _	Acsbg1
Fam102a	5.57E-08	0.933095	0.643	0.161	0.001122	1 M1 _	Fam102a
Rhof	5.40E-08	0.957671	0.429	0.067	0.001087	1 M1 _	Rhof
Arhgap25	5.05E-08	0.497547	0.429	0.065	0.001018	1 M1 _	Arhgap25
Klrc2	4.83E-08	0.741863	0.5	0.085	0.000973	1 M1 _	Klrc2
Srgn	4.78E-08	1.596926	1	0.418	0.000962	1 M1 _	Srgn
Klrb1c	4.67E-08	1.637588	0.643	0.147	0.00094	1 M1 _	Klrb1c
Clnk	4.63E-08	0.880036	0.429	0.067	0.000932	1 M1 _	Clnk
Naip2	4.37E-08	0.77204	0.286	0.03	0.000879	1 M1 _	Naip2
Trat1	4.18E-08	0.305355	0.143	0.008	0.000843	1 M1 _	Trat1
Mirt1	3.90E-08	0.564105	0.214	0.017	0.000785	1 M1 _	Mirt1
Vav1	3.88E-08	0.61378	0.429	0.065	0.00078	1 M1 _	Vav1
Tcf7	3.36E-08	0.591859	0.286	0.03	0.000677	1 M1 _	Tcf7
Gpr174	3.22E-08	0.581214	0.357	0.046	0.000649	1 M1 _	Gpr174
Tespa1	2.81E-08	0.545047	0.5	0.082	0.000566	1 M1 _	Tespa1
Rgs1	2.66E-08	2.110985	0.786	0.219	0.000536	1 M1 _	Rgs1
Prf1	2.62E-08	1.349123	0.5	0.09	0.000528	1 M1 _	Prf1
Klri2	2.54E-08	0.719601	0.5	0.084	0.000511	1 M1 _	Klri2
B4galnt1	2.28E-08	1.034694	0.786	0.223	0.000458	1 M1 _	B4galnt1
Nkg7	2.25E-08	1.929975	0.857	0.267	0.000452	1 M1 _	Nkg7
Rinl	2.17E-08	1.323613	0.714	0.177	0.000438	1 M1 _	Rinl
Neurl3	1.99E-08	1.035838	0.643	0.136	0.000401	1 M1 _	Neurl3
Cxcr6	1.80E-08	0.883002	0.571	0.106	0.000362	1 M1 _	Cxcr6
Selp	1.68E-08	0.726261	0.143	0.007	0.000339	1 M1 _	Selp
Ptpn18	1.44E-08	1.452926	0.929	0.296	0.00029	1 M1 _	Ptpn18
Cd37	1.41E-08	0.805701	0.786	0.18	0.000284	1 M1 _	Cd37
Fcho1	1.36E-08	0.646315	0.286	0.029	0.000275	1 M1 _	Fcho1
Fermt3	1.36E-08	1.355109	0.643	0.13	0.000274	1 M1 _	Fermt3
Gm265261	1.27E-08	0.349544	0.286	0.028	0.000256	1 M1 _	Gm26526
Gpr65	1.23E-08	0.62979	0.5	0.08	0.000249	1 M1 _	Gpr65
Stk17b	1.12E-08	1.411062	0.929	0.342	0.000225	1 M1 _	Stk17b
Vipr2	1.09E-08	0.311708	0.357	0.041	0.000219	1 M1 _	Vipr2
Samd3	1.08E-08	1.025523	0.5	0.085	0.000217	1 M1 _	Samd3

Clec2i	1.07E-08	0.8894	0.357	0.043	0.000216	1 M1 _	Clec2i
Cd52	9.67E-09	2.058759	1	0.446	0.000195	1 M1 _	Cd52
Klhl6	9.19E-09	0.650985	0.5	0.078	0.000185	1 M1 _	Klhl6
Selplg	7.65E-09	1.279135	0.786	0.196	0.000154	1 M1 _	Selplg
Dapp1	7.19E-09	0.645047	0.5	0.082	0.000145	1 M1 _	Dapp1
Arnt2	5.88E-09	0.363569	0.357	0.041	0.000118	1 M1 _	Arnt2
Skap1	5.45E-09	0.991509	0.786	0.179	0.00011	1 M1 _	Skap1
Evl	5.40E-09	1.343079	0.929	0.297	0.000109	1 M1 _	Evl
Styk1	5.02E-09	1.648429	0.571	0.109	0.000101	1 M1 _	Styk1
Ptk2b	5.01E-09	0.752783	0.643	0.122	0.000101	1 M1 _	Ptk2b
Rab27a	4.58E-09	0.601278	0.286	0.027	9.22E-05	1 M1 _	Rab27a
Tmem2	4.58E-09	0.459856	0.286	0.027	9.22E-05	1 M1 _	Tmem2
Cd2	4.33E-09	0.836404	0.643	0.121	8.72E-05	1 M1 _	Cd2
Rac2	4.21E-09	1.430177	0.929	0.262	8.48E-05	1 M1 _	Rac2
Klre1	4.03E-09	1.261233	0.643	0.124	8.11E-05	1 M1 _	Klre1
Acap1	3.81E-09	1.154066	0.571	0.102	7.68E-05	1 M1 _	Acap1
Cst7	3.64E-09	0.669243	0.643	0.115	7.34E-05	1 M1 _	Cst7
Arhgap4	3.46E-09	0.518254	0.429	0.056	6.97E-05	1 M1 _	Arhgap4
Rbm121	3.07E-09	0.316554	0.286	0.026	6.19E-05	1 M1 _	Rbm12
Ccr5	3.00E-09	1.261309	0.643	0.124	6.03E-05	1 M1 _	Ccr5
Smpd5	2.93E-09	0.621751	0.429	0.058	5.91E-05	1 M1 _	Smpd5
Gm9493	2.35E-09	0.389152	0.214	0.015	4.73E-05	1 M1 _	Gm9493
Parvg	2.20E-09	0.779701	0.357	0.04	4.44E-05	1 M1 _	Parvg
Tbx21	1.97E-09	0.509688	0.429	0.056	3.97E-05	1 M1 _	Tbx21
Ms4a4b	1.77E-09	1.799564	0.786	0.188	3.56E-05	1 M1 _	Ms4a4b
Txk	1.59E-09	1.837168	0.714	0.15	3.20E-05	1 M1 _	Txk
Mkl1	1.41E-09	0.440277	0.286	0.025	2.85E-05	1 M1 _	Mkl1
Ptp4a12	1.29E-09	0.536682	0.5	0.071	2.60E-05	1 M1 _	Ptp4a1
1-Sep	1.21E-09	1.049045	0.929	0.255	2.43E-05	1 M1 _	1-Sep
Fam46a1	1.12E-09	1.207785	0.429	0.055	2.25E-05	1 M1 _	Fam46a
Gab3	1.09E-09	0.412014	0.357	0.037	2.20E-05	1 M1 _	Gab3
B3gnt5	1.07E-09	0.413827	0.214	0.014	2.16E-05	1 M1 _	B3gnt5
Myo1f	9.62E-10	0.671238	0.571	0.093	1.94E-05	1 M1 _	Myo1f
Hist1h2bb	5.45E-10	0.286971	0.143	0.006	1.10E-05	1 M1 _	Hist1h2bb
Fam96b2	4.18E-10	0.824863	0.5	0.07	8.43E-06	1 M1 _	Fam96b
Lpxn	4.00E-10	0.658605	0.5	0.068	8.06E-06	1 M1 _	Lpxn
Tmem1101	3.79E-10	0.603516	0.357	0.037	7.64E-06	1 M1 _	Tmem110
Fam208a2	3.58E-10	0.770982	0.571	0.084	7.21E-06	1 M1 _	Fam208a
Pkp3	3.19E-10	0.704966	0.571	0.087	6.42E-06	1 M1 _	Pkp3
Ptpre	2.39E-10	0.904188	0.857	0.183	4.82E-06	1 M1 _	Ptpre
Ptprcap	1.83E-10	1.361737	0.857	0.188	3.69E-06	1 M1 _	Ptprcap

Lppos1	1.74E-10	0.489261	0.286	0.023	3.51E-06	1 M1 _	Lppos
Psd4	1.70E-10	0.736296	0.5	0.07	3.42E-06	1 M1 _	Psd4
Bin2	1.67E-10	0.969817	0.643	0.109	3.37E-06	1 M1 _	Bin2
Sit1	1.50E-10	0.552632	0.214	0.013	3.02E-06	1 M1 _	Sit1
Gbp8	1.04E-10	0.830915	0.5	0.068	2.09E-06	1 M1 _	Gbp8
Tmem163	8.44E-11	0.788093	0.429	0.05	1.70E-06	1 M1 _	Tmem163
Stap1	8.38E-11	0.61677	0.643	0.1	1.69E-06	1 M1 _	Stap1
Cyfip2	8.12E-11	0.854449	0.571	0.086	1.63E-06	1 M1 _	Cyfip2
Klrk1	7.69E-11	1.516261	0.714	0.136	1.55E-06	1 M1 _	Klrk1
Ikzf3	7.00E-11	0.717783	0.643	0.099	1.41E-06	1 M1 _	Ikzf3
Sh2d1b1	6.09E-11	0.754995	0.5	0.065	1.23E-06	1 M1 _	Sh2d1b1
Ptprc	4.14E-11	2.000281	0.929	0.236	8.34E-07	1 M1 _	Ptprc
Xlr4a	3.64E-11	0.477568	0.214	0.012	7.33E-07	1 M1 _	Xlr4a
Gimap3	3.46E-11	0.91703	0.929	0.191	6.96E-07	1 M1 _	Gimap3
Zcchc62	2.69E-11	0.993212	0.643	0.097	5.41E-07	1 M1 _	Zcchc6
A430046D13Rik	2.62E-11	0.261359	0.143	0.005	5.27E-07	1 M1 _	A430046D13Rik
1110008F13Rik2	2.60E-11	0.68454	0.643	0.093	5.23E-07	1 M1 _	1110008F13Rik
Gm5547	2.01E-11	0.412063	0.357	0.032	4.04E-07	1 M1 _	Gm5547
Serpinb6b	1.73E-11	1.097059	0.5	0.065	3.49E-07	1 M1 _	Serpinb6b
Gcn1l12	1.19E-11	0.552424	0.429	0.045	2.40E-07	1 M1 _	Gcn1l1
Dusp2	1.16E-11	1.462799	0.857	0.177	2.33E-07	1 M1 _	Dusp2
Laptm5	1.13E-11	1.779198	1	0.235	2.28E-07	1 M1 _	Laptm5
Cdh1	1.04E-11	0.79553	0.5	0.062	2.10E-07	1 M1 _	Cdh1
Lcp1	9.47E-12	1.632119	1	0.249	1.91E-07	1 M1 _	Lcp1
Klrd1	8.88E-12	1.84121	0.857	0.176	1.79E-07	1 M1 _	Klrd1
Hcst	5.68E-12	1.607154	0.929	0.196	1.14E-07	1 M1 _	Hcst
Cited4	4.44E-12	1.182158	0.643	0.093	8.94E-08	1 M1 _	Cited4
Tnfrsf18	4.42E-12	0.99364	0.643	0.099	8.90E-08	1 M1 _	Tnfrsf18
Coro1a	3.82E-12	1.711574	1	0.259	7.69E-08	1 M1 _	Coro1a
Ltb	3.33E-12	1.548587	0.929	0.198	6.70E-08	1 M1 _	Ltb
Akna	3.30E-12	1.035619	0.714	0.12	6.64E-08	1 M1 _	Akna
Il2rg	3.09E-12	1.17648	0.929	0.188	6.22E-08	1 M1 _	Il2rg
B930036N10Rik	2.89E-12	1.134861	0.643	0.103	5.83E-08	1 M1 _	B930036N10Rik
Runx3	2.67E-12	1.853393	0.786	0.148	5.39E-08	1 M1 _	Runx3
Nup210	2.61E-12	0.827655	0.5	0.058	5.26E-08	1 M1 _	Nup210
Minos12	1.12E-12	1.007759	0.786	0.125	2.26E-08	1 M1 _	Minos1
AI606181	9.56E-13	0.35974	0.214	0.01	1.92E-08	1 M1 _	AI606181
Aim2	8.92E-13	0.901531	0.571	0.072	1.80E-08	1 M1 _	Aim2
Rpl27-ps3	8.57E-13	0.430382	0.214	0.01	1.73E-08	1 M1 _	Rpl27-ps3
Gm15232	6.43E-13	0.350264	0.286	0.018	1.30E-08	1 M1 _	Gm15232
Pla2g161	5.88E-13	0.938539	0.429	0.043	1.18E-08	1 M1 _	Pla2g16

AU0198232	5.40E-13	0.579692	0.429	0.04	1.09E-08	1 M1 _	AU019823
Usmg52	4.87E-13	1.296972	0.786	0.13	9.81E-09	1 M1 _	Usmg5
Trdc	3.08E-13	1.133676	0.357	0.028	6.20E-09	1 M1 _	Trdc
Ccr2	3.03E-13	0.885193	0.643	0.085	6.10E-09	1 M1 _	Ccr2
Sorl1	2.76E-13	0.90881	0.714	0.107	5.56E-09	1 M1 _	Sorl1
Arhgap45	2.55E-13	0.910495	0.857	0.148	5.14E-09	1 M1 _	Arhgap45
2010107E04Rik2	2.12E-13	1.151208	0.786	0.127	4.26E-09	1 M1 _	2010107E04Rik
Klrb1b	1.69E-13	1.091136	0.429	0.04	3.40E-09	1 M1 _	Klrb1b
Lpcat4	8.63E-14	0.851811	0.214	0.009	1.74E-09	1 M1 _	Lpcat4
Itgb7	6.30E-14	0.903802	0.643	0.083	1.27E-09	1 M1 _	Itgb7
Spn	6.10E-14	0.828541	0.571	0.066	1.23E-09	1 M1 _	Spn
Ly9	5.99E-14	0.471982	0.357	0.026	1.21E-09	1 M1 _	Ly9
Kif21b	2.91E-14	0.749965	0.571	0.065	5.85E-10	1 M1 _	Kif21b
1810022K09Rik2	2.62E-14	1.009577	0.714	0.101	5.28E-10	1 M1 _	1810022K09Rik
Rasal3	2.01E-14	0.776452	0.714	0.096	4.05E-10	1 M1 _	Rasal3
Itgb2	1.35E-14	1.008499	0.786	0.115	2.72E-10	1 M1 _	Itgb2
Itgal	1.20E-14	1.046629	0.714	0.094	2.42E-10	1 M1 _	Itgal
C920006O11Rik	8.92E-15	0.312038	0.214	0.009	1.80E-10	1 M1 _	C920006O11Rik
Ipcef1	8.79E-15	1.492504	0.714	0.103	1.77E-10	1 M1 _	Ipcef1
D17Wsu92e2	5.88E-15	0.580862	0.571	0.059	1.18E-10	1 M1 _	D17Wsu92e
Ccr9	1.69E-15	0.435168	0.143	0.003	3.40E-11	1 M1 _	Ccr9
Gm26722	1.35E-15	0.353229	0.143	0.003	2.71E-11	1 M1 _	Gm26722
Glipr1	1.22E-15	1.413765	0.643	0.074	2.46E-11	1 M1 _	Glipr1
Epsti1	4.56E-16	1.455547	0.929	0.15	9.19E-12	1 M1 _	Epsti1
Fmnl1	4.42E-16	0.931418	0.714	0.091	8.89E-12	1 M1 _	Fmnl1
Atp5o.12	3.27E-16	1.287291	0.857	0.121	6.58E-12	1 M1 _	Atp5o.1
2410015M20Rik2	1.41E-16	0.971657	0.857	0.114	2.83E-12	1 M1 _	2410015M20Rik
Zcchc112	5.85E-17	1.211646	0.643	0.071	1.18E-12	1 M1 _	Zcchc11
Il18rap	5.68E-17	0.475507	0.286	0.013	1.14E-12	1 M1 _	Il18rap
Jakmip1	1.45E-18	0.60855	0.5	0.037	2.93E-14	1 M1 _	Jakmip1
Haspin	3.75E-19	0.332743	0.286	0.012	7.56E-15	1 M1 _	Haspin
Tnfrsf13c	3.33E-19	0.281364	0.143	0.003	6.71E-15	1 M1 _	Tnfrsf13c
Aes2	2.68E-19	2.00284	0.929	0.134	5.40E-15	1 M1 _	Aes
Gpr132	2.35E-19	0.6958	0.357	0.019	4.73E-15	1 M1 _	Gpr132
Prr36	4.05E-21	0.308881	0.214	0.006	8.16E-17	1 M1 _	Prr36
Gm26853	1.51E-22	0.347901	0.357	0.015	3.05E-18	1 M1 _	Gm26853
Il18r1	1.25E-22	1.12364	0.357	0.016	2.52E-18	1 M1 _	Il18r1
2810474O19Rik2	1.40E-23	1.308218	0.786	0.076	2.81E-19	1 M1 _	2810474O19Rik
1810011H11Rik1	2.12E-27	0.651687	0.357	0.012	4.28E-23	1 M1 _	1810011H11Rik
A730071L15Rik	1.30E-30	0.367248	0.214	0.003	2.61E-26	1 M1 _	A730071L15Rik
AL590144.2	5.80E-33	0.579788	0.286	0.006	1.17E-28	1 M1 _	AL590144.2

Fam60a	3.48E-38	0.542845	0.429	0.012	7.01E-34	1 M1 _	Fam60a
Fam105a	4.60E-46	0.861878	0.357	0.006	9.26E-42	1 M1 _	Fam105a
Gm26884	2.01E-66	0.272701	0.214	0.001	4.04E-62	1 M1 _	Gm26884
Gm28053	4.07E-74	0.303334	0.143	0	8.20E-70	1 M1 _	Gm28053
Dmrta1	4.07E-74	0.261138	0.143	0	8.20E-70	1 M1 _	Dmrta1
B130006D01Rik	3.24E-91	0.417404	0.357	0.002	6.53E-87	1 M1 _	B130006D01Rik
Gm26541	8.44E-102	0.575875	0.357	0.002	1.70E-97	1 M1 _	Gm26541
AC163354.1	2.78E-110	0.429426	0.214	0	5.61E-106	1 M1 _	AC163354.1
Cd244	1.95E-130	0.783626	0.357	0.001	3.93E-126	1 M1 _	Cd244
Ptp4a31	2.43E-06	0.900777	0.383	0.32	0.04884	2 L1 _	Ptp4a3
G0s22	2.31E-06	0.636234	0.54	0.52	0.046516	2 L1 _	G0s2
Gnai11	2.13E-06	0.397178	0.128	0.058	0.042993	2 L1 _	Gnai1
Tmem941	2.13E-06	0.537286	0.204	0.12	0.042852	2 L1 _	Tmem94
P2ry14	2.11E-06	0.746971	0.409	0.364	0.042472	2 L1 _	P2ry14
Map22	1.89E-06	0.665839	0.23	0.145	0.038012	2 L1 _	Map2
Pla2r1	1.66E-06	0.78061	0.358	0.294	0.033437	2 L1 _	Pla2r1
Tmem2731	1.66E-06	0.666036	0.182	0.1	0.033382	2 L1 _	Tmem273
Hnrnpk	1.38E-06	0.373178	0.763	0.88	0.027712	2 L1 _	Hnrnpk
Pmp222	1.05E-06	0.546019	0.664	0.713	0.021074	2 L1 _	Pmp22
Tmem511	9.85E-07	0.503044	0.161	0.081	0.019848	2 L1 _	Tmem51
Hopx1	9.27E-07	0.757695	0.365	0.29	0.01866	2 L1 _	Hopx
Fam53b	9.11E-07	0.710305	0.358	0.291	0.018345	2 L1 _	Fam53b
Galnt162	8.94E-07	0.502177	0.208	0.12	0.018013	2 L1 _	Galnt16
Ifitm31	8.90E-07	0.371157	0.964	0.936	0.01793	2 L1 _	Ifitm3
Prpf4b	7.80E-07	0.529931	0.664	0.753	0.01571	2 L1 _	Prpf4b
Prkar1b1	7.18E-07	0.392635	0.117	0.048	0.014467	2 L1 _	Prkar1b
Nr1h3	6.55E-07	0.700559	0.358	0.291	0.0132	2 L1 _	Nr1h3
Gm26632	6.06E-07	0.452484	0.153	0.074	0.012211	2 L1 _	Gm26632
Spaar1	5.69E-07	0.354619	0.113	0.045	0.011463	2 L1 _	Spaar
Ncf2	5.55E-07	0.436608	0.131	0.057	0.011181	2 L1 _	Ncf2
Synpo21	5.32E-07	0.589888	0.223	0.13	0.010718	2 L1 _	Synpo2
Rabac1	5.13E-07	0.297231	0.821	0.912	0.010335	2 L1 _	Rabac1
Sorbs11	3.92E-07	0.553277	0.255	0.16	0.007892	2 L1 _	Sorbs1
4930523C07Rik2	3.60E-07	0.762661	0.46	0.433	0.007248	2 L1 _	4930523C07Rik
Pacsin31	3.53E-07	0.588395	0.197	0.109	0.007106	2 L1 _	Pacsin3
Smpdl3a2	3.51E-07	0.72406	0.485	0.48	0.00707	2 L1 _	Smpdl3a
Gm14964	3.08E-07	0.450031	0.139	0.061	0.006197	2 L1 _	Gm14964
Arglu1	2.84E-07	0.52988	0.682	0.796	0.00572	2 L1 _	Arglu1
Ndufa5	2.76E-07	0.472749	0.624	0.74	0.005559	2 L1 _	Ndufa5
Snhg181	2.49E-07	0.669305	0.613	0.627	0.005014	2 L1 _	Snhg18
Prrx12	2.31E-07	0.456914	0.745	0.715	0.004647	2 L1 _	Prrx1

Avpr1a	2.29E-07	0.655529	0.23	0.139	0.004607	2 L1 _	Avpr1a
Bcas3	2.08E-07	0.793038	0.245	0.152	0.004186	2 L1 _	Bcas3
Il342	1.85E-07	0.570314	0.551	0.491	0.003719	2 L1 _	Il34
Lpp2	1.69E-07	0.604896	0.591	0.636	0.003412	2 L1 _	Lpp
Hnrnpa2b11	1.69E-07	0.400189	0.781	0.894	0.003407	2 L1 _	Hnrnpa2b1
Fam107b2	1.52E-07	0.556414	0.19	0.098	0.003061	2 L1 _	Fam107b
Tnfrsf1a	1.51E-07	0.648054	0.635	0.729	0.003044	2 L1 _	Tnfrsf1a
Kitl2	1.46E-07	0.508073	0.482	0.4	0.002934	2 L1 _	Kitl
Cnbp	1.44E-07	0.464868	0.69	0.82	0.002891	2 L1 _	Cnbp
Mark11	1.33E-07	0.699514	0.296	0.206	0.002682	2 L1 _	Mark1
Ndufa7	1.13E-07	0.440648	0.712	0.845	0.002274	2 L1 _	Ndufa7
Atp6v0c1	1.12E-07	0.411204	0.679	0.681	0.002256	2 L1 _	Atp6v0c
Zfhx31	9.79E-08	0.782048	0.354	0.268	0.001972	2 L1 _	Zfhx3
Sem1	9.64E-08	0.406685	0.766	0.898	0.001942	2 L1 _	Sem1
Nrarp1	8.81E-08	0.537099	0.201	0.105	0.001774	2 L1 _	Nrarp
Rsu11	7.90E-08	0.648443	0.551	0.602	0.001592	2 L1 _	Rsu1
Nedd42	7.80E-08	0.330773	0.88	0.89	0.001571	2 L1 _	Nedd4
Kcne41	7.35E-08	0.487535	0.142	0.061	0.001481	2 L1 _	Kcne4
Mcam1	6.77E-08	0.535212	0.245	0.137	0.001363	2 L1 _	Mcam
Srrm21	6.43E-08	0.395779	0.818	0.902	0.001296	2 L1 _	Srrm2
Prkar1a1	5.28E-08	0.48243	0.686	0.804	0.001064	2 L1 _	Prkar1a
Gapdh3	5.00E-08	0.35873	0.825	0.919	0.001008	2 L1 _	Gapdh
Flna1	3.65E-08	0.543717	0.595	0.651	0.000736	2 L1 _	Flna
Atp5mpl1	3.57E-08	0.502021	0.62	0.63	0.000718	2 L1 _	Atp5mpl
Rsrp1	3.52E-08	0.446995	0.81	0.903	0.000708	2 L1 _	Rsrp1
Snrnp701	1.81E-08	0.611512	0.686	0.756	0.000365	2 L1 _	Snrnp70
Sema6d2	1.67E-08	0.689059	0.434	0.349	0.000337	2 L1 _	Sema6d
Arntl2	1.42E-08	0.38543	0.12	0.044	0.000287	2 L1 _	Arntl2
Jag11	1.35E-08	0.817523	0.347	0.25	0.000272	2 L1 _	Jag1
Ift431	1.20E-08	0.770904	0.416	0.343	0.000241	2 L1 _	Ift43
Slc11a11	1.16E-08	0.424028	0.106	0.034	0.000233	2 L1 _	Slc11a1
Tsc22d11	9.16E-09	0.491482	0.807	0.839	0.000184	2 L1 _	Tsc22d1
Fez22	8.36E-09	0.69074	0.449	0.371	0.000168	2 L1 _	Fez2
Uqcr111	6.48E-09	0.447853	0.723	0.82	0.000131	2 L1 _	Uqcr11
Srsf7	6.33E-09	0.760648	0.588	0.635	0.000127	2 L1 _	Srsf7
Gpx4	5.32E-09	0.4302	0.803	0.903	0.000107	2 L1 _	Gpx4
B3gnt2	5.21E-09	0.750585	0.442	0.372	0.000105	2 L1 _	B3gnt2
Jam32	4.90E-09	0.729727	0.471	0.398	9.87E-05	2 L1 _	Jam3
Pip5k1b1	4.57E-09	0.393135	0.113	0.037	9.21E-05	2 L1 _	Pip5k1b
Calm21	3.87E-09	0.420526	0.81	0.915	7.79E-05	2 L1 _	Calm2
Dstn1	3.67E-09	0.555698	0.748	0.83	7.40E-05	2 L1 _	Dstn

Ptma1	2.92E-09	0.26864	0.971	0.989	5.88E-05	2 L1 _	Ptma
Chchd21	2.84E-09	0.328638	0.934	0.965	5.72E-05	2 L1 _	Chchd2
Jph21	2.36E-09	0.343355	0.106	0.032	4.75E-05	2 L1 _	Jph2
Pitx11	1.50E-09	0.836209	0.372	0.274	3.02E-05	2 L1 _	Pitx1
Trpv21	1.47E-09	0.46836	0.161	0.065	2.97E-05	2 L1 _	Trpv2
Ccnd2	1.01E-09	0.755837	0.679	0.717	2.04E-05	2 L1 _	Ccnd2
Stard82	7.75E-10	0.647063	0.325	0.201	1.56E-05	2 L1 _	Stard8
Ggt52	6.20E-10	0.854538	0.442	0.353	1.25E-05	2 L1 _	Ggt5
Tshz21	5.60E-10	0.615383	0.639	0.642	1.13E-05	2 L1 _	Tshz2
Ecm21	5.24E-10	0.803245	0.526	0.505	1.06E-05	2 L1 _	Ecm2
Dleu21	4.15E-10	0.839057	0.369	0.255	8.36E-06	2 L1 _	Dleu2
Nckap5	3.62E-10	0.46735	0.135	0.047	7.28E-06	2 L1 _	Nckap5
Procr1	2.77E-10	0.760901	0.23	0.114	5.57E-06	2 L1 _	Procr
Pde1b1	1.91E-10	0.380682	0.131	0.043	3.85E-06	2 L1 _	Pde1b
Atp5b1	1.62E-10	0.450476	0.752	0.843	3.26E-06	2 L1 _	Atp5b
Plcl11	1.33E-10	0.759206	0.255	0.137	2.68E-06	2 L1 _	Plcl1
Gm15270	1.29E-10	0.577289	0.128	0.041	2.59E-06	2 L1 _	Gm15270
Dab2ip2	1.05E-10	0.724797	0.536	0.492	2.12E-06	2 L1 _	Dab2ip
Rnd21	5.83E-11	0.53743	0.153	0.055	1.17E-06	2 L1 _	Rnd2
Dgkg1	5.67E-11	0.353383	0.109	0.03	1.14E-06	2 L1 _	Dgkg
Dmd1	2.39E-11	0.908186	0.365	0.243	4.82E-07	2 L1 _	Dmd
Lbh	2.05E-11	0.809922	0.54	0.5	4.13E-07	2 L1 _	Lbh
Wtip1	1.87E-11	1.065573	0.427	0.334	3.77E-07	2 L1 _	Wtip
Cav12	1.38E-11	0.44128	0.478	0.29	2.77E-07	2 L1 _	Cav1
Ppargc1b1	1.38E-11	0.506726	0.131	0.04	2.77E-07	2 L1 _	Ppargc1b
Pawr1	1.18E-11	0.689732	0.252	0.126	2.38E-07	2 L1 _	Pawr
Afap1l21	7.12E-12	0.780017	0.314	0.186	1.43E-07	2 L1 _	Afap1l2
Rgs16	6.35E-12	0.612713	0.179	0.068	1.28E-07	2 L1 _	Rgs16
Fads31	5.21E-12	0.8069	0.325	0.194	1.05E-07	2 L1 _	Fads3
Id31	4.68E-12	0.683862	0.788	0.758	9.42E-08	2 L1 _	Id3
Eif1b1	2.61E-12	0.830355	0.599	0.615	5.26E-08	2 L1 _	Eif1b
Utrn2	2.61E-12	0.916251	0.558	0.482	5.25E-08	2 L1 _	Utrn
Mtus11	2.25E-12	0.870785	0.416	0.301	4.53E-08	2 L1 _	Mtus1
Khdrbs31	2.16E-12	0.691412	0.285	0.147	4.34E-08	2 L1 _	Khdrbs3
Pakap.1	1.85E-12	0.760083	0.526	0.43	3.72E-08	2 L1 _	Pakap.1
Ywhaq2	1.78E-12	0.575087	0.726	0.807	3.58E-08	2 L1 _	Ywhaq
Rbm39	1.12E-12	0.519149	0.858	0.927	2.25E-08	2 L1 _	Rbm39
Myl62	8.19E-13	0.496459	0.923	0.973	1.65E-08	2 L1 _	Myl6
Arap21	6.71E-13	0.908249	0.485	0.391	1.35E-08	2 L1 _	Arap2
Nudt4	6.47E-13	0.858524	0.584	0.574	1.30E-08	2 L1 _	Nudt4
Klf92	4.32E-13	0.526957	0.803	0.774	8.71E-09	2 L1 _	Klf9

Trpc31	4.30E-13	0.531556	0.124	0.032	8.66E-09	2 L1 _	Trpc3
Gucy1a21	3.82E-13	0.686624	0.248	0.112	7.69E-09	2 L1 _	Gucy1a2
Ccdc1601	3.63E-13	0.388092	0.106	0.024	7.31E-09	2 L1 _	Ccdc160
Herc11	3.62E-13	1.222529	0.566	0.54	7.29E-09	2 L1 _	Herc1
Dusp41	3.06E-13	0.419997	0.131	0.035	6.16E-09	2 L1 _	Dusp4
Sdc11	3.02E-13	0.906244	0.347	0.199	6.08E-09	2 L1 _	Sdc1
Cox6c1	2.45E-13	0.408405	0.85	0.936	4.93E-09	2 L1 _	Cox6c
Mapt1	2.03E-13	0.59288	0.175	0.059	4.08E-09	2 L1 _	Mapt
Gm106871	2.02E-13	0.414425	0.128	0.033	4.08E-09	2 L1 _	Gm10687
Cd362	1.78E-13	0.516038	0.361	0.184	3.58E-09	2 L1 _	Cd36
Uba21	1.64E-13	0.958164	0.5	0.425	3.31E-09	2 L1 _	Uba2
Cyp4b11	9.94E-14	0.52691	0.15	0.044	2.00E-09	2 L1 _	Cyp4b1
Cacna1c1	9.86E-14	0.93018	0.296	0.154	1.99E-09	2 L1 _	Cacna1c
Gm134701	7.49E-14	0.38761	0.128	0.032	1.51E-09	2 L1 _	Gm13470
Hspb12	5.72E-14	0.712979	0.661	0.596	1.15E-09	2 L1 _	Hspb1
Zfp4671	4.63E-14	0.710196	0.321	0.169	9.33E-10	2 L1 _	Zfp467
Nt5dc21	4.52E-14	0.786483	0.274	0.13	9.10E-10	2 L1 _	Nt5dc2
Csnk1e1	3.82E-14	0.91483	0.474	0.368	7.69E-10	2 L1 _	Csnk1e
Prkg11	2.88E-14	0.84805	0.328	0.182	5.80E-10	2 L1 _	Prkg1
Cox8a2	2.63E-14	0.447436	0.891	0.953	5.30E-10	2 L1 _	Cox8a
Txnip	2.24E-14	0.727736	0.803	0.862	4.52E-10	2 L1 _	Txnip
Raph11	1.77E-14	1.109355	0.471	0.339	3.56E-10	2 L1 _	Raph1
Vcl1	1.16E-14	0.952749	0.544	0.472	2.35E-10	2 L1 _	Vcl
Id41	7.07E-15	0.989813	0.321	0.16	1.42E-10	2 L1 _	Id4
Tns12	5.73E-15	0.854083	0.595	0.526	1.15E-10	2 L1 _	Tns1
Selenow1	5.68E-15	0.575746	0.839	0.901	1.14E-10	2 L1 _	Selenow
Timp32	4.23E-15	0.420651	0.88	0.78	8.51E-11	2 L1 _	Timp3
Elmod11	3.61E-15	0.419682	0.168	0.047	7.27E-11	2 L1 _	Elmod1
Bok1	2.93E-15	0.796475	0.281	0.131	5.90E-11	2 L1 _	Bok
Rap2a	2.59E-15	1.144548	0.467	0.352	5.22E-11	2 L1 _	Rap2a
Pnrc1	2.21E-15	0.683199	0.708	0.749	4.45E-11	2 L1 _	Pnrc1
E130102H24Rik	2.07E-15	1.030236	0.438	0.304	4.16E-11	2 L1 _	E130102H24Rik
Meox21	1.63E-15	0.737859	0.307	0.148	3.29E-11	2 L1 _	Meox2
Ajuba1	1.26E-15	0.625794	0.237	0.09	2.53E-11	2 L1 _	Ajuba
Sort11	9.63E-16	0.708869	0.252	0.102	1.94E-11	2 L1 _	Sort1
Pde8b1	9.55E-16	0.596517	0.19	0.059	1.92E-11	2 L1 _	Pde8b
Ptms1	6.16E-16	0.515333	0.894	0.907	1.24E-11	2 L1 _	Ptms
Cd1512	4.35E-16	0.789427	0.613	0.554	8.75E-12	2 L1 _	Cd151
Ppp1r1a1	3.75E-16	0.405905	0.109	0.02	7.54E-12	2 L1 _	Ppp1r1a
Rcan21	3.57E-16	0.60763	0.175	0.051	7.18E-12	2 L1 _	Rcan2
Syne22	3.48E-16	0.897061	0.544	0.406	7.01E-12	2 L1 _	Syne2

B130024G19Rik	2.53E-16	0.896535	0.358	0.195	5.09E-12	2 L1 _	B130024G19Rik
Hmgb1	2.52E-16	0.445738	0.916	0.956	5.07E-12	2 L1 _	Hmgb1
Pik3r12	1.53E-16	0.808368	0.675	0.665	3.09E-12	2 L1 _	Pik3r1
Slc25a41	1.51E-16	0.591745	0.818	0.866	3.05E-12	2 L1 _	Slc25a4
Il2ra1	1.51E-16	0.633549	0.175	0.05	3.04E-12	2 L1 _	Il2ra
Chst12	1.03E-16	0.786639	0.248	0.097	2.07E-12	2 L1 _	Chst1
Gnb41	4.80E-17	0.89966	0.686	0.668	9.68E-13	2 L1 _	Gnb4
Laptm4a2	3.97E-17	0.496806	0.956	0.922	7.99E-13	2 L1 _	Laptm4a
mt-Co3	3.70E-17	0.28163	0.993	0.993	7.44E-13	2 L1 _	mt-Co3
Arhgdib2	3.62E-17	0.84854	0.693	0.575	7.30E-13	2 L1 _	Arhgdib
Htra4	3.56E-17	0.438492	0.157	0.04	7.17E-13	2 L1 _	Htra4
Sox51	3.34E-17	0.62144	0.201	0.064	6.74E-13	2 L1 _	Sox5
Mef2a3	2.72E-17	0.82883	0.679	0.659	5.48E-13	2 L1 _	Mef2a
Ech11	2.52E-17	0.814743	0.642	0.629	5.07E-13	2 L1 _	Ech1
Tbx22	6.95E-18	1.103158	0.442	0.277	1.40E-13	2 L1 _	Tbx2
H2-M91	5.75E-18	0.628659	0.106	0.016	1.16E-13	2 L1 _	H2-M9
Slc38a111	4.85E-18	0.469167	0.128	0.025	9.78E-14	2 L1 _	Slc38a11
Cobll11	4.69E-18	0.643056	0.252	0.09	9.44E-14	2 L1 _	Cobll1
Ktn12	4.43E-18	0.929463	0.653	0.623	8.92E-14	2 L1 _	Ktn1
Dgkb1	3.02E-18	0.511642	0.168	0.042	6.09E-14	2 L1 _	Dgkb
Pdlim11	1.51E-18	0.923779	0.423	0.24	3.03E-14	2 L1 _	Pdlim1
Ptprk1	1.43E-18	1.006045	0.401	0.227	2.89E-14	2 L1 _	Ptprk
Pcdh191	1.30E-18	0.968464	0.573	0.449	2.63E-14	2 L1 _	Pcdh19
AW011738	1.30E-18	0.790931	0.263	0.099	2.61E-14	2 L1 _	AW011738
Angpt21	1.19E-18	0.700509	0.223	0.073	2.39E-14	2 L1 _	Angpt2
Fxyd12	5.41E-19	0.75688	0.723	0.673	1.09E-14	2 L1 _	Fxyd1
Rgs61	4.57E-19	0.53524	0.142	0.029	9.21E-15	2 L1 _	Rgs6
Rock11	2.89E-19	0.801517	0.723	0.741	5.81E-15	2 L1 _	Rock1
Scn3a1	1.47E-19	0.576919	0.142	0.029	2.96E-15	2 L1 _	Scn3a
Tmem1003	1.00E-19	1.033748	0.456	0.264	2.02E-15	2 L1 _	Tmem100
Myom11	7.06E-20	0.47088	0.142	0.028	1.42E-15	2 L1 _	Myom1
Tbx32	5.13E-20	0.819539	0.31	0.127	1.03E-15	2 L1 _	Tbx3
Plxdc11	2.80E-20	0.490338	0.135	0.025	5.63E-16	2 L1 _	Plxdc1
Emid11	1.41E-20	0.952583	0.416	0.218	2.83E-16	2 L1 _	Emid1
Ace21	1.40E-20	0.769059	0.226	0.064	2.83E-16	2 L1 _	Ace2
Cspg41	1.18E-20	1.040449	0.5	0.314	2.38E-16	2 L1 _	Cspg4
Nes2	1.09E-20	0.981199	0.412	0.209	2.19E-16	2 L1 _	Nes
Itga12	8.23E-21	0.632867	0.832	0.796	1.66E-16	2 L1 _	Itga1
Trpc11	6.80E-21	0.815891	0.274	0.1	1.37E-16	2 L1 _	Trpc1
Ndrg21	6.65E-21	0.982256	0.595	0.479	1.34E-16	2 L1 _	Ndrg2
Hey21	5.25E-21	0.436459	0.131	0.022	1.06E-16	2 L1 _	Hey2

Arhgap101	4.06E-21	0.957633	0.526	0.369	8.18E-17	2 L1 _	Arhgap10
Bcam2	2.80E-21	0.791906	0.54	0.304	5.63E-17	2 L1 _	Bcam
Trmt9b	2.60E-21	0.474554	0.124	0.019	5.24E-17	2 L1 _	Trmt9b
Tagln1	2.49E-21	1.441774	0.391	0.182	5.01E-17	2 L1 _	Tagln
Heyl1	2.45E-21	1.05867	0.511	0.331	4.94E-17	2 L1 _	Heyl
Olfml2b2	8.18E-22	0.855638	0.296	0.11	1.65E-17	2 L1 _	Olfml2b
Rdh51	7.94E-22	0.816367	0.266	0.089	1.60E-17	2 L1 _	Rdh5
Aoc32	7.19E-22	0.837052	0.391	0.178	1.45E-17	2 L1 _	Aoc3
Son	6.03E-22	0.616459	0.945	0.95	1.21E-17	2 L1 _	Son
Atpif11	2.64E-22	0.807477	0.745	0.782	5.31E-18	2 L1 _	Atpif1
Arhgef171	1.81E-22	1.101876	0.474	0.284	3.65E-18	2 L1 _	Arhgef17
Ddx51	1.71E-22	0.574071	0.956	0.967	3.45E-18	2 L1 _	Ddx5
Mrvi11	1.17E-22	0.978151	0.412	0.207	2.36E-18	2 L1 _	Mrvi1
Nrip21	3.93E-23	0.660657	0.164	0.032	7.92E-19	2 L1 _	Nrip2
Cbfa2t31	3.31E-23	0.975574	0.449	0.225	6.66E-19	2 L1 _	Cbfa2t3
Hrct11	2.00E-23	0.896715	0.339	0.135	4.02E-19	2 L1 _	Hrct1
Tesc1	1.92E-23	1.090247	0.259	0.078	3.87E-19	2 L1 _	Tesc
Snrk2	1.38E-23	0.872191	0.723	0.595	2.77E-19	2 L1 _	Snrk
Pcp4l12	1.17E-23	0.972626	0.438	0.198	2.35E-19	2 L1 _	Pcp4l1
Rcsd11	9.26E-24	1.041559	0.412	0.201	1.87E-19	2 L1 _	Rcsd1
Cdh21	4.10E-24	0.715141	0.172	0.034	8.25E-20	2 L1 _	Cdh2
Hrc1	1.36E-24	0.493071	0.128	0.017	2.74E-20	2 L1 _	Hrc
B430010I23Rik	1.08E-24	0.933829	0.237	0.065	2.17E-20	2 L1 _	B430010I23Rik
Pde1a1	9.51E-25	1.025852	0.372	0.163	1.92E-20	2 L1 _	Pde1a
Slc4a81	5.35E-25	0.662656	0.212	0.049	1.08E-20	2 L1 _	Slc4a8
Dtx31	4.79E-25	1.094827	0.58	0.423	9.65E-21	2 L1 _	Dtx3
Map7d21	2.18E-25	0.506028	0.15	0.023	4.39E-21	2 L1 _	Map7d2
Pakap	1.85E-25	0.80016	0.248	0.07	3.73E-21	2 L1 _	Pakap
Map3k201	1.78E-25	1.081079	0.489	0.28	3.58E-21	2 L1 _	Map3k20
Cog71	1.54E-25	1.078325	0.394	0.177	3.11E-21	2 L1 _	Cog7
Adra2a	7.87E-26	0.504145	0.113	0.012	1.58E-21	2 L1 _	Adra2a
Mustn12	5.73E-26	1.210983	0.299	0.098	1.15E-21	2 L1 _	Mustn1
Tspan152	3.88E-26	0.920202	0.372	0.146	7.81E-22	2 L1 _	Tspan15
Gprc5c1	2.76E-26	0.975979	0.369	0.152	5.56E-22	2 L1 _	Gprc5c
Arhgap421	2.12E-26	1.051122	0.401	0.177	4.28E-22	2 L1 _	Arhgap42
Map1lc3a1	2.01E-26	0.890956	0.774	0.747	4.05E-22	2 L1 _	Map1lc3a
1500009L16Rik	1.35E-26	0.695705	0.223	0.053	2.72E-22	2 L1 _	1500009L16Rik
Ppp1r14a1	1.13E-26	0.735171	0.354	0.121	2.28E-22	2 L1 _	Ppp1r14a
Tbc1d11	7.61E-27	1.105962	0.507	0.288	1.53E-22	2 L1 _	Tbc1d1
Gamt1	6.32E-27	1.143847	0.46	0.246	1.27E-22	2 L1 _	Gamt
Ccdc681	3.91E-27	0.610562	0.175	0.031	7.88E-23	2 L1 _	Ccdc68

Kcnk31	2.80E-27	0.780693	0.204	0.043	5.63E-23	2 L1 _	Kcnk3
Vstm41	1.12E-27	1.238885	0.511	0.296	2.25E-23	2 L1 _	Vstm4
Ednra1	6.96E-28	1.275125	0.606	0.435	1.40E-23	2 L1 _	Ednra
Sema5a	5.87E-28	1.17345	0.474	0.249	1.18E-23	2 L1 _	Sema5a
Klhl231	5.38E-28	0.960624	0.299	0.093	1.08E-23	2 L1 _	Klhl23
Epas12	2.85E-28	0.841806	0.726	0.504	5.74E-24	2 L1 _	Epas1
Slco3a11	2.75E-28	1.123747	0.526	0.296	5.55E-24	2 L1 _	Slco3a1
Enpep1	2.10E-28	1.114747	0.358	0.135	4.23E-24	2 L1 _	Enpep
Mef2c2	1.21E-28	0.787411	0.792	0.633	2.43E-24	2 L1 _	Mef2c
Pdgfa1	1.08E-28	1.053958	0.474	0.228	2.18E-24	2 L1 _	Pdgfa
Crip22	3.44E-29	0.860588	0.839	0.763	6.93E-25	2 L1 _	Crip2
Mgst31	1.02E-29	1.104328	0.445	0.207	2.06E-25	2 L1 _	Mgst3
Esam2	7.61E-30	0.77294	0.631	0.297	1.53E-25	2 L1 _	Esam
Casq21	5.17E-30	0.940362	0.212	0.041	1.04E-25	2 L1 _	Casq2
Ubb1	2.45E-30	0.510962	0.971	0.98	4.94E-26	2 L1 _	Ubb
Limd11	1.92E-30	1.227917	0.591	0.378	3.86E-26	2 L1 _	Limd1
Adcy61	8.65E-31	1.204447	0.427	0.185	1.74E-26	2 L1 _	Adcy6
Acta21	7.55E-31	2.047854	0.453	0.198	1.52E-26	2 L1 _	Acta2
Ebf22	4.36E-31	1.02455	0.401	0.15	8.79E-27	2 L1 _	Ebf2
Chn11	2.53E-31	0.805592	0.255	0.059	5.10E-27	2 L1 _	Chn1
Lin7a1	4.73E-32	1.29611	0.529	0.288	9.52E-28	2 L1 _	Lin7a
Fzd42	2.77E-32	1.04994	0.595	0.316	5.57E-28	2 L1 _	Fzd4
Stk38l1	2.22E-33	0.977675	0.35	0.113	4.46E-29	2 L1 _	Stk38l
Agtr1a1	1.05E-33	1.013423	0.328	0.092	2.12E-29	2 L1 _	Agtr1a
Ccdc1411	1.00E-33	0.941305	0.288	0.072	2.02E-29	2 L1 _	Ccdc141
Tnfrsf21	8.31E-34	1.287228	0.529	0.281	1.67E-29	2 L1 _	Tnfrsf21
Rrad	7.75E-35	1.024502	0.328	0.094	1.56E-30	2 L1 _	Rrad
Pten1	7.03E-35	1.074515	0.788	0.719	1.42E-30	2 L1 _	Pten
Ppp1r12a1	5.70E-35	1.186444	0.73	0.635	1.15E-30	2 L1 _	Ppp1r12a
Npy1r1	4.04E-35	1.019429	0.332	0.092	8.14E-31	2 L1 _	Npy1r
Sgip11	2.25E-35	0.899916	0.281	0.065	4.53E-31	2 L1 _	Sgip1
Tbx3os11	5.04E-36	0.735019	0.219	0.036	1.01E-31	2 L1 _	Tbx3os1
Mylk1	3.86E-36	1.021842	0.788	0.619	7.77E-32	2 L1 _	Mylk
Daam21	1.15E-36	1.06811	0.398	0.13	2.31E-32	2 L1 _	Daam2
Arhgef161	1.13E-36	0.567736	0.168	0.018	2.28E-32	2 L1 _	Arhgef16
Btbd31	9.46E-37	1.297004	0.533	0.262	1.90E-32	2 L1 _	Btbd3
Adap2os1	6.35E-37	1.082621	0.405	0.133	1.28E-32	2 L1 _	Adap2os
Rbpms21	3.83E-37	1.084199	0.31	0.077	7.71E-33	2 L1 _	Rbpms2
Dlc12	3.76E-37	1.139478	0.792	0.694	7.56E-33	2 L1 _	Dlc1
Pdlim31	2.85E-37	0.748636	0.237	0.042	5.74E-33	2 L1 _	Pdlim3
Cst31	1.61E-37	0.521758	0.982	0.95	3.24E-33	2 L1 _	Cst3

Fam162a1	1.22E-37	1.335555	0.73	0.6	2.45E-33	2 L1 _	Fam162a
Mprip1	4.44E-38	1.117017	0.777	0.71	8.94E-34	2 L1 _	Mprip
Adap21	4.59E-39	1.300829	0.507	0.218	9.25E-35	2 L1 _	Adap2
Cryab1	4.21E-39	1.487451	0.69	0.472	8.49E-35	2 L1 _	Cryab
Gcnt22	3.76E-39	1.213208	0.464	0.172	7.57E-35	2 L1 _	Gcnt2
Fry2	3.43E-39	1.245781	0.445	0.158	6.91E-35	2 L1 _	Fry
Trpc61	4.00E-40	0.840194	0.248	0.043	8.06E-36	2 L1 _	Trpc6
Inka2	1.67E-40	0.700112	0.186	0.021	3.36E-36	2 L1 _	Inka2
Cpm1	9.07E-41	0.947509	0.354	0.084	1.83E-36	2 L1 _	Cpm
Slc2a41	1.97E-41	0.872719	0.31	0.067	3.96E-37	2 L1 _	Slc2a4
Aspn1	1.12E-41	1.327849	0.737	0.492	2.26E-37	2 L1 _	Aspn
Cpe1	4.76E-42	1.219135	0.69	0.415	9.59E-38	2 L1 _	Cpe
Pde3a1	4.29E-43	1.081932	0.409	0.117	8.63E-39	2 L1 _	Pde3a
Crip11	3.13E-43	1.093175	0.956	0.95	6.31E-39	2 L1 _	Crip1
Parm11	1.71E-43	1.130064	0.365	0.094	3.45E-39	2 L1 _	Parm1
Nr2f21	2.60E-44	1.18306	0.821	0.664	5.23E-40	2 L1 _	Nr2f2
Atp1b21	1.28E-44	1.372569	0.573	0.259	2.59E-40	2 L1 _	Atp1b2
Asap21	8.87E-45	1.400089	0.551	0.251	1.79E-40	2 L1 _	Asap2
Mnd11	3.71E-45	1.096717	0.383	0.096	7.47E-41	2 L1 _	Mnd1
Colec111	1.82E-46	1.123533	0.252	0.036	3.67E-42	2 L1 _	Colec11
Meg31	6.02E-47	1.484149	0.858	0.623	1.21E-42	2 L1 _	Meg3
Inpp4b2	2.69E-47	1.316196	0.595	0.247	5.42E-43	2 L1 _	Inpp4b
Sep-71	1.11E-47	0.980769	0.894	0.901	2.24E-43	2 L1 _	7-Sep
Rarres22	2.52E-48	1.113634	0.869	0.667	5.08E-44	2 L1 _	Rarres2
Hspb21	1.61E-48	1.030651	0.35	0.076	3.25E-44	2 L1 _	Hspb2
Serpine21	1.27E-48	1.41022	0.788	0.509	2.56E-44	2 L1 _	Serpine2
Gm138611	5.37E-49	0.853735	0.255	0.034	1.08E-44	2 L1 _	Gm13861
Foxs11	1.63E-49	1.422809	0.672	0.376	3.28E-45	2 L1 _	Foxs1
Tm4sf12	1.05E-50	1.247917	0.876	0.672	2.11E-46	2 L1 _	Tm4sf1
Myh111	1.77E-51	1.351531	0.387	0.082	3.56E-47	2 L1 _	Myh11
H3f3b2	4.16E-53	0.793504	0.978	0.984	8.38E-49	2 L1 _	H3f3b
Ephx31	1.21E-54	1.148026	0.347	0.061	2.44E-50	2 L1 _	Ephx3
Filip1l1	1.08E-56	1.60921	0.639	0.268	2.17E-52	2 L1 _	Filip1l
Lgi11	5.03E-57	1.21579	0.398	0.078	1.01E-52	2 L1 _	Lgi1
Fermt21	1.11E-57	1.175775	0.891	0.76	2.24E-53	2 L1 _	Fermt2
Stac1	4.11E-58	0.899385	0.255	0.027	8.28E-54	2 L1 _	Stac
Dbndd2	2.78E-58	1.497064	0.591	0.239	5.61E-54	2 L1 _	Dbndd2
Trarg1	5.07E-60	0.6519	0.19	0.01	1.02E-55	2 L1 _	Trarg1
Rbpms1	4.04E-61	1.463106	0.839	0.704	8.14E-57	2 L1 _	Rbpms
Gjc11	2.52E-62	1.658049	0.737	0.41	5.08E-58	2 L1 _	Gjc1
Cald11	7.67E-63	1.136737	0.964	0.862	1.55E-58	2 L1 _	Cald1

Rasgrp21	1.58E-63	1.318788	0.62	0.21	3.19E-59	2 L1 _	Rasgrp2
Art31	7.93E-64	1.421863	0.474	0.107	1.60E-59	2 L1 _	Art3
Lmod11	3.86E-66	1.184108	0.383	0.062	7.77E-62	2 L1 _	Lmod1
Tpm11	3.96E-69	1.821895	0.872	0.703	7.97E-65	2 L1 _	Tpm1
Gng112	4.34E-70	1.371329	0.931	0.806	8.74E-66	2 L1 _	Gng11
Tpm21	6.18E-71	1.419255	0.77	0.357	1.25E-66	2 L1 _	Tpm2
Ano11	1.07E-71	1.253552	0.401	0.063	2.15E-67	2 L1 _	Ano1
Cystm11	1.58E-73	1.554703	0.697	0.27	3.17E-69	2 L1 _	Cystm1
Pde5a1	2.53E-75	1.537095	0.602	0.17	5.10E-71	2 L1 _	Pde5a
Phlda11	1.39E-75	2.006173	0.755	0.376	2.81E-71	2 L1 _	Phlda1
Slc12a21	6.04E-76	1.687003	0.679	0.226	1.22E-71	2 L1 _	Slc12a2
Serpini11	7.07E-78	1.477489	0.526	0.11	1.42E-73	2 L1 _	Serpini1
Zeb21	5.77E-80	1.816584	0.898	0.675	1.16E-75	2 L1 _	Zeb2
Ifitm11	4.09E-80	2.320677	0.599	0.169	8.24E-76	2 L1 _	Ifitm1
Ntm1	5.68E-81	1.126883	0.31	0.026	1.14E-76	2 L1 _	Ntm
Ddit4l1	4.13E-83	1.317621	0.383	0.047	8.32E-79	2 L1 _	Ddit4l
Rem11	1.31E-84	1.660565	0.547	0.119	2.64E-80	2 L1 _	Rem1
Mfge81	2.21E-86	1.594787	0.953	0.784	4.46E-82	2 L1 _	Mfge8
Rasl11a1	5.94E-87	1.705126	0.642	0.178	1.20E-82	2 L1 _	Rasl11a
Des1	1.74E-89	1.740653	0.657	0.179	3.51E-85	2 L1 _	Des
Sparcl1	1.19E-91	1.41712	0.978	0.861	2.39E-87	2 L1 _	Sparcl1
Sep-41	9.14E-92	1.82456	0.912	0.693	1.84E-87	2 L1 _	4-Sep
Steap42	1.65E-92	1.909487	0.861	0.397	3.32E-88	2 L1 _	Steap4
Itga71	3.47E-93	1.541433	0.511	0.085	6.98E-89	2 L1 _	Itga7
Ebf11	4.95E-95	1.803943	0.967	0.767	9.98E-91	2 L1 _	Ebf1
Malat11	5.82E-101	1.275969	1	0.997	1.17E-96	2 L1 _	Malat1
Ednrb1	3.47E-101	1.6892	0.836	0.248	7.00E-97	2 L1 _	Ednrb
Abcc91	3.44E-101	1.974554	0.81	0.272	6.93E-97	2 L1 _	Abcc9
Myo1b1	1.59E-101	2.077292	0.872	0.452	3.20E-97	2 L1 _	Myo1b
Ndufa4l21	7.72E-106	2.143224	0.942	0.578	1.55E-101	2 L1 _	Ndufa4l2
Sncg1	8.80E-107	2.044359	0.704	0.166	1.77E-102	2 L1 _	Sncg
Gm138891	2.95E-108	2.48647	0.905	0.446	5.95E-104	2 L1 _	Gm13889
Pdgfrb1	8.74E-110	1.821479	0.996	0.814	1.76E-105	2 L1 _	Pdgfrb
Vtn1	2.83E-112	2.163241	0.701	0.164	5.70E-108	2 L1 _	Vtn
Rasl121	1.08E-113	1.536218	0.54	0.074	2.17E-109	2 L1 _	Rasl12
Myl91	5.62E-118	2.319601	0.88	0.307	1.13E-113	2 L1 _	Myl9
Tinagl12	1.21E-122	1.950837	0.865	0.236	2.44E-118	2 L1 _	Tinagl1
Rgs41	6.15E-123	1.861772	0.624	0.093	1.24E-118	2 L1 _	Rgs4
Gucy1b11	5.61E-125	2.274866	0.865	0.3	1.13E-120	2 L1 _	Gucy1b1
Cox4i21	8.46E-129	1.69644	0.69	0.107	1.70E-124	2 L1 _	Cox4i2
Gja41	9.46E-134	2.210867	0.803	0.182	1.91E-129	2 L1 _	Gja4

Kcnj81	9.28E-141	2.518904	0.814	0.177	1.87E-136	2 L1 _	Kcnj8
Olfr5581	2.09E-146	1.92119	0.635	0.077	4.20E-142	2 L1 _	Olfr558
Carmn1	1.61E-146	2.219002	0.748	0.139	3.25E-142	2 L1 _	Carmn
Gucy1a11	3.94E-148	2.502234	0.938	0.312	7.93E-144	2 L1 _	Gucy1a1
Notch31	4.76E-159	2.18987	0.865	0.17	9.59E-155	2 L1 _	Notch3
Higd1b1	1.89E-194	2.417761	0.85	0.108	3.80E-190	2 L1 _	Higd1b
Rgs51	6.84E-221	3.184942	0.993	0.162	1.38E-216	2 L1 _	Rgs5
St5	2.50E-06	0.468634	0.445	0.308	0.050281	2 M1 _	St5
Nptn	2.36E-06	0.543915	0.68	0.601	0.047626	2 M1 _	Nptn
Sipa1	2.27E-06	0.514971	0.539	0.425	0.045703	2 M1 _	Sipa1
Uqcrq	2.11E-06	0.400118	0.836	0.77	0.042407	2 M1 _	Uqcrq
Rhoj1	1.81E-06	0.379553	0.852	0.736	0.036503	2 M1 _	Rhoj
Cox7a2	1.73E-06	0.385774	0.875	0.852	0.034774	2 M1 _	Cox7a2
Nbr1	1.62E-06	0.539349	0.461	0.333	0.032656	2 M1 _	Nbr1
Armcx3	1.54E-06	0.621644	0.492	0.368	0.031023	2 M1 _	Armcx3
Smtn	1.51E-06	0.641734	0.43	0.306	0.030384	2 M1 _	Smtn
Ndufs7	1.38E-06	0.389612	0.734	0.681	0.027823	2 M1 _	Ndufs7
Fam110b	1.35E-06	0.351871	0.188	0.075	0.027165	2 M1 _	Fam110b
9930111J21Rik2	1.22E-06	0.574695	0.562	0.435	0.024533	2 M1 _	9930111J21Rik2
Mphosph6	1.12E-06	0.553222	0.352	0.207	0.022579	2 M1 _	Mphosph6
Maged21	1.04E-06	0.636805	0.555	0.43	0.020994	2 M1 _	Maged2
Limk1	9.86E-07	0.543677	0.281	0.146	0.019851	2 M1 _	Limk1
Taf6l1	9.75E-07	0.368524	0.203	0.085	0.019628	2 M1 _	Taf6l
Maml3	9.66E-07	0.547692	0.438	0.292	0.019455	2 M1 _	Maml3
Sytl2	9.41E-07	0.343301	0.188	0.072	0.018957	2 M1 _	Sytl2
Bcl9l	9.40E-07	0.491532	0.453	0.307	0.018939	2 M1 _	Bcl9l
Ndufc1	8.95E-07	0.443289	0.75	0.707	0.018021	2 M1 _	Ndufc1
C2cd2	8.66E-07	0.501761	0.344	0.194	0.01744	2 M1 _	C2cd2
Olfr1033	8.64E-07	0.409125	0.188	0.075	0.017409	2 M1 _	Olfr1033
Arhgap26	8.57E-07	0.265992	0.18	0.067	0.017263	2 M1 _	Arhgap26
Ttll7	7.97E-07	0.372612	0.219	0.091	0.016051	2 M1 _	Ttll7
Cox7c	7.76E-07	0.321721	0.945	0.908	0.015619	2 M1 _	Cox7c
Pygm	7.30E-07	0.435649	0.305	0.157	0.01471	2 M1 _	Pygm
Pcdh18	7.29E-07	0.577001	0.586	0.44	0.014679	2 M1 _	Pcdh18
Nr2f11	7.15E-07	0.497689	0.734	0.593	0.014407	2 M1 _	Nr2f1
Timm17a	6.54E-07	0.490886	0.523	0.392	0.013164	2 M1 _	Timm17a
Plxna1	6.19E-07	0.381158	0.352	0.195	0.012466	2 M1 _	Plxna1
Phlda3	6.19E-07	0.426136	0.719	0.547	0.012458	2 M1 _	Phlda3
Dynlt3	6.17E-07	0.55347	0.633	0.526	0.012431	2 M1 _	Dynlt3
Soga1	5.95E-07	0.427698	0.211	0.09	0.011987	2 M1 _	Soga1
Rsbn1l	5.28E-07	0.456038	0.617	0.492	0.01064	2 M1 _	Rsbn1l

Cnnm2	5.08E-07	0.352802	0.18	0.067	0.010233	2 M1 _	Cnnm2
Ybey	4.84E-07	0.293224	0.133	0.04	0.009752	2 M1 _	Ybey
Gm12840	4.79E-07	0.752991	0.18	0.069	0.009656	2 M1 _	Gm12840
Phtf2	4.67E-07	0.335928	0.188	0.072	0.009407	2 M1 _	Phtf2
Fxyd2	4.54E-07	0.583645	0.375	0.216	0.009151	2 M1 _	Fxyd2
Specc1	3.94E-07	0.446996	0.328	0.174	0.007941	2 M1 _	Specc1
Rheb	3.54E-07	0.52087	0.711	0.639	0.007132	2 M1 _	Rheb
Slc25a3	3.44E-07	0.475373	0.867	0.84	0.006933	2 M1 _	Slc25a3
Camk1	3.39E-07	0.516367	0.586	0.467	0.006829	2 M1 _	Camk1
Mark1	3.29E-07	0.522849	0.359	0.208	0.006633	2 M1 _	Mark1
Cyp4b1	2.88E-07	0.331861	0.156	0.05	0.00581	2 M1 _	Cyp4b1
Trp53inp2	2.88E-07	0.544137	0.312	0.167	0.005795	2 M1 _	Trp53inp2
Spry21	2.20E-07	0.659384	0.375	0.225	0.004425	2 M1 _	Spry2
Tmem571	2.13E-07	0.269056	0.133	0.038	0.004294	2 M1 _	Tmem57
Dag11	2.08E-07	0.534258	0.68	0.587	0.004197	2 M1 _	Dag1
Rsu1	1.86E-07	0.558599	0.688	0.591	0.003752	2 M1 _	Rsu1
mt-Atp6	1.85E-07	0.294823	1	0.993	0.003736	2 M1 _	mt-Atp6
Ankrd50	1.65E-07	0.476523	0.391	0.231	0.003322	2 M1 _	Ankrd50
Dab2ip	1.37E-07	0.541405	0.625	0.49	0.002755	2 M1 _	Dab2ip
Sparc	1.29E-07	0.346003	1	0.918	0.002595	2 M1 _	Sparc
Ppp1r12c	1.16E-07	0.875905	0.422	0.286	0.002344	2 M1 _	Ppp1r12c
Cdc42bpa	1.16E-07	0.65829	0.562	0.423	0.002329	2 M1 _	Cdc42bpa
Grk5	1.13E-07	0.408292	0.391	0.219	0.002281	2 M1 _	Grk5
Map1b	1.09E-07	0.77192	0.414	0.262	0.002199	2 M1 _	Map1b
Uqcrb	1.05E-07	0.383568	0.867	0.822	0.00212	2 M1 _	Uqcrb
Jade11	9.83E-08	0.720555	0.492	0.343	0.00198	2 M1 _	Jade1
Ppp2r1a	9.58E-08	0.578419	0.578	0.47	0.00193	2 M1 _	Ppp2r1a
Anxa11	9.45E-08	0.551818	0.336	0.182	0.001904	2 M1 _	Anxa11
Unc45a	8.97E-08	0.472368	0.328	0.172	0.001807	2 M1 _	Unc45a
Cyp7b1	8.80E-08	0.450849	0.312	0.152	0.001772	2 M1 _	Cyp7b1
Rab14	8.71E-08	0.465184	0.844	0.817	0.001754	2 M1 _	Rab14
Rnf152	8.12E-08	0.265921	0.211	0.077	0.001636	2 M1 _	Rnf152
Fbxo30	8.12E-08	0.410308	0.273	0.125	0.001636	2 M1 _	Fbxo30
Tgfb1i1	7.93E-08	0.602903	0.547	0.411	0.001597	2 M1 _	Tgfb1i1
Enpep	7.51E-08	0.380229	0.32	0.152	0.001512	2 M1 _	Enpep
Crip2	7.10E-08	0.42464	0.867	0.766	0.00143	2 M1 _	Crip2
Rapgef2	7.09E-08	0.633501	0.391	0.22	0.001428	2 M1 _	Rapgef2
Pdha1	6.93E-08	0.562153	0.578	0.432	0.001396	2 M1 _	Pdha1
Vdac1	6.81E-08	0.539186	0.625	0.54	0.001371	2 M1 _	Vdac1
Fxr1	6.58E-08	0.600488	0.656	0.537	0.001326	2 M1 _	Fxr1
Hbb-bs	6.42E-08	1.712328	0.398	0.22	0.001293	2 M1 _	Hbb-bs

Ncor1	6.37E-08	0.428683	0.797	0.753	0.001283	2 M1 _	Ncor1
Fgf1	4.86E-08	0.494234	0.242	0.101	0.000978	2 M1 _	Fgf1
Ntm	4.85E-08	0.337065	0.172	0.053	0.000976	2 M1 _	Ntm
Tnfaip1	4.83E-08	0.460415	0.555	0.372	0.000973	2 M1 _	Tnfaip1
Pmp221	4.58E-08	0.518272	0.844	0.7	0.000923	2 M1 _	Pmp22
Fam174a	4.54E-08	0.692959	0.523	0.399	0.000915	2 M1 _	Fam174a
Camk2n11	4.53E-08	0.72616	0.664	0.538	0.000913	2 M1 _	Camk2n1
Itpr1	4.16E-08	0.648083	0.594	0.472	0.000837	2 M1 _	Itpr1
Gpc6	4.16E-08	0.542353	0.578	0.415	0.000837	2 M1 _	Gpc6
Cnn2	3.94E-08	0.584155	0.586	0.437	0.000793	2 M1 _	Cnn2
Cavin1	3.83E-08	0.998158	0.586	0.478	0.000772	2 M1 _	Cavin1
Eif4e3	3.07E-08	0.508789	0.383	0.215	0.000619	2 M1 _	Eif4e3
Stx8	2.93E-08	0.551418	0.516	0.36	0.00059	2 M1 _	Stx8
Pacsin2	2.90E-08	0.604588	0.438	0.278	0.000583	2 M1 _	Pacsin2
Ggta1	2.78E-08	0.372834	0.281	0.12	0.00056	2 M1 _	Ggta1
Angpt1	2.69E-08	0.559185	0.32	0.157	0.000542	2 M1 _	Angpt1
Kank4	2.65E-08	0.256832	0.148	0.043	0.000534	2 M1 _	Kank4
Tprgl	2.55E-08	0.526886	0.766	0.696	0.000514	2 M1 _	Tprgl
Cux1	2.49E-08	0.61384	0.547	0.393	0.000501	2 M1 _	Cux1
Selenom	2.43E-08	0.405848	0.953	0.816	0.000488	2 M1 _	Selenom
Vdac2	2.39E-08	0.470074	0.766	0.673	0.000482	2 M1 _	Vdac2
Utrn	2.22E-08	0.829355	0.617	0.484	0.000446	2 M1 _	Utrn
Hnrnpa0	2.17E-08	0.502442	0.828	0.733	0.000436	2 M1 _	Hnrnpa0
Spry4	2.13E-08	0.571282	0.43	0.258	0.000429	2 M1 _	Spry4
Gnb4	2.03E-08	0.566536	0.797	0.663	0.000408	2 M1 _	Gnb4
Myh9	1.96E-08	0.548546	0.844	0.828	0.000395	2 M1 _	Myh9
Sult1a1	1.86E-08	0.652329	0.5	0.328	0.000375	2 M1 _	Sult1a1
Plxnb2	1.84E-08	0.564604	0.531	0.374	0.000371	2 M1 _	Plxnb2
C1qtnf11	1.60E-08	0.459548	0.328	0.158	0.000323	2 M1 _	C1qtnf1
Mgat4a	1.59E-08	0.454941	0.25	0.101	0.000321	2 M1 _	Mgat4a
Ptp4a3	1.54E-08	0.996253	0.469	0.319	0.000309	2 M1 _	Ptp4a3
4930523C07Rik	1.47E-08	0.629584	0.578	0.428	0.000297	2 M1 _	4930523C07Rik
Ppargc1b	1.40E-08	0.303726	0.156	0.045	0.000282	2 M1 _	Ppargc1b
Hbegf	1.36E-08	0.339973	0.25	0.097	0.000275	2 M1 _	Hbegf
Ostf1	1.26E-08	0.523472	0.727	0.605	0.000255	2 M1 _	Ostf1
Ilk	1.12E-08	0.563603	0.664	0.576	0.000225	2 M1 _	Ilk
Amotl11	1.01E-08	0.577959	0.742	0.633	0.000203	2 M1 _	Amotl1
Anxa51	1.01E-08	0.463824	0.977	0.882	0.000203	2 M1 _	Anxa5
Ubr1	8.42E-09	0.746829	0.531	0.381	0.00017	2 M1 _	Ubr1
Kitl	8.27E-09	0.473099	0.609	0.398	0.000167	2 M1 _	Kitl
Sox5	7.54E-09	0.383489	0.211	0.072	0.000152	2 M1 _	Sox5

Marveld1	7.00E-09	0.715896	0.5	0.351	0.000141	2 M1 _	Marveld1
Hipk1	6.91E-09	0.698579	0.523	0.367	0.000139	2 M1 _	Hipk1
Rasd1	6.83E-09	0.342498	0.133	0.033	0.000138	2 M1 _	Rasd1
Pfn1	6.74E-09	0.42568	0.883	0.79	0.000136	2 M1 _	Pfn1
Ecm2	6.67E-09	0.706256	0.648	0.5	0.000134	2 M1 _	Ecm2
Adam22	6.66E-09	0.27095	0.141	0.037	0.000134	2 M1 _	Adam22
Fhl11	6.35E-09	0.402074	0.203	0.068	0.000128	2 M1 _	Fhl1
Adcy3	5.54E-09	0.71186	0.391	0.225	0.000112	2 M1 _	Adcy3
Mustn1	5.45E-09	1.095518	0.273	0.113	0.00011	2 M1 _	Mustn1
Eif4ebp2	5.08E-09	0.528325	0.625	0.483	0.000102	2 M1 _	Eif4ebp2
Herc1	4.93E-09	0.6602	0.664	0.536	9.94E-05	2 M1 _	Herc1
Sox13	4.90E-09	0.330504	0.234	0.085	9.88E-05	2 M1 _	Sox13
Eif1b	4.00E-09	0.610261	0.727	0.606	8.05E-05	2 M1 _	Eif1b
Ppp2r3a	3.78E-09	0.553977	0.398	0.216	7.62E-05	2 M1 _	Ppp2r3a
Trib2	3.71E-09	0.815498	0.516	0.346	7.48E-05	2 M1 _	Trib2
Dapk2	3.57E-09	0.594969	0.547	0.359	7.19E-05	2 M1 _	Dapk2
Srsf5	2.72E-09	0.478684	0.836	0.758	5.48E-05	2 M1 _	Srsf5
Msrb1	2.69E-09	0.624863	0.484	0.313	5.42E-05	2 M1 _	Msrb1
Arrdc31	2.49E-09	0.775613	0.398	0.221	5.02E-05	2 M1 _	Arrdc3
Cycs	2.46E-09	0.638536	0.648	0.531	4.96E-05	2 M1 _	Cycs
Atp5k	2.36E-09	0.568271	0.781	0.752	4.74E-05	2 M1 _	Atp5k
Stim1	2.13E-09	0.589514	0.578	0.401	4.30E-05	2 M1 _	Stim1
Fam107b	1.89E-09	0.354072	0.266	0.1	3.81E-05	2 M1 _	Fam107b
Lpcat3	1.72E-09	0.642493	0.492	0.316	3.46E-05	2 M1 _	Lpcat3
Pear1	1.65E-09	0.671022	0.578	0.425	3.33E-05	2 M1 _	Pear1
Map2	1.52E-09	0.482181	0.328	0.145	3.06E-05	2 M1 _	Map2
Dpysl21	1.37E-09	0.724781	0.617	0.471	2.76E-05	2 M1 _	Dpysl2
Aig1	1.35E-09	0.66667	0.406	0.219	2.72E-05	2 M1 _	Aig1
Sdccag31	1.20E-09	0.334167	0.156	0.042	2.41E-05	2 M1 _	Sdccag3
Cox6c	1.16E-09	0.453576	0.977	0.923	2.34E-05	2 M1 _	Cox6c
Ppp1r3d	1.10E-09	0.297511	0.148	0.037	2.22E-05	2 M1 _	Ppp1r3d
Ralgps1	9.94E-10	0.313226	0.164	0.044	2.00E-05	2 M1 _	Ralgps1
Suco	9.16E-10	0.810698	0.461	0.272	1.85E-05	2 M1 _	Suco
Gpcpd1	9.16E-10	0.462984	0.352	0.167	1.84E-05	2 M1 _	Gpcpd1
Epb41	8.95E-10	0.500348	0.359	0.169	1.80E-05	2 M1 _	Epb41
Tnfaip8	8.21E-10	0.630682	0.625	0.441	1.65E-05	2 M1 _	Tnfaip8
Klhdc8b	8.19E-10	0.479522	0.375	0.18	1.65E-05	2 M1 _	Klhdc8b
Calm1	6.42E-10	0.427623	0.977	0.95	1.29E-05	2 M1 _	Calm1
Tead1	6.37E-10	0.65964	0.531	0.357	1.28E-05	2 M1 _	Tead1
Ywhab	6.21E-10	0.526862	0.867	0.832	1.25E-05	2 M1 _	Ywhab
Gcn1l11	6.05E-10	0.381237	0.156	0.041	1.22E-05	2 M1 _	Gcn1l1

Map3k20	5.89E-10	1.038977	0.469	0.295	1.19E-05	2 M1 _	Map3k20
Nmt1	5.87E-10	0.650625	0.75	0.659	1.18E-05	2 M1 _	Nmt1
Snrpn	5.84E-10	0.325501	0.141	0.034	1.18E-05	2 M1 _	Snrpn
Ccnd1	5.08E-10	1.127652	0.359	0.167	1.02E-05	2 M1 _	Ccnd1
Dyrk2	4.44E-10	0.582768	0.391	0.202	8.95E-06	2 M1 _	Dyrk2
Sema6d	4.27E-10	0.607882	0.562	0.347	8.60E-06	2 M1 _	Sema6d
Ppm1l	4.21E-10	0.489914	0.164	0.044	8.49E-06	2 M1 _	Ppm1l
Uqcr11	4.20E-10	0.514505	0.82	0.808	8.46E-06	2 M1 _	Uqcr11
Lamb2	2.97E-10	0.747207	0.625	0.511	5.97E-06	2 M1 _	Lamb2
Tuba1b1	2.88E-10	0.521374	0.867	0.82	5.80E-06	2 M1 _	Tuba1b
Filip1	2.86E-10	0.618033	0.383	0.184	5.77E-06	2 M1 _	Filip1
Pcp4l1	2.67E-10	0.70433	0.445	0.214	5.38E-06	2 M1 _	Pcp4l1
Dmpk1	2.57E-10	0.538741	0.273	0.106	5.17E-06	2 M1 _	Dmpk
Lims1	2.43E-10	0.662419	0.656	0.548	4.90E-06	2 M1 _	Lims1
Ptp4a11	2.11E-10	0.324467	0.211	0.065	4.24E-06	2 M1 _	Ptp4a1
Gapdh	2.03E-10	0.497748	0.953	0.905	4.09E-06	2 M1 _	Gapdh
Bok	1.78E-10	0.418461	0.336	0.138	3.58E-06	2 M1 _	Bok
Stk38l	1.67E-10	0.452875	0.32	0.13	3.37E-06	2 M1 _	Stk38l
Prdx1	1.64E-10	0.450863	0.977	0.906	3.31E-06	2 M1 _	Prdx1
Tmco1	1.37E-10	0.66276	0.797	0.721	2.76E-06	2 M1 _	Tmco1
Dlc1	1.32E-10	0.478515	0.836	0.698	2.67E-06	2 M1 _	Dlc1
Rcsd1	1.26E-10	0.447358	0.438	0.214	2.54E-06	2 M1 _	Rcsd1
Cd811	1.25E-10	0.574721	0.992	0.891	2.51E-06	2 M1 _	Cd81
Smpdl3a	1.17E-10	0.795064	0.633	0.472	2.36E-06	2 M1 _	Smpdl3a
Etfb	9.82E-11	0.582395	0.703	0.562	1.98E-06	2 M1 _	Etfb
Ktn1	7.38E-11	0.663149	0.734	0.62	1.49E-06	2 M1 _	Ktn1
Ndufs2	7.16E-11	0.821965	0.68	0.556	1.44E-06	2 M1 _	Ndufs2
Smagp	5.47E-11	0.593746	0.344	0.142	1.10E-06	2 M1 _	Smagp
A230050P20Rik1	5.25E-11	0.33289	0.125	0.025	1.06E-06	2 M1 _	A230050P20Rik
Gnas1	5.04E-11	0.39407	1	0.959	1.01E-06	2 M1 _	Gnas
Rap1a	4.59E-11	0.640751	0.734	0.649	9.24E-07	2 M1 _	Rap1a
S1pr31	4.49E-11	0.785937	0.469	0.281	9.05E-07	2 M1 _	S1pr3
Kcnab1	4.47E-11	0.382482	0.109	0.019	9.01E-07	2 M1 _	Kcnab1
Ephx2	4.12E-11	0.284814	0.164	0.04	8.31E-07	2 M1 _	Ephx2
Mgea51	4.03E-11	0.338736	0.188	0.051	8.12E-07	2 M1 _	Mgea5
Slc11a1	3.89E-11	0.426891	0.156	0.036	7.82E-07	2 M1 _	Slc11a1
Nedd41	3.81E-11	0.486813	0.977	0.884	7.67E-07	2 M1 _	Nedd4
Asap2	3.68E-11	0.52387	0.508	0.274	7.42E-07	2 M1 _	Asap2
Tns3	3.44E-11	0.613546	0.461	0.257	6.92E-07	2 M1 _	Tns3
Papd41	3.02E-11	0.279337	0.156	0.037	6.08E-07	2 M1 _	Papd4
Ate1	2.93E-11	0.751362	0.586	0.414	5.90E-07	2 M1 _	Ate1

Nab1	2.83E-11	0.889189	0.531	0.348	5.71E-07	2 M1 _	Nab1
Ptk2	2.64E-11	0.640595	0.555	0.34	5.32E-07	2 M1 _	Ptk2
Celf1	2.62E-11	0.637815	0.594	0.423	5.28E-07	2 M1 _	Celf1
4933431E20Rik	2.50E-11	0.43872	0.133	0.027	5.04E-07	2 M1 _	4933431E20Rik
Ptpn9	2.46E-11	0.793761	0.594	0.435	4.95E-07	2 M1 _	Ptpn9
Peli2	2.43E-11	0.617975	0.359	0.16	4.89E-07	2 M1 _	Peli2
Mbnl2	2.42E-11	0.644131	0.805	0.697	4.86E-07	2 M1 _	Mbnl2
Pik3r11	2.40E-11	0.634372	0.781	0.659	4.84E-07	2 M1 _	Pik3r1
Cfl2	2.21E-11	0.71484	0.602	0.428	4.44E-07	2 M1 _	Cfl2
Chtf81	2.20E-11	0.409582	0.25	0.083	4.43E-07	2 M1 _	Chtf8
Rhou	2.02E-11	0.826078	0.312	0.133	4.06E-07	2 M1 _	Rhou
Mef2c	1.85E-11	0.436417	0.852	0.64	3.72E-07	2 M1 _	Mef2c
Il34	1.76E-11	0.570851	0.766	0.482	3.54E-07	2 M1 _	Il34
9030624J02Rik1	1.69E-11	0.394857	0.164	0.039	3.41E-07	2 M1 _	9030624J02Rik
Pgrmc1	1.65E-11	0.660137	0.711	0.54	3.32E-07	2 M1 _	Pgrmc1
Slc4a3	1.60E-11	0.474725	0.234	0.075	3.22E-07	2 M1 _	Slc4a3
Pde4d	1.51E-11	0.393202	0.203	0.057	3.03E-07	2 M1 _	Pde4d
Fry	1.36E-11	0.600541	0.406	0.179	2.75E-07	2 M1 _	Fry
Epb41l21	1.36E-11	0.611475	0.797	0.673	2.73E-07	2 M1 _	Epb41l2
Ndufb9	1.06E-11	0.573541	0.906	0.822	2.14E-07	2 M1 _	Ndufb9
Prkar1b	1.03E-11	0.444703	0.188	0.048	2.07E-07	2 M1 _	Prkar1b
Hsp90aa1	9.47E-12	0.644593	0.828	0.736	1.91E-07	2 M1 _	Hsp90aa1
Cdc42ep4	9.35E-12	0.633793	0.469	0.261	1.88E-07	2 M1 _	Cdc42ep4
Epha2	7.87E-12	0.313338	0.188	0.048	1.59E-07	2 M1 _	Epha2
Naf1	7.80E-12	0.636758	0.391	0.184	1.57E-07	2 M1 _	Naf1
Fam124a	7.78E-12	0.323124	0.18	0.044	1.57E-07	2 M1 _	Fam124a
Stbd1	6.51E-12	0.70434	0.234	0.073	1.31E-07	2 M1 _	Stbd1
Syn3	5.53E-12	0.497598	0.289	0.101	1.11E-07	2 M1 _	Syn3
Rbm8a	5.01E-12	0.685589	0.734	0.574	1.01E-07	2 M1 _	Rbm8a
Cyr611	4.64E-12	0.332079	0.148	0.031	9.35E-08	2 M1 _	Cyr61
Svopl	4.40E-12	0.53578	0.172	0.041	8.86E-08	2 M1 _	Svopl
Slc25a33	4.24E-12	0.389254	0.219	0.062	8.54E-08	2 M1 _	Slc25a33
Pik3ca	3.92E-12	0.817988	0.562	0.365	7.90E-08	2 M1 _	Pik3ca
Trpc3	3.61E-12	0.337253	0.164	0.036	7.27E-08	2 M1 _	Trpc3
Anxa3	3.37E-12	0.46027	0.578	0.301	6.80E-08	2 M1 _	Anxa3
Arhgef16	3.11E-12	0.272867	0.148	0.029	6.26E-08	2 M1 _	Arhgef16
Rab8b	2.94E-12	0.706986	0.57	0.349	5.92E-08	2 M1 _	Rab8b
Rras	2.89E-12	0.724435	0.773	0.673	5.81E-08	2 M1 _	Rras
Arap2	2.87E-12	0.766147	0.602	0.39	5.78E-08	2 M1 _	Arap2
Adcyap1r11	2.81E-12	0.886086	0.484	0.274	5.65E-08	2 M1 _	Adcyap1r1
Gnai1	2.50E-12	0.461464	0.211	0.058	5.04E-08	2 M1 _	Gnai1

Trpc1	2.45E-12	0.540451	0.305	0.11	4.93E-08	2 M1 _	Trpc1
Pdcd10	2.26E-12	0.707175	0.719	0.57	4.54E-08	2 M1 _	Pdcd10
Suclg1	2.07E-12	0.652917	0.688	0.526	4.16E-08	2 M1 _	Suclg1
Sh3bgrl	1.70E-12	0.805705	0.75	0.683	3.43E-08	2 M1 _	Sh3bgrl
Papd5	1.64E-12	0.337796	0.133	0.024	3.30E-08	2 M1 _	Papd5
1110004E09Rik	1.22E-12	0.312119	0.148	0.03	2.46E-08	2 M1 _	1110004E09Rik
Tpm4	1.18E-12	0.791759	0.844	0.751	2.39E-08	2 M1 _	Tpm4
Meox2	1.11E-12	0.499548	0.383	0.154	2.23E-08	2 M1 _	Meox2
Dusp11	1.09E-12	0.755547	0.562	0.326	2.19E-08	2 M1 _	Dusp1
Tmem51	1.02E-12	0.495142	0.258	0.08	2.05E-08	2 M1 _	Tmem51
Col4a11	7.69E-13	0.634426	0.992	0.866	1.55E-08	2 M1 _	Col4a1
Stac	7.22E-13	0.325923	0.195	0.046	1.45E-08	2 M1 _	Stac
Tspan3	7.11E-13	0.659639	0.828	0.659	1.43E-08	2 M1 _	Tspan3
Rock2	6.37E-13	0.811271	0.766	0.665	1.28E-08	2 M1 _	Rock2
Atp2b4	6.09E-13	0.802786	0.531	0.301	1.23E-08	2 M1 _	Atp2b4
Fam110a	5.67E-13	0.438091	0.258	0.079	1.14E-08	2 M1 _	Fam110a
0610009O20Rik1	5.30E-13	0.255544	0.156	0.032	1.07E-08	2 M1 _	0610009O20Rik
Daam1	5.25E-13	0.702346	0.594	0.367	1.06E-08	2 M1 _	Daam1
Snrnp27	5.22E-13	0.764444	0.695	0.517	1.05E-08	2 M1 _	Snrnp27
Nbeal1	4.84E-13	0.696045	0.758	0.624	9.74E-09	2 M1 _	Nbeal1
Kdelc21	4.81E-13	0.418497	0.234	0.066	9.68E-09	2 M1 _	Kdelc2
Cav2	4.52E-13	0.501916	0.453	0.194	9.10E-09	2 M1 _	Cav2
Ech1	4.26E-13	0.689106	0.781	0.621	8.58E-09	2 M1 _	Ech1
Hgf	3.85E-13	0.689151	0.328	0.125	7.76E-09	2 M1 _	Hgf
Kank1	3.16E-13	0.552348	0.312	0.114	6.37E-09	2 M1 _	Kank1
Ptprf	2.66E-13	0.364156	0.25	0.073	5.36E-09	2 M1 _	Ptprf
Zfhx3	1.85E-13	1.055904	0.492	0.265	3.73E-09	2 M1 _	Zfhx3
Spag71	1.78E-13	0.61887	0.391	0.172	3.59E-09	2 M1 _	Spag7
Clstn1	1.76E-13	0.744922	0.742	0.56	3.54E-09	2 M1 _	Clstn1
Akap2	1.64E-13	0.30909	0.125	0.02	3.30E-09	2 M1 _	Akap2
Atp9a	1.23E-13	0.573735	0.281	0.091	2.47E-09	2 M1 _	Atp9a
Sntb1	1.19E-13	0.380863	0.172	0.036	2.40E-09	2 M1 _	Sntb1
Pkig	1.08E-13	0.668494	0.789	0.611	2.18E-09	2 M1 _	Pkig
Vasp	7.23E-14	0.824294	0.648	0.445	1.46E-09	2 M1 _	Vasp
Ubb	6.88E-14	0.422361	1	0.978	1.38E-09	2 M1 _	Ubb
Hrc	6.39E-14	0.271885	0.141	0.024	1.29E-09	2 M1 _	Hrc
Afap1l2	5.39E-14	0.567276	0.43	0.188	1.09E-09	2 M1 _	Afap1l2
Pacsin3	4.43E-14	0.684487	0.312	0.108	8.93E-10	2 M1 _	Pacsin3
Plekha2	3.80E-14	0.758696	0.445	0.212	7.66E-10	2 M1 _	Plekha2
Adora2a	3.60E-14	0.420812	0.234	0.062	7.25E-10	2 M1 _	Adora2a
Apold1	3.53E-14	0.42421	0.258	0.072	7.12E-10	2 M1 _	Apold1

Fabp5	3.49E-14	0.850912	0.5	0.239	7.03E-10	2 M1 _	Fabp5
Chchd2	3.29E-14	0.436797	0.984	0.96	6.63E-10	2 M1 _	Chchd2
Ggt51	3.10E-14	0.808192	0.609	0.349	6.24E-10	2 M1 _	Ggt5
Wtip	2.86E-14	1.230742	0.539	0.334	5.76E-10	2 M1 _	Wtip
Atp5g3	2.24E-14	0.759621	0.797	0.689	4.51E-10	2 M1 _	Atp5g3
Selenow	1.85E-14	0.601273	0.969	0.889	3.73E-10	2 M1 _	Selenow
Rgs7bp1	1.45E-14	0.928509	0.375	0.151	2.92E-10	2 M1 _	Rgs7bp
Bdnf	1.44E-14	0.326003	0.109	0.014	2.90E-10	2 M1 _	Bdnf
Cds2	1.35E-14	0.664039	0.547	0.292	2.73E-10	2 M1 _	Cds2
Plxdc1	1.26E-14	0.334808	0.164	0.03	2.54E-10	2 M1 _	Plxdc1
Hspb1	1.20E-14	0.843266	0.797	0.592	2.41E-10	2 M1 _	Hspb1
Dopey11	1.09E-14	0.382292	0.203	0.046	2.20E-10	2 M1 _	Dopey1
11-Sep	7.38E-15	0.699197	0.859	0.735	1.49E-10	2 M1 _	11-Sep
Plce1	5.55E-15	0.681001	0.445	0.194	1.12E-10	2 M1 _	Plce1
Grk3	5.47E-15	0.460183	0.242	0.062	1.10E-10	2 M1 _	Grk3
Gm13470	4.64E-15	0.394126	0.18	0.036	9.34E-11	2 M1 _	Gm13470
Rbm24	4.14E-15	0.261212	0.102	0.011	8.34E-11	2 M1 _	Rbm24
D17Wsu92e1	3.83E-15	0.471783	0.219	0.053	7.70E-11	2 M1 _	D17Wsu92e
Fam26e1	3.68E-15	0.26073	0.133	0.02	7.42E-11	2 M1 _	Fam26e
Ptges3l	3.31E-15	0.367352	0.172	0.034	6.66E-11	2 M1 _	Ptges3l
Prkg1	3.27E-15	0.781952	0.438	0.185	6.58E-11	2 M1 _	Prkg1
Anxa1	2.25E-15	0.908912	0.75	0.564	4.53E-11	2 M1 _	Anxa1
Mkl2	2.17E-15	0.320011	0.211	0.047	4.37E-11	2 M1 _	Mkl2
Ifitm1	2.04E-15	1.037288	0.484	0.204	4.11E-11	2 M1 _	Ifitm1
Aldh21	1.99E-15	0.698509	0.891	0.751	4.01E-11	2 M1 _	Aldh2
Prune2	1.98E-15	0.376634	0.109	0.013	4.00E-11	2 M1 _	Prune2
Sep-81	1.76E-15	0.962585	0.594	0.381	3.55E-11	2 M1 _	8-Sep
Ndufs5	1.63E-15	0.619141	0.875	0.778	3.28E-11	2 M1 _	Ndufs5
Tm4sf1	9.35E-16	0.552553	0.891	0.685	1.88E-11	2 M1 _	Tm4sf1
Msn	7.66E-16	0.574318	0.945	0.789	1.54E-11	2 M1 _	Msn
Nr2f2	7.39E-16	0.73299	0.852	0.673	1.49E-11	2 M1 _	Nr2f2
Tspan2	5.74E-16	0.447079	0.227	0.053	1.16E-11	2 M1 _	Tspan2
Snx181	5.67E-16	0.84221	0.648	0.404	1.14E-11	2 M1 _	Snx18
Angpt2	4.11E-16	0.484292	0.289	0.079	8.28E-12	2 M1 _	Angpt2
AU0198231	3.92E-16	0.445615	0.18	0.034	7.90E-12	2 M1 _	AU019823
Arhgef25	3.29E-16	0.683658	0.625	0.367	6.63E-12	2 M1 _	Arhgef25
Epb41l1	3.05E-16	0.759277	0.414	0.169	6.15E-12	2 M1 _	Epb41l1
Cited2	2.71E-16	0.819245	0.555	0.271	5.46E-12	2 M1 _	Cited2
Jag1	2.59E-16	0.741342	0.516	0.247	5.21E-12	2 M1 _	Jag1
Kcnq4	2.39E-16	0.439459	0.234	0.055	4.81E-12	2 M1 _	Kcnq4
Cend1	2.00E-16	0.32819	0.117	0.014	4.04E-12	2 M1 _	Cend1

Tln1	1.72E-16	0.676666	0.867	0.812	3.47E-12	2 M1 _	Tln1
Plcl1	1.66E-16	0.660464	0.383	0.137	3.33E-12	2 M1 _	Plcl1
0610037L13Rik1	1.38E-16	0.416831	0.211	0.045	2.78E-12	2 M1 _	0610037L13Rik
2410089E03Rik1	1.09E-16	0.494566	0.172	0.031	2.19E-12	2 M1 _	2410089E03Rik
Olfr78	1.01E-16	0.347171	0.117	0.014	2.03E-12	2 M1 _	Olfr78
Smarcd3	8.83E-17	0.560849	0.289	0.081	1.78E-12	2 M1 _	Smarcd3
Stard8	8.74E-17	0.845035	0.477	0.201	1.76E-12	2 M1 _	Stard8
Ppp1r12b	7.12E-17	1.010283	0.445	0.188	1.43E-12	2 M1 _	Ppp1r12b
Fahd1	6.76E-17	0.723711	0.375	0.134	1.36E-12	2 M1 _	Fahd1
Pip5k1b	6.35E-17	0.47003	0.195	0.038	1.28E-12	2 M1 _	Pip5k1b
Cadm3	6.14E-17	0.564195	0.227	0.051	1.24E-12	2 M1 _	Cadm3
Il2ra	4.91E-17	0.444933	0.242	0.055	9.88E-13	2 M1 _	Il2ra
4931406P16Rik	4.26E-17	0.977189	0.688	0.433	8.57E-13	2 M1 _	4931406P16Rik
Tubb4b	4.10E-17	0.766312	0.867	0.693	8.25E-13	2 M1 _	Tubb4b
Nes	3.98E-17	0.78215	0.516	0.216	8.02E-13	2 M1 _	Nes
Ldlrap1	3.78E-17	0.738898	0.453	0.191	7.62E-13	2 M1 _	Ldlrap1
Fam58b1	3.44E-17	0.428603	0.18	0.033	6.93E-13	2 M1 _	Fam58b
Pawr	3.17E-17	0.690793	0.375	0.127	6.37E-13	2 M1 _	Pawr
Zufsp1	2.78E-17	0.437447	0.172	0.029	5.59E-13	2 M1 _	Zufsp
Gstm11	2.50E-17	1.055422	0.859	0.655	5.03E-13	2 M1 _	Gstm1
Laptm4a	2.11E-17	0.706803	1	0.922	4.24E-13	2 M1 _	Laptm4a
Gstm5	1.97E-17	1.314971	0.609	0.37	3.97E-13	2 M1 _	Gstm5
Gm26699	1.80E-17	0.373136	0.109	0.011	3.62E-13	2 M1 _	Gm26699
Gamt	1.77E-17	0.715816	0.547	0.255	3.56E-13	2 M1 _	Gamt
Grm7	1.14E-17	0.312988	0.133	0.016	2.30E-13	2 M1 _	Grm7
Zfp467	9.96E-18	0.698487	0.453	0.171	2.01E-13	2 M1 _	Zfp467
Uba2	8.95E-18	0.971246	0.648	0.422	1.80E-13	2 M1 _	Uba2
Bmp2	8.25E-18	0.690368	0.289	0.079	1.66E-13	2 M1 _	Bmp2
Cdh2	8.12E-18	0.499157	0.211	0.041	1.63E-13	2 M1 _	Cdh2
Bmp5	7.44E-18	0.365032	0.141	0.019	1.50E-13	2 M1 _	Bmp5
Ddit4l	6.59E-18	0.486948	0.297	0.074	1.33E-13	2 M1 _	Ddit4l
Nxf11	4.92E-18	0.860135	0.539	0.261	9.91E-14	2 M1 _	Nxf1
mt-Nd2	4.91E-18	0.518629	0.992	0.98	9.88E-14	2 M1 _	mt-Nd2
2310035C23Rik1	4.77E-18	0.384111	0.18	0.031	9.60E-14	2 M1 _	2310035C23Rik
Gpx8	3.21E-18	1.008116	0.875	0.64	6.46E-14	2 M1 _	Gpx8
Col4a2	3.10E-18	0.761792	0.977	0.85	6.25E-14	2 M1 _	Col4a2
Tnks1bp1	2.17E-18	0.913032	0.602	0.331	4.37E-14	2 M1 _	Tnks1bp1
Trabd2b1	1.98E-18	0.70772	0.344	0.104	3.99E-14	2 M1 _	Trabd2b
Cox8a	1.95E-18	0.656166	0.969	0.944	3.92E-14	2 M1 _	Cox8a
Mut1	1.73E-18	0.461288	0.211	0.041	3.48E-14	2 M1 _	Mut
Fam129a1	1.71E-18	0.916473	0.664	0.381	3.45E-14	2 M1 _	Fam129a

Ywhaq	1.35E-18	0.707637	0.883	0.793	2.72E-14	2 M1 _	Ywhaq
Tbx3os1	1.34E-18	0.445413	0.234	0.048	2.71E-14	2 M1 _	Tbx3os1
Plcb4	1.24E-18	0.975718	0.523	0.243	2.50E-14	2 M1 _	Plcb4
Olfml2b1	1.10E-18	0.583579	0.383	0.117	2.22E-14	2 M1 _	Olfml2b
Kcnb1	9.30E-19	0.457827	0.258	0.058	1.87E-14	2 M1 _	Kcnb1
Ehd1	8.53E-19	0.859248	0.555	0.275	1.72E-14	2 M1 _	Ehd1
Ctgf1	8.07E-19	0.320321	0.219	0.04	1.63E-14	2 M1 _	Ctgf
Csnk1e	4.86E-19	0.9387	0.641	0.365	9.79E-15	2 M1 _	Csnk1e
Mtus1	2.98E-19	0.862331	0.602	0.298	6.01E-15	2 M1 _	Mtus1
Phlda1	2.87E-19	0.957375	0.688	0.405	5.78E-15	2 M1 _	Phlda1
Sort1	2.75E-19	0.617848	0.359	0.106	5.55E-15	2 M1 _	Sort1
Tmem38b	2.06E-19	0.723238	0.391	0.131	4.15E-15	2 M1 _	Tmem38b
Postn	1.76E-19	0.868678	0.836	0.45	3.55E-15	2 M1 _	Postn
Cacna1c	1.25E-19	0.675658	0.438	0.155	2.53E-15	2 M1 _	Cacna1c
Gprc5c	1.17E-19	0.719943	0.453	0.162	2.36E-15	2 M1 _	Gprc5c
Ppp1r12a	1.07E-19	1.056533	0.781	0.639	2.16E-15	2 M1 _	Ppp1r12a
Dgkb	8.49E-20	0.651106	0.234	0.047	1.71E-15	2 M1 _	Dgkb
Map1lc3a	6.25E-20	0.772149	0.883	0.742	1.26E-15	2 M1 _	Map1lc3a
Esyt1	5.23E-20	0.858984	0.688	0.412	1.05E-15	2 M1 _	Esyt1
Atp5b	2.79E-20	0.801881	0.922	0.827	5.62E-16	2 M1 _	Atp5b
Fblim1	2.66E-20	0.853634	0.445	0.162	5.36E-16	2 M1 _	Fblim1
Thsd4	2.62E-20	0.329778	0.156	0.02	5.28E-16	2 M1 _	Thsd4
Mef2a	2.52E-20	0.823569	0.844	0.651	5.08E-16	2 M1 _	Mef2a
Fbxl22	1.98E-20	0.550715	0.133	0.014	3.99E-16	2 M1 _	Fbxl22
Rasal2	1.64E-20	0.869674	0.633	0.32	3.29E-16	2 M1 _	Rasal2
Lrrc8c	1.57E-20	0.843216	0.57	0.231	3.17E-16	2 M1 _	Lrrc8c
Myof	1.49E-20	0.983929	0.711	0.435	3.00E-16	2 M1 _	Myof
Speg	1.42E-20	0.457217	0.234	0.045	2.86E-16	2 M1 _	Speg
Nrarp	1.38E-20	0.764652	0.359	0.102	2.77E-16	2 M1 _	Nrarp
AC149090.1	1.10E-20	0.973424	0.773	0.457	2.21E-16	2 M1 _	AC149090.1
Jam3	9.04E-21	0.876718	0.711	0.389	1.82E-16	2 M1 _	Jam3
Myadm1	7.57E-21	0.921299	0.609	0.322	1.52E-16	2 M1 _	Myadm
Pitx1	6.98E-21	0.97631	0.57	0.269	1.41E-16	2 M1 _	Pitx1
Aaed11	6.40E-21	0.494291	0.281	0.062	1.29E-16	2 M1 _	Aaed1
Arvcf	5.86E-21	0.662757	0.305	0.075	1.18E-16	2 M1 _	Arvcf
Rnd2	5.63E-21	0.5079	0.266	0.055	1.13E-16	2 M1 _	Rnd2
Lurap1l	5.34E-21	0.604024	0.273	0.059	1.08E-16	2 M1 _	Lurap1l
Sssca11	4.28E-21	0.445642	0.18	0.026	8.63E-17	2 M1 _	Sssca1
Ndrg2	4.03E-21	0.855456	0.766	0.476	8.11E-17	2 M1 _	Ndrg2
B230219D22Rik	2.29E-21	0.753774	0.891	0.699	4.61E-17	2 M1 _	B230219D22Rik
Atp2a21	2.01E-21	1.227636	0.828	0.676	4.05E-17	2 M1 _	Atp2a2

AI3141801	1.77E-21	0.630819	0.281	0.062	3.57E-17	2 M1 _	AI314180
Pparg	1.71E-21	0.565883	0.234	0.043	3.45E-17	2 M1 _	Pparg
Ldhb	1.58E-21	0.939394	0.594	0.279	3.18E-17	2 M1 _	Ldhb
Fbxo181	1.14E-21	0.478461	0.227	0.041	2.29E-17	2 M1 _	Fbxo18
Rock1	9.72E-22	1.047364	0.852	0.732	1.96E-17	2 M1 _	Rock1
Hes1	9.49E-22	0.98647	0.883	0.585	1.91E-17	2 M1 _	Hes1
Klf91	7.38E-22	0.888387	0.953	0.767	1.49E-17	2 M1 _	Klf9
Rcan2	6.41E-22	0.71741	0.266	0.054	1.29E-17	2 M1 _	Rcan2
Fads3	6.06E-22	0.911933	0.5	0.192	1.22E-17	2 M1 _	Fads3
Tsc22d1	5.42E-22	0.814544	0.969	0.827	1.09E-17	2 M1 _	Tsc22d1
Csrp1	5.22E-22	1.522849	0.703	0.455	1.05E-17	2 M1 _	Csrp1
Ntn4	4.17E-22	1.039817	0.414	0.139	8.40E-18	2 M1 _	Ntn4
Oaz2	4.06E-22	0.901501	0.727	0.431	8.18E-18	2 M1 _	Oaz2
Arhgdib	3.71E-22	0.936652	0.852	0.574	7.47E-18	2 M1 _	Arhgdib
Fam69a1	3.37E-22	0.582697	0.266	0.055	6.80E-18	2 M1 _	Fam69a
Heyl	3.19E-22	1.017439	0.664	0.334	6.43E-18	2 M1 _	Heyl
Iqsec3	3.01E-22	0.260174	0.133	0.012	6.07E-18	2 M1 _	Iqsec3
Bzw2	2.10E-22	0.876352	0.523	0.21	4.22E-18	2 M1 _	Bzw2
Dync1i2	1.93E-22	1.148966	0.891	0.78	3.88E-18	2 M1 _	Dync1i2
Rab3a	1.41E-22	0.909939	0.43	0.143	2.85E-18	2 M1 _	Rab3a
H3f3b	1.23E-22	0.586794	1	0.982	2.48E-18	2 M1 _	H3f3b
Myom1	1.17E-22	0.342619	0.211	0.032	2.35E-18	2 M1 _	Myom1
Neurl1b	1.08E-22	0.53506	0.305	0.069	2.18E-18	2 M1 _	Neurl1b
Ssfa2	1.06E-22	0.371148	0.195	0.028	2.14E-18	2 M1 _	Ssfa2
H2-M9	7.33E-23	0.731675	0.164	0.019	1.48E-18	2 M1 _	H2-M9
Ywhaz	6.11E-23	0.918135	0.906	0.804	1.23E-18	2 M1 _	Ywhaz
Syne2	5.99E-23	0.928155	0.727	0.404	1.21E-18	2 M1 _	Syne2
Gm424181	4.69E-23	0.735081	1	0.999	9.45E-19	2 M1 _	Gm42418
Tubb4a	4.54E-23	0.341579	0.164	0.019	9.14E-19	2 M1 _	Tubb4a
Cd151	4.38E-23	0.962174	0.828	0.546	8.81E-19	2 M1 _	Cd151
Zbtb161	3.78E-23	0.754359	0.344	0.086	7.62E-19	2 M1 _	Zbtb16
Calm2	3.38E-23	0.839137	0.961	0.899	6.80E-19	2 M1 _	Calm2
Edil3	3.09E-23	0.630872	0.164	0.019	6.22E-19	2 M1 _	Edil3
3110062M04Rik	2.37E-23	0.520177	0.195	0.028	4.78E-19	2 M1 _	3110062M04Rik
Tmem55b1	2.07E-23	0.420494	0.234	0.04	4.17E-19	2 M1 _	Tmem55b
Pdgfc	1.52E-23	0.275373	0.117	0.009	3.07E-19	2 M1 _	Pdgfc
Ednra	1.43E-23	0.917024	0.758	0.438	2.88E-19	2 M1 _	Ednra
Them6	7.52E-24	0.650543	0.281	0.058	1.51E-19	2 M1 _	Them6
Ehd2	6.61E-24	1.116394	0.734	0.473	1.33E-19	2 M1 _	Ehd2
Hey2	6.20E-24	0.405789	0.195	0.026	1.25E-19	2 M1 _	Hey2
Etv1	5.65E-24	1.104913	0.602	0.283	1.14E-19	2 M1 _	Etv1

Fez2	5.12E-24	0.995564	0.695	0.362	1.03E-19	2 M1 _	Fez2
Vstm4	3.80E-24	1.104088	0.633	0.303	7.65E-20	2 M1 _	Vstm4
Pkia	3.63E-24	0.58443	0.258	0.047	7.31E-20	2 M1 _	Pkia
Fabp7	3.60E-24	0.270792	0.117	0.008	7.25E-20	2 M1 _	Fabp7
Efhd2	3.54E-24	0.96251	0.664	0.309	7.12E-20	2 M1 _	Efhd2
Nt5dc2	3.20E-24	0.84352	0.43	0.13	6.44E-20	2 M1 _	Nt5dc2
Sorbs2	2.95E-24	1.120658	0.531	0.174	5.93E-20	2 M1 _	Sorbs2
Epn2	2.87E-24	1.133664	0.664	0.364	5.78E-20	2 M1 _	Epn2
Selenof	2.68E-24	0.87148	0.953	0.842	5.39E-20	2 M1 _	Selenof
Kcne4	2.12E-24	0.689172	0.289	0.058	4.27E-20	2 M1 _	Kcne4
Crim1	1.78E-24	1.002026	0.648	0.277	3.59E-20	2 M1 _	Crim1
Ehd3	1.75E-24	0.766696	0.43	0.13	3.52E-20	2 M1 _	Ehd3
Scn3a	1.31E-24	0.495174	0.219	0.032	2.64E-20	2 M1 _	Scn3a
Alkal2	6.27E-25	0.672313	0.273	0.051	1.26E-20	2 M1 _	Alkal2
Stom	4.72E-25	0.758542	0.477	0.151	9.50E-21	2 M1 _	Stom
Tspan12	4.63E-25	0.819723	0.469	0.139	9.32E-21	2 M1 _	Tspan12
Procr	4.45E-25	0.819533	0.406	0.112	8.96E-21	2 M1 _	Procr
Pdlim3	3.85E-25	0.680984	0.281	0.052	7.75E-21	2 M1 _	Pdlim3
Meg3	1.89E-25	1.236532	0.898	0.636	3.81E-21	2 M1 _	Meg3
BC0033311	1.55E-25	0.606634	0.289	0.056	3.13E-21	2 M1 _	BC003331
Adcy6	1.47E-25	0.889404	0.539	0.194	2.97E-21	2 M1 _	Adcy6
Atp1b1	1.21E-25	1.232862	0.398	0.11	2.44E-21	2 M1 _	Atp1b1
Mum11	1.20E-25	0.574643	0.266	0.047	2.42E-21	2 M1 _	Mum1
Myl6	1.17E-25	1.053	0.984	0.966	2.35E-21	2 M1 _	Myl6
Pten	9.26E-26	0.956577	0.914	0.716	1.87E-21	2 M1 _	Pten
Lpp1	7.52E-26	1.348739	0.797	0.621	1.51E-21	2 M1 _	Lpp
Ppp1r1a	6.28E-26	0.463633	0.188	0.022	1.26E-21	2 M1 _	Ppp1r1a
Prkar1a	6.08E-26	0.91399	0.906	0.783	1.22E-21	2 M1 _	Prkar1a
Fam213b	5.10E-26	0.257349	0.148	0.013	1.03E-21	2 M1 _	Fam213b
Mgst3	4.49E-26	0.992226	0.562	0.216	9.04E-22	2 M1 _	Mgst3
Ptprk	4.10E-26	0.839892	0.594	0.227	8.27E-22	2 M1 _	Ptprk
Prkar2b	3.44E-26	0.676997	0.32	0.067	6.94E-22	2 M1 _	Prkar2b
G0s21	3.11E-26	1.473209	0.797	0.507	6.25E-22	2 M1 _	G0s2
Acta2	2.86E-26	3.239459	0.539	0.21	5.76E-22	2 M1 _	Acta2
Actb	2.82E-26	0.657782	1	0.992	5.69E-22	2 M1 _	Actb
Lgalsl	2.45E-26	1.267549	0.672	0.351	4.93E-22	2 M1 _	Lgalsl
7-Sep	1.96E-26	0.879213	0.969	0.896	3.96E-22	2 M1 _	7-Sep
Pde1a	1.91E-26	0.853752	0.516	0.169	3.85E-22	2 M1 _	Pde1a
Zeb2	1.28E-26	1.080025	0.898	0.69	2.58E-22	2 M1 _	Zeb2
Pde1b	1.19E-26	0.508044	0.258	0.042	2.39E-22	2 M1 _	Pde1b
Mrvi1	1.17E-26	1.035748	0.562	0.212	2.36E-22	2 M1 _	Mrvi1

Gm10687	1.05E-26	0.430406	0.234	0.034	2.10E-22	2 M1 _	Gm10687
Kcnmb1	5.21E-27	0.357619	0.125	0.008	1.05E-22	2 M1 _	Kcnmb1
Lars21	4.01E-27	0.86389	0.969	0.805	8.08E-23	2 M1 _	Lars2
Ccl111	3.13E-27	1.806674	0.734	0.424	6.31E-23	2 M1 _	Ccl11
Galnt16	2.80E-27	0.805958	0.422	0.114	5.64E-23	2 M1 _	Galnt16
Chst1	2.18E-27	0.772908	0.398	0.099	4.38E-23	2 M1 _	Chst1
Pcdh1	2.05E-27	0.791692	0.516	0.161	4.12E-23	2 M1 _	Pcdh1
Malat1	1.98E-27	0.834549	0.984	0.998	3.98E-23	2 M1 _	Malat1
Sod3	1.94E-27	1.110127	0.641	0.279	3.91E-23	2 M1 _	Sod3
Stum	1.67E-27	0.315224	0.156	0.014	3.37E-23	2 M1 _	Stum
Aatk	1.12E-27	0.426275	0.227	0.03	2.25E-23	2 M1 _	Aatk
Klhl23	1.06E-27	0.767669	0.406	0.101	2.13E-23	2 M1 _	Klhl23
Oraov1	8.36E-28	0.4945	0.195	0.023	1.68E-23	2 M1 _	Oraov1
Crip1	8.17E-28	1.571276	0.992	0.948	1.65E-23	2 M1 _	Crip1
Atp2a3	6.63E-28	0.792075	0.359	0.081	1.33E-23	2 M1 _	Atp2a3
Ift43	4.21E-28	1.140894	0.688	0.332	8.47E-24	2 M1 _	Ift43
Btbd3	3.63E-28	1.147943	0.648	0.273	7.31E-24	2 M1 _	Btbd3
Eps81	2.59E-28	1.173237	0.75	0.409	5.22E-24	2 M1 _	Eps8
Arhgap10	1.90E-28	1.115156	0.727	0.368	3.82E-24	2 M1 _	Arhgap10
Dstn	1.54E-28	1.473502	0.969	0.812	3.09E-24	2 M1 _	Dstn
Cog7	1.36E-28	1.046216	0.539	0.183	2.73E-24	2 M1 _	Cog7
Aldoa	7.24E-29	0.998356	0.961	0.841	1.46E-24	2 M1 _	Aldoa
Mamdc2	6.31E-29	0.399164	0.219	0.027	1.27E-24	2 M1 _	Mamdc2
Slc38a11	2.04E-29	0.545744	0.219	0.026	4.11E-25	2 M1 _	Slc38a11
Ppm1e	1.63E-29	0.467939	0.203	0.022	3.27E-25	2 M1 _	Ppm1e
2810474O19Rik1	1.53E-29	0.661513	0.336	0.065	3.08E-25	2 M1 _	2810474O19Rik
Fam220a1	1.41E-29	0.659189	0.281	0.047	2.83E-25	2 M1 _	Fam220a
Inpp4b	1.00E-29	1.237421	0.672	0.265	2.02E-25	2 M1 _	Inpp4b
Coro1b1	8.13E-30	0.770939	0.383	0.089	1.64E-25	2 M1 _	Coro1b
Mpp2	7.53E-30	0.394106	0.203	0.022	1.52E-25	2 M1 _	Mpp2
Map3k7cl	7.49E-30	0.586353	0.133	0.008	1.51E-25	2 M1 _	Map3k7cl
Vcl	4.44E-30	1.215865	0.781	0.463	8.95E-26	2 M1 _	Vcl
mt-Atp81	2.09E-30	1.162232	0.852	0.593	4.21E-26	2 M1 _	mt-Atp8
Cd36	1.97E-30	0.907701	0.602	0.182	3.97E-26	2 M1 _	Cd36
Ccdc160	1.78E-30	0.413215	0.211	0.023	3.58E-26	2 M1 _	Ccdc160
Cdkn1a	1.33E-30	0.941564	0.555	0.168	2.68E-26	2 M1 _	Cdkn1a
Pde4b	1.05E-30	0.976253	0.453	0.12	2.11E-26	2 M1 _	Pde4b
Tmem1001	8.92E-31	1.180328	0.672	0.264	1.80E-26	2 M1 _	Tmem100
Mylk	7.90E-31	1.65274	0.891	0.624	1.59E-26	2 M1 _	Mylk
Errfi11	6.47E-31	1.444423	0.898	0.614	1.30E-26	2 M1 _	Errfi1
Snrk	6.46E-31	0.98975	0.938	0.591	1.30E-26	2 M1 _	Snrk

Emid1	3.20E-31	1.140546	0.617	0.219	6.45E-27	2 M1 _	Emid1
Plekhg3	2.67E-31	0.914134	0.453	0.12	5.39E-27	2 M1 _	Plekhg3
Ptms	2.29E-31	0.990743	0.969	0.902	4.61E-27	2 M1 _	Ptms
Flna	1.77E-31	1.773493	0.844	0.633	3.57E-27	2 M1 _	Flna
Hcn2	6.53E-32	0.497362	0.188	0.017	1.32E-27	2 M1 _	Hcn2
Ccdc68	4.65E-32	0.490954	0.266	0.035	9.36E-28	2 M1 _	Ccdc68
Insc	4.22E-32	0.537401	0.195	0.019	8.50E-28	2 M1 _	Insc
Mprip	3.12E-32	1.222945	0.875	0.708	6.28E-28	2 M1 _	Mprip
Rtn4rl1	9.16E-33	0.812961	0.344	0.063	1.84E-28	2 M1 _	Rtn4rl1
Zcchc111	3.92E-33	0.721943	0.336	0.059	7.89E-29	2 M1 _	Zcchc11
Foxs1	3.38E-33	1.158727	0.789	0.389	6.81E-29	2 M1 _	Foxs1
Nrip2	2.18E-33	0.856172	0.266	0.034	4.39E-29	2 M1 _	Nrip2
Dmd	1.99E-33	1.227925	0.633	0.236	4.00E-29	2 M1 _	Dmd
Cacna2d1	1.63E-33	1.249383	0.75	0.372	3.28E-29	2 M1 _	Cacna2d1
Adgrv1	1.51E-33	0.39922	0.141	0.008	3.05E-29	2 M1 _	Adgrv1
Zcchc61	1.35E-33	0.805527	0.406	0.082	2.71E-29	2 M1 _	Zcchc6
Fermt2	1.30E-33	1.113236	0.977	0.764	2.62E-29	2 M1 _	Fermt2
Pde4c	1.18E-33	0.402068	0.172	0.013	2.38E-29	2 M1 _	Pde4c
Skiv2l21	8.98E-34	0.643734	0.305	0.047	1.81E-29	2 M1 _	Skiv2l2
Tmem35a	7.51E-34	0.445218	0.18	0.014	1.51E-29	2 M1 _	Tmem35a
Ebf1	4.67E-34	1.172025	0.961	0.78	9.40E-30	2 M1 _	Ebf1
Synpo2	3.26E-34	1.170751	0.477	0.122	6.57E-30	2 M1 _	Synpo2
Atpif1	2.18E-34	1.035382	0.961	0.767	4.40E-30	2 M1 _	Atpif1
Cbfa2t3	1.64E-34	1.24156	0.656	0.228	3.30E-30	2 M1 _	Cbfa2t3
Trpv2	1.38E-34	0.670413	0.352	0.06	2.79E-30	2 M1 _	Trpv2
Slc25a4	1.18E-34	0.980632	0.977	0.854	2.37E-30	2 M1 _	Slc25a4
Trove21	9.28E-35	0.831406	0.375	0.069	1.87E-30	2 M1 _	Trove2
4632415L05Rik1	1.91E-35	0.550284	0.227	0.024	3.84E-31	2 M1 _	4632415L05Rik
Adap2	5.26E-36	1.161319	0.656	0.229	1.06E-31	2 M1 _	Adap2
Tbx2	4.57E-36	1.365148	0.688	0.274	9.21E-32	2 M1 _	Tbx2
Pyurf1	4.08E-36	0.568963	0.273	0.035	8.22E-32	2 M1 _	Pyurf
Lin7a	3.50E-36	1.100281	0.734	0.292	7.04E-32	2 M1 _	Lin7a
Fam81a	2.71E-36	0.633114	0.219	0.021	5.45E-32	2 M1 _	Fam81a
Colec11	1.99E-36	1.172251	0.32	0.046	4.00E-32	2 M1 _	Colec11
Tbx3	6.50E-37	1.023181	0.516	0.127	1.31E-32	2 M1 _	Tbx3
Gm26526	3.24E-37	0.439713	0.211	0.019	6.52E-33	2 M1 _	Gm26526
Angptl2	1.91E-37	1.582118	0.75	0.339	3.85E-33	2 M1 _	Angptl2
Bcam	7.89E-38	1.218828	0.805	0.304	1.59E-33	2 M1 _	Bcam
Ngf	6.16E-38	0.935224	0.516	0.128	1.24E-33	2 M1 _	Ngf
Tns1	5.69E-38	1.446716	0.852	0.516	1.15E-33	2 M1 _	Tns1
Khdrbs3	5.03E-38	1.05663	0.539	0.142	1.01E-33	2 M1 _	Khdrbs3

Raph1	4.91E-38	1.454774	0.758	0.332	9.89E-34	2 M1 _	Raph1
Hspb2	1.95E-38	1.014713	0.445	0.088	3.93E-34	2 M1 _	Hspb2
Rbpms2	1.55E-38	0.88904	0.438	0.085	3.12E-34	2 M1 _	Rbpms2
Slc24a3	8.66E-39	0.871233	0.375	0.063	1.74E-34	2 M1 _	Slc24a3
Parm1	3.93E-39	1.069676	0.484	0.105	7.91E-35	2 M1 _	Parm1
Pdgfrb	2.35E-39	1.381282	1	0.826	4.74E-35	2 M1 _	Pdgfrb
Cap2	2.09E-39	0.742337	0.305	0.04	4.21E-35	2 M1 _	Cap2
Fam103a11	1.08E-39	0.680041	0.344	0.052	2.18E-35	2 M1 _	Fam103a1
Tmem94	7.97E-40	1.084108	0.484	0.109	1.61E-35	2 M1 _	Tmem94
Rarres21	3.66E-40	1.424075	0.969	0.675	7.38E-36	2 M1 _	Rarres2
Cspg4	3.09E-40	1.35627	0.766	0.311	6.22E-36	2 M1 _	Cspg4
Map7d2	1.91E-40	0.590921	0.258	0.025	3.85E-36	2 M1 _	Map7d2
Epas1	9.21E-41	1.513304	0.906	0.509	1.86E-36	2 M1 _	Epas1
Rgs6	9.16E-41	0.689862	0.273	0.029	1.84E-36	2 M1 _	Rgs6
Rbpms	4.17E-41	1.289905	0.953	0.706	8.40E-37	2 M1 _	Rbpms
Ccdc141	1.89E-41	0.780415	0.438	0.078	3.80E-37	2 M1 _	Ccdc141
Dtx3	1.63E-41	1.379761	0.844	0.418	3.29E-37	2 M1 _	Dtx3
P2rx1	1.44E-41	0.574161	0.141	0.005	2.90E-37	2 M1 _	P2rx1
Actn4	1.03E-41	1.436316	0.938	0.707	2.08E-37	2 M1 _	Actn4
Timp3	6.41E-42	1.438321	0.984	0.781	1.29E-37	2 M1 _	Timp3
Wfdc1	6.14E-42	1.042753	0.414	0.074	1.24E-37	2 M1 _	Wfdc1
D10Jhu81e1	5.17E-42	0.647206	0.289	0.034	1.04E-37	2 M1 _	D10Jhu81e
Limd1	1.96E-42	1.459066	0.812	0.38	3.94E-38	2 M1 _	Limd1
4-Sep	8.13E-43	1.513959	0.961	0.705	1.64E-38	2 M1 _	4-Sep
Cfap69	6.51E-43	0.63289	0.203	0.014	1.31E-38	2 M1 _	Cfap69
Mtss1l	4.33E-43	0.488509	0.195	0.012	8.72E-39	2 M1 _	Mtss1l
Gcnt2	2.55E-43	1.203672	0.664	0.18	5.14E-39	2 M1 _	Gcnt2
1500011K16Rik1	1.67E-43	0.643623	0.305	0.036	3.36E-39	2 M1 _	1500011K16Rik
Sgip1	1.43E-43	0.830152	0.43	0.07	2.88E-39	2 M1 _	Sgip1
Olfr558	1.06E-43	1.380104	0.555	0.118	2.14E-39	2 M1 _	Olfr558
Tesc	8.00E-44	1.499826	0.445	0.079	1.61E-39	2 M1 _	Tesc
Itga1	6.58E-44	1.510149	0.977	0.79	1.32E-39	2 M1 _	Itga1
Slco3a1	5.60E-44	1.336493	0.789	0.296	1.13E-39	2 M1 _	Slco3a1
Tpm1	2.34E-44	2.172516	0.938	0.711	4.72E-40	2 M1 _	Tpm1
Arhgap42	2.33E-44	1.215594	0.641	0.178	4.69E-40	2 M1 _	Arhgap42
Pcdh19	1.55E-44	1.551265	0.883	0.439	3.13E-40	2 M1 _	Pcdh19
Aspn	1.18E-44	1.572581	0.922	0.498	2.38E-40	2 M1 _	Aspn
Gm13889	1.15E-44	1.448109	0.93	0.475	2.31E-40	2 M1 _	Gm13889
Gm13861	2.18E-45	0.823417	0.352	0.043	4.39E-41	2 M1 _	Gm13861
Lrrc8b	9.68E-46	0.745588	0.328	0.038	1.95E-41	2 M1 _	Lrrc8b
Ccdc3	8.83E-46	0.641633	0.312	0.034	1.78E-41	2 M1 _	Ccdc3

Cav1	7.86E-46	1.371781	0.82	0.283	1.58E-41	2 M1 _	Cav1
Trpc6	6.42E-46	0.703711	0.375	0.049	1.29E-41	2 M1 _	Trpc6
Mtcl1	2.56E-46	0.548067	0.242	0.019	5.16E-42	2 M1 _	Mtcl1
Ano4	1.40E-46	0.484588	0.172	0.007	2.83E-42	2 M1 _	Ano4
Id4	5.40E-47	1.661954	0.609	0.154	1.09E-42	2 M1 _	Id4
Rhob1	3.59E-47	1.787128	0.938	0.599	7.23E-43	2 M1 _	Rhob
Arhgef17	1.97E-47	1.415795	0.773	0.279	3.96E-43	2 M1 _	Arhgef17
Gucy1a2	9.23E-48	0.970839	0.523	0.105	1.86E-43	2 M1 _	Gucy1a2
Cryab	3.75E-48	1.510929	0.914	0.474	7.54E-44	2 M1 _	Cryab
Myh11	3.68E-48	3.195112	0.5	0.096	7.41E-44	2 M1 _	Myh11
Esam	2.34E-48	1.276176	0.914	0.303	4.72E-44	2 M1 _	Esam
Cald1	2.09E-48	1.453937	1	0.866	4.20E-44	2 M1 _	Cald1
Kcnh1	4.41E-49	0.685765	0.211	0.012	8.89E-45	2 M1 _	Kcnh1
Hrct1	8.26E-50	1.286075	0.594	0.134	1.66E-45	2 M1 _	Hrct1
Fzd4	7.52E-51	1.462537	0.859	0.319	1.51E-46	2 M1 _	Fzd4
Casq2	5.11E-51	1.177449	0.367	0.043	1.03E-46	2 M1 _	Casq2
Tspan15	1.91E-51	1.32374	0.633	0.146	3.84E-47	2 M1 _	Tspan15
Fam162a	7.40E-52	2.065189	0.93	0.597	1.49E-47	2 M1 _	Fam162a
Jph2	7.36E-52	0.685646	0.297	0.026	1.48E-47	2 M1 _	Jph2
Rdh5	3.49E-53	1.142679	0.508	0.087	7.02E-49	2 M1 _	Rdh5
Myo1b	5.62E-54	1.700577	0.938	0.476	1.13E-49	2 M1 _	Myo1b
Rasl12	3.05E-54	1.012489	0.578	0.103	6.15E-50	2 M1 _	Rasl12
Fam208a1	2.45E-54	0.982657	0.453	0.066	4.94E-50	2 M1 _	Fam208a
Kcna5	1.62E-54	0.598335	0.25	0.016	3.27E-50	2 M1 _	Kcna5
Sdc1	3.91E-55	1.748314	0.688	0.189	7.86E-51	2 M1 _	Sdc1
Dirc21	2.48E-55	0.821306	0.359	0.039	4.99E-51	2 M1 _	Dirc2
Ebf2	2.04E-56	1.296978	0.672	0.151	4.12E-52	2 M1 _	Ebf2
Kcnk3	1.18E-56	0.806341	0.391	0.043	2.37E-52	2 M1 _	Kcnk3
Itgb1	1.11E-56	1.458128	0.984	0.895	2.23E-52	2 M1 _	Itgb1
Npy1r	8.98E-57	1.309514	0.547	0.096	1.81E-52	2 M1 _	Npy1r
Ndufa4l2	3.99E-57	1.96895	0.977	0.6	8.04E-53	2 M1 _	Ndufa4l2
Carmn	3.68E-57	1.291576	0.75	0.179	7.41E-53	2 M1 _	Carmn
AY0361181	3.53E-57	2.21072	1	0.542	7.11E-53	2 M1 _	AY036118
Pdlim1	3.27E-57	1.280796	0.789	0.231	6.59E-53	2 M1 _	Pdlim1
Dusp4	2.09E-57	0.686418	0.336	0.03	4.22E-53	2 M1 _	Dusp4
Rem1	1.71E-57	1.287915	0.664	0.141	3.43E-53	2 M1 _	Rem1
Vtn	1.28E-57	1.64695	0.758	0.197	2.58E-53	2 M1 _	Vtn
Tagln	1.18E-57	3.570739	0.695	0.178	2.38E-53	2 M1 _	Tagln
Hopx	6.89E-58	1.917124	0.812	0.269	1.39E-53	2 M1 _	Hopx
Tbc1d1	1.53E-58	1.431149	0.852	0.283	3.08E-54	2 M1 _	Tbc1d1
Cpe	1.31E-58	1.903571	0.906	0.42	2.64E-54	2 M1 _	Cpe

Spaar	1.01E-58	0.854414	0.359	0.035	2.04E-54	2 M1 _	Spaar
Palm2	9.02E-59	0.358574	0.133	0.001	1.82E-54	2 M1 _	Palm2
Gjc1	9.00E-59	1.846177	0.922	0.421	1.81E-54	2 M1 _	Gjc1
1700020I14Rik1	7.08E-59	1.190925	0.523	0.085	1.43E-54	2 M1 _	1700020I14Rik
Steap41	4.15E-59	2.154556	0.938	0.423	8.35E-55	2 M1 _	Steap4
Cdk15	5.59E-61	0.508628	0.211	0.008	1.12E-56	2 M1 _	Cdk15
Pdgfa	2.41E-62	1.621745	0.789	0.226	4.86E-58	2 M1 _	Pdgfa
Gng11	1.12E-62	1.900109	1	0.81	2.25E-58	2 M1 _	Gng11
Rasl11a	4.66E-63	1.415821	0.797	0.2	9.39E-59	2 M1 _	Rasl11a
Kcnj8	1.59E-64	2.73249	0.805	0.22	3.20E-60	2 M1 _	Kcnj8
Cobll1	1.41E-64	1.218083	0.547	0.084	2.84E-60	2 M1 _	Cobll1
Tmem52	3.12E-65	0.968366	0.492	0.065	6.28E-61	2 M1 _	Tmem5
1810011H11Rik	7.03E-66	0.663417	0.188	0.004	1.42E-61	2 M1 _	1810011H11Rik
Mfge8	5.18E-66	2.266503	0.984	0.793	1.04E-61	2 M1 _	Mfge8
Fam96b1	1.63E-66	0.919315	0.445	0.051	3.29E-62	2 M1 _	Fam96b
Filip1l	1.20E-66	2.095781	0.859	0.279	2.42E-62	2 M1 _	Filip1l
Gucy1a1	1.77E-67	1.984181	0.953	0.353	3.57E-63	2 M1 _	Gucy1a1
Mcam	9.88E-68	1.51007	0.648	0.121	1.99E-63	2 M1 _	Mcam
1810022K09Rik1	6.83E-68	1.054988	0.547	0.079	1.38E-63	2 M1 _	1810022K09Rik
Gucy1b1	2.61E-68	1.923912	0.945	0.333	5.26E-64	2 M1 _	Gucy1b1
Bcar3	3.50E-69	0.870365	0.414	0.039	7.04E-65	2 M1 _	Bcar3
Tinagl1	2.35E-69	1.79119	0.945	0.273	4.74E-65	2 M1 _	Tinagl1
Plxnb1	3.03E-70	0.730129	0.336	0.023	6.10E-66	2 M1 _	Plxnb1
Dgkg	2.77E-71	0.798869	0.336	0.022	5.59E-67	2 M1 _	Dgkg
Slc12a2	1.94E-71	2.290456	0.852	0.245	3.91E-67	2 M1 _	Slc12a2
Aoc31	1.58E-71	1.707271	0.75	0.172	3.19E-67	2 M1 _	Aoc3
Atp1b2	1.49E-71	1.722282	0.875	0.263	3.00E-67	2 M1 _	Atp1b2
Lmod1	6.63E-72	1.432205	0.555	0.073	1.33E-67	2 M1 _	Lmod1
Adap2os	1.09E-72	1.486627	0.703	0.134	2.19E-68	2 M1 _	Adap2os
Aes1	3.03E-73	1.4382	0.664	0.108	6.11E-69	2 M1 _	Aes
Cystm1	1.63E-73	1.807844	0.914	0.286	3.29E-69	2 M1 _	Cystm1
Abcc9	9.17E-74	2.891062	0.906	0.302	1.85E-69	2 M1 _	Abcc9
2410015M20Rik1	9.09E-74	1.191395	0.602	0.091	1.83E-69	2 M1 _	2410015M20Rik
Chn1	3.27E-74	1.110782	0.508	0.057	6.60E-70	2 M1 _	Chn1
Atp5o.11	2.19E-74	1.301331	0.625	0.097	4.41E-70	2 M1 _	Atp5o.1
Mnd1	1.65E-74	1.668482	0.633	0.101	3.33E-70	2 M1 _	Mnd1
2010111I01Rik1	1.03E-74	0.982252	0.492	0.056	2.07E-70	2 M1 _	2010111I01Rik
Des	3.85E-76	1.917845	0.844	0.2	7.76E-72	2 M1 _	Des
Pde3a	5.89E-77	1.661412	0.688	0.12	1.19E-72	2 M1 _	Pde3a
Gja4	6.38E-78	1.685003	0.93	0.216	1.28E-73	2 M1 _	Gja4
Mapt	8.25E-79	1.150495	0.484	0.048	1.66E-74	2 M1 _	Mapt

Pde5a	4.93E-82	1.817907	0.836	0.185	9.94E-78	2 M1 _	Pde5a
Slc4a8	1.81E-83	1.235719	0.484	0.044	3.65E-79	2 M1 _	Slc4a8
Agtr1a	5.19E-84	1.629851	0.641	0.09	1.05E-79	2 M1 _	Agtr1a
Slc2a4	4.89E-85	1.18378	0.586	0.067	9.85E-81	2 M1 _	Slc2a4
Ednrb	3.20E-85	3.056014	0.938	0.281	6.43E-81	2 M1 _	Ednrb
Rasgrp2	4.33E-86	2.114367	0.891	0.221	8.72E-82	2 M1 _	Rasgrp2
Daam2	2.83E-87	1.526932	0.75	0.127	5.69E-83	2 M1 _	Daam2
Tpm2	2.64E-87	3.315021	0.984	0.372	5.31E-83	2 M1 _	Tpm2
Itga7	6.00E-88	1.277585	0.719	0.101	1.21E-83	2 M1 _	Itga7
Sncg	1.76E-88	2.141263	0.891	0.191	3.55E-84	2 M1 _	Sncg
Ephx3	1.36E-89	1.627182	0.594	0.066	2.73E-85	2 M1 _	Ephx3
Ano1	5.26E-91	1.282782	0.633	0.072	1.06E-86	2 M1 _	Ano1
Myl9	4.81E-91	3.052524	1	0.338	9.69E-87	2 M1 _	Myl9
2010107E04Rik1	1.89E-91	1.48712	0.688	0.098	3.80E-87	2 M1 _	2010107E04Rik
Minos11	2.39E-98	1.461851	0.711	0.095	4.81E-94	2 M1 _	Minos1
Art3	1.15E-98	2.047923	0.766	0.115	2.32E-94	2 M1 _	Art3
Rgs5	1.02E-98	2.838544	0.984	0.217	2.06E-94	2 M1 _	Rgs5
6430573F11Rik	1.34E-99	0.657038	0.266	0.005	2.69E-95	2 M1 _	6430573F11Rik
Ajuba	2.30E-106	1.371846	0.672	0.075	4.64E-102	2 M1 _	Ajuba
1110008F13Rik1	6.55E-107	1.336702	0.633	0.066	1.32E-102	2 M1 _	1110008F13Rik
Notch3	1.35E-108	2.5046	0.977	0.21	2.72E-104	2 M1 _	Notch3
Lgi1	4.46E-110	2.011954	0.711	0.081	8.99E-106	2 M1 _	Lgi1
Serpini1	1.45E-110	2.009952	0.828	0.12	2.92E-106	2 M1 _	Serpini1
Rgs4	4.77E-114	1.955958	0.852	0.115	9.60E-110	2 M1 _	Rgs4
Higd1b	7.34E-119	2.420674	0.945	0.152	1.48E-114	2 M1 _	Higd1b
Fam212b	2.21E-121	0.830621	0.328	0.006	4.44E-117	2 M1 _	Fam212b
Usmg51	1.55E-123	1.748283	0.789	0.096	3.12E-119	2 M1 _	Usmg5
Pde8b	2.81E-127	1.490355	0.609	0.043	5.67E-123	2 M1 _	Pde8b
Cpm	3.82E-129	2.193179	0.758	0.078	7.70E-125	2 M1 _	Cpm
Cox4i2	1.08E-142	2.590002	0.953	0.13	2.18E-138	2 M1 _	Cox4i2
Gm14005	7.44E-149	1.112219	0.453	0.013	1.50E-144	2 M1 _	Gm14005
Elmod1	1.34E-158	1.841322	0.609	0.029	2.71E-154	2 M1 _	Elmod1
Ppp1r14a	6.04E-162	2.511993	0.922	0.103	1.22E-157	2 M1 _	Ppp1r14a
Ace2	9.49E-164	2.255285	0.719	0.046	1.91E-159	2 M1 _	Ace2
Tusc5	1.21E-207	1.19777	0.469	0.003	2.44E-203	2 M1 _	Tusc5
Inpp5k	2.42E-06	0.303866	0.273	0.162	0.048746	3 L1 _	Inpp5k
Cct6a	2.33E-06	0.276628	0.596	0.446	0.046923	3 L1 _	Cct6a
Sun1	2.25E-06	0.253589	0.319	0.202	0.045307	3 L1 _	Sun1
Purb1	2.14E-06	0.274604	0.75	0.667	0.043071	3 L1 _	Purb
Eif4ebp22	2.08E-06	0.320421	0.596	0.477	0.041811	3 L1 _	Eif4ebp2
Sbno2	2.02E-06	0.288267	0.3	0.185	0.040739	3 L1 _	Sbno2

Snai21	1.82E-06	0.307758	0.327	0.204	0.036614	3 L1 _	Snai2
Pcgf5	1.79E-06	0.310148	0.238	0.132	0.036087	3 L1 _	Pcgf5
Znrf11	1.53E-06	0.275235	0.381	0.248	0.030714	3 L1 _	Znrf1
Fosb1	1.36E-06	0.505995	0.523	0.397	0.027386	3 L1 _	Fosb
Prrc2c	1.32E-06	0.309153	0.85	0.799	0.026539	3 L1 _	Prrc2c
Zkscan3	1.27E-06	0.365322	0.492	0.365	0.025493	3 L1 _	Zkscan3
Slc44a1	1.22E-06	0.292172	0.415	0.282	0.024659	3 L1 _	Slc44a1
Ccser2	1.21E-06	0.296579	0.665	0.555	0.02428	3 L1 _	Ccser2
Idnk1	1.16E-06	0.281856	0.527	0.395	0.023428	3 L1 _	Idnk
Spag9	1.14E-06	0.277349	0.762	0.666	0.023008	3 L1 _	Spag9
Fas1	1.07E-06	0.271764	0.273	0.159	0.021527	3 L1 _	Fas
Phf14	1.03E-06	0.353619	0.469	0.333	0.020671	3 L1 _	Phf14
Polg	1.02E-06	0.287214	0.296	0.181	0.020631	3 L1 _	Polg
Spaca6	9.74E-07	0.269785	0.373	0.244	0.019614	3 L1 _	Spaca6
Lrrfip11	9.21E-07	0.301326	0.612	0.464	0.018549	3 L1 _	Lrrfip1
Rnf215	8.95E-07	0.287691	0.4	0.27	0.018029	3 L1 _	Rnf215
Ppp1r9b	8.80E-07	0.275779	0.423	0.294	0.017729	3 L1 _	Ppp1r9b
Sft2d1	8.63E-07	0.367886	0.346	0.232	0.017381	3 L1 _	Sft2d1
Ash1l	8.46E-07	0.319955	0.723	0.624	0.017047	3 L1 _	Ash1l
Epn1	8.30E-07	0.288239	0.481	0.343	0.016714	3 L1 _	Epn1
Stx12	6.86E-07	0.346546	0.477	0.349	0.013808	3 L1 _	Stx12
Zfp644	6.85E-07	0.321526	0.665	0.546	0.01379	3 L1 _	Zfp644
Sash11	6.80E-07	0.261143	0.708	0.563	0.013689	3 L1 _	Sash1
Fcho2	6.39E-07	0.403956	0.612	0.5	0.012875	3 L1 _	Fcho2
Herc6	6.14E-07	0.377721	0.327	0.201	0.01236	3 L1 _	Herc6
Eef21	6.13E-07	0.250586	0.931	0.895	0.012353	3 L1 _	Eef2
Mtm1	5.29E-07	0.272734	0.215	0.11	0.010657	3 L1 _	Mtm1
Elovl1	5.26E-07	0.260783	0.5	0.361	0.010586	3 L1 _	Elovl1
Dpp8	5.21E-07	0.362193	0.496	0.36	0.010497	3 L1 _	Dpp8
Pcnx1	4.97E-07	0.302685	0.377	0.237	0.010012	3 L1 _	Pcnx
Tra2a	4.97E-07	0.276933	0.719	0.63	0.010003	3 L1 _	Tra2a
Hspa1a	4.76E-07	0.428954	0.342	0.207	0.009585	3 L1 _	Hspa1a
Ocel1	4.48E-07	0.254474	0.227	0.12	0.00903	3 L1 _	Ocel1
Tspo	4.28E-07	0.333982	0.915	0.857	0.008611	3 L1 _	Tspo
Myh92	4.05E-07	0.315058	0.881	0.822	0.008154	3 L1 _	Myh9
Tsc22d32	3.99E-07	0.261352	0.627	0.461	0.008026	3 L1 _	Tsc22d3
Tmem234	3.87E-07	0.264814	0.8	0.707	0.007791	3 L1 _	Tmem234
Pak21	3.78E-07	0.336728	0.746	0.65	0.007623	3 L1 _	Pak2
Btbd9	3.75E-07	0.263579	0.342	0.211	0.007555	3 L1 _	Btbd9
Ifnar2	3.48E-07	0.378133	0.762	0.669	0.007001	3 L1 _	Ifnar2
Osmr	3.46E-07	0.296207	0.558	0.42	0.006973	3 L1 _	Osmr

Dip2b1	3.44E-07	0.290762	0.385	0.246	0.006926	3 L1 _	Dip2b
Csnk2a1	3.35E-07	0.276145	0.785	0.699	0.006752	3 L1 _	Csnk2a1
Cactin1	3.33E-07	0.296707	0.227	0.119	0.006708	3 L1 _	Cactin
Gpd21	3.30E-07	0.305598	0.219	0.115	0.006648	3 L1 _	Gpd2
Tmbim6	3.30E-07	0.329547	0.854	0.784	0.006636	3 L1 _	Tmbim6
Ifit2	3.26E-07	0.416211	0.312	0.188	0.006572	3 L1 _	Ifit2
Nrbp11	3.19E-07	0.294817	0.615	0.483	0.006422	3 L1 _	Nrbp1
Serpinb9	2.89E-07	0.295801	0.462	0.326	0.005819	3 L1 _	Serpinb9
Nbea	2.83E-07	0.284098	0.396	0.254	0.005697	3 L1 _	Nbea
2700081O15Rik1	2.72E-07	0.278111	0.415	0.268	0.005483	3 L1 _	2700081O15Rik
Cttnbp2nl1	2.66E-07	0.287752	0.258	0.144	0.005359	3 L1 _	Cttnbp2nl
Hpcal12	2.56E-07	0.315239	0.488	0.352	0.005157	3 L1 _	Hpcal1
Tapbpl1	2.51E-07	0.272274	0.315	0.188	0.005062	3 L1 _	Tapbpl
Dram21	2.30E-07	0.278482	0.342	0.21	0.004633	3 L1 _	Dram2
Ralgds	2.23E-07	0.318882	0.3	0.177	0.004483	3 L1 _	Ralgds
Skap21	2.07E-07	0.250095	0.265	0.143	0.004162	3 L1 _	Skap2
Fam129b1	1.95E-07	0.311903	0.346	0.211	0.003931	3 L1 _	Fam129b
Raly	1.75E-07	0.32452	0.665	0.57	0.003531	3 L1 _	Raly
Gatad2a1	1.49E-07	0.251715	0.465	0.315	0.002996	3 L1 _	Gatad2a
Mcmbp	1.44E-07	0.305829	0.385	0.249	0.0029	3 L1 _	Mcmbp
Tap11	1.37E-07	0.28557	0.458	0.307	0.002756	3 L1 _	Tap1
R3hdm1	1.34E-07	0.287381	0.569	0.424	0.002704	3 L1 _	R3hdm1
Smarca41	1.34E-07	0.321312	0.396	0.258	0.002694	3 L1 _	Smarca4
Stim21	1.27E-07	0.324597	0.385	0.244	0.002566	3 L1 _	Stim2
Mgat11	1.18E-07	0.33823	0.45	0.319	0.002382	3 L1 _	Mgat1
Sdcbp1	1.14E-07	0.35774	0.712	0.608	0.002289	3 L1 _	Sdcbp
Btbd7	1.11E-07	0.358735	0.692	0.561	0.002227	3 L1 _	Btbd7
Rarg	1.09E-07	0.300886	0.431	0.277	0.002205	3 L1 _	Rarg
Frmd4b	1.06E-07	0.289809	0.262	0.141	0.002136	3 L1 _	Frmd4b
Fam117b1	1.00E-07	0.28561	0.362	0.219	0.002023	3 L1 _	Fam117b
Map4k31	9.46E-08	0.35819	0.358	0.216	0.001906	3 L1 _	Map4k3
Fam53b1	9.46E-08	0.297162	0.438	0.282	0.001905	3 L1 _	Fam53b
Tet3	9.07E-08	0.378621	0.396	0.261	0.001827	3 L1 _	Tet3
Afap11	8.95E-08	0.317983	0.531	0.38	0.001802	3 L1 _	Afap1
Pomp	8.84E-08	0.373376	0.827	0.756	0.00178	3 L1 _	Pomp
Tax1bp31	8.40E-08	0.290451	0.542	0.395	0.001691	3 L1 _	Tax1bp3
Ube2v1	8.35E-08	0.289477	0.546	0.403	0.001682	3 L1 _	Ube2v1
Cdk19	8.15E-08	0.274862	0.304	0.173	0.001641	3 L1 _	Cdk19
Gm26802	7.11E-08	0.347648	0.177	0.079	0.001431	3 L1 _	Gm26802
Mcl12	6.74E-08	0.345602	0.719	0.619	0.001357	3 L1 _	Mcl1
Rock21	6.67E-08	0.287042	0.773	0.658	0.001343	3 L1 _	Rock2

Gcn1	6.63E-08	0.26268	0.381	0.23	0.001335	3 L1 _	Gcn1
Rbm391	6.40E-08	0.250339	0.931	0.917	0.001289	3 L1 _	Rbm39
Bloc1s6	6.33E-08	0.275846	0.281	0.154	0.001275	3 L1 _	Bloc1s6
Socs5	5.89E-08	0.277931	0.454	0.296	0.001186	3 L1 _	Socs5
Fmo22	5.88E-08	0.30992	0.623	0.447	0.001185	3 L1 _	Fmo2
Nr1h2	5.59E-08	0.284914	0.454	0.304	0.001127	3 L1 _	Nr1h2
Nsd1	5.26E-08	0.34423	0.723	0.602	0.001059	3 L1 _	Nsd1
Osbpl9	5.16E-08	0.353632	0.542	0.395	0.00104	3 L1 _	Osbpl9
Mid2	5.05E-08	0.263113	0.231	0.113	0.001016	3 L1 _	Mid2
Atp6v1e1	5.04E-08	0.350914	0.685	0.549	0.001015	3 L1 _	Atp6v1e1
Map3k12	4.66E-08	0.339883	0.269	0.147	0.000938	3 L1 _	Map3k1
Trim2	4.48E-08	0.446788	0.377	0.231	0.000903	3 L1 _	Trim2
Rnf19a1	4.44E-08	0.275839	0.338	0.196	0.000893	3 L1 _	Rnf19a
Rap1a2	4.40E-08	0.3315	0.765	0.639	0.000886	3 L1 _	Rap1a
Pea15a1	4.31E-08	0.290876	0.696	0.553	0.000869	3 L1 _	Pea15a
Pik3c2a1	4.00E-08	0.336531	0.431	0.28	0.000805	3 L1 _	Pik3c2a
Baz2b1	3.71E-08	0.429817	0.519	0.386	0.000746	3 L1 _	Baz2b
Nkapd1	3.34E-08	0.275265	0.304	0.167	0.000672	3 L1 _	Nkapd1
Tasor	3.23E-08	0.382526	0.5	0.336	0.00065	3 L1 _	Tasor
Rilpl1	3.17E-08	0.297696	0.315	0.18	0.000638	3 L1 _	Rilpl1
Larp1b1	2.91E-08	0.250754	0.246	0.123	0.000585	3 L1 _	Larp1b
Etnk11	2.86E-08	0.314555	0.592	0.423	0.000575	3 L1 _	Etnk1
Hspg23	2.68E-08	0.270386	0.846	0.681	0.000539	3 L1 _	Hspg2
Klc11	2.64E-08	0.316894	0.512	0.35	0.000532	3 L1 _	Klc1
Ccnl2	2.59E-08	0.368381	0.565	0.406	0.000522	3 L1 _	Ccnl2
Dstyk	2.34E-08	0.37681	0.438	0.291	0.000472	3 L1 _	Dstyk
Nod11	2.34E-08	0.274292	0.265	0.139	0.000472	3 L1 _	Nod1
3222401L13Rik	2.29E-08	0.269633	0.173	0.073	0.000462	3 L1 _	3222401L13Rik
Eif4g22	2.10E-08	0.30568	0.838	0.773	0.000423	3 L1 _	Eif4g2
Cdipt1	2.10E-08	0.404955	0.431	0.289	0.000423	3 L1 _	Cdipt
Kctd101	2.01E-08	0.291603	0.469	0.307	0.000404	3 L1 _	Kctd10
Ciao2b	1.84E-08	0.281239	0.454	0.293	0.00037	3 L1 _	Ciao2b
Tmem30a1	1.73E-08	0.36358	0.715	0.598	0.000348	3 L1 _	Tmem30a
Map3k3	1.60E-08	0.274072	0.312	0.174	0.000322	3 L1 _	Map3k3
Cebpg1	1.39E-08	0.380734	0.527	0.391	0.000281	3 L1 _	Cebpg
Tsc22d12	1.32E-08	0.303218	0.946	0.821	0.000265	3 L1 _	Tsc22d1
Pdzd2	1.31E-08	0.301435	0.338	0.187	0.000264	3 L1 _	Pdzd2
Micall1	1.30E-08	0.261874	0.231	0.113	0.000261	3 L1 _	Micall1
St6galnac2	1.17E-08	0.272102	0.281	0.149	0.000236	3 L1 _	St6galnac2
Tmtc11	1.11E-08	0.295687	0.192	0.082	0.000224	3 L1 _	Tmtc1
Xdh2	9.58E-09	0.392158	0.573	0.416	0.000193	3 L1 _	Xdh

Diaph21	9.49E-09	0.330272	0.542	0.387	0.000191	3 L1 _	Diaph2
Plekha1	8.75E-09	0.370843	0.404	0.249	0.000176	3 L1 _	Plekha1
Ints91	7.36E-09	0.277938	0.246	0.12	0.000148	3 L1 _	Ints9
Baiap21	6.80E-09	0.266404	0.269	0.138	0.000137	3 L1 _	Baiap2
Nmi1	6.74E-09	0.364881	0.454	0.301	0.000136	3 L1 _	Nmi
Rab13	6.65E-09	0.410193	0.523	0.371	0.000134	3 L1 _	Rab13
St3gal41	6.39E-09	0.338152	0.362	0.208	0.000129	3 L1 _	St3gal4
Ifi203	6.28E-09	0.36345	0.838	0.711	0.000126	3 L1 _	Ifi203
Tpm42	6.11E-09	0.356819	0.854	0.744	0.000123	3 L1 _	Tpm4
Tgfb11	6.03E-09	0.31322	0.642	0.458	0.000121	3 L1 _	Tgfb1
Fbxo221	5.68E-09	0.330378	0.427	0.266	0.000114	3 L1 _	Fbxo22
Magi3	5.65E-09	0.281948	0.254	0.125	0.000114	3 L1 _	Magi3
Fos1	5.35E-09	0.759581	0.627	0.487	0.000108	3 L1 _	Fos
Meis2	4.81E-09	0.451504	0.527	0.369	9.68E-05	3 L1 _	Meis2
Dgkz	4.35E-09	0.292536	0.358	0.208	8.76E-05	3 L1 _	Dgkz
Slc39a10	4.34E-09	0.372699	0.377	0.223	8.75E-05	3 L1 _	Slc39a10
Gnb2	4.14E-09	0.333649	0.877	0.854	8.33E-05	3 L1 _	Gnb2
Fam171a11	4.11E-09	0.293454	0.285	0.147	8.28E-05	3 L1 _	Fam171a1
Bag3	4.00E-09	0.337938	0.408	0.244	8.07E-05	3 L1 _	Bag3
Exoc61	3.92E-09	0.275934	0.2	0.087	7.90E-05	3 L1 _	Exoc6
Ywhae1	3.90E-09	0.328919	0.869	0.775	7.86E-05	3 L1 _	Ywhae
Arhgap171	3.83E-09	0.362648	0.485	0.328	7.71E-05	3 L1 _	Arhgap17
Hprt1	3.27E-09	0.39608	0.473	0.316	6.59E-05	3 L1 _	Hprt
Ccdc88c1	3.26E-09	0.327409	0.235	0.109	6.57E-05	3 L1 _	Ccdc88c
Pdlim12	3.17E-09	0.329415	0.412	0.243	6.39E-05	3 L1 _	Pdlim1
Rbms11	3.13E-09	0.331173	0.858	0.77	6.30E-05	3 L1 _	Rbms1
Phf20l1	3.04E-09	0.414167	0.65	0.509	6.13E-05	3 L1 _	Phf20l1
Atp5j	2.95E-09	0.326625	0.927	0.882	5.95E-05	3 L1 _	Atp5j
Ehd22	2.80E-09	0.368121	0.619	0.471	5.63E-05	3 L1 _	Ehd2
B230219D22Rik2	2.72E-09	0.358935	0.785	0.7	5.47E-05	3 L1 _	B230219D22Rik
Slk1	2.71E-09	0.412458	0.608	0.464	5.46E-05	3 L1 _	Slk
Gbp2	2.62E-09	0.353844	0.508	0.344	5.28E-05	3 L1 _	Gbp2
Card191	2.11E-09	0.406247	0.438	0.279	4.25E-05	3 L1 _	Card19
Palm1	2.07E-09	0.385347	0.388	0.234	4.16E-05	3 L1 _	Palm
Rab5c1	1.82E-09	0.39062	0.696	0.572	3.66E-05	3 L1 _	Rab5c
Klhl24	1.79E-09	0.431948	0.642	0.499	3.61E-05	3 L1 _	Klhl24
Zfp36l1	1.68E-09	0.38101	0.881	0.823	3.39E-05	3 L1 _	Zfp36l1
Dnm2	1.66E-09	0.43049	0.531	0.365	3.34E-05	3 L1 _	Dnm2
Dazap2	1.62E-09	0.430323	0.6	0.452	3.27E-05	3 L1 _	Dazap2
Trim56	1.61E-09	0.449493	0.481	0.321	3.23E-05	3 L1 _	Trim56
Fut81	1.56E-09	0.472609	0.465	0.314	3.14E-05	3 L1 _	Fut8

Igtp	1.49E-09	0.36521	0.354	0.201	3.00E-05	3 L1 _	Igtp
Arhgef2	1.42E-09	0.384998	0.485	0.328	2.85E-05	3 L1 _	Arhgef2
Tmpo1	1.31E-09	0.291729	0.527	0.338	2.63E-05	3 L1 _	Tmpo
Arpc51	1.29E-09	0.32129	0.808	0.67	2.60E-05	3 L1 _	Arpc5
Cds22	1.24E-09	0.35057	0.458	0.287	2.50E-05	3 L1 _	Cds2
S100a11	1.11E-09	0.34336	0.608	0.425	2.23E-05	3 L1 _	S100a1
Pnkd	1.07E-09	0.385583	0.612	0.452	2.16E-05	3 L1 _	Pnkd
Casp81	1.05E-09	0.370274	0.358	0.206	2.11E-05	3 L1 _	Casp8
Kmt2e	1.00E-09	0.397605	0.823	0.76	2.02E-05	3 L1 _	Kmt2e
Map2k3	9.99E-10	0.295642	0.377	0.212	2.01E-05	3 L1 _	Map2k3
Trib22	9.63E-10	0.42052	0.512	0.336	1.94E-05	3 L1 _	Trib2
Stat1	9.28E-10	0.404453	0.558	0.394	1.87E-05	3 L1 _	Stat1
Stard9	9.05E-10	0.381271	0.408	0.248	1.82E-05	3 L1 _	Stard9
Atp6v0c2	8.44E-10	0.289779	0.846	0.66	1.70E-05	3 L1 _	Atp6v0c
Ndufc21	8.30E-10	0.358185	0.812	0.699	1.67E-05	3 L1 _	Ndufc2
Cenpb	7.65E-10	0.432521	0.669	0.527	1.54E-05	3 L1 _	Cenpb
Stx21	7.25E-10	0.421847	0.412	0.252	1.46E-05	3 L1 _	Stx2
Mfap3	7.11E-10	0.335099	0.338	0.182	1.43E-05	3 L1 _	Mfap3
Midn1	6.63E-10	0.357397	0.512	0.343	1.34E-05	3 L1 _	Midn
Apc1	6.43E-10	0.375559	0.5	0.337	1.30E-05	3 L1 _	Apc
Mrpl17	6.38E-10	0.427574	0.592	0.439	1.28E-05	3 L1 _	Mrpl17
Clstn12	6.21E-10	0.331197	0.7	0.553	1.25E-05	3 L1 _	Clstn1
Sh3glb12	5.88E-10	0.35926	0.892	0.804	1.18E-05	3 L1 _	Sh3glb1
Ahnak3	5.80E-10	0.368287	0.973	0.885	1.17E-05	3 L1 _	Ahnak
Zswim6	5.63E-10	0.285732	0.223	0.097	1.13E-05	3 L1 _	Zswim6
Tmem63b1	4.69E-10	0.270163	0.258	0.123	9.45E-06	3 L1 _	Tmem63b
Gna111	4.53E-10	0.408731	0.723	0.603	9.13E-06	3 L1 _	Gna11
Taf7	4.49E-10	0.27588	0.419	0.239	9.04E-06	3 L1 _	Taf7
Prrg21	4.46E-10	0.301985	0.223	0.097	8.98E-06	3 L1 _	Prrg2
Txnip1	4.38E-10	0.33352	0.927	0.846	8.83E-06	3 L1 _	Txnip
Tecpr11	4.01E-10	0.318954	0.435	0.264	8.07E-06	3 L1 _	Tecpr1
Cdc42ep3	3.55E-10	0.397458	0.388	0.231	7.15E-06	3 L1 _	Cdc42ep3
Snn	2.56E-10	0.259806	0.15	0.052	5.16E-06	3 L1 _	Snn
Psme21	2.48E-10	0.365548	0.738	0.611	5.00E-06	3 L1 _	Psme2
Unc119b1	2.36E-10	0.349779	0.385	0.222	4.75E-06	3 L1 _	Unc119b
Phc2	2.02E-10	0.371597	0.454	0.29	4.06E-06	3 L1 _	Phc2
Ifi351	1.98E-10	0.42801	0.554	0.383	3.98E-06	3 L1 _	Ifi35
Pelo	1.86E-10	0.306038	0.358	0.191	3.75E-06	3 L1 _	Pelo
Ddit41	1.78E-10	0.343421	0.292	0.145	3.59E-06	3 L1 _	Ddit4
Eml1	1.64E-10	0.398301	0.312	0.161	3.30E-06	3 L1 _	Eml1
Emp21	1.29E-10	0.49564	0.692	0.546	2.59E-06	3 L1 _	Emp2

Pfkfb31	1.25E-10	0.258224	0.135	0.042	2.51E-06	3 L1 _	Pfkfb3
Mtss1	1.19E-10	0.370331	0.312	0.159	2.39E-06	3 L1 _	Mtss1
Map4k4	1.13E-10	0.484359	0.742	0.627	2.28E-06	3 L1 _	Map4k4
Stmn11	1.08E-10	0.258878	0.227	0.096	2.18E-06	3 L1 _	Stmn1
Asap12	1.07E-10	0.3741	0.769	0.6	2.16E-06	3 L1 _	Asap1
Ndst1	1.02E-10	0.459828	0.446	0.285	2.06E-06	3 L1 _	Ndst1
Actg12	9.14E-11	0.290428	0.969	0.942	1.84E-06	3 L1 _	Actg1
Slc50a1	8.10E-11	0.465903	0.738	0.632	1.63E-06	3 L1 _	Slc50a1
Rtp4	7.59E-11	0.362951	0.519	0.318	1.53E-06	3 L1 _	Rtp4
Tcn21	7.54E-11	0.544235	0.654	0.505	1.52E-06	3 L1 _	Tcn2
Ptk22	7.36E-11	0.468677	0.512	0.332	1.48E-06	3 L1 _	Ptk2
Rps6ka31	7.21E-11	0.460739	0.462	0.286	1.45E-06	3 L1 _	Rps6ka3
Rell1	6.08E-11	0.365668	0.358	0.189	1.22E-06	3 L1 _	Rell1
Prickle3	6.00E-11	0.322099	0.242	0.107	1.21E-06	3 L1 _	Prickle3
Triobp	5.90E-11	0.370737	0.431	0.257	1.19E-06	3 L1 _	Triobp
Atp2b42	5.74E-11	0.32246	0.496	0.291	1.16E-06	3 L1 _	Atp2b4
Sod12	5.55E-11	0.421546	0.688	0.515	1.12E-06	3 L1 _	Sod1
Pdlim51	5.16E-11	0.439845	0.485	0.304	1.04E-06	3 L1 _	Pdlim5
Maco1	4.69E-11	0.361055	0.331	0.172	9.44E-07	3 L1 _	Maco1
Tubb4b2	4.64E-11	0.491988	0.804	0.69	9.35E-07	3 L1 _	Tubb4b
Ptpn121	4.56E-11	0.472659	0.492	0.315	9.19E-07	3 L1 _	Ptpn12
Rac11	4.46E-11	0.383496	0.835	0.751	8.97E-07	3 L1 _	Rac1
Abhd17a	4.19E-11	0.46177	0.642	0.496	8.43E-07	3 L1 _	Abhd17a
Itsn21	4.00E-11	0.454199	0.481	0.296	8.05E-07	3 L1 _	Itsn2
Colgalt21	3.88E-11	0.307275	0.25	0.108	7.81E-07	3 L1 _	Colgalt2
Snx31	3.66E-11	0.430438	0.788	0.683	7.37E-07	3 L1 _	Snx3
Trim30a1	3.55E-11	0.461081	0.465	0.285	7.15E-07	3 L1 _	Trim30a
Map4	2.49E-11	0.545965	0.638	0.494	5.01E-07	3 L1 _	Map4
Ier21	2.48E-11	0.61162	0.608	0.441	4.99E-07	3 L1 _	Ier2
Fyn	2.39E-11	0.407667	0.573	0.38	4.81E-07	3 L1 _	Fyn
Rap1b1	2.20E-11	0.440944	0.785	0.674	4.43E-07	3 L1 _	Rap1b
4930581F22Rik1	2.05E-11	0.328027	0.323	0.16	4.12E-07	3 L1 _	4930581F22Rik
Kif5b1	2.00E-11	0.465938	0.785	0.668	4.02E-07	3 L1 _	Kif5b
Mmd1	1.90E-11	0.292408	0.208	0.082	3.83E-07	3 L1 _	Mmd
Hip1r	1.88E-11	0.277872	0.212	0.083	3.78E-07	3 L1 _	Hip1r
Nop53	1.69E-11	0.54326	0.642	0.48	3.40E-07	3 L1 _	Nop53
Atp8b1	1.62E-11	0.428936	0.369	0.206	3.25E-07	3 L1 _	Atp8b1
Ccrl2	1.54E-11	0.318043	0.292	0.135	3.11E-07	3 L1 _	Ccrl2
Tnfaip21	1.40E-11	0.442526	0.6	0.409	2.83E-07	3 L1 _	Tnfaip2
Rasa4	1.08E-11	0.25462	0.188	0.066	2.17E-07	3 L1 _	Rasa4
Cox7a2l1	1.04E-11	0.43629	0.85	0.738	2.09E-07	3 L1 _	Cox7a2l

Cyb5r31	8.89E-12	0.456752	0.719	0.566	1.79E-07	3 L1 _	Cyb5r3
Kmt2a	8.73E-12	0.378616	0.742	0.551	1.76E-07	3 L1 _	Kmt2a
Myl12b1	8.27E-12	0.402873	0.869	0.787	1.67E-07	3 L1 _	Myl12b
Aopep	8.13E-12	0.342618	0.523	0.325	1.64E-07	3 L1 _	Aopep
Cd472	8.05E-12	0.39911	0.919	0.856	1.62E-07	3 L1 _	Cd47
Zhx32	7.65E-12	0.474642	0.492	0.311	1.54E-07	3 L1 _	Zhx3
Pdgfd1	7.04E-12	0.322611	0.242	0.101	1.42E-07	3 L1 _	Pdgfd
Gnb11	5.82E-12	0.419702	0.827	0.678	1.17E-07	3 L1 _	Gnb1
Mndal2	5.65E-12	0.443681	0.815	0.65	1.14E-07	3 L1 _	Mndal
Gle1	5.62E-12	0.34945	0.4	0.217	1.13E-07	3 L1 _	Gle1
Bok2	5.46E-12	0.261977	0.292	0.131	1.10E-07	3 L1 _	Bok
Ndufa81	4.94E-12	0.436656	0.735	0.589	9.95E-08	3 L1 _	Ndufa8
Elf12	4.91E-12	0.494578	0.619	0.447	9.89E-08	3 L1 _	Elf1
Cr1l	4.61E-12	0.528651	0.688	0.521	9.28E-08	3 L1 _	Cr1l
Tapbp1	4.37E-12	0.451368	0.765	0.624	8.80E-08	3 L1 _	Tapbp
Zeb11	4.25E-12	0.427636	0.738	0.592	8.56E-08	3 L1 _	Zeb1
Cyb5611	4.22E-12	0.294631	0.227	0.091	8.50E-08	3 L1 _	Cyb561
Rnasek1	3.71E-12	0.425039	0.796	0.571	7.47E-08	3 L1 _	Rnasek
Atp1a11	3.68E-12	0.453657	0.596	0.422	7.40E-08	3 L1 _	Atp1a1
Lats21	3.31E-12	0.423998	0.5	0.317	6.66E-08	3 L1 _	Lats2
Ninj1	3.22E-12	0.348858	0.488	0.292	6.48E-08	3 L1 _	Ninj1
Isg15	2.81E-12	0.508441	0.531	0.321	5.66E-08	3 L1 _	Isg15
Sgk31	2.81E-12	0.297622	0.273	0.119	5.65E-08	3 L1 _	Sgk3
Itm2b1	2.64E-12	0.345752	1	0.989	5.31E-08	3 L1 _	Itm2b
Chd71	2.53E-12	0.316964	0.242	0.096	5.10E-08	3 L1 _	Chd7
Sema3c2	2.28E-12	0.414106	0.254	0.105	4.60E-08	3 L1 _	Sema3c
Ostf13	2.14E-12	0.472957	0.746	0.594	4.32E-08	3 L1 _	Ostf1
Arl6ip51	2.08E-12	0.444268	0.669	0.487	4.19E-08	3 L1 _	Arl6ip5
Hes11	2.00E-12	0.454049	0.762	0.581	4.04E-08	3 L1 _	Hes1
Znfx11	1.98E-12	0.456008	0.423	0.241	3.98E-08	3 L1 _	Znfx1
Hras1	1.85E-12	0.394192	0.615	0.42	3.72E-08	3 L1 _	Hras
Ipo111	1.85E-12	0.366348	0.262	0.113	3.72E-08	3 L1 _	Ipo11
Epha41	1.83E-12	0.379387	0.354	0.176	3.69E-08	3 L1 _	Epha4
Sntb21	1.68E-12	0.458523	0.708	0.532	3.39E-08	3 L1 _	Sntb2
Nuak1	1.67E-12	0.379214	0.285	0.127	3.35E-08	3 L1 _	Nuak1
Zdhhc182	1.54E-12	0.465817	0.373	0.191	3.09E-08	3 L1 _	Zdhhc18
Pkn11	1.50E-12	0.447713	0.473	0.274	3.02E-08	3 L1 _	Pkn1
Tmem1401	1.38E-12	0.47414	0.585	0.397	2.78E-08	3 L1 _	Tmem140
Ro601	1.35E-12	0.375661	0.469	0.27	2.73E-08	3 L1 _	Ro60
Mrpl53	1.35E-12	0.40374	0.45	0.256	2.71E-08	3 L1 _	Mrpl53
Cyp26b12	1.19E-12	0.507047	0.242	0.094	2.39E-08	3 L1 _	Cyp26b1

Cyp2d22	1.14E-12	0.440528	0.55	0.361	2.30E-08	3 L1 _	Cyp2d22
Adipor11	1.13E-12	0.429587	0.592	0.414	2.28E-08	3 L1 _	Adipor1
Wwc21	1.10E-12	0.404413	0.638	0.448	2.22E-08	3 L1 _	Wwc2
Gm40701	1.10E-12	0.400639	0.231	0.091	2.21E-08	3 L1 _	Gm4070
Trim472	1.06E-12	0.452063	0.581	0.385	2.13E-08	3 L1 _	Trim47
Peak11	8.05E-13	0.458633	0.754	0.616	1.62E-08	3 L1 _	Peak1
Dab21	6.76E-13	0.453117	0.604	0.404	1.36E-08	3 L1 _	Dab2
Ncoa71	6.71E-13	0.403869	0.558	0.348	1.35E-08	3 L1 _	Ncoa7
Tmem2042	6.53E-13	0.444269	0.758	0.558	1.32E-08	3 L1 _	Tmem204
Golm11	6.38E-13	0.455694	0.362	0.185	1.29E-08	3 L1 _	Golm1
Ccnyl11	5.89E-13	0.301943	0.2	0.07	1.19E-08	3 L1 _	Ccnyl1
Irf2bpl1	5.69E-13	0.491204	0.658	0.492	1.15E-08	3 L1 _	Irf2bpl
Pwwp3a	5.40E-13	0.398436	0.431	0.23	1.09E-08	3 L1 _	Pwwp3a
Slfn21	5.09E-13	0.602995	0.504	0.302	1.02E-08	3 L1 _	Slfn2
Cdc42ep2	5.06E-13	0.493455	0.542	0.357	1.02E-08	3 L1 _	Cdc42ep2
Ppfibp1	4.43E-13	0.509674	0.592	0.402	8.93E-09	3 L1 _	Ppfibp1
H2-T22	4.25E-13	0.570246	0.692	0.501	8.56E-09	3 L1 _	H2-T22
Timeless1	3.71E-13	0.310305	0.246	0.098	7.47E-09	3 L1 _	Timeless
A930005H10Rik	3.69E-13	0.306787	0.2	0.069	7.42E-09	3 L1 _	A930005H10Rik
Dchs1	3.47E-13	0.311382	0.315	0.145	6.98E-09	3 L1 _	Dchs1
Vamp31	3.37E-13	0.427858	0.492	0.302	6.78E-09	3 L1 _	Vamp3
Ier51	2.99E-13	0.509093	0.669	0.489	6.02E-09	3 L1 _	Ier5
Sp1001	2.98E-13	0.597596	0.696	0.532	6.01E-09	3 L1 _	Sp100
Tnfsf13	2.86E-13	0.292408	0.338	0.156	5.75E-09	3 L1 _	Tnfsf13
Cox5a1	2.77E-13	0.422096	0.869	0.756	5.57E-09	3 L1 _	Cox5a
Prrg1	2.04E-13	0.402417	0.288	0.126	4.11E-09	3 L1 _	Prrg1
Dusp13	1.85E-13	0.593369	0.523	0.316	3.73E-09	3 L1 _	Dusp1
Enpp4	1.85E-13	0.415864	0.281	0.12	3.72E-09	3 L1 _	Enpp4
Stard41	1.57E-13	0.364561	0.231	0.088	3.16E-09	3 L1 _	Stard4
Endod11	1.55E-13	0.495383	0.554	0.359	3.12E-09	3 L1 _	Endod1
Zfyve9	1.40E-13	0.350721	0.404	0.204	2.82E-09	3 L1 _	Zfyve9
Nedd92	1.31E-13	0.581465	0.527	0.33	2.63E-09	3 L1 _	Nedd9
Tmod31	1.02E-13	0.507363	0.75	0.594	2.06E-09	3 L1 _	Tmod3
Oaz22	8.41E-14	0.568961	0.619	0.426	1.69E-09	3 L1 _	Oaz2
Gja11	7.14E-14	0.727819	0.519	0.333	1.44E-09	3 L1 _	Gja1
Flnb	7.08E-14	0.429321	0.438	0.246	1.42E-09	3 L1 _	Flnb
Sorbs31	7.06E-14	0.340482	0.323	0.147	1.42E-09	3 L1 _	Sorbs3
Rgs3	6.89E-14	0.563195	0.577	0.384	1.39E-09	3 L1 _	Rgs3
Zmiz11	6.71E-14	0.499134	0.673	0.493	1.35E-09	3 L1 _	Zmiz1
Cavin12	6.05E-14	0.364121	0.696	0.458	1.22E-09	3 L1 _	Cavin1
Cdc42ep11	6.00E-14	0.358035	0.458	0.247	1.21E-09	3 L1 _	Cdc42ep1

Phactr41	5.71E-14	0.449926	0.423	0.221	1.15E-09	3 L1 _	Phactr4
Samhd1	4.96E-14	0.522587	0.608	0.419	1.00E-09	3 L1 _	Samhd1
Lgals9	3.84E-14	0.491709	0.692	0.498	7.73E-10	3 L1 _	Lgals9
Rgl21	3.73E-14	0.33847	0.342	0.16	7.52E-10	3 L1 _	Rgl2
Ctnnd11	3.30E-14	0.464163	0.519	0.32	6.64E-10	3 L1 _	Ctnnd1
Agrn1	3.10E-14	0.369115	0.381	0.185	6.24E-10	3 L1 _	Agrn
Peg13	3.09E-14	0.382562	0.335	0.149	6.22E-10	3 L1 _	Peg13
Psmb101	2.67E-14	0.479091	0.742	0.563	5.37E-10	3 L1 _	Psmb10
Sppl2a1	2.01E-14	0.514071	0.696	0.505	4.05E-10	3 L1 _	Sppl2a
Atrx	1.99E-14	0.55143	0.877	0.769	4.00E-10	3 L1 _	Atrx
Ier31	1.98E-14	0.507687	0.327	0.145	3.98E-10	3 L1 _	Ier3
Dock11	1.86E-14	0.498037	0.562	0.362	3.75E-10	3 L1 _	Dock1
Rps141	1.62E-14	0.299021	0.985	0.972	3.26E-10	3 L1 _	Rps14
Ripor11	1.38E-14	0.444669	0.458	0.25	2.78E-10	3 L1 _	Ripor1
Cmip1	1.35E-14	0.462109	0.45	0.245	2.72E-10	3 L1 _	Cmip
Exoc3l4	1.30E-14	0.483961	0.431	0.233	2.61E-10	3 L1 _	Exoc3l4
Dlc13	1.16E-14	0.440322	0.823	0.691	2.34E-10	3 L1 _	Dlc1
Dgkh1	1.05E-14	0.490906	0.404	0.203	2.11E-10	3 L1 _	Dgkh
Cep85l1	8.60E-15	0.324375	0.292	0.118	1.73E-10	3 L1 _	Cep85l
Dock61	8.24E-15	0.415573	0.373	0.176	1.66E-10	3 L1 _	Dock6
Slco2a11	7.86E-15	0.541528	0.308	0.134	1.58E-10	3 L1 _	Slco2a1
Gch11	7.28E-15	0.311484	0.196	0.063	1.47E-10	3 L1 _	Gch1
Sh3bgrl4	6.93E-15	0.520238	0.819	0.671	1.40E-10	3 L1 _	Sh3bgrl
Slc3a22	6.62E-15	0.543179	0.723	0.57	1.33E-10	3 L1 _	Slc3a2
Iqgap11	6.12E-15	0.441628	0.854	0.746	1.23E-10	3 L1 _	Iqgap1
0610010K14Rik	5.96E-15	0.47777	0.531	0.314	1.20E-10	3 L1 _	0610010K14Rik
Plec	5.89E-15	0.498795	0.731	0.548	1.19E-10	3 L1 _	Plec
Swap702	5.82E-15	0.43334	0.585	0.358	1.17E-10	3 L1 _	Swap70
Ubald2	5.44E-15	0.487763	0.615	0.414	1.09E-10	3 L1 _	Ubald2
Reep32	4.73E-15	0.481405	0.823	0.682	9.52E-11	3 L1 _	Reep3
Trim25	4.42E-15	0.497635	0.442	0.238	8.89E-11	3 L1 _	Trim25
Gbp3	4.20E-15	0.391122	0.377	0.177	8.47E-11	3 L1 _	Gbp3
Cxcl121	3.89E-15	0.885087	0.854	0.786	7.84E-11	3 L1 _	Cxcl12
Dleu22	3.09E-15	0.473697	0.465	0.244	6.23E-11	3 L1 _	Dleu2
Hsp90ab1	2.96E-15	0.331416	0.992	0.979	5.95E-11	3 L1 _	Hsp90ab1
Tns21	2.53E-15	0.460186	0.55	0.327	5.09E-11	3 L1 _	Tns2
Phlpp11	2.42E-15	0.34441	0.342	0.151	4.87E-11	3 L1 _	Phlpp1
Tspan141	2.38E-15	0.353368	0.238	0.086	4.80E-11	3 L1 _	Tspan14
Eogt1	2.21E-15	0.329868	0.2	0.062	4.45E-11	3 L1 _	Eogt
Ifitm32	2.21E-15	0.36825	0.996	0.933	4.45E-11	3 L1 _	Ifitm3
Spata132	1.93E-15	0.315909	0.25	0.091	3.88E-11	3 L1 _	Spata13

H2-T231	1.84E-15	0.593093	0.715	0.513	3.71E-11	3 L1 _	H2-T23
Tap2	1.70E-15	0.527638	0.508	0.29	3.42E-11	3 L1 _	Tap2
Cavin3	1.61E-15	0.459101	0.881	0.716	3.24E-11	3 L1 _	Cavin3
Sox41	1.56E-15	0.674258	0.615	0.419	3.15E-11	3 L1 _	Sox4
Dennd5b1	1.54E-15	0.471016	0.415	0.204	3.09E-11	3 L1 _	Dennd5b
Efhd12	1.32E-15	0.256675	0.138	0.031	2.65E-11	3 L1 _	Efhd1
Tagln2	1.23E-15	0.462539	0.877	0.789	2.48E-11	3 L1 _	Tagln2
Cpne81	1.20E-15	0.58864	0.519	0.315	2.41E-11	3 L1 _	Cpne8
Phldb21	1.07E-15	0.488722	0.496	0.28	2.15E-11	3 L1 _	Phldb2
Pola2	9.21E-16	0.400715	0.304	0.126	1.85E-11	3 L1 _	Pola2
Rnf213	6.86E-16	0.677032	0.5	0.284	1.38E-11	3 L1 _	Rnf213
Ptbp32	4.83E-16	0.563648	0.75	0.57	9.74E-12	3 L1 _	Ptbp3
Ralgapa11	4.06E-16	0.50753	0.519	0.305	8.17E-12	3 L1 _	Ralgapa1
Cables2	3.92E-16	0.307743	0.227	0.075	7.90E-12	3 L1 _	Cables2
St3gal51	3.74E-16	0.258175	0.165	0.043	7.53E-12	3 L1 _	St3gal5
Fam174b	3.55E-16	0.375971	0.215	0.069	7.16E-12	3 L1 _	Fam174b
Slco2b11	3.31E-16	0.389893	0.358	0.159	6.67E-12	3 L1 _	Slco2b1
Wwtr11	3.10E-16	0.498563	0.765	0.572	6.25E-12	3 L1 _	Wwtr1
Ppm1f1	2.44E-16	0.260936	0.231	0.076	4.91E-12	3 L1 _	Ppm1f
Dcbld11	2.18E-16	0.351775	0.204	0.061	4.39E-12	3 L1 _	Dcbld1
Plekhg51	2.03E-16	0.304071	0.258	0.093	4.09E-12	3 L1 _	Plekhg5
Hmcn1	1.93E-16	0.607594	0.523	0.303	3.90E-12	3 L1 _	Hmcn1
Ifi471	1.85E-16	0.514889	0.496	0.256	3.73E-12	3 L1 _	Ifi47
Vgll41	1.80E-16	0.575117	0.677	0.444	3.63E-12	3 L1 _	Vgll4
Cpd2	1.73E-16	0.56692	0.673	0.461	3.48E-12	3 L1 _	Cpd
Ace4	1.64E-16	0.563039	0.608	0.372	3.29E-12	3 L1 _	Ace
Xaf1	1.43E-16	0.575361	0.596	0.363	2.87E-12	3 L1 _	Xaf1
Nes3	8.43E-17	0.398385	0.446	0.206	1.70E-12	3 L1 _	Nes
Chst7	8.27E-17	0.297041	0.188	0.053	1.67E-12	3 L1 _	Chst7
Abcc42	7.64E-17	0.348595	0.246	0.083	1.54E-12	3 L1 _	Abcc4
Prkce1	7.51E-17	0.330294	0.269	0.097	1.51E-12	3 L1 _	Prkce
Mxd41	6.24E-17	0.537009	0.873	0.763	1.26E-12	3 L1 _	Mxd4
Gpr182	6.16E-17	0.272677	0.15	0.034	1.24E-12	3 L1 _	Gpr182
Arhgef7	5.54E-17	0.507478	0.512	0.281	1.12E-12	3 L1 _	Arhgef7
Slc27a31	4.73E-17	0.331058	0.177	0.047	9.53E-13	3 L1 _	Slc27a3
Tut71	4.65E-17	0.58411	0.627	0.401	9.36E-13	3 L1 _	Tut7
Ralb	3.99E-17	0.489039	0.519	0.291	8.04E-13	3 L1 _	Ralb
Sorbs12	3.75E-17	0.273425	0.362	0.147	7.55E-13	3 L1 _	Sorbs1
Gm20559	3.53E-17	0.448248	0.496	0.259	7.10E-13	3 L1 _	Gm20559
Klf10	3.48E-17	0.45408	0.446	0.22	7.02E-13	3 L1 _	Klf10
Slc28a21	3.25E-17	0.300588	0.165	0.041	6.55E-13	3 L1 _	Slc28a2

Arhgap18	3.03E-17	0.671691	0.554	0.329	6.10E-13	3 L1 _	Arhgap18
Dennd31	2.85E-17	0.304459	0.192	0.053	5.73E-13	3 L1 _	Dennd3
Xiap1	2.72E-17	0.542794	0.715	0.504	5.48E-13	3 L1 _	Xiap
Irf7	2.18E-17	0.626969	0.612	0.369	4.40E-13	3 L1 _	Irf7
Rnf1522	1.84E-17	0.30105	0.223	0.066	3.70E-13	3 L1 _	Rnf152
Lbr1	1.52E-17	0.450234	0.419	0.194	3.07E-13	3 L1 _	Lbr
Myo102	1.48E-17	0.564408	0.65	0.429	2.98E-13	3 L1 _	Myo10
Fnbp1l1	1.43E-17	0.461735	0.392	0.182	2.89E-13	3 L1 _	Fnbp1l
Foxo11	1.40E-17	0.524671	0.488	0.263	2.83E-13	3 L1 _	Foxo1
Hey11	1.16E-17	0.340915	0.15	0.033	2.34E-13	3 L1 _	Hey1
Arhgef32	1.11E-17	0.395971	0.269	0.094	2.24E-13	3 L1 _	Arhgef3
Il10rb1	8.33E-18	0.522212	0.581	0.33	1.68E-13	3 L1 _	Il10rb
Vwa11	6.17E-18	0.55588	0.562	0.311	1.24E-13	3 L1 _	Vwa1
Btg3	4.81E-18	0.474275	0.477	0.242	9.68E-14	3 L1 _	Btg3
Sos11	4.18E-18	0.529746	0.388	0.177	8.42E-14	3 L1 _	Sos1
Cemip2	2.91E-18	0.43747	0.438	0.202	5.85E-14	3 L1 _	Cemip2
Esm11	2.88E-18	0.706663	0.146	0.03	5.81E-14	3 L1 _	Esm1
Zfp6641	2.59E-18	0.460322	0.523	0.282	5.22E-14	3 L1 _	Zfp664
Samd9l1	2.53E-18	0.690527	0.585	0.347	5.10E-14	3 L1 _	Samd9l
Rhoc	2.13E-18	0.593767	0.831	0.702	4.28E-14	3 L1 _	Rhoc
Ktn13	2.11E-18	0.485164	0.792	0.605	4.26E-14	3 L1 _	Ktn1
Fat41	1.99E-18	0.438464	0.258	0.086	4.00E-14	3 L1 _	Fat4
Nfe2l11	1.63E-18	0.599422	0.8	0.622	3.27E-14	3 L1 _	Nfe2l1
Psme12	1.43E-18	0.574822	0.854	0.714	2.88E-14	3 L1 _	Psme1
Ifi213	9.05E-19	0.358929	0.165	0.038	1.82E-14	3 L1 _	Ifi213
Actn42	8.15E-19	0.484077	0.838	0.705	1.64E-14	3 L1 _	Actn4
Sh3bgrl21	7.89E-19	0.303194	0.162	0.036	1.59E-14	3 L1 _	Sh3bgrl2
Gng113	7.53E-19	0.345787	0.935	0.806	1.52E-14	3 L1 _	Gng11
Pggt1b	5.86E-19	0.539807	0.477	0.249	1.18E-14	3 L1 _	Pggt1b
Kctd12	5.46E-19	0.610954	0.742	0.531	1.10E-14	3 L1 _	Kctd12
Gnaq2	5.34E-19	0.566541	0.665	0.419	1.07E-14	3 L1 _	Gnaq
Prpf40b	5.18E-19	0.475823	0.281	0.1	1.04E-14	3 L1 _	Prpf40b
Ier5l	3.45E-19	0.583974	0.55	0.306	6.95E-15	3 L1 _	Ier5l
Serinc31	3.40E-19	0.497123	0.931	0.851	6.85E-15	3 L1 _	Serinc3
Hhex1	3.18E-19	0.38684	0.227	0.066	6.40E-15	3 L1 _	Hhex
Oip5os1	2.41E-19	0.499712	0.681	0.426	4.86E-15	3 L1 _	Oip5os1
N4bp1	2.25E-19	0.546743	0.508	0.27	4.53E-15	3 L1 _	N4bp1
Hdac72	1.85E-19	0.550153	0.662	0.426	3.72E-15	3 L1 _	Hdac7
Fry3	1.74E-19	0.343036	0.415	0.164	3.51E-15	3 L1 _	Fry
Ptp4a32	1.45E-19	0.556546	0.554	0.299	2.92E-15	3 L1 _	Ptp4a3
Cd813	1.38E-19	0.45361	0.981	0.886	2.79E-15	3 L1 _	Cd81

Mcam2	1.30E-19	0.361158	0.346	0.125	2.63E-15	3 L1 _	Mcam
Parp141	1.24E-19	0.6567	0.623	0.363	2.49E-15	3 L1 _	Parp14
Gimap82	1.13E-19	0.496664	0.304	0.108	2.27E-15	3 L1 _	Gimap8
Arl151	8.63E-20	0.445721	0.304	0.108	1.74E-15	3 L1 _	Arl15
Tmcc31	6.82E-20	0.507698	0.362	0.143	1.37E-15	3 L1 _	Tmcc3
Myo1c1	6.78E-20	0.600429	0.627	0.391	1.37E-15	3 L1 _	Myo1c
Gbp71	5.97E-20	0.709728	0.665	0.428	1.20E-15	3 L1 _	Gbp7
Git11	5.43E-20	0.440457	0.408	0.176	1.09E-15	3 L1 _	Git1
Phf11d	5.03E-20	0.54015	0.488	0.236	1.01E-15	3 L1 _	Phf11d
Lyn1	3.39E-20	0.46676	0.4	0.167	6.83E-16	3 L1 _	Lyn
Dhrs32	3.25E-20	0.625129	0.635	0.395	6.55E-16	3 L1 _	Dhrs3
Gnas3	2.75E-20	0.36395	0.981	0.959	5.54E-16	3 L1 _	Gnas
Rapgef11	2.63E-20	0.495048	0.431	0.192	5.30E-16	3 L1 _	Rapgef1
Lnx21	2.44E-20	0.376145	0.227	0.062	4.92E-16	3 L1 _	Lnx2
Taok2	2.32E-20	0.627515	0.488	0.258	4.67E-16	3 L1 _	Taok2
Gda2	2.21E-20	0.368295	0.231	0.065	4.45E-16	3 L1 _	Gda
Fryl1	2.08E-20	0.62382	0.469	0.231	4.18E-16	3 L1 _	Fryl
Mast4	1.94E-20	0.706082	0.708	0.486	3.92E-16	3 L1 _	Mast4
Wars1	1.43E-20	0.520144	0.4	0.174	2.89E-16	3 L1 _	Wars
Zbtb46	1.35E-20	0.390502	0.369	0.146	2.72E-16	3 L1 _	Zbtb46
Cd1513	1.30E-20	0.631816	0.758	0.537	2.61E-16	3 L1 _	Cd151
Fxyd51	1.22E-20	0.684979	0.892	0.73	2.46E-16	3 L1 _	Fxyd5
Piezo22	1.21E-20	0.580234	0.596	0.331	2.43E-16	3 L1 _	Piezo2
4833403J16Rik1	1.18E-20	0.285851	0.192	0.045	2.38E-16	3 L1 _	4833403J16Rik
B2m2	1.18E-20	0.462734	0.985	0.947	2.38E-16	3 L1 _	B2m
Tgtp2	1.16E-20	0.392844	0.181	0.041	2.34E-16	3 L1 _	Tgtp2
Crybg31	1.09E-20	0.597903	0.481	0.236	2.20E-16	3 L1 _	Crybg3
Syne23	1.03E-20	0.640398	0.658	0.393	2.07E-16	3 L1 _	Syne2
Zfpm12	9.79E-21	0.427648	0.392	0.16	1.97E-16	3 L1 _	Zfpm1
Nck11	9.13E-21	0.662186	0.735	0.498	1.84E-16	3 L1 _	Nck1
Ctnna11	8.75E-21	0.503056	0.815	0.654	1.76E-16	3 L1 _	Ctnna1
Nfkbia1	8.66E-21	0.69599	0.762	0.516	1.74E-16	3 L1 _	Nfkbia
Qk1	8.24E-21	0.6713	0.808	0.667	1.66E-16	3 L1 _	Qk
Dusp31	8.06E-21	0.585605	0.592	0.332	1.62E-16	3 L1 _	Dusp3
Tmed51	7.88E-21	0.640896	0.638	0.387	1.59E-16	3 L1 _	Tmed5
Gbp5	7.46E-21	0.450569	0.281	0.09	1.50E-16	3 L1 _	Gbp5
Dnm31	7.34E-21	0.682003	0.477	0.238	1.48E-16	3 L1 _	Dnm3
Cmah1	5.44E-21	0.385308	0.181	0.039	1.10E-16	3 L1 _	Cmah
Calcrl1	5.18E-21	0.593732	0.55	0.287	1.04E-16	3 L1 _	Calcrl
Yes1	3.26E-21	0.538871	0.404	0.171	6.56E-17	3 L1 _	Yes1
Sypl1	2.88E-21	0.661259	0.685	0.446	5.80E-17	3 L1 _	Sypl

Ccnd12	2.88E-21	0.351716	0.4	0.15	5.79E-17	3 L1 _	Ccnd1
Ankrd291	2.86E-21	0.254254	0.188	0.042	5.75E-17	3 L1 _	Ankrd29
Tspan153	2.25E-21	0.313759	0.4	0.144	4.52E-17	3 L1 _	Tspan15
Tinagl13	1.81E-21	0.251409	0.635	0.269	3.65E-17	3 L1 _	Tinagl1
Trpv41	1.73E-21	0.298976	0.131	0.02	3.48E-17	3 L1 _	Trpv4
Sema6d3	1.32E-21	0.629488	0.612	0.328	2.66E-17	3 L1 _	Sema6d
Ptma2	1.15E-21	0.379125	0.996	0.986	2.32E-17	3 L1 _	Ptma
Gcnt23	1.08E-21	0.338774	0.45	0.176	2.18E-17	3 L1 _	Gcnt2
Rapgef22	9.08E-22	0.424131	0.469	0.199	1.83E-17	3 L1 _	Rapgef2
Tmsb4x3	9.08E-22	0.352099	1	0.994	1.83E-17	3 L1 _	Tmsb4x
Ppp1r16b2	7.71E-22	0.417581	0.292	0.092	1.55E-17	3 L1 _	Ppp1r16b
2-Jun	6.77E-22	0.9215	0.865	0.676	1.36E-17	3 L1 _	Jun
Myo61	6.58E-22	0.874373	0.558	0.33	1.33E-17	3 L1 _	Myo6
Prdm161	6.50E-22	0.345375	0.181	0.039	1.31E-17	3 L1 _	Prdm16
Sh2d3c2	6.39E-22	0.486027	0.35	0.127	1.29E-17	3 L1 _	Sh2d3c
Tns13	5.63E-22	0.530782	0.781	0.503	1.13E-17	3 L1 _	Tns1
Pde8a2	4.99E-22	0.546427	0.423	0.175	1.00E-17	3 L1 _	Pde8a
Stard83	4.74E-22	0.466306	0.45	0.186	9.54E-18	3 L1 _	Stard8
Tnfaip8l1	4.35E-22	0.264359	0.127	0.018	8.75E-18	3 L1 _	Tnfaip8l1
Adamts91	4.10E-22	0.579422	0.404	0.166	8.26E-18	3 L1 _	Adamts9
Sipa1l21	3.83E-22	0.3647	0.269	0.079	7.71E-18	3 L1 _	Sipa1l2
Glul1	3.69E-22	1.161216	0.512	0.273	7.44E-18	3 L1 _	Glul
Ifi2081	3.63E-22	0.470298	0.219	0.055	7.32E-18	3 L1 _	Ifi208
Adam101	3.24E-22	0.622445	0.554	0.296	6.52E-18	3 L1 _	Adam10
Plcg11	3.20E-22	0.637093	0.477	0.23	6.44E-18	3 L1 _	Plcg1
Ppia1	2.77E-22	0.404726	1	0.982	5.59E-18	3 L1 _	Ppia
Tbkbp1	2.04E-22	0.418308	0.262	0.076	4.10E-18	3 L1 _	Tbkbp1
Castor1	1.71E-22	0.446421	0.315	0.105	3.44E-18	3 L1 _	Castor1
Als2cl1	1.70E-22	0.310282	0.196	0.043	3.43E-18	3 L1 _	Als2cl
Fez23	1.58E-22	0.568264	0.642	0.348	3.17E-18	3 L1 _	Fez2
Lmo71	1.40E-22	0.384239	0.196	0.044	2.83E-18	3 L1 _	Lmo7
Arhgef121	1.36E-22	0.601549	0.838	0.65	2.75E-18	3 L1 _	Arhgef12
Elmo12	1.22E-22	0.552093	0.35	0.127	2.45E-18	3 L1 _	Elmo1
Ccdc28b1	1.10E-22	0.567748	0.585	0.314	2.22E-18	3 L1 _	Ccdc28b
Lrrc3b1	7.39E-23	0.303633	0.115	0.014	1.49E-18	3 L1 _	Lrrc3b
Hs3st11	6.18E-23	0.324293	0.165	0.03	1.25E-18	3 L1 _	Hs3st1
Pcdh121	5.05E-23	0.5768	0.365	0.139	1.02E-18	3 L1 _	Pcdh12
Cdc42bpb1	4.52E-23	0.603795	0.546	0.283	9.10E-19	3 L1 _	Cdc42bpb
Bst21	4.28E-23	0.784054	0.896	0.693	8.62E-19	3 L1 _	Bst2
Plekhg11	3.63E-23	0.485366	0.4	0.161	7.32E-19	3 L1 _	Plekhg1
H2-Q71	3.12E-23	0.642166	0.773	0.487	6.29E-19	3 L1 _	H2-Q7

Bend71	2.99E-23	0.281745	0.165	0.03	6.02E-19	3 L1 _	Bend7
Bambi	2.97E-23	0.31386	0.181	0.037	5.97E-19	3 L1 _	Bambi
H2-Q4	2.36E-23	0.67499	0.531	0.263	4.76E-19	3 L1 _	H2-Q4
Rab11a1	2.18E-23	0.672322	0.673	0.415	4.40E-19	3 L1 _	Rab11a
S100a162	2.18E-23	0.617899	0.842	0.567	4.39E-19	3 L1 _	S100a16
Fmnl31	2.01E-23	0.580728	0.535	0.27	4.05E-19	3 L1 _	Fmnl3
Git21	1.09E-23	0.70084	0.638	0.381	2.20E-19	3 L1 _	Git2
Hmg20b	1.05E-23	0.647247	0.731	0.498	2.11E-19	3 L1 _	Hmg20b
Piezo11	8.35E-24	0.490501	0.323	0.109	1.68E-19	3 L1 _	Piezo1
Cldn15	7.72E-24	0.372132	0.146	0.023	1.55E-19	3 L1 _	Cldn15
Macf11	6.80E-24	0.700189	0.923	0.798	1.37E-19	3 L1 _	Macf1
Ablim12	6.55E-24	0.724873	0.638	0.369	1.32E-19	3 L1 _	Ablim1
Tpm32	5.87E-24	0.630862	0.885	0.736	1.18E-19	3 L1 _	Tpm3
Ndufa42	4.59E-24	0.633442	0.892	0.717	9.25E-20	3 L1 _	Ndufa4
H2-Q61	3.82E-24	0.773807	0.535	0.263	7.69E-20	3 L1 _	H2-Q6
Smad1	3.28E-24	0.689175	0.45	0.198	6.62E-20	3 L1 _	Smad1
Prkd21	2.94E-24	0.438761	0.342	0.117	5.93E-20	3 L1 _	Prkd2
Flywch11	2.46E-24	0.674589	0.535	0.274	4.96E-20	3 L1 _	Flywch1
Aig12	2.30E-24	0.606545	0.469	0.199	4.63E-20	3 L1 _	Aig1
Plcb42	2.24E-24	0.565047	0.512	0.227	4.52E-20	3 L1 _	Plcb4
St3gal62	1.31E-24	0.617791	0.473	0.204	2.63E-20	3 L1 _	St3gal6
Arhgef51	9.59E-25	0.564823	0.404	0.157	1.93E-20	3 L1 _	Arhgef5
Hyal21	9.52E-25	0.46092	0.331	0.107	1.92E-20	3 L1 _	Hyal2
Nrp21	5.34E-25	0.635305	0.608	0.317	1.08E-20	3 L1 _	Nrp2
9930111J21Rik22	3.83E-25	0.657186	0.696	0.41	7.71E-21	3 L1 _	9930111J21Rik2
Map3k111	3.80E-25	0.437946	0.296	0.087	7.66E-21	3 L1 _	Map3k11
Psmb92	3.43E-25	0.59974	0.662	0.349	6.91E-21	3 L1 _	Psmb9
Thsd11	2.66E-25	0.407883	0.215	0.048	5.35E-21	3 L1 _	Thsd1
S100a131	2.46E-25	0.621497	0.896	0.741	4.95E-21	3 L1 _	S100a13
Abi32	2.02E-25	0.621196	0.431	0.167	4.07E-21	3 L1 _	Abi3
Nhsl21	1.80E-25	0.619915	0.438	0.177	3.62E-21	3 L1 _	Nhsl2
Rasal22	1.79E-25	0.61453	0.604	0.303	3.60E-21	3 L1 _	Rasal2
Pcdh13	1.69E-25	0.465555	0.415	0.151	3.40E-21	3 L1 _	Pcdh1
Pitpnc11	1.68E-25	0.741171	0.631	0.342	3.39E-21	3 L1 _	Pitpnc1
Acer21	1.46E-25	0.266947	0.192	0.036	2.94E-21	3 L1 _	Acer2
Atp8a11	1.46E-25	0.534591	0.331	0.109	2.94E-21	3 L1 _	Atp8a1
2-Mar	1.43E-25	0.813416	0.762	0.535	2.88E-21	3 L1 _	2-Mar
Plscr11	1.07E-25	0.720119	0.423	0.172	2.16E-21	3 L1 _	Plscr1
Myzap	1.03E-25	0.259258	0.138	0.018	2.08E-21	3 L1 _	Myzap
Pik3r6	4.17E-26	0.254783	0.142	0.019	8.39E-22	3 L1 _	Pik3r6
Lpcat11	4.02E-26	0.658059	0.438	0.177	8.10E-22	3 L1 _	Lpcat1

Gimap52	3.04E-26	0.475964	0.477	0.183	6.12E-22	3 L1 _	Gimap5
Numb1	2.05E-26	0.666926	0.523	0.238	4.12E-22	3 L1 _	Numb
Bnip21	2.02E-26	0.705873	0.773	0.567	4.07E-22	3 L1 _	Bnip2
Iigp12	1.83E-26	0.737146	0.696	0.391	3.69E-22	3 L1 _	Iigp1
Lysmd2	1.66E-26	0.638994	0.585	0.287	3.34E-22	3 L1 _	Lysmd2
Csgalnact11	1.23E-26	0.519415	0.419	0.154	2.47E-22	3 L1 _	Csgalnact1
Galnt151	8.90E-27	0.45012	0.346	0.108	1.79E-22	3 L1 _	Galnt15
Inka1	7.63E-27	0.538243	0.419	0.159	1.54E-22	3 L1 _	Inka1
Grap3	7.22E-27	0.39215	0.369	0.12	1.45E-22	3 L1 _	Grap
Exoc3l1	7.16E-27	0.262498	0.154	0.022	1.44E-22	3 L1 _	Exoc3l
Irf12	6.81E-27	0.653751	0.619	0.325	1.37E-22	3 L1 _	Irf1
Mapk121	5.73E-27	0.473678	0.365	0.121	1.15E-22	3 L1 _	Mapk12
Timp33	4.18E-27	0.653792	0.965	0.77	8.41E-23	3 L1 _	Timp3
Apold12	2.47E-27	0.442844	0.258	0.06	4.98E-23	3 L1 _	Apold1
Luzp11	2.35E-27	0.709793	0.8	0.575	4.73E-23	3 L1 _	Luzp1
Tcf43	1.82E-27	0.576539	0.969	0.879	3.67E-23	3 L1 _	Tcf4
Sptan11	1.70E-27	0.747377	0.838	0.647	3.43E-23	3 L1 _	Sptan1
Anp32a1	1.44E-27	0.739368	0.831	0.644	2.89E-23	3 L1 _	Anp32a
Clic41	1.08E-27	0.705377	0.812	0.557	2.17E-23	3 L1 _	Clic4
H2-D11	9.64E-28	0.610173	0.981	0.951	1.94E-23	3 L1 _	H2-D1
Gbp91	9.44E-28	0.592091	0.415	0.155	1.90E-23	3 L1 _	Gbp9
Dab2ip3	8.20E-28	0.726351	0.75	0.466	1.65E-23	3 L1 _	Dab2ip
Car71	7.22E-28	0.321751	0.162	0.023	1.45E-23	3 L1 _	Car7
Slc30a11	6.43E-28	0.536718	0.331	0.099	1.30E-23	3 L1 _	Slc30a1
Bmpr21	5.69E-28	0.755427	0.846	0.624	1.15E-23	3 L1 _	Bmpr2
Bik	5.33E-28	0.330607	0.208	0.039	1.07E-23	3 L1 _	Bik
Fam13c	3.63E-28	0.607624	0.492	0.204	7.31E-24	3 L1 _	Fam13c
Gipc31	2.29E-28	0.26852	0.15	0.019	4.62E-24	3 L1 _	Gipc3
Pxn1	2.17E-28	0.572778	0.462	0.179	4.36E-24	3 L1 _	Pxn
Fgd61	1.68E-28	0.326978	0.2	0.036	3.39E-24	3 L1 _	Fgd6
Foxf11	1.55E-28	0.315521	0.162	0.023	3.13E-24	3 L1 _	Foxf1
Alpl1	1.48E-28	0.659157	0.246	0.056	2.97E-24	3 L1 _	Alpl
Cd2ap1	1.19E-28	0.757309	0.8	0.548	2.40E-24	3 L1 _	Cd2ap
Tpst22	8.73E-29	0.750568	0.569	0.274	1.76E-24	3 L1 _	Tpst2
Klhl51	8.56E-29	0.599477	0.504	0.21	1.72E-24	3 L1 _	Klhl5
Gimap42	5.90E-29	0.533345	0.623	0.268	1.19E-24	3 L1 _	Gimap4
St8sia42	5.07E-29	0.630916	0.496	0.188	1.02E-24	3 L1 _	St8sia4
Rgs121	4.04E-29	0.494316	0.365	0.117	8.13E-25	3 L1 _	Rgs12
Casz11	2.91E-29	0.283563	0.146	0.017	5.85E-25	3 L1 _	Casz1
Klhl4	2.51E-29	0.26938	0.131	0.013	5.06E-25	3 L1 _	Klhl4
Magi11	1.51E-29	0.436849	0.219	0.042	3.04E-25	3 L1 _	Magi1

Tspan91	8.50E-30	0.678486	0.565	0.263	1.71E-25	3 L1 _	Tspan9
Col4a13	6.13E-30	0.777864	0.981	0.86	1.23E-25	3 L1 _	Col4a1
Col4a22	4.27E-30	0.755172	0.954	0.844	8.61E-26	3 L1 _	Col4a2
Clec2d1	2.90E-30	0.82268	0.858	0.625	5.85E-26	3 L1 _	Clec2d
Pim31	2.87E-30	0.595291	0.412	0.139	5.78E-26	3 L1 _	Pim3
Tmem441	2.20E-30	0.396181	0.192	0.031	4.44E-26	3 L1 _	Tmem44
H2-K11	9.77E-31	0.621424	0.996	0.927	1.97E-26	3 L1 _	H2-K1
Lama51	8.92E-31	0.391172	0.242	0.049	1.80E-26	3 L1 _	Lama5
Arpc31	6.92E-31	0.704379	0.923	0.785	1.39E-26	3 L1 _	Arpc3
Tanc11	4.24E-31	0.683962	0.596	0.287	8.54E-27	3 L1 _	Tanc1
Sec14l11	4.01E-31	0.736257	0.623	0.314	8.07E-27	3 L1 _	Sec14l1
Mcc1	2.88E-31	0.564247	0.419	0.143	5.79E-27	3 L1 _	Mcc
Jcad1	2.55E-31	0.712669	0.496	0.204	5.14E-27	3 L1 _	Jcad
Rgl11	2.20E-31	0.850763	0.715	0.433	4.43E-27	3 L1 _	Rgl1
Tal11	2.15E-31	0.263243	0.131	0.012	4.33E-27	3 L1 _	Tal1
Klf71	2.10E-31	0.831934	0.777	0.522	4.23E-27	3 L1 _	Klf7
Efnb11	9.36E-32	0.737685	0.515	0.207	1.89E-27	3 L1 _	Efnb1
Gm34455	8.00E-32	0.549005	0.273	0.061	1.61E-27	3 L1 _	Gm34455
Hoxd81	7.80E-32	0.400472	0.254	0.053	1.57E-27	3 L1 _	Hoxd8
Slfn31	7.01E-32	0.306247	0.138	0.013	1.41E-27	3 L1 _	Slfn3
Sybu1	5.25E-32	0.422387	0.2	0.032	1.06E-27	3 L1 _	Sybu
Ttll72	4.43E-32	0.474687	0.304	0.072	8.93E-28	3 L1 _	Ttll7
B3gnt31	3.22E-32	0.385627	0.25	0.051	6.49E-28	3 L1 _	B3gnt3
Dusp71	2.83E-32	0.643306	0.462	0.171	5.70E-28	3 L1 _	Dusp7
Enah1	2.59E-32	0.581869	0.296	0.069	5.22E-28	3 L1 _	Enah
Fzd43	1.57E-32	0.571744	0.708	0.304	3.16E-28	3 L1 _	Fzd4
Sgk11	1.46E-32	0.92132	0.581	0.256	2.95E-28	3 L1 _	Sgk1
Slc26a101	1.37E-32	0.528543	0.196	0.029	2.76E-28	3 L1 _	Slc26a10
Foxp11	1.25E-32	0.840813	0.804	0.526	2.51E-28	3 L1 _	Foxp1
Ybx11	1.05E-32	0.674979	0.985	0.899	2.11E-28	3 L1 _	Ybx1
Klf3	1.03E-32	0.816944	0.715	0.432	2.07E-28	3 L1 _	Klf3
Gm326881	9.88E-33	0.308807	0.177	0.023	1.99E-28	3 L1 _	Gm32688
Sox132	9.74E-33	0.41097	0.296	0.067	1.96E-28	3 L1 _	Sox13
Psmb82	8.75E-33	0.815095	0.869	0.604	1.76E-28	3 L1 _	Psmb8
Tdrp1	7.80E-33	0.376836	0.235	0.043	1.57E-28	3 L1 _	Tdrp
2900026A02Rik1	5.43E-33	0.797683	0.585	0.27	1.09E-28	3 L1 _	2900026A02Rik
Utrn3	4.60E-33	0.858421	0.769	0.456	9.26E-29	3 L1 _	Utrn
Nrp11	3.68E-33	0.825451	0.942	0.783	7.41E-29	3 L1 _	Nrp1
Smad72	2.37E-33	0.847812	0.723	0.374	4.78E-29	3 L1 _	Smad7
Epb41l4a	1.49E-33	0.276275	0.138	0.012	3.01E-29	3 L1 _	Epb41l4a
Wasf21	1.49E-33	0.745053	0.865	0.71	3.01E-29	3 L1 _	Wasf2

Ptpn141	9.47E-34	0.594228	0.354	0.098	1.91E-29	3 L1 _	Ptpn14
Rtl8b1	8.79E-34	0.758486	0.573	0.26	1.77E-29	3 L1 _	Rtl8b
Tjp11	8.43E-34	0.807372	0.681	0.364	1.70E-29	3 L1 _	Tjp1
Stox21	7.42E-34	0.709925	0.481	0.184	1.49E-29	3 L1 _	Stox2
Fkbp1a1	2.91E-34	0.748618	0.915	0.77	5.85E-30	3 L1 _	Fkbp1a
Stc11	1.65E-34	0.670517	0.396	0.115	3.32E-30	3 L1 _	Stc1
Adarb11	1.56E-34	0.774066	0.431	0.146	3.13E-30	3 L1 _	Adarb1
Sigirr2	1.41E-34	0.421786	0.304	0.069	2.84E-30	3 L1 _	Sigirr
Shroom41	1.25E-34	0.455113	0.223	0.038	2.51E-30	3 L1 _	Shroom4
Gas62	1.21E-34	0.818468	0.842	0.536	2.44E-30	3 L1 _	Gas6
6430548M08Rik1	4.99E-35	0.502846	0.3	0.068	1.00E-30	3 L1 _	6430548M08Rik
Hoxd4	4.92E-35	0.32073	0.142	0.012	9.91E-31	3 L1 _	Hoxd4
Slc12a71	4.87E-35	0.46485	0.273	0.056	9.81E-31	3 L1 _	Slc12a7
Ggta12	2.94E-35	0.572302	0.369	0.098	5.93E-31	3 L1 _	Ggta1
Hip11	2.79E-35	0.807686	0.662	0.318	5.62E-31	3 L1 _	Hip1
Ivns1abp1	2.75E-35	1.095433	0.6	0.287	5.53E-31	3 L1 _	Ivns1abp
App3	2.20E-35	0.72015	0.965	0.824	4.43E-31	3 L1 _	App
Fabp52	2.18E-35	0.833811	0.562	0.215	4.39E-31	3 L1 _	Fabp5
Sncaip1	2.04E-35	0.346524	0.173	0.02	4.10E-31	3 L1 _	Sncaip
Rnf1251	1.98E-35	0.422655	0.277	0.056	3.99E-31	3 L1 _	Rnf125
Caskin21	1.59E-35	0.565662	0.412	0.12	3.21E-31	3 L1 _	Caskin2
Jam33	1.55E-35	0.864614	0.708	0.368	3.12E-31	3 L1 _	Jam3
Ctnnb11	1.32E-35	0.835968	0.869	0.682	2.65E-31	3 L1 _	Ctnnb1
Stom2	1.12E-35	0.648009	0.438	0.135	2.25E-31	3 L1 _	Stom
Tox31	8.33E-36	0.335576	0.142	0.012	1.68E-31	3 L1 _	Tox3
Thbd2	7.44E-36	0.790638	0.715	0.379	1.50E-31	3 L1 _	Thbd
Eps8l2	7.15E-36	0.306525	0.138	0.011	1.44E-31	3 L1 _	Eps8l2
Grasp1	2.68E-36	0.744923	0.515	0.201	5.40E-32	3 L1 _	Grasp
Tnfrsf10b1	1.52E-36	0.425259	0.219	0.033	3.07E-32	3 L1 _	Tnfrsf10b
Tbxa2r1	1.44E-36	0.425379	0.25	0.044	2.90E-32	3 L1 _	Tbxa2r
Efr3b1	1.24E-36	0.400606	0.231	0.037	2.49E-32	3 L1 _	Efr3b
F11r1	1.13E-36	0.487343	0.412	0.115	2.27E-32	3 L1 _	F11r
Hspa12b1	7.42E-37	0.572215	0.281	0.057	1.49E-32	3 L1 _	Hspa12b
N4bp3	7.37E-37	0.462085	0.258	0.047	1.48E-32	3 L1 _	N4bp3
Ptprg1	7.31E-37	0.840816	0.727	0.385	1.47E-32	3 L1 _	Ptprg
Arhgap271	5.68E-37	0.498325	0.338	0.081	1.14E-32	3 L1 _	Arhgap27
Plxnd11	5.37E-37	0.81936	0.685	0.346	1.08E-32	3 L1 _	Plxnd1
Mapk31	5.07E-37	0.798672	0.808	0.517	1.02E-32	3 L1 _	Mapk3
Abcb1a1	3.06E-37	0.606902	0.331	0.079	6.17E-33	3 L1 _	Abcb1a
Inhbb	2.19E-37	0.639222	0.273	0.053	4.41E-33	3 L1 _	Inhbb
Lrrc8c2	1.74E-37	0.705138	0.573	0.209	3.50E-33	3 L1 _	Lrrc8c

Hecw21	1.63E-37	0.726517	0.469	0.153	3.29E-33	3 L1 _	Hecw2
Prdm11	3.72E-38	0.536246	0.319	0.068	7.49E-34	3 L1 _	Prdm1
Tgm21	3.68E-38	0.905963	0.608	0.273	7.42E-34	3 L1 _	Tgm2
Tspan71	3.28E-38	0.587216	0.25	0.043	6.61E-34	3 L1 _	Tspan7
Hmbox11	2.49E-38	0.795539	0.585	0.236	5.01E-34	3 L1 _	Hmbox1
Stab1	1.35E-38	0.48793	0.296	0.059	2.72E-34	3 L1 _	Stab1
Dock91	1.09E-38	0.861163	0.527	0.193	2.19E-34	3 L1 _	Dock9
Mpzl1	9.34E-39	0.788754	0.623	0.275	1.88E-34	3 L1 _	Mpzl1
Nxn	6.66E-39	0.827636	0.538	0.211	1.34E-34	3 L1 _	Nxn
Sema3g1	4.78E-39	0.718394	0.196	0.024	9.62E-35	3 L1 _	Sema3g
9530082P21Rik1	3.33E-39	0.581737	0.346	0.081	6.71E-35	3 L1 _	9530082P21Rik
Gbp42	2.85E-39	0.79751	0.481	0.153	5.73E-35	3 L1 _	Gbp4
Syt151	1.65E-39	0.486257	0.212	0.027	3.33E-35	3 L1 _	Syt15
Ephb11	8.88E-40	0.327344	0.165	0.014	1.79E-35	3 L1 _	Ephb1
Endou1	5.42E-40	0.334029	0.204	0.025	1.09E-35	3 L1 _	Endou
Slfn52	3.04E-40	0.960364	0.923	0.691	6.12E-36	3 L1 _	Slfn5
St8sia6	2.96E-40	0.322604	0.138	0.008	5.95E-36	3 L1 _	St8sia6
Pkp41	2.67E-40	0.627941	0.431	0.125	5.37E-36	3 L1 _	Pkp4
Klf131	2.18E-40	0.941877	0.831	0.519	4.40E-36	3 L1 _	Klf13
Heg12	1.95E-40	0.845776	0.681	0.297	3.93E-36	3 L1 _	Heg1
Rai141	1.18E-40	0.865247	0.681	0.318	2.37E-36	3 L1 _	Rai14
Pik3c2b	9.98E-41	0.480446	0.246	0.038	2.01E-36	3 L1 _	Pik3c2b
Mfng1	9.32E-41	0.579044	0.331	0.071	1.88E-36	3 L1 _	Mfng
Ctnnbip11	7.64E-41	0.869761	0.588	0.244	1.54E-36	3 L1 _	Ctnnbip1
Slc43a32	5.26E-41	1.07024	0.8	0.473	1.06E-36	3 L1 _	Slc43a3
Tnfaip12	3.35E-41	0.954152	0.692	0.343	6.75E-37	3 L1 _	Tnfaip1
Slc6a61	2.84E-41	1.235762	0.673	0.327	5.73E-37	3 L1 _	Slc6a6
Ptgs11	2.20E-41	0.88614	0.588	0.238	4.44E-37	3 L1 _	Ptgs1
Rtp31	1.57E-41	0.507898	0.223	0.029	3.16E-37	3 L1 _	Rtp3
Shank31	1.24E-41	0.413359	0.25	0.038	2.50E-37	3 L1 _	Shank3
Ephb41	1.12E-41	0.67657	0.435	0.125	2.26E-37	3 L1 _	Ephb4
Ltbp42	4.82E-42	0.946622	0.9	0.644	9.70E-38	3 L1 _	Ltbp4
Impdh11	3.92E-42	0.708553	0.458	0.138	7.89E-38	3 L1 _	Impdh1
Card101	3.17E-42	0.593523	0.392	0.099	6.38E-38	3 L1 _	Card10
Ppp1r13b1	2.90E-42	0.65856	0.392	0.099	5.85E-38	3 L1 _	Ppp1r13b
Galnt18	1.93E-42	0.503734	0.281	0.047	3.90E-38	3 L1 _	Galnt18
Rtl8a1	1.07E-42	1.043428	0.781	0.452	2.16E-38	3 L1 _	Rtl8a
Cbfa2t32	9.34E-43	0.738722	0.604	0.207	1.88E-38	3 L1 _	Cbfa2t3
Acvrl11	7.41E-43	0.880887	0.723	0.368	1.49E-38	3 L1 _	Acvrl1
Msn2	7.07E-43	0.989224	0.935	0.78	1.42E-38	3 L1 _	Msn
Spns21	5.54E-43	0.413893	0.242	0.034	1.12E-38	3 L1 _	Spns2

Stmn2	5.51E-43	0.982882	0.212	0.025	1.11E-38	3 L1 _	Stmn2
Itga13	3.52E-43	0.905432	0.962	0.78	7.10E-39	3 L1 _	Itga1
Filip12	3.43E-43	0.881675	0.5	0.157	6.91E-39	3 L1 _	Filip1
Kitl3	3.16E-43	1.123323	0.75	0.367	6.37E-39	3 L1 _	Kitl
Ifi441	2.35E-43	1.062504	0.473	0.143	4.74E-39	3 L1 _	Ifi44
Gngt21	2.20E-43	0.812125	0.6	0.22	4.44E-39	3 L1 _	Gngt2
St6galnac31	1.80E-43	0.461208	0.242	0.033	3.63E-39	3 L1 _	St6galnac3
Proser21	1.57E-43	0.49973	0.258	0.038	3.16E-39	3 L1 _	Proser2
Itpkb1	1.29E-43	1.01999	0.723	0.364	2.60E-39	3 L1 _	Itpkb
Ackr32	1.18E-43	0.620891	0.396	0.092	2.38E-39	3 L1 _	Ackr3
B3gnt21	1.15E-43	0.969529	0.704	0.34	2.32E-39	3 L1 _	B3gnt2
Notch11	1.13E-43	0.892647	0.692	0.306	2.28E-39	3 L1 _	Notch1
Lpar61	6.78E-44	0.952892	0.5	0.158	1.37E-39	3 L1 _	Lpar6
Pitpnm21	3.04E-44	0.624807	0.415	0.106	6.12E-40	3 L1 _	Pitpnm2
Rassf31	2.96E-44	0.635856	0.404	0.097	5.95E-40	3 L1 _	Rassf3
Ptprm1	1.79E-44	0.707527	0.462	0.132	3.60E-40	3 L1 _	Ptprm
Prex21	9.38E-45	0.917459	0.758	0.377	1.89E-40	3 L1 _	Prex2
Insr	6.91E-45	1.097194	0.531	0.182	1.39E-40	3 L1 _	Insr
Pkn31	5.15E-45	0.446945	0.227	0.027	1.04E-40	3 L1 _	Pkn3
Abcg21	3.39E-45	1.033868	0.681	0.308	6.82E-41	3 L1 _	Abcg2
Mmp151	2.85E-45	0.46968	0.319	0.057	5.74E-41	3 L1 _	Mmp15
Cldn5	1.71E-45	0.994721	0.212	0.023	3.45E-41	3 L1 _	Cldn5
Sh3bp51	2.75E-46	0.943042	0.577	0.218	5.54E-42	3 L1 _	Sh3bp5
Hspb13	8.29E-47	1.123523	0.888	0.568	1.67E-42	3 L1 _	Hspb1
Car82	2.87E-47	0.54415	0.281	0.041	5.77E-43	3 L1 _	Car8
Ablim31	2.29E-47	0.392955	0.181	0.013	4.62E-43	3 L1 _	Ablim3
Apbb21	2.15E-47	0.999127	0.792	0.434	4.33E-43	3 L1 _	Apbb2
Bvht1	1.71E-47	0.717288	0.477	0.134	3.44E-43	3 L1 _	Bvht
Crim12	1.29E-47	0.852373	0.673	0.25	2.60E-43	3 L1 _	Crim1
Ets11	1.01E-47	1.095669	0.869	0.577	2.03E-43	3 L1 _	Ets1
Gimap12	6.36E-48	0.688692	0.677	0.223	1.28E-43	3 L1 _	Gimap1
Lmo21	3.47E-48	0.82447	0.462	0.124	6.99E-44	3 L1 _	Lmo2
Myo18a1	3.29E-48	0.861816	0.608	0.226	6.63E-44	3 L1 _	Myo18a
Id32	2.43E-48	1.036455	0.985	0.733	4.89E-44	3 L1 _	Id3
Kcnq11	2.19E-48	0.40278	0.212	0.02	4.41E-44	3 L1 _	Kcnq1
Tshz22	1.80E-48	1.11328	0.888	0.611	3.62E-44	3 L1 _	Tshz2
Dipk2b	6.80E-49	0.30553	0.142	0.005	1.37E-44	3 L1 _	Dipk2b
Meox11	6.38E-49	0.97644	0.577	0.2	1.28E-44	3 L1 _	Meox1
Ssu21	4.47E-49	0.503227	0.223	0.023	9.00E-45	3 L1 _	Ssu2
Sox71	3.92E-49	0.452346	0.231	0.025	7.90E-45	3 L1 _	Sox7
Scarf11	3.51E-49	0.477802	0.265	0.034	7.06E-45	3 L1 _	Scarf1

Igfbp72	3.31E-49	0.874353	1	0.937	6.66E-45	3 L1 _	Igfbp7
Ahr1	2.07E-49	0.879252	0.535	0.161	4.17E-45	3 L1 _	Ahr
Nos31	1.76E-49	0.4844	0.262	0.034	3.54E-45	3 L1 _	Nos3
4931406P16Rik2	6.44E-50	1.025341	0.796	0.404	1.30E-45	3 L1 _	4931406P16Rik
Tm4sf13	5.49E-50	1.301005	0.938	0.666	1.11E-45	3 L1 _	Tm4sf1
Atox11	2.86E-50	1.097419	0.942	0.764	5.76E-46	3 L1 _	Atox1
Ndrg11	1.19E-50	1.049479	0.662	0.275	2.39E-46	3 L1 _	Ndrg1
Clu1	9.46E-51	1.491896	0.415	0.099	1.91E-46	3 L1 _	Clu
Adam151	7.21E-51	1.086909	0.7	0.316	1.45E-46	3 L1 _	Adam15
Gimap62	4.84E-51	1.071903	0.792	0.358	9.74E-47	3 L1 _	Gimap6
Hoxd3os11	4.67E-51	0.406744	0.246	0.027	9.40E-47	3 L1 _	Hoxd3os1
Chst21	4.56E-51	0.919648	0.488	0.136	9.18E-47	3 L1 _	Chst2
Pakap.11	4.42E-51	1.098788	0.804	0.395	8.91E-47	3 L1 _	Pakap.1
Ednrb2	4.05E-51	0.498754	0.777	0.259	8.15E-47	3 L1 _	Ednrb
Fzd6	2.70E-51	0.414426	0.2	0.016	5.44E-47	3 L1 _	Fzd6
Cd363	2.56E-51	1.061033	0.558	0.16	5.16E-47	3 L1 _	Cd36
Zfp9791	2.51E-51	0.52194	0.288	0.04	5.06E-47	3 L1 _	Zfp979
Eng1	1.60E-51	1.062793	0.942	0.641	3.22E-47	3 L1 _	Eng
Prkch2	3.57E-52	0.926787	0.515	0.141	7.19E-48	3 L1 _	Prkch
Fbxl71	7.09E-53	1.03916	0.585	0.195	1.43E-48	3 L1 _	Fbxl7
Nxpe41	5.75E-53	0.481837	0.277	0.035	1.16E-48	3 L1 _	Nxpe4
Notch41	3.16E-53	0.755735	0.465	0.113	6.36E-49	3 L1 _	Notch4
Pik3r31	2.11E-53	0.647957	0.342	0.057	4.26E-49	3 L1 _	Pik3r3
Lipe1	1.52E-53	0.692229	0.396	0.08	3.05E-49	3 L1 _	Lipe
Arhgap291	1.20E-53	1.269611	0.885	0.59	2.42E-49	3 L1 _	Arhgap29
Bcl6b	6.54E-54	0.441958	0.212	0.017	1.32E-49	3 L1 _	Bcl6b
Nav21	4.85E-54	0.502539	0.327	0.05	9.76E-50	3 L1 _	Nav2
Npr12	2.15E-54	0.717449	0.415	0.088	4.34E-50	3 L1 _	Npr1
Rhbdl21	8.24E-55	0.430608	0.2	0.013	1.66E-50	3 L1 _	Rhbdl2
Adcy41	5.73E-55	0.746083	0.488	0.12	1.15E-50	3 L1 _	Adcy4
Alox121	4.68E-55	0.752081	0.292	0.038	9.43E-51	3 L1 _	Alox12
Parvb1	2.35E-55	0.553791	0.327	0.048	4.73E-51	3 L1 _	Parvb
Irx31	5.23E-56	0.593882	0.292	0.037	1.05E-51	3 L1 _	Irx3
Afdn1	1.85E-56	1.104627	0.758	0.366	3.72E-52	3 L1 _	Afdn
Dok41	1.81E-56	0.68546	0.412	0.081	3.64E-52	3 L1 _	Dok4
Gpr41	1.25E-56	0.479747	0.254	0.025	2.52E-52	3 L1 _	Gpr4
Crip23	1.12E-56	1.063426	0.969	0.747	2.26E-52	3 L1 _	Crip2
Tspan122	8.09E-57	0.73807	0.5	0.115	1.63E-52	3 L1 _	Tspan12
Wscd1	1.37E-57	0.543627	0.219	0.016	2.76E-53	3 L1 _	Wscd1
Stap21	8.91E-58	0.468854	0.246	0.023	1.79E-53	3 L1 _	Stap2
Fam43a1	7.13E-58	1.001776	0.515	0.137	1.44E-53	3 L1 _	Fam43a

Rdx1	1.39E-58	1.103743	0.946	0.785	2.80E-54	3 L1 _	Rdx
Jup1	7.33E-59	1.043484	0.769	0.348	1.48E-54	3 L1 _	Jup
Nxpe21	2.00E-59	0.442013	0.231	0.018	4.03E-55	3 L1 _	Nxpe2
Elk31	1.90E-59	1.12947	0.842	0.457	3.82E-55	3 L1 _	Elk3
Snrk3	1.41E-59	1.252229	0.923	0.57	2.84E-55	3 L1 _	Snrk
Etl41	1.12E-59	0.981585	0.531	0.139	2.25E-55	3 L1 _	Etl4
Fbln22	6.42E-60	1.270676	0.896	0.502	1.29E-55	3 L1 _	Fbln2
Tmem255b1	5.76E-60	0.620846	0.308	0.039	1.16E-55	3 L1 _	Tmem255b
Hlx1	5.48E-60	0.798491	0.373	0.062	1.10E-55	3 L1 _	Hlx
Ica11	4.53E-60	0.558773	0.285	0.031	9.12E-56	3 L1 _	Ica1
Gpr1461	3.67E-60	0.709334	0.404	0.074	7.39E-56	3 L1 _	Gpr146
Ldb21	2.17E-60	1.155603	0.762	0.336	4.38E-56	3 L1 _	Ldb2
Uaca1	2.02E-60	1.155316	0.65	0.231	4.07E-56	3 L1 _	Uaca
Lama31	1.01E-60	0.62258	0.335	0.046	2.03E-56	3 L1 _	Lama3
Ptprr1	4.40E-61	0.673679	0.246	0.02	8.86E-57	3 L1 _	Ptprr
Rapgef31	3.95E-61	0.828532	0.485	0.111	7.95E-57	3 L1 _	Rapgef3
Pde2a	1.28E-61	1.089286	0.515	0.134	2.59E-57	3 L1 _	Pde2a
Epas13	8.89E-62	1.144162	0.919	0.482	1.79E-57	3 L1 _	Epas1
Tspan181	2.67E-62	0.761369	0.408	0.074	5.37E-58	3 L1 _	Tspan18
Cd381	2.18E-62	1.038687	0.612	0.186	4.39E-58	3 L1 _	Cd38
B4galt41	7.65E-63	0.782706	0.504	0.116	1.54E-58	3 L1 _	B4galt4
Sptbn11	2.82E-63	1.116647	0.992	0.89	5.67E-59	3 L1 _	Sptbn1
Kctd12b1	2.55E-63	1.216956	0.723	0.279	5.14E-59	3 L1 _	Kctd12b
Plpp32	6.71E-64	1.35453	0.958	0.703	1.35E-59	3 L1 _	Plpp3
Lrg1	4.03E-64	0.6265	0.262	0.022	8.12E-60	3 L1 _	Lrg1
Adgrl21	2.33E-64	1.140499	0.688	0.234	4.69E-60	3 L1 _	Adgrl2
Ces2e1	1.53E-64	0.680938	0.377	0.054	3.08E-60	3 L1 _	Ces2e
Ankrd33b1	1.04E-65	0.56607	0.269	0.024	2.09E-61	3 L1 _	Ankrd33b
Vamp51	5.19E-66	1.197671	0.842	0.427	1.04E-61	3 L1 _	Vamp5
Plxna21	3.38E-66	0.839455	0.508	0.109	6.81E-62	3 L1 _	Plxna2
Ccdc85b	1.79E-66	1.31702	0.835	0.458	3.61E-62	3 L1 _	Ccdc85b
Plpp11	1.25E-66	1.501949	0.908	0.563	2.52E-62	3 L1 _	Plpp1
BC0285281	4.93E-67	1.042336	0.585	0.156	9.93E-63	3 L1 _	BC028528
Rassf91	4.36E-67	0.946224	0.438	0.079	8.78E-63	3 L1 _	Rassf9
Cdh131	1.50E-67	0.645495	0.338	0.041	3.02E-63	3 L1 _	Cdh13
Sema7a1	9.80E-68	1.110086	0.642	0.189	1.97E-63	3 L1 _	Sema7a
Depp1	4.82E-68	0.60643	0.2	0.008	9.70E-64	3 L1 _	Depp1
Tnfsf102	1.06E-68	1.06503	0.654	0.177	2.14E-64	3 L1 _	Tnfsf10
Klf42	9.19E-69	1.558457	0.742	0.263	1.85E-64	3 L1 _	Klf4
Mef2c3	9.45E-70	1.388562	0.95	0.615	1.90E-65	3 L1 _	Mef2c
Aplp21	4.10E-70	1.406379	0.954	0.673	8.26E-66	3 L1 _	Aplp2

Smagp2	3.78E-70	0.87656	0.523	0.106	7.61E-66	3 L1 _	Smagp
Ccdc85a1	3.49E-70	0.643047	0.335	0.038	7.04E-66	3 L1 _	Ccdc85a
Anxa33	2.99E-70	1.070288	0.773	0.259	6.02E-66	3 L1 _	Anxa3
Mrtfb	1.32E-71	1.011474	0.562	0.137	2.65E-67	3 L1 _	Mrtfb
Trp53i11	8.10E-72	1.369964	0.623	0.175	1.63E-67	3 L1 _	Trp53i11
Cav22	5.60E-72	0.995511	0.627	0.156	1.13E-67	3 L1 _	Cav2
Ly6e1	3.41E-72	1.19529	0.992	0.865	6.87E-68	3 L1 _	Ly6e
Mecom1	1.54E-72	1.024528	0.362	0.045	3.10E-68	3 L1 _	Mecom
Tspan132	1.37E-72	1.098766	0.588	0.143	2.76E-68	3 L1 _	Tspan13
Plscr21	5.88E-73	1.327931	0.808	0.33	1.18E-68	3 L1 _	Plscr2
Ccm2l1	5.88E-73	0.758549	0.358	0.042	1.18E-68	3 L1 _	Ccm2l
Kif26a1	4.48E-73	0.590577	0.308	0.028	9.01E-69	3 L1 _	Kif26a
Chst151	1.87E-73	0.928704	0.512	0.1	3.77E-69	3 L1 _	Chst15
Gnai21	1.54E-73	1.041292	0.973	0.919	3.09E-69	3 L1 _	Gnai2
Mall1	9.04E-74	0.617859	0.296	0.025	1.82E-69	3 L1 _	Mall
Ripply31	3.35E-74	0.783874	0.458	0.077	6.74E-70	3 L1 _	Ripply3
Calm12	2.81E-74	1.094692	0.992	0.947	5.66E-70	3 L1 _	Calm1
Rasgrf21	6.98E-75	0.593406	0.323	0.031	1.41E-70	3 L1 _	Rasgrf2
Lmcd11	5.58E-75	1.208931	0.577	0.138	1.12E-70	3 L1 _	Lmcd1
Syne11	2.53E-75	1.448675	0.762	0.289	5.09E-71	3 L1 _	Syne1
Klf21	5.14E-76	1.811151	0.877	0.416	1.03E-71	3 L1 _	Klf2
Pcp4l13	3.09E-76	1.121929	0.688	0.169	6.23E-72	3 L1 _	Pcp4l1
Bcam3	2.44E-76	1.266926	0.815	0.271	4.91E-72	3 L1 _	Bcam
C430049B03Rik	2.35E-76	0.468828	0.262	0.015	4.72E-72	3 L1 _	C430049B03Rik
Srgn3	1.54E-76	1.348922	0.915	0.359	3.09E-72	3 L1 _	Srgn
Nfib2	6.83E-77	1.303151	0.981	0.734	1.38E-72	3 L1 _	Nfib
Cav13	1.66E-77	1.247946	0.804	0.25	3.35E-73	3 L1 _	Cav1
Pdgfb1	1.51E-77	1.130293	0.658	0.164	3.04E-73	3 L1 _	Pdgfb
Limch11	1.12E-79	0.86305	0.392	0.048	2.26E-75	3 L1 _	Limch1
Unc45b1	4.88E-80	0.822768	0.469	0.074	9.83E-76	3 L1 _	Unc45b
Mfsd61	4.79E-80	0.841028	0.465	0.073	9.65E-76	3 L1 _	Mfsd6
Sema3f1	3.62E-80	0.848527	0.431	0.06	7.30E-76	3 L1 _	Sema3f
Jag21	1.94E-80	0.827085	0.388	0.046	3.91E-76	3 L1 _	Jag2
Nostrin1	1.83E-80	1.173584	0.623	0.139	3.69E-76	3 L1 _	Nostrin
Flt41	2.01E-81	0.745915	0.381	0.042	4.06E-77	3 L1 _	Flt4
Zfp3661	5.04E-82	0.675119	0.35	0.033	1.01E-77	3 L1 _	Zfp366
Rsad21	4.61E-82	1.391144	0.677	0.166	9.29E-78	3 L1 _	Rsad2
Pltp1	1.85E-82	1.406833	0.792	0.268	3.72E-78	3 L1 _	Pltp
Sema6a1	1.74E-82	0.946673	0.569	0.117	3.50E-78	3 L1 _	Sema6a
Ece11	1.88E-83	1.39463	0.819	0.312	3.79E-79	3 L1 _	Ece1
Afap1l11	2.45E-84	0.672995	0.404	0.046	4.94E-80	3 L1 _	Afap1l1

Fli11	1.39E-84	1.236053	0.692	0.187	2.79E-80	3 L1 _	Fli1
Dysf1	1.09E-85	0.942097	0.535	0.091	2.19E-81	3 L1 _	Dysf
Ehd41	4.97E-86	1.427332	0.823	0.294	1.00E-81	3 L1 _	Ehd4
Aplnr1	3.85E-86	1.090759	0.373	0.037	7.76E-82	3 L1 _	Aplnr
Nova21	1.74E-86	0.680637	0.369	0.035	3.51E-82	3 L1 _	Nova2
Lims21	4.15E-87	1.122498	0.565	0.105	8.35E-83	3 L1 _	Lims2
Tcim1	4.61E-88	1.545132	0.762	0.224	9.29E-84	3 L1 _	Tcim
Gm120021	1.41E-88	0.799027	0.362	0.031	2.83E-84	3 L1 _	Gm12002
Ly6a1	8.02E-89	1.856265	0.996	0.7	1.62E-84	3 L1 _	Ly6a
Samd121	2.23E-91	0.719643	0.377	0.034	4.50E-87	3 L1 _	Samd12
Clec14a1	2.44E-92	1.639557	0.865	0.324	4.92E-88	3 L1 _	Clec14a
Plekho11	2.18E-93	1.13055	0.608	0.116	4.39E-89	3 L1 _	Plekho1
Mcf2l1	1.27E-93	1.085698	0.531	0.08	2.55E-89	3 L1 _	Mcf2l
Id11	9.14E-94	1.533782	0.792	0.224	1.84E-89	3 L1 _	Id1
Gata21	1.88E-94	0.73672	0.388	0.034	3.80E-90	3 L1 _	Gata2
Ctla2a2	1.20E-94	1.910712	0.869	0.293	2.42E-90	3 L1 _	Ctla2a
Car41	1.53E-95	1.066499	0.442	0.048	3.08E-91	3 L1 _	Car4
Exoc3l21	5.96E-96	1.005729	0.508	0.068	1.20E-91	3 L1 _	Exoc3l2
Smco41	3.34E-96	1.272001	0.758	0.204	6.73E-92	3 L1 _	Smco4
Cracr2b1	1.29E-96	1.068712	0.542	0.087	2.60E-92	3 L1 _	Cracr2b
Tcf151	1.43E-97	1.189558	0.527	0.074	2.87E-93	3 L1 _	Tcf15
Dll4	8.97E-99	0.764431	0.331	0.019	1.81E-94	3 L1 _	Dll4
Ets21	2.44E-99	1.519684	0.738	0.201	4.91E-95	3 L1 _	Ets2
Erg1	2.38E-99	1.064415	0.508	0.072	4.80E-95	3 L1 _	Erg
Cd343	7.71E-100	1.725247	0.938	0.388	1.55E-95	3 L1 _	Cd34
Adgrg11	5.46E-101	0.838689	0.512	0.064	1.10E-96	3 L1 _	Adgrg1
Plk21	2.23E-101	1.230307	0.558	0.087	4.50E-97	3 L1 _	Plk2
Efnb21	1.57E-101	1.181683	0.55	0.083	3.16E-97	3 L1 _	Efnb2
Slc9a3r21	6.15E-103	1.847641	0.931	0.415	1.24E-98	3 L1 _	Slc9a3r2
Clec1a1	5.05E-103	1.280176	0.631	0.117	1.02E-98	3 L1 _	Clec1a
Fgd51	2.85E-103	1.178187	0.658	0.132	5.74E-99	3 L1 _	Fgd5
Arap31	1.62E-103	1.148422	0.658	0.129	3.26E-99	3 L1 _	Arap3
Ramp22	1.10E-104	1.859265	0.95	0.442	2.23E-100	3 L1 _	Ramp2
Jam21	3.80E-107	1.784196	0.838	0.275	7.65E-103	3 L1 _	Jam2
She1	1.64E-107	1.046552	0.546	0.074	3.30E-103	3 L1 _	She
Rapgef41	9.26E-108	0.922837	0.488	0.054	1.86E-103	3 L1 _	Rapgef4
Robo41	3.67E-110	1.019484	0.492	0.053	7.39E-106	3 L1 _	Robo4
Esam3	7.49E-111	1.698911	0.908	0.265	1.51E-106	3 L1 _	Esam
Upp11	4.06E-111	0.912644	0.427	0.034	8.18E-107	3 L1 _	Upp1
Cd24a1	1.89E-112	1.085693	0.604	0.085	3.80E-108	3 L1 _	Cd24a
Sox181	4.45E-114	0.978804	0.462	0.042	8.96E-110	3 L1 _	Sox18

Dock41	7.81E-115	1.054494	0.558	0.073	1.57E-110	3 L1 _	Dock4
Sorbs22	6.66E-116	1.417044	0.712	0.129	1.34E-111	3 L1 _	Sorbs2
Entpd11	4.36E-116	1.372157	0.715	0.141	8.78E-112	3 L1 _	Entpd1
Sox171	2.87E-116	1.359258	0.485	0.046	5.79E-112	3 L1 _	Sox17
Col13a11	2.31E-116	1.495998	0.677	0.118	4.66E-112	3 L1 _	Col13a1
Arhgef151	2.27E-117	1.034834	0.573	0.071	4.56E-113	3 L1 _	Arhgef15
Arhgap311	2.17E-122	1.883795	0.9	0.295	4.37E-118	3 L1 _	Arhgap31
Fabp41	1.56E-122	2.227095	0.973	0.39	3.15E-118	3 L1 _	Fabp4
Plvap1	6.06E-123	2.646672	0.973	0.471	1.22E-118	3 L1 _	Plvap
Rflnb1	9.29E-126	1.97734	0.885	0.252	1.87E-121	3 L1 _	Rflnb
Rgcc1	3.07E-127	1.746869	0.842	0.197	6.18E-123	3 L1 _	Rgcc
Kank31	7.81E-129	1.225005	0.665	0.098	1.57E-124	3 L1 _	Kank3
Ecscr1	1.61E-131	1.650773	0.785	0.166	3.23E-127	3 L1 _	Ecscr
C130074G19Rik1	2.36E-132	1.141514	0.55	0.053	4.74E-128	3 L1 _	C130074G19Rik
Cavin21	5.91E-133	1.904657	0.9	0.214	1.19E-128	3 L1 _	Cavin2
S1pr11	3.26E-133	1.582394	0.738	0.129	6.57E-129	3 L1 _	S1pr1
Plcb11	3.26E-134	1.436807	0.673	0.092	6.57E-130	3 L1 _	Plcb1
Ceacam11	6.24E-135	1.162365	0.554	0.052	1.26E-130	3 L1 _	Ceacam1
Ramp31	1.18E-138	1.699742	0.669	0.089	2.37E-134	3 L1 _	Ramp3
Rbp71	1.19E-141	2.220824	0.8	0.149	2.40E-137	3 L1 _	Rbp7
Cmtm81	1.59E-142	1.285629	0.619	0.065	3.20E-138	3 L1 _	Cmtm8
Palmd1	2.27E-147	1.263781	0.6	0.057	4.57E-143	3 L1 _	Palmd
Fam167b1	2.05E-153	1.469743	0.65	0.067	4.13E-149	3 L1 _	Fam167b
Efna11	4.20E-154	1.469633	0.635	0.067	8.46E-150	3 L1 _	Efna1
Ly6c11	3.42E-154	2.937014	1	0.355	6.88E-150	3 L1 _	Ly6c1
Tmem881	2.13E-154	1.284048	0.619	0.057	4.29E-150	3 L1 _	Tmem88
Btnl91	1.57E-154	1.67197	0.712	0.082	3.16E-150	3 L1 _	Btnl9
Rapgef51	2.35E-155	1.595312	0.677	0.078	4.74E-151	3 L1 _	Rapgef5
Cd2001	2.15E-155	2.13356	0.915	0.194	4.33E-151	3 L1 _	Cd200
Cd300lg1	1.81E-156	1.796513	0.769	0.11	3.64E-152	3 L1 _	Cd300lg
Itga61	3.38E-158	1.538434	0.773	0.106	6.81E-154	3 L1 _	Itga6
Aqp1	2.89E-159	2.173589	0.727	0.101	5.81E-155	3 L1 _	Aqp1
Adgrf51	2.61E-159	1.895289	0.881	0.16	5.26E-155	3 L1 _	Adgrf5
Tek2	5.22E-160	1.485466	0.738	0.094	1.05E-155	3 L1 _	Tek
Rasgrp31	4.20E-160	1.408151	0.688	0.074	8.46E-156	3 L1 _	Rasgrp3
Grrp11	8.91E-161	1.470478	0.654	0.066	1.79E-156	3 L1 _	Grrp1
Igfbp31	2.72E-161	3.235897	0.888	0.196	5.47E-157	3 L1 _	Igfbp3
Thrsp1	2.66E-161	1.561309	0.762	0.095	5.37E-157	3 L1 _	Thrsp
Mgll1	1.23E-161	2.030258	0.923	0.199	2.47E-157	3 L1 _	Mgll
Ushbp11	1.34E-162	1.606179	0.735	0.092	2.69E-158	3 L1 _	Ushbp1
Egfl71	1.12E-165	2.901588	0.996	0.318	2.26E-161	3 L1 _	Egfl7

Tie11	4.66E-169	1.643916	0.815	0.111	9.39E-165	3 L1 _	Tie1
Emcn1	2.92E-172	2.431317	0.946	0.197	5.89E-168	3 L1 _	Emcn
Icam21	4.11E-173	1.60089	0.75	0.09	8.28E-169	3 L1 _	Icam2
Cd931	1.46E-175	1.960879	0.808	0.108	2.93E-171	3 L1 _	Cd93
Rasip11	6.90E-180	1.525299	0.742	0.078	1.39E-175	3 L1 _	Rasip1
Pcdh171	1.28E-185	1.756564	0.735	0.07	2.58E-181	3 L1 _	Pcdh17
Gpihbp11	8.10E-186	2.8208	0.881	0.144	1.63E-181	3 L1 _	Gpihbp1
Flt11	6.20E-188	2.770948	0.985	0.206	1.25E-183	3 L1 _	Flt1
Kdr1	4.06E-188	2.237426	0.931	0.152	8.19E-184	3 L1 _	Kdr
Scarb11	1.66E-191	2.217317	0.819	0.105	3.35E-187	3 L1 _	Scarb1
Myct11	4.48E-192	1.609764	0.731	0.067	9.03E-188	3 L1 _	Myct1
Ptprb1	1.01E-192	2.73324	0.981	0.198	2.03E-188	3 L1 _	Ptprb
Podxl1	1.69E-195	1.977278	0.904	0.121	3.40E-191	3 L1 _	Podxl
Pecam11	1.00E-197	2.713041	0.988	0.194	2.02E-193	3 L1 _	Pecam1
Clic51	1.12E-198	1.87346	0.8	0.079	2.25E-194	3 L1 _	Clic5
Adgrl41	3.78E-202	2.274273	0.9	0.121	7.60E-198	3 L1 _	Adgrl4
Mmrn21	3.54E-205	2.024343	0.831	0.092	7.12E-201	3 L1 _	Mmrn2
Cyyr11	5.57E-220	2.101367	0.869	0.087	1.12E-215	3 L1 _	Cyyr1
Cdh51	3.50E-231	2.369626	0.938	0.109	7.05E-227	3 L1 _	Cdh5
Rab7	2.49E-06	0.349418	0.829	0.642	0.050181	3 M1 _	Rab7
Tax1bp3	2.44E-06	0.391532	0.634	0.404	0.049081	3 M1 _	Tax1bp3
Usp25	2.36E-06	0.417775	0.598	0.379	0.047598	3 M1 _	Usp25
Mmd	2.21E-06	0.277472	0.244	0.091	0.044495	3 M1 _	Mmd
Lin7c	2.16E-06	0.28423	0.439	0.22	0.043449	3 M1 _	Lin7c
Socs2	2.14E-06	0.373606	0.744	0.488	0.043177	3 M1 _	Socs2
Capza2	2.11E-06	0.427399	0.854	0.689	0.042458	3 M1 _	Capza2
Myl61	2.08E-06	0.331547	1	0.966	0.041825	3 M1 _	Myl6
Trak2	2.03E-06	0.353681	0.366	0.167	0.040923	3 M1 _	Trak2
Otud4	2.01E-06	0.456293	0.427	0.22	0.040484	3 M1 _	Otud4
Metap2	1.84E-06	0.389509	0.854	0.692	0.037051	3 M1 _	Metap2
Erf	1.78E-06	0.279129	0.305	0.126	0.035877	3 M1 _	Erf
Ass1	1.78E-06	0.312907	0.329	0.143	0.035778	3 M1 _	Ass1
Fam126a	1.77E-06	0.349877	0.354	0.159	0.035731	3 M1 _	Fam126a
Map21	1.75E-06	0.34877	0.341	0.149	0.035151	3 M1 _	Map2
Ep400	1.74E-06	0.367279	0.573	0.343	0.035093	3 M1 _	Ep400
Emc2	1.73E-06	0.426884	0.61	0.386	0.034804	3 M1 _	Emc2
Kbtbd2	1.69E-06	0.347162	0.378	0.179	0.033999	3 M1 _	Kbtbd2
Plcb41	1.63E-06	0.371264	0.488	0.25	0.032825	3 M1 _	Plcb4
Mindy1	1.57E-06	0.39678	0.402	0.197	0.031685	3 M1 _	Mindy1
Kmt2d	1.53E-06	0.380298	0.402	0.194	0.030791	3 M1 _	Kmt2d
Ubc	1.46E-06	0.33134	0.988	0.928	0.029439	3 M1 _	Ubc

Iigp1	1.42E-06	0.561256	0.634	0.417	0.028523	3 M1 _	Iigp1
Atxn3	1.36E-06	0.267687	0.305	0.126	0.027374	3 M1 _	Atxn3
Ddx54	1.34E-06	0.373565	0.5	0.274	0.027005	3 M1 _	Ddx54
Fam220a2	1.31E-06	0.298119	0.183	0.055	0.026477	3 M1 _	Fam220a
Nexn	1.24E-06	0.384936	0.366	0.167	0.024979	3 M1 _	Nexn
Arhgap5	1.22E-06	0.500464	0.78	0.589	0.024562	3 M1 _	Arhgap5
AU0202061	1.21E-06	0.27354	0.451	0.219	0.024289	3 M1 _	AU020206
G3bp1	1.19E-06	0.54656	0.72	0.491	0.024003	3 M1 _	G3bp1
Ywhae	1.19E-06	0.450865	0.878	0.782	0.023964	3 M1 _	Ywhae
Cpne8	1.13E-06	0.383866	0.561	0.33	0.022782	3 M1 _	Cpne8
Tsnax	1.12E-06	0.346535	0.512	0.278	0.022613	3 M1 _	Tsnax
Fam210b	1.10E-06	0.284203	0.28	0.106	0.022158	3 M1 _	Fam210b
Hnrnpf	1.08E-06	0.36244	0.878	0.727	0.021768	3 M1 _	Hnrnpf
Stox2	1.05E-06	0.448697	0.415	0.21	0.021095	3 M1 _	Stox2
Wdfy1	1.01E-06	0.315493	0.329	0.14	0.020266	3 M1 _	Wdfy1
Tcn2	9.77E-07	0.561866	0.695	0.515	0.019668	3 M1 _	Tcn2
Pdcl3	9.58E-07	0.49924	0.622	0.401	0.019292	3 M1 _	Pdcl3
Hdac7	8.91E-07	0.488597	0.671	0.444	0.017947	3 M1 _	Hdac7
Mef2a1	8.34E-07	0.414476	0.866	0.654	0.016789	3 M1 _	Mef2a
Nomo1	8.09E-07	0.336475	0.341	0.148	0.016295	3 M1 _	Nomo1
Cyth1	7.91E-07	0.262712	0.268	0.101	0.015934	3 M1 _	Cyth1
Wbp4	7.84E-07	0.515952	0.659	0.467	0.015799	3 M1 _	Wbp4
Nras	7.39E-07	0.414743	0.573	0.338	0.01488	3 M1 _	Nras
Mef2d	7.17E-07	0.422202	0.451	0.236	0.01445	3 M1 _	Mef2d
Zmynd11	7.07E-07	0.463126	0.756	0.545	0.01424	3 M1 _	Zmynd11
Fkbp1a	6.75E-07	0.444231	0.854	0.784	0.013594	3 M1 _	Fkbp1a
Nhsl2	6.73E-07	0.367487	0.415	0.198	0.013549	3 M1 _	Nhsl2
Hras	6.70E-07	0.492525	0.634	0.435	0.013502	3 M1 _	Hras
Smurf2	6.64E-07	0.401843	0.549	0.307	0.013364	3 M1 _	Smurf2
Mndal	6.56E-07	0.477909	0.866	0.661	0.013222	3 M1 _	Mndal
Zfp462	6.56E-07	0.499497	0.354	0.158	0.013211	3 M1 _	Zfp462
Afap1	6.51E-07	0.467301	0.61	0.389	0.013117	3 M1 _	Afap1
Peak1	6.45E-07	0.42669	0.805	0.625	0.012991	3 M1 _	Peak1
Eno1	6.28E-07	0.536225	0.585	0.377	0.012651	3 M1 _	Eno1
Wdr37	6.21E-07	0.351382	0.329	0.141	0.012497	3 M1 _	Wdr37
Baz2b	6.04E-07	0.532164	0.61	0.394	0.012167	3 M1 _	Baz2b
Bmi1	5.73E-07	0.320622	0.341	0.151	0.011535	3 M1 _	Bmi1
B2m	5.67E-07	0.409489	1	0.949	0.011424	3 M1 _	B2m
Ctbp1	5.65E-07	0.457936	0.732	0.518	0.011376	3 M1 _	Ctbp1
Sin3a	5.44E-07	0.350979	0.402	0.189	0.010962	3 M1 _	Sin3a
Taok11	5.23E-07	0.424462	0.707	0.516	0.010528	3 M1 _	Taok1

Tmtc4	5.08E-07	0.258895	0.171	0.047	0.010241	3 M1 _	Tmtc4
Hif1a	5.08E-07	0.533788	0.646	0.422	0.010225	3 M1 _	Hif1a
Apc	4.90E-07	0.441002	0.573	0.347	0.009872	3 M1 _	Apc
Tns11	4.61E-07	0.527914	0.744	0.527	0.009276	3 M1 _	Tns1
Tmpo	4.57E-07	0.396788	0.61	0.35	0.0092	3 M1 _	Tmpo
Aplnr	4.50E-07	0.289909	0.22	0.069	0.009067	3 M1 _	Aplnr
Nqo2	4.50E-07	0.315046	0.329	0.134	0.00906	3 M1 _	Nqo2
Cnn21	4.33E-07	0.467813	0.695	0.436	0.008717	3 M1 _	Cnn2
Cic	4.27E-07	0.339699	0.476	0.245	0.008591	3 M1 _	Cic
Shoc2	4.19E-07	0.509617	0.61	0.365	0.008441	3 M1 _	Shoc2
Hbb-bs1	4.02E-07	3.356176	0.451	0.221	0.008099	3 M1 _	Hbb-bs
Myl12b	3.92E-07	0.430483	0.902	0.792	0.007886	3 M1 _	Myl12b
Parp14	3.79E-07	0.405253	0.634	0.383	0.007624	3 M1 _	Parp14
Dab2	3.77E-07	0.536077	0.646	0.418	0.0076	3 M1 _	Dab2
Palm	3.66E-07	0.492339	0.463	0.244	0.007374	3 M1 _	Palm
Swap70	3.64E-07	0.484159	0.598	0.376	0.007339	3 M1 _	Swap70
Kifc3	3.64E-07	0.314083	0.305	0.116	0.007333	3 M1 _	Kifc3
Slco2b1	3.51E-07	0.53998	0.378	0.174	0.007074	3 M1 _	Slco2b1
Sh3bgrl1	3.32E-07	0.521376	0.817	0.682	0.006677	3 M1 _	Sh3bgrl
Selenot1	3.18E-07	0.483157	0.622	0.384	0.006395	3 M1 _	Selenot
Tollip	3.00E-07	0.285433	0.329	0.131	0.006048	3 M1 _	Tollip
Cnot6	2.86E-07	0.376321	0.61	0.36	0.005758	3 M1 _	Cnot6
Golm1	2.79E-07	0.472157	0.415	0.197	0.005629	3 M1 _	Golm1
Gtf2i	2.76E-07	0.485971	0.659	0.422	0.005551	3 M1 _	Gtf2i
Gapdh1	2.61E-07	0.395936	0.951	0.906	0.005262	3 M1 _	Gapdh
Trappc6b	2.50E-07	0.45896	0.707	0.499	0.005043	3 M1 _	Trappc6b
Nmi	2.39E-07	0.545241	0.561	0.309	0.004814	3 M1 _	Nmi
Inpp5a	2.36E-07	0.389926	0.451	0.221	0.004754	3 M1 _	Inpp5a
Il6st1	2.32E-07	0.552152	0.78	0.572	0.004665	3 M1 _	Il6st
Snrnp200	2.31E-07	0.447015	0.561	0.319	0.004651	3 M1 _	Snrnp200
Ppp1r15a	2.25E-07	0.34542	0.549	0.279	0.004537	3 M1 _	Ppp1r15a
Rgl2	2.16E-07	0.444585	0.378	0.173	0.004357	3 M1 _	Rgl2
Rab12	2.13E-07	0.466067	0.732	0.503	0.00429	3 M1 _	Rab12
Tm6sf1	2.12E-07	0.363333	0.305	0.116	0.004275	3 M1 _	Tm6sf1
2700081O15Rik	2.12E-07	0.37183	0.524	0.276	0.004271	3 M1 _	2700081O15Rik
Prdx4	2.12E-07	0.48574	0.732	0.516	0.00427	3 M1 _	Prdx4
Stim2	2.04E-07	0.478843	0.488	0.251	0.004103	3 M1 _	Stim2
Lrp5	1.85E-07	0.31869	0.341	0.138	0.003732	3 M1 _	Lrp5
Kat6a	1.84E-07	0.485205	0.634	0.4	0.003712	3 M1 _	Kat6a
Lifr1	1.82E-07	0.642673	0.634	0.403	0.003666	3 M1 _	Lifr
Fbxo7	1.80E-07	0.258246	0.329	0.129	0.003631	3 M1 _	Fbxo7

Sptlc2	1.75E-07	0.48284	0.549	0.303	0.003518	3 M1 _	Sptlc2
Nmt11	1.69E-07	0.444513	0.902	0.655	0.003405	3 M1 _	Nmt1
Slk	1.58E-07	0.543441	0.683	0.472	0.00319	3 M1 _	Slk
Scoc1	1.55E-07	0.385008	0.61	0.341	0.003122	3 M1 _	Scoc
Ptpn12	1.53E-07	0.4591	0.573	0.326	0.003091	3 M1 _	Ptpn12
Mapre1	1.52E-07	0.52158	0.756	0.583	0.00307	3 M1 _	Mapre1
Jade2	1.51E-07	0.389405	0.366	0.153	0.003045	3 M1 _	Jade2
Actb1	1.50E-07	0.330553	1	0.992	0.003018	3 M1 _	Actb
H2-T23	1.48E-07	0.486151	0.78	0.527	0.002991	3 M1 _	H2-T23
Prkce	1.48E-07	0.371123	0.293	0.11	0.002983	3 M1 _	Prkce
Elovl5	1.41E-07	0.493158	0.463	0.233	0.002845	3 M1 _	Elovl5
Nploc4	1.36E-07	0.329136	0.305	0.118	0.00273	3 M1 _	Nploc4
Atxn1	1.32E-07	0.494572	0.451	0.231	0.002666	3 M1 _	Atxn1
Tapbp	1.30E-07	0.643543	0.817	0.634	0.00261	3 M1 _	Tapbp
Kctd10	1.28E-07	0.509975	0.573	0.316	0.002587	3 M1 _	Kctd10
Aldh22	1.27E-07	0.570659	0.915	0.753	0.002554	3 M1 _	Aldh2
Arhgdia	1.26E-07	0.449307	0.902	0.764	0.002532	3 M1 _	Arhgdia
Asap1	1.25E-07	0.554294	0.841	0.61	0.002519	3 M1 _	Asap1
Crk	1.21E-07	0.62061	0.585	0.366	0.00244	3 M1 _	Crk
Mtor	1.20E-07	0.314557	0.317	0.123	0.002421	3 M1 _	Mtor
Xylt2	1.16E-07	0.324665	0.329	0.13	0.002342	3 M1 _	Xylt2
Pdcl	1.14E-07	0.371521	0.451	0.213	0.002305	3 M1 _	Pdcl
Klc1	1.11E-07	0.508322	0.622	0.359	0.002245	3 M1 _	Klc1
Chd1	1.09E-07	0.510185	0.549	0.308	0.002201	3 M1 _	Chd1
Psmb9	1.07E-07	0.551177	0.634	0.374	0.002152	3 M1 _	Psmb9
Gosr2	1.01E-07	0.416175	0.439	0.209	0.002041	3 M1 _	Gosr2
Map3k1	9.45E-08	0.337358	0.366	0.153	0.001902	3 M1 _	Map3k1
Nrp2	8.81E-08	0.506683	0.622	0.339	0.001775	3 M1 _	Nrp2
Hnrnpa1	8.69E-08	0.502695	0.756	0.54	0.00175	3 M1 _	Hnrnpa1
Hipk11	8.35E-08	0.462284	0.61	0.367	0.001682	3 M1 _	Hipk1
Herc2	8.12E-08	0.603308	0.646	0.437	0.001635	3 M1 _	Herc2
Suz12	7.71E-08	0.411941	0.5	0.251	0.001552	3 M1 _	Suz12
Top1	7.38E-08	0.550426	0.854	0.703	0.001485	3 M1 _	Top1
Eogt	7.31E-08	0.276682	0.232	0.072	0.001473	3 M1 _	Eogt
Rbm8a1	7.27E-08	0.483719	0.817	0.574	0.001464	3 M1 _	Rbm8a
Ddx21	6.67E-08	0.623422	0.695	0.445	0.001343	3 M1 _	Ddx21
Tmod3	6.47E-08	0.559866	0.78	0.606	0.001303	3 M1 _	Tmod3
Elmo1	6.24E-08	0.260763	0.366	0.144	0.001257	3 M1 _	Elmo1
Sertad2	5.94E-08	0.377305	0.561	0.287	0.001196	3 M1 _	Sertad2
Jun	5.54E-08	0.914576	0.915	0.689	0.001116	3 M1 _	Jun
Ywhaz1	5.33E-08	0.459694	0.902	0.806	0.001073	3 M1 _	Ywhaz

Psmc1	5.30E-08	0.524611	0.756	0.503	0.001068	3 M1 _	Psmc1
Vamp3	4.77E-08	0.440685	0.573	0.314	0.00096	3 M1 _	Vamp3
Ivns1abp	4.43E-08	0.571158	0.561	0.313	0.000891	3 M1 _	Ivns1abp
Coro2b	4.40E-08	0.275936	0.244	0.077	0.000885	3 M1 _	Coro2b
Itga5	4.27E-08	0.35577	0.415	0.18	0.00086	3 M1 _	Itga5
Pfkfb3	4.25E-08	0.300511	0.183	0.047	0.000856	3 M1 _	Pfkfb3
Smim10l1	3.84E-08	0.536003	0.768	0.537	0.000773	3 M1 _	Smim10l1
Ifi35	3.79E-08	0.51731	0.659	0.393	0.000764	3 M1 _	Ifi35
Rab5c	3.55E-08	0.407642	0.805	0.578	0.000715	3 M1 _	Rab5c
Rapgef1	3.32E-08	0.481086	0.451	0.21	0.000668	3 M1 _	Rapgef1
Nod1	3.32E-08	0.363726	0.366	0.145	0.000668	3 M1 _	Nod1
Abca3	3.28E-08	0.345459	0.317	0.117	0.00066	3 M1 _	Abca3
Gimap5	3.21E-08	0.422617	0.476	0.206	0.000646	3 M1 _	Gimap5
Egln1	3.06E-08	0.49034	0.659	0.418	0.000616	3 M1 _	Egln1
Ucp21	2.97E-08	0.49671	0.622	0.327	0.000598	3 M1 _	Ucp2
Gpd2	2.87E-08	0.35252	0.317	0.119	0.000577	3 M1 _	Gpd2
Rb1	2.85E-08	0.530499	0.451	0.205	0.000574	3 M1 _	Rb1
Pogk	2.61E-08	0.467434	0.366	0.149	0.000525	3 M1 _	Pogk
Tmem30a	2.50E-08	0.506115	0.793	0.604	0.000504	3 M1 _	Tmem30a
Tlr3	2.44E-08	0.515411	0.378	0.156	0.000491	3 M1 _	Tlr3
Baiap2	2.33E-08	0.310632	0.366	0.145	0.00047	3 M1 _	Baiap2
Spop	2.32E-08	0.548386	0.744	0.549	0.000467	3 M1 _	Spop
Chmp5	2.30E-08	0.433602	0.817	0.543	0.000463	3 M1 _	Chmp5
Rnf19a	2.26E-08	0.452589	0.439	0.204	0.000455	3 M1 _	Rnf19a
Aig11	2.16E-08	0.331201	0.488	0.22	0.000435	3 M1 _	Aig1
Mxd4	2.16E-08	0.532879	0.866	0.772	0.000434	3 M1 _	Mxd4
Irf1	2.03E-08	0.643285	0.598	0.349	0.000409	3 M1 _	Irf1
Cdc42ep41	2.01E-08	0.464042	0.524	0.264	0.000405	3 M1 _	Cdc42ep4
Snx3	2.00E-08	0.481138	0.878	0.688	0.000403	3 M1 _	Snx3
Rras1	2.00E-08	0.542875	0.841	0.673	0.000402	3 M1 _	Rras
Hey1	1.97E-08	0.442644	0.171	0.041	0.000397	3 M1 _	Hey1
Adarb1	1.94E-08	0.450674	0.402	0.169	0.000392	3 M1 _	Adarb1
Bend7	1.79E-08	0.30105	0.171	0.041	0.00036	3 M1 _	Bend7
Smtn1	1.76E-08	0.485775	0.561	0.303	0.000354	3 M1 _	Smtn
Dock4	1.76E-08	0.385939	0.329	0.119	0.000353	3 M1 _	Dock4
B230219D22Rik1	1.74E-08	0.51611	0.866	0.704	0.000351	3 M1 _	B230219D22Rik
Agrn	1.74E-08	0.39384	0.439	0.198	0.000351	3 M1 _	Agrn
Mink1	1.62E-08	0.382856	0.378	0.154	0.000326	3 M1 _	Mink1
Hprt	1.54E-08	0.446983	0.585	0.324	0.00031	3 M1 _	Hprt
Timeless	1.28E-08	0.380465	0.305	0.107	0.000258	3 M1 _	Timeless
Phldb2	1.08E-08	0.47617	0.573	0.295	0.000218	3 M1 _	Phldb2

Trim65	1.06E-08	0.295803	0.256	0.078	0.000213	3 M1 _	Trim65
Bst2	1.04E-08	0.578352	0.939	0.708	0.000209	3 M1 _	Bst2
Ddx3x	1.04E-08	0.435451	0.854	0.661	0.000209	3 M1 _	Ddx3x
Txn11	9.88E-09	0.502622	0.939	0.83	0.000199	3 M1 _	Txn1
Ace1	9.39E-09	0.644025	0.646	0.39	0.000189	3 M1 _	Ace
Zhx31	9.28E-09	0.53838	0.585	0.322	0.000187	3 M1 _	Zhx3
Pfkl	9.22E-09	0.418075	0.378	0.149	0.000186	3 M1 _	Pfkl
Rnf44	9.02E-09	0.341574	0.402	0.165	0.000182	3 M1 _	Rnf44
Tmem63b	8.63E-09	0.343241	0.354	0.13	0.000174	3 M1 _	Tmem63b
Csgalnact1	8.52E-09	0.452359	0.415	0.175	0.000172	3 M1 _	Csgalnact1
Rin31	8.50E-09	0.259452	0.366	0.134	0.000171	3 M1 _	Rin3
Prrg2	7.49E-09	0.355191	0.305	0.104	0.000151	3 M1 _	Prrg2
Hhex	7.38E-09	0.321276	0.256	0.077	0.000149	3 M1 _	Hhex
Grap	7.08E-09	0.256773	0.378	0.14	0.000143	3 M1 _	Grap
Card10	6.87E-09	0.356757	0.341	0.124	0.000138	3 M1 _	Card10
Fbxl7	6.85E-09	0.319886	0.512	0.228	0.000138	3 M1 _	Fbxl7
Pak2	6.72E-09	0.544644	0.841	0.654	0.000135	3 M1 _	Pak2
Plekhg5	6.58E-09	0.357396	0.305	0.105	0.000132	3 M1 _	Plekhg5
Znfx1	6.42E-09	0.484301	0.512	0.252	0.000129	3 M1 _	Znfx1
Prkd2	6.24E-09	0.348432	0.354	0.134	0.000126	3 M1 _	Prkd2
Dusp6	6.19E-09	0.683074	0.561	0.309	0.000125	3 M1 _	Dusp6
Setx	5.99E-09	0.501559	0.476	0.223	0.000121	3 M1 _	Setx
Atp11c	5.93E-09	0.356887	0.378	0.146	0.00012	3 M1 _	Atp11c
Smarca4	5.48E-09	0.646442	0.512	0.265	0.00011	3 M1 _	Smarca4
Cab39	5.38E-09	0.521483	0.573	0.315	0.000108	3 M1 _	Cab39
Gng111	5.33E-09	0.447123	0.939	0.816	0.000107	3 M1 _	Gng11
Ttc7b	5.24E-09	0.38612	0.244	0.072	0.000105	3 M1 _	Ttc7b
Myh91	5.07E-09	0.652404	0.915	0.826	0.000102	3 M1 _	Myh9
Wasf2	5.02E-09	0.592651	0.817	0.724	0.000101	3 M1 _	Wasf2
Pcnx	4.99E-09	0.403914	0.512	0.243	0.0001	3 M1 _	Pcnx
Gna11	4.58E-09	0.625773	0.78	0.61	9.23E-05	3 M1 _	Gna11
Ybx1	4.46E-09	0.449504	0.963	0.906	8.99E-05	3 M1 _	Ybx1
Fes	4.46E-09	0.388723	0.329	0.116	8.98E-05	3 M1 _	Fes
Ppp1r21	4.43E-09	0.576475	0.878	0.681	8.92E-05	3 M1 _	Ppp1r2
Hk1	4.27E-09	0.488563	0.585	0.306	8.60E-05	3 M1 _	Hk1
Tmem50b	4.21E-09	0.358806	0.39	0.155	8.47E-05	3 M1 _	Tmem50b
Picalm	4.18E-09	0.549458	0.793	0.583	8.43E-05	3 M1 _	Picalm
Birc6	4.17E-09	0.641134	0.768	0.563	8.39E-05	3 M1 _	Birc6
Cd2ap	3.85E-09	0.591544	0.78	0.569	7.75E-05	3 M1 _	Cd2ap
Fbxo22	3.80E-09	0.520791	0.549	0.275	7.66E-05	3 M1 _	Fbxo22
Trim30a	3.66E-09	0.564835	0.573	0.295	7.37E-05	3 M1 _	Trim30a

Pdlim5	3.50E-09	0.448079	0.585	0.315	7.04E-05	3 M1 _	Pdlim5
Iqsec2	3.46E-09	0.372407	0.317	0.109	6.97E-05	3 M1 _	Iqsec2
Smpdl3a1	3.34E-09	0.623336	0.744	0.471	6.72E-05	3 M1 _	Smpdl3a
Xpo1	3.30E-09	0.325943	0.5	0.225	6.65E-05	3 M1 _	Xpo1
Ralgapa1	3.11E-09	0.607909	0.61	0.319	6.26E-05	3 M1 _	Ralgapa1
Cd812	3.10E-09	0.455266	0.963	0.894	6.24E-05	3 M1 _	Cd81
Slc35b1	2.93E-09	0.552916	0.573	0.296	5.89E-05	3 M1 _	Slc35b1
Stx2	2.87E-09	0.526005	0.524	0.261	5.78E-05	3 M1 _	Stx2
Slc3a2	2.83E-09	0.640805	0.805	0.579	5.70E-05	3 M1 _	Slc3a2
Itm2b	2.79E-09	0.473966	1	0.99	5.61E-05	3 M1 _	Itm2b
Dusp12	2.78E-09	0.95082	0.598	0.329	5.59E-05	3 M1 _	Dusp1
Kcnb11	2.65E-09	0.346512	0.232	0.063	5.33E-05	3 M1 _	Kcnb1
Flywch1	2.63E-09	0.631988	0.573	0.293	5.29E-05	3 M1 _	Flywch1
Prex2	2.58E-09	0.624567	0.695	0.409	5.20E-05	3 M1 _	Prex2
Piezo1	2.28E-09	0.422995	0.341	0.126	4.59E-05	3 M1 _	Piezo1
Kcnq1	2.21E-09	0.309341	0.171	0.037	4.45E-05	3 M1 _	Kcnq1
Il10rb	2.19E-09	0.597438	0.622	0.348	4.42E-05	3 M1 _	Il10rb
Reep31	2.15E-09	0.591079	0.878	0.691	4.33E-05	3 M1 _	Reep3
Pip5k1c	2.12E-09	0.388814	0.451	0.186	4.26E-05	3 M1 _	Pip5k1c
Dram2	2.01E-09	0.459194	0.488	0.215	4.05E-05	3 M1 _	Dram2
Ablim3	1.92E-09	0.283815	0.146	0.028	3.86E-05	3 M1 _	Ablim3
BC0055371	1.89E-09	0.537672	0.72	0.442	3.80E-05	3 M1 _	BC005537
Irf2bpl	1.81E-09	0.606263	0.744	0.502	3.65E-05	3 M1 _	Irf2bpl
Nrbp1	1.69E-09	0.650811	0.744	0.488	3.41E-05	3 M1 _	Nrbp1
Ctnnd1	1.59E-09	0.546168	0.61	0.332	3.20E-05	3 M1 _	Ctnnd1
4833403J16Rik	1.58E-09	0.314798	0.22	0.056	3.18E-05	3 M1 _	4833403J16Rik
Arhgef10	1.56E-09	0.26032	0.378	0.134	3.14E-05	3 M1 _	Arhgef10
Tspan151	1.43E-09	0.340929	0.439	0.163	2.89E-05	3 M1 _	Tspan15
Csnk1a1	1.32E-09	0.508275	0.951	0.803	2.66E-05	3 M1 _	Csnk1a1
Casz1	1.14E-09	0.338086	0.146	0.028	2.30E-05	3 M1 _	Casz1
Ktn11	1.11E-09	0.54973	0.829	0.619	2.24E-05	3 M1 _	Ktn1
Mark4	1.02E-09	0.359747	0.378	0.14	2.05E-05	3 M1 _	Mark4
Hs3st1	1.01E-09	0.251617	0.183	0.04	2.03E-05	3 M1 _	Hs3st1
Kif13a	1.00E-09	0.3575	0.378	0.136	2.02E-05	3 M1 _	Kif13a
Adamts9	9.94E-10	0.538638	0.451	0.183	2.00E-05	3 M1 _	Adamts9
Psme1	9.78E-10	0.563337	0.902	0.724	1.97E-05	3 M1 _	Psme1
Ier2	9.55E-10	0.745917	0.744	0.449	1.92E-05	3 M1 _	Ier2
Mgat1	9.35E-10	0.539778	0.598	0.324	1.88E-05	3 M1 _	Mgat1
Sorbs1	8.60E-10	0.377115	0.427	0.161	1.73E-05	3 M1 _	Sorbs1
Npr2	8.50E-10	0.493324	0.378	0.149	1.71E-05	3 M1 _	Npr2
Git1	8.05E-10	0.383134	0.463	0.193	1.62E-05	3 M1 _	Git1

Myo10	7.69E-10	0.851677	0.695	0.445	1.55E-05	3 M1 _	Myo10
Fos	7.29E-10	1.546778	0.744	0.494	1.47E-05	3 M1 _	Fos
Tbx31	6.61E-10	0.437111	0.39	0.14	1.33E-05	3 M1 _	Tbx3
Zfp36	6.33E-10	0.898027	0.524	0.249	1.27E-05	3 M1 _	Zfp36
Gemin7	5.91E-10	0.518364	0.598	0.315	1.19E-05	3 M1 _	Gemin7
Tspan7	5.83E-10	0.369565	0.232	0.06	1.17E-05	3 M1 _	Tspan7
Sh3bgrl2	5.79E-10	0.275214	0.195	0.045	1.17E-05	3 M1 _	Sh3bgrl2
Abcc41	5.54E-10	0.277627	0.305	0.094	1.12E-05	3 M1 _	Abcc4
Per1	5.35E-10	0.350123	0.329	0.107	1.08E-05	3 M1 _	Per1
Stard4	5.32E-10	0.460587	0.305	0.097	1.07E-05	3 M1 _	Stard4
Itpr3	5.28E-10	0.416836	0.28	0.084	1.06E-05	3 M1 _	Itpr3
Dock1	5.19E-10	0.793184	0.622	0.375	1.05E-05	3 M1 _	Dock1
Hbegf1	4.02E-10	0.341594	0.317	0.098	8.09E-06	3 M1 _	Hbegf
Tjp2	3.97E-10	0.380033	0.293	0.089	7.99E-06	3 M1 _	Tjp2
Ttc39c	3.78E-10	0.387694	0.232	0.062	7.62E-06	3 M1 _	Ttc39c
Sppl2a	3.78E-10	0.657471	0.756	0.518	7.62E-06	3 M1 _	Sppl2a
Dennd5a	3.69E-10	0.492232	0.646	0.35	7.43E-06	3 M1 _	Dennd5a
Grb101	3.65E-10	0.665354	0.634	0.347	7.35E-06	3 M1 _	Grb10
S100a1	3.58E-10	0.595397	0.707	0.436	7.22E-06	3 M1 _	S100a1
4930523C07Rik1	3.14E-10	0.564475	0.732	0.425	6.33E-06	3 M1 _	4930523C07Rik
Gas6	3.04E-10	0.581227	0.89	0.559	6.13E-06	3 M1 _	Gas6
S100a13	2.84E-10	0.595373	0.939	0.752	5.71E-06	3 M1 _	S100a13
Nedd9	2.63E-10	0.681458	0.622	0.343	5.30E-06	3 M1 _	Nedd9
Rgl1	2.46E-10	0.631469	0.72	0.455	4.95E-06	3 M1 _	Rgl1
Msi2	2.16E-10	0.667896	0.61	0.311	4.36E-06	3 M1 _	Msi2
Fam219a	1.83E-10	0.284759	0.317	0.098	3.68E-06	3 M1 _	Fam219a
Ifi208	1.78E-10	0.308253	0.256	0.067	3.58E-06	3 M1 _	Ifi208
Foxf1	1.74E-10	0.263618	0.171	0.033	3.50E-06	3 M1 _	Foxf1
Psmb8	1.69E-10	0.604835	0.902	0.623	3.41E-06	3 M1 _	Psmb8
Mcl1	1.69E-10	0.629093	0.866	0.622	3.40E-06	3 M1 _	Mcl1
Arid5b	1.68E-10	0.760517	0.829	0.602	3.38E-06	3 M1 _	Arid5b
Samd9l	1.47E-10	0.64051	0.695	0.362	2.96E-06	3 M1 _	Samd9l
Fbln21	1.39E-10	0.917473	0.805	0.537	2.80E-06	3 M1 _	Fbln2
Tshz2	1.35E-10	0.65655	0.89	0.633	2.71E-06	3 M1 _	Tshz2
Xdh1	1.27E-10	0.673199	0.744	0.422	2.56E-06	3 M1 _	Xdh
Map3k5	1.22E-10	0.375555	0.415	0.15	2.46E-06	3 M1 _	Map3k5
Stap2	1.17E-10	0.285539	0.195	0.042	2.37E-06	3 M1 _	Stap2
Dock6	1.14E-10	0.40785	0.476	0.188	2.29E-06	3 M1 _	Dock6
Dip2b	1.12E-10	0.545082	0.549	0.251	2.25E-06	3 M1 _	Dip2b
Plxna4	1.11E-10	0.337808	0.159	0.029	2.23E-06	3 M1 _	Plxna4
Zfp715	1.08E-10	0.350006	0.305	0.09	2.18E-06	3 M1 _	Zfp715

Grasp	1.05E-10	0.545465	0.512	0.226	2.12E-06	3 M1 _	Grasp
Arhgef5	9.43E-11	0.535811	0.439	0.175	1.90E-06	3 M1 _	Arhgef5
Ptbp3	9.16E-11	0.72214	0.793	0.583	1.85E-06	3 M1 _	Ptbp3
Etnk1	8.63E-11	0.707373	0.72	0.432	1.74E-06	3 M1 _	Etnk1
Sgms1	8.14E-11	0.398642	0.5	0.201	1.64E-06	3 M1 _	Sgms1
Gbp9	7.79E-11	0.477897	0.451	0.174	1.57E-06	3 M1 _	Gbp9
Zmiz1	7.70E-11	0.636664	0.78	0.503	1.55E-06	3 M1 _	Zmiz1
B3gnt3	7.49E-11	0.35004	0.256	0.066	1.51E-06	3 M1 _	B3gnt3
Gatad2a	7.37E-11	0.635438	0.622	0.321	1.48E-06	3 M1 _	Gatad2a
Bag5	7.12E-11	0.534768	0.707	0.393	1.43E-06	3 M1 _	Bag5
Lyn	6.88E-11	0.476228	0.463	0.183	1.39E-06	3 M1 _	Lyn
Zfp664	6.02E-11	0.519197	0.598	0.298	1.21E-06	3 M1 _	Zfp664
Gbp7	5.96E-11	0.820253	0.732	0.444	1.20E-06	3 M1 _	Gbp7
Trim47	5.92E-11	0.648606	0.695	0.397	1.19E-06	3 M1 _	Trim47
Cit	5.91E-11	0.303915	0.146	0.024	1.19E-06	3 M1 _	Cit
Rap1b	5.81E-11	0.707846	0.854	0.681	1.17E-06	3 M1 _	Rap1b
Elf1	5.04E-11	0.653793	0.756	0.456	1.02E-06	3 M1 _	Elf1
St3gal4	4.93E-11	0.413169	0.512	0.214	9.92E-07	3 M1 _	St3gal4
Sh3bp5	4.77E-11	0.624448	0.549	0.247	9.60E-07	3 M1 _	Sh3bp5
Ywhaq1	4.76E-11	0.543547	0.878	0.795	9.58E-07	3 M1 _	Ywhaq
Fnbp1l	4.26E-11	0.582755	0.476	0.195	8.59E-07	3 M1 _	Fnbp1l
Tlnrd1	4.20E-11	0.370516	0.427	0.157	8.46E-07	3 M1 _	Tlnrd1
Cactin	4.11E-11	0.400872	0.366	0.123	8.27E-07	3 M1 _	Cactin
Nfe2l1	4.06E-11	0.803198	0.817	0.636	8.18E-07	3 M1 _	Nfe2l1
Stk10	4.02E-11	0.423078	0.415	0.146	8.09E-07	3 M1 _	Stk10
Syt7	3.89E-11	0.273782	0.11	0.014	7.84E-07	3 M1 _	Syt7
Cttnbp2nl	3.84E-11	0.50269	0.402	0.147	7.74E-07	3 M1 _	Cttnbp2nl
Erg	3.83E-11	0.30228	0.354	0.112	7.72E-07	3 M1 _	Erg
Atp8a1	3.54E-11	0.389662	0.378	0.125	7.13E-07	3 M1 _	Atp8a1
Ada	3.35E-11	0.710264	0.293	0.088	6.74E-07	3 M1 _	Ada
Spry41	3.28E-11	0.587332	0.549	0.257	6.60E-07	3 M1 _	Spry4
Atp1a1	3.26E-11	0.720154	0.732	0.431	6.57E-07	3 M1 _	Atp1a1
Cep85l	3.02E-11	0.530878	0.378	0.129	6.08E-07	3 M1 _	Cep85l
Atox1	3.01E-11	0.612751	0.902	0.78	6.06E-07	3 M1 _	Atox1
Tmem184b	2.50E-11	0.490307	0.488	0.201	5.03E-07	3 M1 _	Tmem184b
H2afy2	2.26E-11	0.455352	0.378	0.128	4.56E-07	3 M1 _	H2afy2
Tpm3	2.25E-11	0.696982	0.915	0.746	4.54E-07	3 M1 _	Tpm3
Arhgef28	2.05E-11	0.253523	0.195	0.039	4.13E-07	3 M1 _	Arhgef28
Nedd4l	2.03E-11	0.430819	0.305	0.089	4.09E-07	3 M1 _	Nedd4l
Sos1	1.93E-11	0.576781	0.476	0.191	3.90E-07	3 M1 _	Sos1
Galnt7	1.83E-11	0.418818	0.463	0.175	3.68E-07	3 M1 _	Galnt7

Cdc14a	1.82E-11	0.369506	0.329	0.098	3.66E-07	3 M1 _	Cdc14a
Csde11	1.70E-11	0.589352	0.866	0.697	3.43E-07	3 M1 _	Csde1
Rtl8b	1.67E-11	0.65404	0.598	0.284	3.37E-07	3 M1 _	Rtl8b
Edn1	1.60E-11	1.158525	0.159	0.028	3.22E-07	3 M1 _	Edn1
Cd274	1.36E-11	0.337958	0.256	0.063	2.74E-07	3 M1 _	Cd274
Fgd6	1.32E-11	0.293604	0.22	0.048	2.66E-07	3 M1 _	Fgd6
Arl15	1.14E-11	0.490492	0.366	0.122	2.29E-07	3 M1 _	Arl15
Cyb561	1.09E-11	0.388385	0.329	0.098	2.19E-07	3 M1 _	Cyb561
Sh3glb1	9.46E-12	0.61629	0.915	0.81	1.91E-07	3 M1 _	Sh3glb1
Rala	9.06E-12	0.587627	0.744	0.438	1.82E-07	3 M1 _	Rala
Mecom	8.82E-12	0.568641	0.28	0.073	1.78E-07	3 M1 _	Mecom
Rac1	7.99E-12	0.646475	0.939	0.754	1.61E-07	3 M1 _	Rac1
Pik3cb	7.39E-12	0.273776	0.22	0.047	1.49E-07	3 M1 _	Pik3cb
Serinc3	6.63E-12	0.718485	0.902	0.858	1.33E-07	3 M1 _	Serinc3
Ndufa4	6.47E-12	0.670282	0.915	0.73	1.30E-07	3 M1 _	Ndufa4
Midn	5.56E-12	0.664749	0.659	0.351	1.12E-07	3 M1 _	Midn
Larp1b	5.13E-12	0.544463	0.39	0.128	1.03E-07	3 M1 _	Larp1b
Ggta11	4.74E-12	0.303122	0.39	0.119	9.54E-08	3 M1 _	Ggta1
Kras	4.23E-12	0.566317	0.756	0.436	8.53E-08	3 M1 _	Kras
Ptk21	3.91E-12	0.525426	0.671	0.34	7.87E-08	3 M1 _	Ptk2
St3gal11	3.71E-12	0.553306	0.524	0.225	7.48E-08	3 M1 _	St3gal1
Fmnl3	3.44E-12	0.644506	0.598	0.288	6.93E-08	3 M1 _	Fmnl3
Adipor1	3.21E-12	0.78695	0.695	0.424	6.47E-08	3 M1 _	Adipor1
Baz1a	3.10E-12	0.52563	0.439	0.159	6.24E-08	3 M1 _	Baz1a
Magi1	2.98E-12	0.369009	0.244	0.055	6.00E-08	3 M1 _	Magi1
Stmn1	2.65E-12	0.504512	0.341	0.102	5.34E-08	3 M1 _	Stmn1
Fam181b	2.57E-12	0.373614	0.207	0.042	5.18E-08	3 M1 _	Fam181b
Rtn3	2.35E-12	0.688385	0.829	0.604	4.74E-08	3 M1 _	Rtn3
Gda	2.25E-12	0.469293	0.293	0.075	4.53E-08	3 M1 _	Gda
Tmed5	2.05E-12	0.832382	0.72	0.404	4.12E-08	3 M1 _	Tmed5
Col4a3bp	2.01E-12	0.652231	0.671	0.358	4.05E-08	3 M1 _	Col4a3bp
Nxf12	1.99E-12	0.547336	0.598	0.264	4.00E-08	3 M1 _	Nxf1
Sh3rf1	1.95E-12	0.347377	0.354	0.104	3.93E-08	3 M1 _	Sh3rf1
Gse1	1.54E-12	0.444494	0.402	0.128	3.10E-08	3 M1 _	Gse1
Tnk2	1.54E-12	0.550859	0.476	0.181	3.09E-08	3 M1 _	Tnk2
Ift172	1.47E-12	0.49909	0.354	0.107	2.96E-08	3 M1 _	Ift172
Coro1b2	1.28E-12	0.394808	0.341	0.096	2.57E-08	3 M1 _	Coro1b
Pxn	1.26E-12	0.544896	0.512	0.2	2.55E-08	3 M1 _	Pxn
Foxo1	1.01E-12	0.570789	0.61	0.277	2.03E-08	3 M1 _	Foxo1
Fam57a	9.86E-13	0.362729	0.195	0.036	1.99E-08	3 M1 _	Fam57a
Srf	9.01E-13	0.344088	0.244	0.054	1.81E-08	3 M1 _	Srf

Ywhab1	8.69E-13	0.609412	0.963	0.829	1.75E-08	3 M1 _	Ywhab
Zfp46	6.39E-13	0.411075	0.378	0.118	1.29E-08	3 M1 _	Zfp46
Dusp16	6.30E-13	0.378771	0.256	0.058	1.27E-08	3 M1 _	Dusp16
Chmp3	5.45E-13	0.666397	0.841	0.559	1.10E-08	3 M1 _	Chmp3
Tanc1	5.18E-13	0.61368	0.659	0.309	1.04E-08	3 M1 _	Tanc1
Dusp7	4.97E-13	0.491217	0.512	0.193	1.00E-08	3 M1 _	Dusp7
Tns2	4.93E-13	0.689741	0.671	0.34	9.93E-09	3 M1 _	Tns2
Rbms1	4.67E-13	0.758353	0.927	0.774	9.40E-09	3 M1 _	Rbms1
Arhgap261	4.00E-13	0.309686	0.28	0.066	8.05E-09	3 M1 _	Arhgap26
Dock9	3.77E-13	0.482222	0.549	0.218	7.59E-09	3 M1 _	Dock9
Mapk12	3.54E-13	0.449937	0.427	0.138	7.13E-09	3 M1 _	Mapk12
Sigirr	3.40E-13	0.372986	0.329	0.087	6.85E-09	3 M1 _	Sigirr
Fam129b	3.27E-13	0.521349	0.537	0.215	6.58E-09	3 M1 _	Fam129b
Sgk3	3.12E-13	0.480494	0.402	0.126	6.28E-09	3 M1 _	Sgk3
Cd59a	3.07E-13	0.330792	0.39	0.116	6.17E-09	3 M1 _	Cd59a
Capn2	2.90E-13	0.692838	0.768	0.45	5.83E-09	3 M1 _	Capn2
Dync1i21	2.73E-13	0.642934	0.915	0.782	5.50E-09	3 M1 _	Dync1i2
Pls3	2.70E-13	0.834723	0.854	0.586	5.43E-09	3 M1 _	Pls3
Ptpn14	2.57E-13	0.499513	0.378	0.117	5.17E-09	3 M1 _	Ptpn14
Crybg3	2.17E-13	0.637652	0.585	0.252	4.38E-09	3 M1 _	Crybg3
Lipa	2.17E-13	0.865056	0.768	0.481	4.37E-09	3 M1 _	Lipa
Purb	2.04E-13	0.751044	0.902	0.668	4.11E-09	3 M1 _	Purb
Gm424182	1.72E-13	0.943592	1	0.999	3.46E-09	3 M1 _	Gm42418
Wwc2	1.64E-13	0.68531	0.768	0.458	3.31E-09	3 M1 _	Wwc2
Ints9	1.42E-13	0.411823	0.402	0.125	2.86E-09	3 M1 _	Ints9
Fam212b1	1.31E-13	0.259148	0.146	0.019	2.64E-09	3 M1 _	Fam212b
Amotl2	1.18E-13	0.6155	0.537	0.217	2.38E-09	3 M1 _	Amotl2
Actg1	1.08E-13	0.680657	0.988	0.944	2.17E-09	3 M1 _	Actg1
Tmem140	1.06E-13	0.693134	0.756	0.406	2.13E-09	3 M1 _	Tmem140
Lmo7	1.01E-13	0.404459	0.256	0.054	2.03E-09	3 M1 _	Lmo7
Oaz21	9.33E-14	0.874412	0.732	0.437	1.88E-09	3 M1 _	Oaz2
Tmsb4x1	9.23E-14	0.529063	1	0.994	1.86E-09	3 M1 _	Tmsb4x
Nov	7.66E-14	0.309448	0.11	0.011	1.54E-09	3 M1 _	Nov
Diaph2	7.56E-14	0.868089	0.695	0.394	1.52E-09	3 M1 _	Diaph2
Macf1	7.00E-14	0.775673	0.939	0.807	1.41E-09	3 M1 _	Macf1
Hoxd3os1	6.91E-14	0.363879	0.232	0.045	1.39E-09	3 M1 _	Hoxd3os1
Ankrd33b	6.49E-14	0.291665	0.232	0.044	1.31E-09	3 M1 _	Ankrd33b
Hpcal1	6.29E-14	0.6901	0.707	0.354	1.27E-09	3 M1 _	Hpcal1
Ncoa3	6.22E-14	0.748167	0.634	0.307	1.25E-09	3 M1 _	Ncoa3
Lmcd1	5.89E-14	0.391111	0.512	0.175	1.19E-09	3 M1 _	Lmcd1
Nr4a1	4.95E-14	0.404237	0.293	0.068	9.97E-10	3 M1 _	Nr4a1

Cdc42ep1	4.78E-14	0.647377	0.61	0.258	9.63E-10	3 M1 _	Cdc42ep1
Stom1	4.33E-14	0.511136	0.476	0.157	8.73E-10	3 M1 _	Stom
Cyp26b11	2.41E-14	0.57901	0.366	0.102	4.86E-10	3 M1 _	Cyp26b1
Vwa1	2.21E-14	0.913421	0.659	0.327	4.45E-10	3 M1 _	Vwa1
Etv5	2.21E-14	0.574794	0.451	0.157	4.44E-10	3 M1 _	Etv5
Zufsp3	1.92E-14	0.271948	0.195	0.031	3.86E-10	3 M1 _	Zufsp
Zfpm1	1.79E-14	0.609255	0.488	0.175	3.60E-10	3 M1 _	Zfpm1
9530082P21Rik	1.69E-14	0.480523	0.366	0.101	3.40E-10	3 M1 _	9530082P21Rik
Pitpnc1	1.58E-14	0.702464	0.72	0.362	3.19E-10	3 M1 _	Pitpnc1
Sec14l1	1.49E-14	0.676928	0.683	0.336	3.00E-10	3 M1 _	Sec14l1
Xiap	1.41E-14	0.830366	0.817	0.517	2.84E-10	3 M1 _	Xiap
Cyb5r3	1.23E-14	0.681667	0.866	0.573	2.48E-10	3 M1 _	Cyb5r3
Pqlc1	1.21E-14	0.589676	0.524	0.197	2.43E-10	3 M1 _	Pqlc1
Fkbp3	1.16E-14	0.905507	0.805	0.589	2.33E-10	3 M1 _	Fkbp3
Jdp2	1.04E-14	0.606421	0.585	0.237	2.09E-10	3 M1 _	Jdp2
Fbxw2	9.23E-15	0.728407	0.78	0.425	1.86E-10	3 M1 _	Fbxw2
Sypl	8.87E-15	0.700558	0.817	0.46	1.79E-10	3 M1 _	Sypl
Tjp1	7.53E-15	0.695581	0.744	0.386	1.52E-10	3 M1 _	Tjp1
Nes1	6.60E-15	0.762913	0.573	0.22	1.33E-10	3 M1 _	Nes
Rhoa	6.22E-15	0.587989	0.963	0.901	1.25E-10	3 M1 _	Rhoa
Rassf9	6.04E-15	0.677514	0.39	0.11	1.22E-10	3 M1 _	Rassf9
Fam171a1	5.67E-15	0.627629	0.463	0.151	1.14E-10	3 M1 _	Fam171a1
Apbb2	5.39E-15	0.743863	0.805	0.461	1.09E-10	3 M1 _	Apbb2
Papss2	5.03E-15	0.499724	0.366	0.102	1.01E-10	3 M1 _	Papss2
Fmo2	4.97E-15	0.979778	0.793	0.455	1.00E-10	3 M1 _	Fmo2
Tspan14	4.56E-15	0.318871	0.366	0.093	9.19E-11	3 M1 _	Tspan14
Plekhg1	4.11E-15	0.542496	0.5	0.176	8.28E-11	3 M1 _	Plekhg1
Dcbld1	4.05E-15	0.388714	0.305	0.068	8.17E-11	3 M1 _	Dcbld1
Acvr2a1	3.91E-15	0.606843	0.488	0.164	7.87E-11	3 M1 _	Acvr2a
Cds21	3.36E-15	0.758087	0.659	0.293	6.77E-11	3 M1 _	Cds2
Glul	3.17E-15	1.349189	0.61	0.288	6.39E-11	3 M1 _	Glul
Gimap1	2.87E-15	0.536632	0.683	0.258	5.79E-11	3 M1 _	Gimap1
Trib21	2.59E-15	0.889281	0.695	0.343	5.21E-11	3 M1 _	Trib2
Rab11a	2.33E-15	0.748311	0.756	0.433	4.70E-11	3 M1 _	Rab11a
Arl4d	2.31E-15	0.453303	0.244	0.046	4.65E-11	3 M1 _	Arl4d
Eif4ebp21	2.30E-15	0.851998	0.793	0.48	4.63E-11	3 M1 _	Eif4ebp2
Cavin11	2.27E-15	0.870422	0.793	0.473	4.57E-11	3 M1 _	Cavin1
Fnip2	1.94E-15	0.456102	0.354	0.091	3.90E-11	3 M1 _	Fnip2
Zdhhc18	1.93E-15	0.555464	0.549	0.199	3.88E-11	3 M1 _	Zdhhc18
Pde8a	1.87E-15	0.546568	0.537	0.191	3.76E-11	3 M1 _	Pde8a
Mlec1	1.69E-15	0.893517	0.841	0.528	3.41E-11	3 M1 _	Mlec

Limch1	1.65E-15	0.479411	0.329	0.077	3.33E-11	3 M1 _	Limch1
Sorbs3	1.65E-15	0.609051	0.463	0.156	3.32E-11	3 M1 _	Sorbs3
Numb	1.63E-15	0.686802	0.61	0.258	3.29E-11	3 M1 _	Numb
Tcf41	1.58E-15	0.741978	1	0.885	3.17E-11	3 M1 _	Tcf4
Tubb4b1	1.47E-15	0.911767	0.915	0.695	2.95E-11	3 M1 _	Tubb4b
Itsn2	1.43E-15	0.713706	0.671	0.304	2.89E-11	3 M1 _	Itsn2
Lpar6	1.23E-15	0.713708	0.524	0.184	2.49E-11	3 M1 _	Lpar6
Colgalt2	1.18E-15	0.545113	0.402	0.114	2.38E-11	3 M1 _	Colgalt2
Gimap6	1.14E-15	0.682397	0.817	0.391	2.30E-11	3 M1 _	Gimap6
Ipo11	1.10E-15	0.544778	0.415	0.119	2.21E-11	3 M1 _	Ipo11
Tsc22d31	1.05E-15	0.80086	0.817	0.467	2.12E-11	3 M1 _	Tsc22d3
Lfng	1.01E-15	0.82578	0.5	0.179	2.04E-11	3 M1 _	Lfng
AU021092	1.01E-15	0.289877	0.159	0.02	2.03E-11	3 M1 _	AU021092
Prdm1	1.01E-15	0.498114	0.354	0.087	2.03E-11	3 M1 _	Prdm1
Klf12	9.99E-16	0.396534	0.341	0.083	2.01E-11	3 M1 _	Klf12
Epha4	9.72E-16	0.698276	0.512	0.184	1.96E-11	3 M1 _	Epha4
Ptp4a13	8.20E-16	0.301906	0.305	0.065	1.65E-11	3 M1 _	Ptp4a1
Rnf125	7.17E-16	0.400406	0.317	0.072	1.44E-11	3 M1 _	Rnf125
Itga11	6.55E-16	0.730884	0.976	0.794	1.32E-11	3 M1 _	Itga1
Gimap4	6.16E-16	0.548559	0.744	0.291	1.24E-11	3 M1 _	Gimap4
Ctnnb1	5.01E-16	0.814351	0.878	0.696	1.01E-11	3 M1 _	Ctnnb1
Tmem204	4.92E-16	0.806114	0.902	0.569	9.92E-12	3 M1 _	Tmem204
Atoh8	4.88E-16	0.38237	0.195	0.029	9.82E-12	3 M1 _	Atoh8
Ostf11	4.15E-16	0.77471	0.878	0.602	8.35E-12	3 M1 _	Ostf1
Gnaq1	3.90E-16	0.739489	0.78	0.434	7.85E-12	3 M1 _	Gnaq
Adam10	3.81E-16	0.719547	0.695	0.311	7.68E-12	3 M1 _	Adam10
Ehd21	3.22E-16	0.973922	0.768	0.477	6.49E-12	3 M1 _	Ehd2
Upp1	3.20E-16	0.44814	0.317	0.069	6.44E-12	3 M1 _	Upp1
Ica1	2.83E-16	0.431859	0.268	0.052	5.70E-12	3 M1 _	Ica1
Tpst2	2.74E-16	0.572767	0.695	0.292	5.52E-12	3 M1 _	Tpst2
Rhob2	2.48E-16	1.147425	0.854	0.609	4.99E-12	3 M1 _	Rhob
Tgm2	2.36E-16	0.835224	0.671	0.297	4.75E-12	3 M1 _	Tgm2
Cd471	2.33E-16	0.737883	0.976	0.859	4.68E-12	3 M1 _	Cd47
Slc28a2	2.30E-16	0.459565	0.256	0.047	4.63E-12	3 M1 _	Slc28a2
Endod1	2.22E-16	0.840297	0.72	0.368	4.47E-12	3 M1 _	Endod1
Snai2	2.21E-16	0.646825	0.561	0.205	4.45E-12	3 M1 _	Snai2
Col4a21	2.16E-16	0.819182	0.988	0.852	4.36E-12	3 M1 _	Col4a2
Bnip2	2.05E-16	0.861815	0.854	0.58	4.13E-12	3 M1 _	Bnip2
Tmem44	2.04E-16	0.315688	0.244	0.042	4.11E-12	3 M1 _	Tmem44
Rap1a1	2.04E-16	0.78853	0.89	0.645	4.11E-12	3 M1 _	Rap1a
Notch1	1.88E-16	0.940114	0.683	0.337	3.79E-12	3 M1 _	Notch1

St3gal6	1.85E-16	0.703155	0.61	0.22	3.73E-12	3 M1 _	St3gal6
Dab2ip1	1.81E-16	0.816545	0.829	0.485	3.65E-12	3 M1 _	Dab2ip
Slc30a1	1.63E-16	0.524911	0.415	0.114	3.29E-12	3 M1 _	Slc30a1
Slfn3	1.58E-16	0.413704	0.171	0.022	3.18E-12	3 M1 _	Slfn3
Tnfrsf10b	1.51E-16	0.312935	0.256	0.047	3.04E-12	3 M1 _	Tnfrsf10b
Map4k3	1.36E-16	0.629213	0.585	0.219	2.73E-12	3 M1 _	Map4k3
Myo6	1.30E-16	0.838941	0.72	0.343	2.61E-12	3 M1 _	Myo6
Ets2	1.24E-16	0.588527	0.646	0.247	2.50E-12	3 M1 _	Ets2
Cd1511	1.18E-16	0.706882	0.866	0.55	2.38E-12	3 M1 _	Cd151
Sncaip	1.13E-16	0.271868	0.207	0.031	2.28E-12	3 M1 _	Sncaip
Nxpe2	1.11E-16	0.487998	0.22	0.035	2.24E-12	3 M1 _	Nxpe2
Kdelc22	1.03E-16	0.385042	0.317	0.066	2.07E-12	3 M1 _	Kdelc2
Pcdh12	6.12E-17	0.565478	0.488	0.152	1.23E-12	3 M1 _	Pcdh12
Adamts11	5.75E-17	1.077478	0.707	0.33	1.16E-12	3 M1 _	Adamts1
Ptma	5.07E-17	0.610598	1	0.986	1.02E-12	3 M1 _	Ptma
Gimap8	5.01E-17	0.598009	0.439	0.119	1.01E-12	3 M1 _	Gimap8
Cdipt	4.65E-17	0.750422	0.671	0.291	9.36E-13	3 M1 _	Cdipt
Ldb2	4.63E-17	0.902037	0.732	0.37	9.32E-13	3 M1 _	Ldb2
Kif5b	4.25E-17	0.85518	0.878	0.674	8.57E-13	3 M1 _	Kif5b
Sema6b	3.43E-17	0.386056	0.256	0.046	6.91E-13	3 M1 _	Sema6b
Dhrs31	2.79E-17	0.841118	0.817	0.407	5.61E-13	3 M1 _	Dhrs3
Pik3c2a	2.13E-17	0.734435	0.671	0.283	4.29E-13	3 M1 _	Pik3c2a
Mlf1	1.90E-17	0.358204	0.22	0.033	3.83E-13	3 M1 _	Mlf1
Rps6ka3	1.83E-17	0.822195	0.671	0.292	3.69E-13	3 M1 _	Rps6ka3
Sod11	1.28E-17	0.957422	0.829	0.524	2.58E-13	3 M1 _	Sod1
Elk3	1.18E-17	1.009282	0.817	0.488	2.37E-13	3 M1 _	Elk3
Tcf7l1	1.16E-17	0.82666	0.671	0.287	2.34E-13	3 M1 _	Tcf7l1
9930111J21Rik21	1.05E-17	0.817481	0.841	0.428	2.12E-13	3 M1 _	9930111J21Rik2
Afdn	8.97E-18	1.092136	0.744	0.398	1.81E-13	3 M1 _	Afdn
Lpcat1	8.30E-18	0.701691	0.561	0.193	1.67E-13	3 M1 _	Lpcat1
Bcl2l1	8.21E-18	0.704193	0.537	0.183	1.65E-13	3 M1 _	Bcl2l1
Cpd1	8.13E-18	0.939305	0.793	0.473	1.64E-13	3 M1 _	Cpd
Sh2d3c	7.59E-18	0.620447	0.476	0.14	1.53E-13	3 M1 _	Sh2d3c
Ndufc2	7.02E-18	0.924088	0.951	0.703	1.41E-13	3 M1 _	Ndufc2
Rai14	6.61E-18	0.894644	0.72	0.345	1.33E-13	3 M1 _	Rai14
Crip21	6.26E-18	0.837985	0.951	0.765	1.26E-13	3 M1 _	Crip2
Cyb5r1	5.62E-18	0.526424	0.415	0.106	1.13E-13	3 M1 _	Cyb5r1
Col4a12	4.72E-18	0.872841	1	0.869	9.51E-14	3 M1 _	Col4a1
Anp32a	4.68E-18	1.01488	0.878	0.657	9.42E-14	3 M1 _	Anp32a
Gbp4	4.56E-18	0.629366	0.549	0.176	9.18E-14	3 M1 _	Gbp4
Fam117b	4.32E-18	0.801797	0.585	0.222	8.71E-14	3 M1 _	Fam117b

Luzp1	4.28E-18	0.856862	0.878	0.59	8.61E-14	3 M1 _	Luzp1
Iqgap1	3.90E-18	0.92114	0.951	0.751	7.85E-14	3 M1 _	Iqgap1
Pdgfd	3.31E-18	0.540773	0.415	0.106	6.66E-14	3 M1 _	Pdgfd
Ctnnbip1	3.26E-18	0.786071	0.659	0.269	6.57E-14	3 M1 _	Ctnnbip1
Tspan18	2.20E-18	0.512123	0.402	0.101	4.43E-14	3 M1 _	Tspan18
Lats2	2.10E-18	0.834137	0.707	0.324	4.24E-14	3 M1 _	Lats2
Car7	1.96E-18	0.496752	0.22	0.032	3.95E-14	3 M1 _	Car7
Cmip	1.78E-18	0.822933	0.634	0.255	3.58E-14	3 M1 _	Cmip
Tpm41	1.53E-18	1.017727	0.902	0.751	3.07E-14	3 M1 _	Tpm4
Ifi44	1.50E-18	0.680734	0.549	0.166	3.02E-14	3 M1 _	Ifi44
Cebpg	1.32E-18	0.837788	0.768	0.393	2.66E-14	3 M1 _	Cebpg
Tmtc2	1.18E-18	0.336355	0.22	0.031	2.38E-14	3 M1 _	Tmtc2
2900026A02Rik	9.55E-19	0.789148	0.707	0.291	1.92E-14	3 M1 _	2900026A02Rik
Gcnt21	9.45E-19	0.558847	0.622	0.191	1.90E-14	3 M1 _	Gcnt2
3110062M04Rik2	8.96E-19	0.295227	0.22	0.031	1.80E-14	3 M1 _	3110062M04Rik
Irx3	8.09E-19	0.436856	0.305	0.057	1.63E-14	3 M1 _	Irx3
Thsd1	6.32E-19	0.575131	0.305	0.058	1.27E-14	3 M1 _	Thsd1
Wars	4.52E-19	0.781175	0.549	0.186	9.10E-15	3 M1 _	Wars
Piezo2	4.12E-19	0.967147	0.744	0.346	8.29E-15	3 M1 _	Piezo2
Efna1	3.00E-19	0.543818	0.463	0.118	6.04E-15	3 M1 _	Efna1
Sox7	2.25E-19	0.40638	0.256	0.04	4.54E-15	3 M1 _	Sox7
Rtl8a	2.08E-19	1.213659	0.793	0.477	4.19E-15	3 M1 _	Rtl8a
Csrnp1	1.27E-19	0.386753	0.256	0.041	2.56E-15	3 M1 _	Csrnp1
Qk	1.18E-19	0.930914	0.902	0.675	2.38E-15	3 M1 _	Qk
AY0361182	1.01E-19	1.685148	0.89	0.555	2.03E-15	3 M1 _	AY036118
Adgrl2	9.84E-20	0.886933	0.683	0.27	1.98E-15	3 M1 _	Adgrl2
Ier3	9.62E-20	0.969444	0.512	0.152	1.94E-15	3 M1 _	Ier3
Utrn1	9.03E-20	1.061699	0.854	0.478	1.82E-15	3 M1 _	Utrn
Phactr4	8.63E-20	0.82145	0.622	0.23	1.74E-15	3 M1 _	Phactr4
Nck1	8.53E-20	1.06806	0.829	0.513	1.72E-15	3 M1 _	Nck1
Ndufa8	7.37E-20	0.947702	0.89	0.594	1.48E-15	3 M1 _	Ndufa8
Spag72	5.93E-20	0.628729	0.561	0.17	1.19E-15	3 M1 _	Spag7
Eepd1	5.87E-20	0.42185	0.256	0.039	1.18E-15	3 M1 _	Eepd1
Stc1	5.32E-20	0.660552	0.5	0.133	1.07E-15	3 M1 _	Stc1
Klf7	4.64E-20	1.081865	0.866	0.539	9.34E-16	3 M1 _	Klf7
Jam31	4.35E-20	1.068704	0.78	0.393	8.77E-16	3 M1 _	Jam3
Spata13	4.21E-20	0.548003	0.415	0.098	8.48E-16	3 M1 _	Spata13
Clu	3.63E-20	0.846916	0.463	0.122	7.31E-16	3 M1 _	Clu
Igfbp7	3.54E-20	0.833601	0.988	0.943	7.13E-16	3 M1 _	Igfbp7
Hspa12b	3.37E-20	0.40735	0.354	0.072	6.79E-16	3 M1 _	Hspa12b
Jcad	3.37E-20	0.759265	0.622	0.222	6.79E-16	3 M1 _	Jcad

Rgs12	2.96E-20	0.596263	0.476	0.132	5.96E-16	3 M1 _	Rgs12
App1	2.91E-20	0.906957	0.988	0.835	5.87E-16	3 M1 _	App
Dgkh	2.71E-20	0.744953	0.622	0.211	5.45E-16	3 M1 _	Dgkh
Fhod1	2.66E-20	0.504957	0.317	0.058	5.36E-16	3 M1 _	Fhod1
St8sia4	2.31E-20	0.774759	0.634	0.208	4.66E-16	3 M1 _	St8sia4
Lama5	2.22E-20	0.376613	0.329	0.061	4.48E-16	3 M1 _	Lama5
Arhgef12	2.01E-20	1.06404	0.878	0.663	4.04E-16	3 M1 _	Arhgef12
Mertk	1.55E-20	0.65916	0.5	0.137	3.13E-16	3 M1 _	Mertk
Plxnd1	1.23E-20	1.063538	0.78	0.369	2.47E-16	3 M1 _	Plxnd1
Unc119b	1.19E-20	0.947023	0.61	0.227	2.39E-16	3 M1 _	Unc119b
Lars22	1.12E-20	1.244504	0.976	0.808	2.26E-16	3 M1 _	Lars2
Slc12a7	9.47E-21	0.416506	0.354	0.07	1.91E-16	3 M1 _	Slc12a7
Smad7	9.26E-21	1.007337	0.829	0.398	1.87E-16	3 M1 _	Smad7
Abcb1a	8.15E-21	0.677033	0.415	0.096	1.64E-16	3 M1 _	Abcb1a
Syn31	8.14E-21	0.689872	0.427	0.1	1.64E-16	3 M1 _	Syn3
Nav2	7.42E-21	0.575256	0.354	0.071	1.49E-16	3 M1 _	Nav2
BC028528	7.35E-21	0.762964	0.598	0.189	1.48E-16	3 M1 _	BC028528
Gnb1	7.01E-21	0.871239	0.927	0.686	1.41E-16	3 M1 _	Gnb1
Ppm1f	6.67E-21	0.52477	0.378	0.083	1.34E-16	3 M1 _	Ppm1f
Epb411	4.25E-21	0.557511	0.573	0.165	8.55E-17	3 M1 _	Epb41
Plcg1	3.53E-21	0.693606	0.671	0.243	7.12E-17	3 M1 _	Plcg1
Ripor1	2.18E-21	0.869106	0.671	0.259	4.39E-17	3 M1 _	Ripor1
Scarf1	2.13E-21	0.415114	0.305	0.051	4.30E-17	3 M1 _	Scarf1
Fam69a2	1.87E-21	0.295794	0.329	0.057	3.77E-17	3 M1 _	Fam69a
Mapk3	1.26E-21	0.937121	0.902	0.537	2.53E-17	3 M1 _	Mapk3
Pparg1	1.08E-21	0.341735	0.293	0.045	2.18E-17	3 M1 _	Pparg
Rnf144b	1.08E-21	0.280979	0.195	0.021	2.17E-17	3 M1 _	Rnf144b
Fut4	9.52E-22	0.53091	0.28	0.044	1.92E-17	3 M1 _	Fut4
Tmem55b2	8.15E-22	0.322928	0.28	0.043	1.64E-17	3 M1 _	Tmem55b
Ccnyl1	7.30E-22	0.577622	0.366	0.074	1.47E-17	3 M1 _	Ccnyl1
Map3k11	5.62E-22	0.557824	0.427	0.098	1.13E-17	3 M1 _	Map3k11
Myo1c	5.00E-22	0.98708	0.805	0.403	1.01E-17	3 M1 _	Myo1c
Gch1	4.78E-22	0.456377	0.354	0.067	9.62E-18	3 M1 _	Gch1
Smagp1	4.66E-22	0.641181	0.537	0.139	9.39E-18	3 M1 _	Smagp
Hspg21	4.26E-22	1.1064	0.963	0.69	8.59E-18	3 M1 _	Hspg2
Chst2	3.93E-22	0.894247	0.549	0.162	7.91E-18	3 M1 _	Chst2
Chtf82	3.22E-22	0.60298	0.39	0.082	6.48E-18	3 M1 _	Chtf8
Sez6l2	2.95E-22	0.380694	0.183	0.018	5.94E-18	3 M1 _	Sez6l2
mt-Atp82	2.95E-22	1.03947	0.915	0.596	5.94E-18	3 M1 _	mt-Atp8
Nfkbia	2.82E-22	1.204716	0.866	0.531	5.68E-18	3 M1 _	Nfkbia
Sema3g	2.82E-22	0.696666	0.256	0.035	5.68E-18	3 M1 _	Sema3g

Acacb	2.23E-22	0.384636	0.256	0.035	4.50E-18	3 M1 _	Acacb
Tspan9	2.16E-22	0.948883	0.695	0.283	4.35E-18	3 M1 _	Tspan9
Trpv4	1.95E-22	0.286709	0.22	0.026	3.92E-18	3 M1 _	Trpv4
Fas	1.16E-22	0.715284	0.549	0.158	2.33E-18	3 M1 _	Fas
Dnm3	1.03E-22	0.859875	0.683	0.25	2.07E-18	3 M1 _	Dnm3
Skiv2l22	9.04E-23	0.476763	0.317	0.052	1.82E-18	3 M1 _	Skiv2l2
Lmo2	6.39E-23	0.732069	0.537	0.148	1.29E-18	3 M1 _	Lmo2
Uaca	5.87E-23	1.043719	0.707	0.262	1.18E-18	3 M1 _	Uaca
2810403A07Rik	5.26E-23	0.274478	0.22	0.025	1.06E-18	3 M1 _	2810403A07Rik
Tmcc3	3.92E-23	0.779584	0.549	0.154	7.90E-19	3 M1 _	Tmcc3
C2cd21	3.22E-23	0.652463	0.622	0.187	6.48E-19	3 M1 _	C2cd2
Ephb1	2.38E-23	0.315927	0.22	0.024	4.80E-19	3 M1 _	Ephb1
Ets1	1.56E-23	1.092875	0.951	0.597	3.14E-19	3 M1 _	Ets1
C1qtnf9	1.41E-23	0.366529	0.146	0.01	2.84E-19	3 M1 _	C1qtnf9
Tcf15	1.21E-23	0.771219	0.488	0.111	2.43E-19	3 M1 _	Tcf15
Tal1	1.09E-23	0.258428	0.195	0.019	2.19E-19	3 M1 _	Tal1
Syne21	9.88E-24	1.156968	0.854	0.406	1.99E-19	3 M1 _	Syne2
Mall	9.59E-24	0.461323	0.305	0.046	1.93E-19	3 M1 _	Mall
Klf13	8.74E-24	1.046224	0.915	0.54	1.76E-19	3 M1 _	Klf13
Ctnna1	8.48E-24	1.059239	0.951	0.662	1.71E-19	3 M1 _	Ctnna1
Dennd5b	6.96E-24	0.856211	0.646	0.213	1.40E-19	3 M1 _	Dennd5b
Rasal21	6.38E-24	0.860523	0.805	0.32	1.28E-19	3 M1 _	Rasal2
Dscr3	6.12E-24	0.28448	0.22	0.024	1.23E-19	3 M1 _	Dscr3
Ltbp41	6.08E-24	1.350535	0.927	0.663	1.22E-19	3 M1 _	Ltbp4
Plk2	5.80E-24	0.813711	0.512	0.126	1.17E-19	3 M1 _	Plk2
Efnb1	4.59E-24	1.079237	0.659	0.226	9.25E-20	3 M1 _	Efnb1
Sema6a	4.59E-24	0.873184	0.561	0.153	9.24E-20	3 M1 _	Sema6a
Ly6e	3.55E-24	1.012602	1	0.874	7.15E-20	3 M1 _	Ly6e
Gm26669	3.39E-24	0.281495	0.146	0.01	6.83E-20	3 M1 _	Gm26669
Slc43a31	3.21E-24	1.301482	0.902	0.495	6.47E-20	3 M1 _	Slc43a3
Tmem47	2.76E-24	0.98108	0.78	0.321	5.56E-20	3 M1 _	Tmem47
Papd42	2.61E-24	0.333929	0.268	0.035	5.26E-20	3 M1 _	Papd4
Dopey12	2.48E-24	0.44969	0.305	0.046	5.00E-20	3 M1 _	Dopey1
Tnfaip11	2.43E-24	0.990492	0.817	0.366	4.89E-20	3 M1 _	Tnfaip1
Mfhas1	2.40E-24	0.844209	0.61	0.189	4.83E-20	3 M1 _	Mfhas1
Sox131	2.30E-24	0.560007	0.415	0.081	4.63E-20	3 M1 _	Sox13
Slfn51	2.30E-24	1.219896	0.939	0.709	4.62E-20	3 M1 _	Slfn5
Rapgef21	2.18E-24	1.045395	0.646	0.214	4.39E-20	3 M1 _	Rapgef2
Helz2	1.94E-24	0.652071	0.512	0.128	3.91E-20	3 M1 _	Helz2
Klhl5	1.45E-24	0.936907	0.659	0.227	2.92E-20	3 M1 _	Klhl5
Actn41	1.43E-24	1.125904	0.939	0.712	2.88E-20	3 M1 _	Actn4

Foxp1	1.36E-24	1.171668	0.902	0.544	2.73E-20	3 M1 _	Foxp1
Prdm16	1.12E-24	0.550007	0.305	0.045	2.26E-20	3 M1 _	Prdm16
Ddah1	8.55E-25	0.434061	0.232	0.026	1.72E-20	3 M1 _	Ddah1
Ackr3	5.68E-25	0.654032	0.5	0.113	1.14E-20	3 M1 _	Ackr3
Ssu2	5.10E-25	0.40663	0.28	0.037	1.03E-20	3 M1 _	Ssu2
Dopey21	4.13E-25	0.320562	0.195	0.018	8.31E-21	3 M1 _	Dopey2
Efhd1	3.62E-25	0.716073	0.268	0.035	7.29E-21	3 M1 _	Efhd1
Aplp2	3.35E-25	1.155317	0.939	0.696	6.74E-21	3 M1 _	Aplp2
Cdc42bpb	2.99E-25	0.892887	0.756	0.296	6.01E-21	3 M1 _	Cdc42bpb
Filip11	2.86E-25	0.734914	0.634	0.179	5.76E-21	3 M1 _	Filip1
Alpl	2.57E-25	0.763537	0.378	0.067	5.17E-21	3 M1 _	Alpl
Stard81	2.24E-25	0.871651	0.671	0.199	4.52E-21	3 M1 _	Stard8
Trib1	1.93E-25	0.696375	0.5	0.117	3.89E-21	3 M1 _	Trib1
Dgke	1.83E-25	0.388554	0.329	0.05	3.68E-21	3 M1 _	Dgke
Pcdh11	1.81E-25	0.732183	0.61	0.165	3.65E-21	3 M1 _	Pcdh1
Als2cl	1.59E-25	0.495383	0.329	0.051	3.20E-21	3 M1 _	Als2cl
Id3	1.57E-25	1.415778	0.963	0.754	3.15E-21	3 M1 _	Id3
Rps6ka2	1.47E-25	0.466379	0.305	0.044	2.97E-21	3 M1 _	Rps6ka2
Eif4g21	1.38E-25	1.069507	0.988	0.772	2.78E-21	3 M1 _	Eif4g2
Tox3	1.26E-25	0.333185	0.207	0.02	2.55E-21	3 M1 _	Tox3
Plscr1	1.19E-25	0.917643	0.61	0.185	2.40E-21	3 M1 _	Plscr1
Atp2b41	6.69E-26	0.994861	0.793	0.296	1.35E-21	3 M1 _	Atp2b4
1500011K16Rik2	5.02E-26	0.347447	0.305	0.041	1.01E-21	3 M1 _	1500011K16Rik
Pyurf3	4.77E-26	0.437305	0.293	0.039	9.60E-22	3 M1 _	Pyurf
Fat4	3.71E-26	0.57191	0.451	0.092	7.47E-22	3 M1 _	Fat4
Phlpp1	3.46E-26	0.739937	0.585	0.157	6.98E-22	3 M1 _	Phlpp1
Fry1	2.83E-26	0.717433	0.659	0.175	5.70E-22	3 M1 _	Fry
Aaed12	2.82E-26	0.517229	0.378	0.063	5.69E-22	3 M1 _	Aaed1
Smad6	2.77E-26	0.530184	0.305	0.043	5.59E-22	3 M1 _	Smad6
Hip1	2.62E-26	1.055013	0.805	0.34	5.28E-22	3 M1 _	Hip1
Hyal2	2.19E-26	0.790919	0.5	0.118	4.41E-22	3 M1 _	Hyal2
AI3141802	2.12E-26	0.502087	0.378	0.063	4.28E-22	3 M1 _	AI314180
Ahr	2.10E-26	0.968304	0.646	0.187	4.24E-22	3 M1 _	Ahr
Shroom4	1.85E-26	0.477703	0.329	0.048	3.73E-22	3 M1 _	Shroom4
Fam58b2	1.82E-26	0.403551	0.268	0.032	3.67E-22	3 M1 _	Fam58b
4931406P16Rik1	1.61E-26	1.206082	0.866	0.432	3.23E-22	3 M1 _	4931406P16Rik
Fam43a	1.02E-26	0.829237	0.61	0.163	2.05E-22	3 M1 _	Fam43a
Clec2d	7.49E-27	1.479963	0.927	0.641	1.51E-22	3 M1 _	Clec2d
Fut8	5.81E-27	0.990712	0.793	0.314	1.17E-22	3 M1 _	Fut8
Ppp1r13b	2.51E-27	0.583017	0.524	0.118	5.05E-23	3 M1 _	Ppp1r13b
6430548M08Rik	1.49E-27	0.659749	0.427	0.082	3.00E-23	3 M1 _	6430548M08Rik

Spns2	1.29E-27	0.501131	0.329	0.047	2.60E-23	3 M1 _	Spns2
Cd341	1.20E-27	1.186386	0.927	0.432	2.41E-23	3 M1 _	Cd34
Cd38	1.13E-27	0.88004	0.695	0.217	2.27E-23	3 M1 _	Cd38
Sdccag32	6.14E-28	0.377105	0.305	0.039	1.24E-23	3 M1 _	Sdccag3
Acvrl1	5.66E-28	1.249699	0.841	0.391	1.14E-23	3 M1 _	Acvrl1
Fez21	5.56E-28	1.147296	0.854	0.363	1.12E-23	3 M1 _	Fez2
Kif26a	5.09E-28	0.628421	0.341	0.049	1.02E-23	3 M1 _	Kif26a
Sptan1	3.56E-28	1.307695	0.951	0.658	7.18E-24	3 M1 _	Sptan1
Clstn11	3.21E-28	1.159639	0.927	0.557	6.46E-24	3 M1 _	Clstn1
Arhgap29	1.70E-28	1.317556	0.927	0.611	3.43E-24	3 M1 _	Arhgap29
Zbtb162	1.42E-28	0.648595	0.463	0.087	2.86E-24	3 M1 _	Zbtb16
Arhgef3	1.41E-28	0.650524	0.488	0.1	2.84E-24	3 M1 _	Arhgef3
Endou	1.04E-28	0.437091	0.293	0.035	2.09E-24	3 M1 _	Endou
Lnx2	9.04E-29	0.632127	0.402	0.069	1.82E-24	3 M1 _	Lnx2
Tmcc2	7.13E-29	0.571648	0.402	0.068	1.44E-24	3 M1 _	Tmcc2
BC0033312	5.67E-29	0.534893	0.378	0.057	1.14E-24	3 M1 _	BC003331
Ptprm	5.59E-29	0.789057	0.598	0.153	1.12E-24	3 M1 _	Ptprm
Gpr4	5.40E-29	0.443592	0.317	0.041	1.09E-24	3 M1 _	Gpr4
Sox17	4.93E-29	0.838197	0.451	0.082	9.93E-25	3 M1 _	Sox17
Rhbdl2	2.84E-29	0.456662	0.256	0.026	5.72E-25	3 M1 _	Rhbdl2
Cited21	2.70E-29	1.09664	0.78	0.269	5.44E-25	3 M1 _	Cited2
Mgea53	2.41E-29	0.560757	0.341	0.048	4.85E-25	3 M1 _	Mgea5
Dusp3	2.13E-29	1.408965	0.817	0.345	4.29E-25	3 M1 _	Dusp3
Ncoa7	1.43E-29	1.517954	0.829	0.355	2.88E-25	3 M1 _	Ncoa7
Msn1	1.42E-29	1.286183	0.963	0.791	2.86E-25	3 M1 _	Msn
Gm32688	1.31E-29	0.442179	0.28	0.031	2.63E-25	3 M1 _	Gm32688
Fam103a13	1.29E-29	0.476835	0.378	0.056	2.60E-25	3 M1 _	Fam103a1
Ptgs1	1.26E-29	1.261661	0.732	0.26	2.53E-25	3 M1 _	Ptgs1
2810474O19Rik3	8.40E-30	0.860498	0.415	0.068	1.69E-25	3 M1 _	2810474O19Rik
Adam15	7.10E-30	1.235531	0.805	0.343	1.43E-25	3 M1 _	Adam15
Gnai2	5.24E-30	1.075432	0.976	0.923	1.06E-25	3 M1 _	Gnai2
Ephb4	5.01E-30	0.863564	0.585	0.144	1.01E-25	3 M1 _	Ephb4
Deup1	5.00E-30	0.251651	0.146	0.007	1.01E-25	3 M1 _	Deup1
St6galnac3	3.57E-30	0.513109	0.341	0.046	7.19E-26	3 M1 _	St6galnac3
Slco2a1	3.09E-30	0.950214	0.573	0.138	6.23E-26	3 M1 _	Slco2a1
Zfp979	1.77E-30	0.719215	0.378	0.056	3.57E-26	3 M1 _	Zfp979
Jup	1.38E-30	1.538205	0.829	0.379	2.78E-26	3 M1 _	Jup
St3gal5	1.02E-30	0.522831	0.341	0.046	2.05E-26	3 M1 _	St3gal5
Chml	7.34E-31	0.536967	0.366	0.052	1.48E-26	3 M1 _	Chml
Ripply3	6.08E-31	0.782257	0.512	0.105	1.22E-26	3 M1 _	Ripply3
0610009O20Rik2	5.01E-31	0.409051	0.28	0.03	1.01E-26	3 M1 _	0610009O20Rik

Hmbox1	4.39E-31	1.10648	0.768	0.256	8.84E-27	3 M1 _	Hmbox1
Bmpr2	3.61E-31	1.440104	0.939	0.638	7.27E-27	3 M1 _	Bmpr2
Ccdc28b	3.40E-31	1.258046	0.829	0.326	6.84E-27	3 M1 _	Ccdc28b
Vamp5	3.15E-31	1.327735	0.927	0.457	6.35E-27	3 M1 _	Vamp5
Itpkb	2.77E-31	1.316526	0.878	0.387	5.59E-27	3 M1 _	Itpkb
0610037L13Rik3	2.64E-31	0.484376	0.341	0.044	5.32E-27	3 M1 _	0610037L13Rik
Gpr146	1.67E-31	0.713808	0.5	0.097	3.36E-27	3 M1 _	Gpr146
Apold11	1.13E-31	0.536738	0.439	0.069	2.28E-27	3 M1 _	Apold1
Ndrg1	1.03E-31	1.213579	0.817	0.299	2.07E-27	3 M1 _	Ndrg1
Ctla2a	7.22E-32	1.366884	0.927	0.336	1.45E-27	3 M1 _	Ctla2a
Gm9917	6.25E-32	0.338481	0.232	0.019	1.26E-27	3 M1 _	Gm9917
Pitpnm2	4.42E-32	0.805369	0.573	0.125	8.89E-28	3 M1 _	Pitpnm2
Ramp21	4.07E-32	1.435999	0.939	0.482	8.19E-28	3 M1 _	Ramp2
Zcchc113	4.03E-32	0.489535	0.415	0.062	8.12E-28	3 M1 _	Zcchc11
Akr1c14	3.20E-32	0.671321	0.415	0.067	6.44E-28	3 M1 _	Akr1c14
Nat6	2.58E-32	0.261776	0.232	0.019	5.20E-28	3 M1 _	Nat6
Meox1	1.50E-32	1.122058	0.744	0.224	3.02E-28	3 M1 _	Meox1
Myo18a	7.64E-33	1.42364	0.744	0.251	1.54E-28	3 M1 _	Myo18a
Ly6a	2.48E-33	1.671361	1	0.724	5.00E-29	3 M1 _	Ly6a
Slc16a1	2.21E-33	0.839395	0.341	0.043	4.46E-29	3 M1 _	Slc16a1
Rapgef5	1.57E-33	0.931144	0.61	0.127	3.16E-29	3 M1 _	Rapgef5
Dlc11	1.38E-33	1.551995	0.976	0.696	2.77E-29	3 M1 _	Dlc1
Pla2g162	9.15E-34	0.44895	0.317	0.035	1.84E-29	3 M1 _	Pla2g16
Cdh13	8.89E-34	0.801918	0.415	0.062	1.79E-29	3 M1 _	Cdh13
Tmem54	7.74E-34	0.661799	0.463	0.075	1.56E-29	3 M1 _	Tmem5
AU0198233	7.60E-34	0.459947	0.305	0.032	1.53E-29	3 M1 _	AU019823
Cracr2b	6.81E-34	0.770579	0.585	0.121	1.37E-29	3 M1 _	Cracr2b
Heg1	7.07E-35	1.585225	0.829	0.322	1.42E-30	3 M1 _	Heg1
Npr1	5.86E-35	0.846915	0.549	0.109	1.18E-30	3 M1 _	Npr1
Unc45b	5.17E-35	0.926092	0.537	0.103	1.04E-30	3 M1 _	Unc45b
Rassf3	4.98E-35	0.890893	0.573	0.115	1.00E-30	3 M1 _	Rassf3
Scarb1	4.18E-35	0.840494	0.72	0.165	8.41E-31	3 M1 _	Scarb1
Nxpe4	3.63E-35	0.723319	0.378	0.05	7.30E-31	3 M1 _	Nxpe4
Snrk1	3.17E-35	1.582523	0.988	0.596	6.38E-31	3 M1 _	Snrk
Timp31	2.81E-35	1.689011	1	0.784	5.66E-31	3 M1 _	Timp3
Mpped2	2.54E-35	0.436693	0.244	0.019	5.11E-31	3 M1 _	Mpped2
Slc27a3	2.51E-35	0.706065	0.378	0.05	5.06E-31	3 M1 _	Slc27a3
Mfsd6	1.03E-35	0.907668	0.537	0.101	2.08E-31	3 M1 _	Mfsd6
Hoxd8	9.32E-36	0.615057	0.427	0.062	1.88E-31	3 M1 _	Hoxd8
Igfbp3	4.53E-36	1.323391	0.829	0.252	9.13E-32	3 M1 _	Igfbp3
Abi3	4.40E-36	1.056553	0.707	0.177	8.86E-32	3 M1 _	Abi3

Notch4	4.29E-36	0.983864	0.622	0.135	8.64E-32	3 M1 _	Notch4
Calcrl	2.94E-36	1.441992	0.817	0.298	5.93E-32	3 M1 _	Calcrl
Prkch	2.74E-36	0.799869	0.707	0.164	5.53E-32	3 M1 _	Prkch
Pkp4	2.30E-36	1.026964	0.634	0.142	4.63E-32	3 M1 _	Pkp4
Epas11	1.96E-36	1.796681	0.951	0.515	3.95E-32	3 M1 _	Epas1
Acss1	1.60E-36	0.435032	0.293	0.027	3.23E-32	3 M1 _	Acss1
Nos3	1.45E-36	0.640479	0.378	0.047	2.91E-32	3 M1 _	Nos3
1110004E09Rik1	1.13E-36	0.442066	0.293	0.027	2.28E-32	3 M1 _	1110004E09Rik
Prickle1	1.09E-36	0.82389	0.549	0.106	2.20E-32	3 M1 _	Prickle1
Nrp1	1.02E-36	1.829335	0.976	0.794	2.06E-32	3 M1 _	Nrp1
1110008F13Rik3	9.92E-37	0.760635	0.5	0.082	2.00E-32	3 M1 _	1110008F13Rik
Ttll71	9.57E-37	0.970434	0.5	0.083	1.93E-32	3 M1 _	Ttll7
Klf2	8.34E-37	2.083978	0.927	0.45	1.68E-32	3 M1 _	Klf2
Nfib1	4.65E-37	1.629491	1	0.753	9.37E-33	3 M1 _	Nfib
Dennd3	3.35E-37	0.559623	0.415	0.055	6.74E-33	3 M1 _	Dennd3
Tm4sf11	2.40E-37	1.906983	0.988	0.685	4.83E-33	3 M1 _	Tm4sf1
Jam2	2.38E-37	1.430941	0.866	0.319	4.79E-33	3 M1 _	Jam2
Hspb11	2.16E-37	1.832418	0.988	0.59	4.36E-33	3 M1 _	Hspb1
Tspan121	1.80E-37	0.991399	0.659	0.139	3.62E-33	3 M1 _	Tspan12
Sorbs21	1.78E-37	0.974153	0.744	0.174	3.59E-33	3 M1 _	Sorbs2
Entpd1	1.15E-37	1.271077	0.732	0.185	2.32E-33	3 M1 _	Entpd1
Clic4	6.88E-38	1.69877	0.951	0.572	1.38E-33	3 M1 _	Clic4
Arap3	5.72E-38	1.066177	0.707	0.169	1.15E-33	3 M1 _	Arap3
Sybu	5.24E-38	0.598482	0.354	0.039	1.06E-33	3 M1 _	Sybu
Ppp1r16b	4.79E-38	0.797036	0.549	0.098	9.65E-34	3 M1 _	Ppp1r16b
Calm11	3.95E-38	1.496157	0.988	0.95	7.95E-34	3 M1 _	Calm1
Fam208b2	3.30E-38	0.549234	0.305	0.028	6.65E-34	3 M1 _	Fam208b
Anxa31	2.37E-38	1.34183	0.878	0.296	4.77E-34	3 M1 _	Anxa3
Fli1	2.32E-38	1.078762	0.805	0.223	4.68E-34	3 M1 _	Fli1
Lipe	2.11E-38	0.996133	0.549	0.1	4.25E-34	3 M1 _	Lipe
Ccm2l	1.35E-38	0.72556	0.451	0.064	2.72E-34	3 M1 _	Ccm2l
Rapgef3	4.59E-39	1.082126	0.634	0.135	9.23E-35	3 M1 _	Rapgef3
Scube3	2.28E-39	0.341193	0.207	0.011	4.59E-35	3 M1 _	Scube3
Ankrd29	2.15E-39	0.684539	0.39	0.046	4.33E-35	3 M1 _	Ankrd29
Tmem255b	2.11E-39	0.644398	0.427	0.055	4.24E-35	3 M1 _	Tmem255b
Arhgap27	8.52E-40	0.849561	0.549	0.094	1.72E-35	3 M1 _	Arhgap27
Plxna2	8.03E-40	1.192037	0.634	0.136	1.62E-35	3 M1 _	Plxna2
Mef2c1	7.22E-40	1.992195	0.988	0.64	1.45E-35	3 M1 _	Mef2c
Ptprg	5.26E-40	1.539824	0.927	0.405	1.06E-35	3 M1 _	Ptprg
Abcg2	2.08E-40	1.674733	0.89	0.33	4.19E-36	3 M1 _	Abcg2
Sptbn1	1.75E-40	1.684171	1	0.898	3.52E-36	3 M1 _	Sptbn1

Plpp31	1.47E-40	2.411758	0.988	0.722	2.96E-36	3 M1 _	Plpp3
Ece1	9.89E-41	1.667696	0.902	0.349	1.99E-36	3 M1 _	Ece1
Afap1l1	6.48E-41	0.83681	0.488	0.071	1.30E-36	3 M1 _	Afap1l1
Cxcl12	6.38E-41	3.080739	0.988	0.787	1.28E-36	3 M1 _	Cxcl12
Kitl1	3.71E-41	1.885658	0.915	0.391	7.47E-37	3 M1 _	Kitl
Rapgef4	2.55E-41	0.973223	0.537	0.087	5.13E-37	3 M1 _	Rapgef4
Esam1	2.11E-41	1.447976	0.963	0.314	4.25E-37	3 M1 _	Esam
Tmem572	1.58E-41	0.538911	0.341	0.033	3.17E-37	3 M1 _	Tmem57
Jag2	1.54E-41	0.870555	0.488	0.069	3.10E-37	3 M1 _	Jag2
Pik3r3	1.45E-41	0.709653	0.5	0.074	2.93E-37	3 M1 _	Pik3r3
Sipa1l2	1.42E-41	1.008771	0.524	0.085	2.87E-37	3 M1 _	Sipa1l2
Cav21	1.21E-41	1.162373	0.768	0.188	2.44E-37	3 M1 _	Cav2
Syne1	8.32E-42	1.774331	0.89	0.322	1.68E-37	3 M1 _	Syne1
Eng	7.65E-42	1.778519	1	0.662	1.54E-37	3 M1 _	Eng
Sdk2	5.34E-42	0.591878	0.354	0.035	1.08E-37	3 M1 _	Sdk2
Fam96a1	5.22E-42	0.456491	0.354	0.035	1.05E-37	3 M1 _	Fam96a
Gnas2	1.68E-42	1.358998	1	0.96	3.38E-38	3 M1 _	Gnas
Tmtc1	1.52E-42	0.725259	0.524	0.079	3.07E-38	3 M1 _	Tmtc1
Gpr150	1.36E-42	0.307836	0.183	0.007	2.73E-38	3 M1 _	Gpr150
Plpp1	6.00E-43	2.791234	0.951	0.588	1.21E-38	3 M1 _	Plpp1
Bcam1	4.36E-43	1.520976	0.951	0.309	8.79E-39	3 M1 _	Bcam
Etl4	3.38E-43	1.212575	0.707	0.163	6.81E-39	3 M1 _	Etl4
Tspan13	1.40E-43	1.384688	0.744	0.172	2.81E-39	3 M1 _	Tspan13
Gipc3	9.64E-44	0.465756	0.305	0.024	1.94E-39	3 M1 _	Gipc3
Exoc3l	9.39E-44	0.553001	0.317	0.026	1.89E-39	3 M1 _	Exoc3l
Ccnd11	8.84E-44	1.469225	0.732	0.157	1.78E-39	3 M1 _	Ccnd1
Pcp4l11	4.19E-44	1.492326	0.829	0.205	8.43E-40	3 M1 _	Pcp4l1
Bvht	4.18E-44	1.214955	0.695	0.154	8.42E-40	3 M1 _	Bvht
Efr3b	1.35E-44	0.618827	0.415	0.046	2.72E-40	3 M1 _	Efr3b
Minos13	1.15E-44	0.974521	0.646	0.11	2.33E-40	3 M1 _	Minos1
9030624J02Rik2	8.81E-45	0.475578	0.366	0.035	1.78E-40	3 M1 _	9030624J02Rik
Fzd41	8.21E-45	1.648944	0.939	0.327	1.65E-40	3 M1 _	Fzd4
Efnb2	5.10E-45	1.044343	0.646	0.117	1.03E-40	3 M1 _	Efnb2
Papd51	4.96E-45	0.497889	0.293	0.021	9.99E-41	3 M1 _	Papd5
Klf4	4.44E-45	1.843763	0.902	0.295	8.95E-41	3 M1 _	Klf4
Fabp51	4.21E-45	2.321958	0.805	0.233	8.49E-41	3 M1 _	Fabp5
Tinagl11	3.04E-45	1.671647	0.963	0.286	6.11E-41	3 M1 _	Tinagl1
Cyr612	1.33E-45	0.558793	0.329	0.027	2.69E-41	3 M1 _	Cyr61
Sgpp2	1.19E-45	0.274905	0.183	0.006	2.39E-41	3 M1 _	Sgpp2
Enpp6	1.05E-45	0.445181	0.22	0.01	2.12E-41	3 M1 _	Enpp6
Lrrc3b	7.96E-46	0.389059	0.268	0.016	1.60E-41	3 M1 _	Lrrc3b

Sema3c	4.34E-46	1.277553	0.61	0.104	8.75E-42	3 M1 _	Sema3c
Tdrp	4.29E-46	0.74852	0.439	0.051	8.64E-42	3 M1 _	Tdrp
1810022K09Rik3	3.39E-46	0.79729	0.585	0.087	6.82E-42	3 M1 _	1810022K09Rik
Mum13	2.92E-46	0.706049	0.427	0.046	5.89E-42	3 M1 _	Mum1
Cd361	2.28E-46	2.066302	0.793	0.183	4.60E-42	3 M1 _	Cd36
Srgn1	9.13E-47	2.127405	0.976	0.401	1.84E-42	3 M1 _	Srgn
Nr5a2	7.69E-47	0.71058	0.256	0.015	1.55E-42	3 M1 _	Nr5a2
Sema3f	4.78E-47	0.938953	0.561	0.084	9.63E-43	3 M1 _	Sema3f
Dok4	3.15E-47	1.009302	0.61	0.1	6.35E-43	3 M1 _	Dok4
Plekho1	2.79E-47	1.119263	0.744	0.15	5.63E-43	3 M1 _	Plekho1
Egfl7	2.30E-47	2.200471	0.976	0.373	4.64E-43	3 M1 _	Egfl7
Mmp15	1.61E-47	0.890948	0.524	0.071	3.25E-43	3 M1 _	Mmp15
C130074G19Rik	1.23E-47	1.198087	0.585	0.091	2.48E-43	3 M1 _	C130074G19Rik
Zfp366	1.11E-47	0.717631	0.463	0.054	2.24E-43	3 M1 _	Zfp366
Thbd1	9.56E-48	2.317095	0.963	0.397	1.92E-43	3 M1 _	Thbd
Impdh1	4.29E-48	1.152306	0.732	0.153	8.63E-44	3 M1 _	Impdh1
Fgd5	4.01E-48	1.310431	0.756	0.17	8.08E-44	3 M1 _	Fgd5
Nostrin	2.99E-48	1.509548	0.78	0.171	6.02E-44	3 M1 _	Nostrin
Enah	2.42E-48	0.962342	0.549	0.078	4.87E-44	3 M1 _	Enah
Rasgrf2	1.30E-48	0.652145	0.451	0.049	2.62E-44	3 M1 _	Rasgrf2
Gpihbp1	1.15E-48	1.958321	0.829	0.204	2.32E-44	3 M1 _	Gpihbp1
Rnf1521	1.00E-48	0.858908	0.524	0.068	2.02E-44	3 M1 _	Rnf152
She	9.62E-49	1.007315	0.646	0.108	1.94E-44	3 M1 _	She
Rdx	7.59E-49	3.013101	0.988	0.796	1.53E-44	3 M1 _	Rdx
Hlx	5.70E-49	1.004945	0.561	0.08	1.15E-44	3 M1 _	Hlx
Flt4	4.51E-49	0.693187	0.512	0.064	9.08E-45	3 M1 _	Flt4
Plvap	3.54E-49	2.893919	1	0.51	7.12E-45	3 M1 _	Plvap
Lrrc8c1	3.42E-49	1.438191	0.866	0.227	6.88E-45	3 M1 _	Lrrc8c
Shank3	3.04E-49	0.819392	0.439	0.047	6.13E-45	3 M1 _	Shank3
Rmst	1.90E-49	0.53242	0.268	0.015	3.83E-45	3 M1 _	Rmst
Rtp3	1.35E-49	0.683465	0.402	0.038	2.72E-45	3 M1 _	Rtp3
Tnfsf10	1.16E-49	1.578094	0.841	0.208	2.33E-45	3 M1 _	Tnfsf10
Slc26a10	7.16E-50	0.733159	0.39	0.035	1.44E-45	3 M1 _	Slc26a10
Kctd12b	2.48E-50	1.86657	0.915	0.307	4.99E-46	3 M1 _	Kctd12b
Gcn1l13	2.25E-50	0.587202	0.39	0.035	4.53E-46	3 M1 _	Gcn1l1
Cav11	1.15E-50	1.899306	0.939	0.289	2.31E-46	3 M1 _	Cav1
Ecscr	9.42E-51	1.338428	0.878	0.211	1.90E-46	3 M1 _	Ecscr
2010107E04Rik3	4.75E-51	0.956862	0.695	0.11	9.57E-47	3 M1 _	2010107E04Rik
Arhgap31	3.81E-51	2.001532	0.951	0.341	7.67E-47	3 M1 _	Arhgap31
Clec14a	1.51E-51	2.056745	0.976	0.362	3.04E-47	3 M1 _	Clec14a
Pim3	1.68E-52	1.369691	0.744	0.149	3.38E-48	3 M1 _	Pim3

Gatsl3	1.20E-52	0.451732	0.305	0.019	2.41E-48	3 M1 _	Gatsl3
Slc9a3r2	3.79E-53	2.634004	0.988	0.453	7.63E-49	3 M1 _	Slc9a3r2
Grrp1	2.26E-53	1.221655	0.695	0.11	4.56E-49	3 M1 _	Grrp1
Ly6c1	5.14E-54	2.721514	1	0.406	1.04E-49	3 M1 _	Ly6c1
Oraov11	2.25E-54	0.54501	0.329	0.021	4.54E-50	3 M1 _	Oraov1
Pkn3	1.53E-54	0.602727	0.415	0.036	3.09E-50	3 M1 _	Pkn3
Crim11	1.30E-54	2.211794	0.915	0.275	2.61E-50	3 M1 _	Crim1
Sema7a	7.66E-55	1.703875	0.866	0.217	1.54E-50	3 M1 _	Sema7a
2010111I01Rik2	5.70E-55	0.93735	0.537	0.063	1.15E-50	3 M1 _	2010111I01Rik
Adcy4	2.15E-55	1.081266	0.768	0.139	4.33E-51	3 M1 _	Adcy4
Sox18	1.83E-55	0.858033	0.573	0.071	3.70E-51	3 M1 _	Sox18
Chrm3	1.28E-55	0.458057	0.244	0.01	2.58E-51	3 M1 _	Chrm3
Gata2	9.74E-56	0.906698	0.512	0.058	1.96E-51	3 M1 _	Gata2
Pltp	8.81E-56	2.232135	0.951	0.304	1.77E-51	3 M1 _	Pltp
Ehd4	6.03E-56	2.711368	0.951	0.331	1.21E-51	3 M1 _	Ehd4
Fam60a1	4.66E-56	0.355969	0.22	0.007	9.38E-52	3 M1 _	Fam60a
2410015M20Rik3	1.36E-56	0.891132	0.683	0.098	2.74E-52	3 M1 _	2410015M20Rik
B4galt4	1.32E-56	1.351613	0.744	0.138	2.66E-52	3 M1 _	B4galt4
D17Wsu92e3	9.81E-57	0.734992	0.476	0.047	1.98E-52	3 M1 _	D17Wsu92e
Plscr2	6.37E-57	2.63546	0.976	0.362	1.28E-52	3 M1 _	Plscr2
Tcim	4.61E-57	2.153882	0.927	0.261	9.29E-53	3 M1 _	Tcim
Ushbp1	4.61E-57	1.437574	0.78	0.141	9.28E-53	3 M1 _	Ushbp1
Gngt2	4.59E-57	1.757485	0.902	0.239	9.24E-53	3 M1 _	Gngt2
Usmg53	1.98E-57	1.269469	0.732	0.112	3.98E-53	3 M1 _	Usmg5
Tek	1.85E-57	1.397099	0.793	0.143	3.72E-53	3 M1 _	Tek
Cdkn1a1	7.18E-58	1.687151	0.817	0.166	1.45E-53	3 M1 _	Cdkn1a
Caskin2	6.08E-58	1.292735	0.744	0.131	1.22E-53	3 M1 _	Caskin2
Atp5o.13	5.11E-58	1.037798	0.707	0.105	1.03E-53	3 M1 _	Atp5o.1
Fabp4	3.87E-58	3.876567	1	0.435	7.79E-54	3 M1 _	Fabp4
Samd12	2.01E-58	0.762818	0.524	0.055	4.06E-54	3 M1 _	Samd12
Rflnb	1.49E-58	2.734064	0.951	0.3	3.00E-54	3 M1 _	Rflnb
Fam96b3	1.22E-58	0.84038	0.524	0.056	2.45E-54	3 M1 _	Fam96b
Nova2	1.04E-58	0.795117	0.524	0.056	2.10E-54	3 M1 _	Nova2
Lhx6	6.52E-59	0.760791	0.317	0.018	1.31E-54	3 M1 _	Lhx6
Smco4	2.39E-59	2.03169	0.915	0.242	4.82E-55	3 M1 _	Smco4
Slc6a6	8.43E-60	3.102767	0.963	0.343	1.70E-55	3 M1 _	Slc6a6
Chst15	4.85E-60	1.276507	0.744	0.124	9.76E-56	3 M1 _	Chst15
F11r	9.77E-61	1.205651	0.756	0.126	1.97E-56	3 M1 _	F11r
Ptprr	6.95E-61	0.946184	0.415	0.032	1.40E-56	3 M1 _	Ptprr
Mcc	3.14E-61	1.656111	0.78	0.152	6.32E-57	3 M1 _	Mcc
Tbxa2r	2.76E-61	0.776277	0.512	0.051	5.57E-57	3 M1 _	Tbxa2r

Hecw2	1.01E-61	1.582948	0.829	0.165	2.03E-57	3 M1 _	Hecw2
Trove22	7.18E-62	1.049805	0.585	0.067	1.45E-57	3 M1 _	Trove2
Olfr1396	3.15E-62	0.808434	0.341	0.02	6.35E-58	3 M1 _	Olfr1396
Cavin2	3.78E-63	2.294266	0.988	0.265	7.61E-59	3 M1 _	Cavin2
Car8	2.88E-63	0.893013	0.524	0.051	5.81E-59	3 M1 _	Car8
Rsad2	2.42E-63	2.632818	0.89	0.199	4.87E-59	3 M1 _	Rsad2
Lama3	1.13E-63	0.952465	0.561	0.06	2.27E-59	3 M1 _	Lama3
Pecam1	8.60E-64	2.357342	0.988	0.257	1.73E-59	3 M1 _	Pecam1
Sgk1	5.44E-64	2.773484	0.939	0.269	1.10E-59	3 M1 _	Sgk1
Fam208a3	5.23E-64	1.0833	0.598	0.068	1.05E-59	3 M1 _	Fam208a
Icam2	3.80E-64	1.349581	0.829	0.139	7.65E-60	3 M1 _	Icam2
Syt15	2.31E-64	0.740485	0.439	0.034	4.64E-60	3 M1 _	Syt15
Cd200	7.82E-65	2.240684	0.976	0.249	1.57E-60	3 M1 _	Cd200
Itga6	3.66E-65	1.704248	0.854	0.156	7.36E-61	3 M1 _	Itga6
Aes3	1.32E-66	1.456532	0.78	0.115	2.67E-62	3 M1 _	Aes
Emcn	6.03E-67	2.884653	0.963	0.256	1.22E-62	3 M1 _	Emcn
Ptprb	4.15E-67	2.688812	0.988	0.26	8.35E-63	3 M1 _	Ptprb
Id1	1.40E-67	2.710077	0.963	0.263	2.83E-63	3 M1 _	Id1
Dysf	7.18E-68	1.66509	0.756	0.118	1.45E-63	3 M1 _	Dysf
Grin2a	4.69E-68	0.345451	0.183	0.002	9.44E-64	3 M1 _	Grin2a
Kank3	1.26E-68	1.43511	0.829	0.137	2.54E-64	3 M1 _	Kank3
Clec1a	9.29E-69	1.918281	0.829	0.15	1.87E-64	3 M1 _	Clec1a
Adgrf5	7.10E-69	2.289982	0.951	0.214	1.43E-64	3 M1 _	Adgrf5
Palmd	7.98E-70	1.452152	0.72	0.096	1.61E-65	3 M1 _	Palmd
Vwa3a	7.79E-70	0.544149	0.305	0.012	1.57E-65	3 M1 _	Vwa3a
Ccdc85a	3.70E-71	0.965042	0.561	0.053	7.45E-67	3 M1 _	Ccdc85a
Fam212a1	3.37E-71	0.716245	0.39	0.022	6.78E-67	3 M1 _	Fam212a
Pcdh17	1.48E-71	1.783992	0.805	0.12	2.97E-67	3 M1 _	Pcdh17
Plcb1	8.93E-72	1.645986	0.829	0.133	1.80E-67	3 M1 _	Plcb1
Ceacam1	5.87E-72	1.284626	0.707	0.086	1.18E-67	3 M1 _	Ceacam1
Mmrn2	4.94E-72	1.602746	0.89	0.149	9.95E-68	3 M1 _	Mmrn2
Rasip1	7.50E-73	1.393653	0.841	0.127	1.51E-68	3 M1 _	Rasip1
Myct1	4.41E-73	1.338292	0.817	0.117	8.87E-69	3 M1 _	Myct1
Robo4	4.16E-73	1.216443	0.683	0.081	8.38E-69	3 M1 _	Robo4
Mgll	2.07E-73	3.167218	0.976	0.254	4.17E-69	3 M1 _	Mgll
Flt1	9.10E-74	3.47485	1	0.267	1.83E-69	3 M1 _	Flt1
Gm12002	2.54E-74	1.135431	0.561	0.05	5.11E-70	3 M1 _	Gm12002
Alox12	3.35E-75	1.089989	0.561	0.048	6.75E-71	3 M1 _	Alox12
Acer2	5.42E-76	0.954235	0.5	0.037	1.09E-71	3 M1 _	Acer2
Parvb	3.41E-77	1.076357	0.61	0.059	6.86E-73	3 M1 _	Parvb
Lims2	1.86E-77	1.452784	0.866	0.13	3.74E-73	3 M1 _	Lims2

S1pr1	1.41E-77	2.291023	0.915	0.17	2.84E-73	3 M1 _	S1pr1
Rgcc	7.35E-78	3.662971	0.988	0.243	1.48E-73	3 M1 _	Rgcc
Adgrl4	2.34E-78	2.161645	0.976	0.18	4.71E-74	3 M1 _	Adgrl4
Proser2	2.12E-78	1.019509	0.549	0.045	4.27E-74	3 M1 _	Proser2
Col13a1	5.32E-80	2.103377	0.902	0.154	1.07E-75	3 M1 _	Col13a1
4930578C19Rik	4.29E-80	0.31933	0.159	0	8.64E-76	3 M1 _	4930578C19Rik
Pdgfb	4.09E-80	2.544089	0.951	0.193	8.23E-76	3 M1 _	Pdgfb
Cdh5	2.85E-80	2.237979	0.963	0.174	5.73E-76	3 M1 _	Cdh5
Ssfa21	2.83E-80	0.706379	0.427	0.023	5.70E-76	3 M1 _	Ssfa2
Fam167b	1.24E-81	1.86797	0.805	0.107	2.50E-77	3 M1 _	Fam167b
Akap21	7.66E-82	0.665999	0.354	0.014	1.54E-77	3 M1 _	Akap2
Cd93	4.46E-82	2.137187	0.939	0.158	8.98E-78	3 M1 _	Cd93
Magix	5.15E-83	0.76326	0.427	0.022	1.04E-78	3 M1 _	Magix
Ramp3	1.07E-84	1.86512	0.878	0.127	2.16E-80	3 M1 _	Ramp3
Tie1	5.51E-85	2.133856	0.951	0.161	1.11E-80	3 M1 _	Tie1
Car4	4.07E-85	1.923353	0.683	0.07	8.20E-81	3 M1 _	Car4
Kdr	1.13E-85	3.589535	0.988	0.211	2.27E-81	3 M1 _	Kdr
Rasgrp3	1.73E-86	1.853898	0.854	0.116	3.48E-82	3 M1 _	Rasgrp3
Cmtm8	4.93E-87	1.851554	0.805	0.102	9.92E-83	3 M1 _	Cmtm8
Rbp7	1.38E-87	3.471194	1	0.193	2.78E-83	3 M1 _	Rbp7
Cd300lg	2.39E-89	2.479438	0.939	0.156	4.82E-85	3 M1 _	Cd300lg
Adgrg1	2.01E-90	1.443022	0.78	0.089	4.06E-86	3 M1 _	Adgrg1
Podxl	1.08E-90	3.083257	0.976	0.18	2.17E-86	3 M1 _	Podxl
Tmem88	1.57E-91	1.522528	0.817	0.094	3.16E-87	3 M1 _	Tmem88
Fam198b1	4.34E-93	1.621172	0.768	0.074	8.75E-89	3 M1 _	Fam198b
1700020I14Rik2	8.07E-94	1.51086	0.793	0.084	1.63E-89	3 M1 _	1700020I14Rik
Arhgef15	4.98E-95	1.576205	0.854	0.101	1.00E-90	3 M1 _	Arhgef15
Esm1	5.09E-96	1.467349	0.5	0.027	1.02E-91	3 M1 _	Esm1
Cyyr1	7.46E-97	2.588824	0.963	0.145	1.50E-92	3 M1 _	Cyyr1
Clic5	2.11E-100	2.331695	0.951	0.131	4.25E-96	3 M1 _	Clic5
Thrsp	2.23E-101	2.873073	0.951	0.141	4.49E-97	3 M1 _	Thrsp
Btnl9	1.15E-102	3.064075	0.915	0.124	2.32E-98	3 M1 _	Btnl9
Mcf2l	5.94E-104	1.862431	0.878	0.103	1.20E-99	3 M1 _	Mcf2l
Ces2e	2.72E-104	1.622393	0.744	0.067	5.47E-100	3 M1 _	Ces2e
Cd24a	2.61E-107	2.470109	0.915	0.114	5.26E-103	3 M1 _	Cd24a
Zcchc63	5.83E-108	1.483826	0.805	0.075	1.17E-103	3 M1 _	Zcchc6
Exoc3l2	5.50E-112	1.98132	0.866	0.09	1.11E-107	3 M1 _	Exoc3l2
Tmem21	2.74E-123	0.744372	0.451	0.013	5.52E-119	3 M1 _	Tmem2
Mkl21	1.30E-140	1.461867	0.671	0.033	2.61E-136	3 M1 _	Mkl2
8430408G22Rik	1.37E-245	1.410893	0.512	0.001	2.77E-241	3 M1 _	8430408G22Rik
Steap32	2.32E-06	0.88659	0.941	0.36	0.046685	4 L1 _	Steap3

Acyp1	2.06E-06	0.260375	0.529	0.12	0.04148	4 L1 _	Acyp1
Adam17	1.87E-06	0.588332	0.882	0.336	0.037729	4 L1 _	Adam17
Serinc5	1.82E-06	0.486668	0.471	0.108	0.036664	4 L1 _	Serinc5
Trim30d1	1.82E-06	0.615831	0.471	0.111	0.036621	4 L1 _	Trim30d
Galnt171	1.80E-06	0.409765	0.412	0.085	0.036152	4 L1 _	Galnt17
Dock42	1.74E-06	0.400405	0.529	0.124	0.035141	4 L1 _	Dock4
Amotl21	1.52E-06	0.640423	0.706	0.225	0.030526	4 L1 _	Amotl2
Rpl37a2	1.48E-06	0.640381	1	0.985	0.029815	4 L1 _	Rpl37a
Dock111	1.44E-06	0.503889	0.588	0.15	0.02891	4 L1 _	Dock11
Rarres23	1.37E-06	0.956697	1	0.689	0.027659	4 L1 _	Rarres2
Tmem43	1.36E-06	0.567359	0.647	0.191	0.027308	4 L1 _	Tmem43
Fpgt	1.32E-06	0.342721	0.353	0.062	0.026533	4 L1 _	Fpgt
Rab11fip5	1.27E-06	0.57325	0.529	0.132	0.025653	4 L1 _	Rab11fip5
Fbln23	1.27E-06	1.053499	0.941	0.543	0.025585	4 L1 _	Fbln2
Tmem19	1.27E-06	0.361074	0.588	0.146	0.025482	4 L1 _	Tmem19
Cercam	1.17E-06	0.589471	0.471	0.108	0.023648	4 L1 _	Cercam
Sestd1	1.01E-06	0.529993	0.529	0.131	0.020298	4 L1 _	Sestd1
Prss231	9.87E-07	1.357291	0.941	0.72	0.01987	4 L1 _	Prss23
Atp10a	9.55E-07	0.331447	0.471	0.098	0.019228	4 L1 _	Atp10a
Triobp1	8.70E-07	0.684939	0.765	0.273	0.017531	4 L1 _	Triobp
Rack11	7.18E-07	0.737294	1	0.928	0.014461	4 L1 _	Rack1
Bicd11	7.03E-07	0.401253	0.294	0.044	0.014153	4 L1 _	Bicd1
Fgfr12	6.94E-07	0.969663	1	0.487	0.01398	4 L1 _	Fgfr1
Mcub	6.66E-07	0.308252	0.353	0.059	0.013413	4 L1 _	Mcub
Cpz1	6.11E-07	0.416222	0.471	0.095	0.012307	4 L1 _	Cpz
Rps282	6.01E-07	0.718733	1	0.962	0.012108	4 L1 _	Rps28
Abca61	5.99E-07	0.473043	0.529	0.122	0.01207	4 L1 _	Abca6
Tmeff1	5.49E-07	0.394826	0.235	0.029	0.011052	4 L1 _	Tmeff1
Wnt11	5.12E-07	0.334305	0.235	0.029	0.010311	4 L1 _	Wnt11
Radil	4.74E-07	0.295032	0.412	0.074	0.00954	4 L1 _	Radil
Ptgs2	4.67E-07	0.318467	0.176	0.016	0.009408	4 L1 _	Ptgs2
Thbs21	4.45E-07	0.75465	1	0.374	0.008965	4 L1 _	Thbs2
Trnp1	3.95E-07	0.413477	0.353	0.059	0.007949	4 L1 _	Trnp1
Mgst13	3.93E-07	0.973787	1	0.498	0.007914	4 L1 _	Mgst1
Gm50012	3.79E-07	0.394996	0.235	0.028	0.007639	4 L1 _	Gm50012
Anks1	3.66E-07	0.4579	0.588	0.135	0.007374	4 L1 _	Anks1
Ehd11	3.60E-07	0.629939	0.882	0.286	0.007254	4 L1 _	Ehd1
Palm3	3.57E-07	0.764554	0.765	0.248	0.007188	4 L1 _	Palm
Gsdme	3.57E-07	0.293449	0.412	0.074	0.007181	4 L1 _	Gsdme
Flrt22	3.46E-07	0.862576	0.941	0.376	0.006972	4 L1 _	Flrt2
Add32	2.87E-07	1.079693	1	0.505	0.005778	4 L1 _	Add3

Npc21	2.82E-07	0.957223	1	0.753	0.005677	4 L1 _	Npc2
Plat1	2.56E-07	0.963217	1	0.523	0.005161	4 L1 _	Plat
Sh3d192	2.42E-07	0.905805	0.941	0.408	0.004865	4 L1 _	Sh3d19
Jpt11	2.39E-07	0.987368	1	0.378	0.004817	4 L1 _	Jpt1
Plce11	2.31E-07	0.764958	0.706	0.204	0.004654	4 L1 _	Plce1
Plagl1	2.21E-07	0.377398	0.353	0.058	0.004449	4 L1 _	Plagl1
Spsb1	1.92E-07	0.777457	0.706	0.204	0.003862	4 L1 _	Spsb1
Mcc2	1.83E-07	0.279491	0.706	0.17	0.003694	4 L1 _	Mcc
Ddhd1	1.79E-07	0.522647	0.529	0.117	0.003604	4 L1 _	Ddhd1
Rab7b1	1.76E-07	0.571085	0.706	0.194	0.003544	4 L1 _	Rab7b
C430049B03Rik1	1.64E-07	0.810824	0.294	0.041	0.003311	4 L1 _	C430049B03Rik
Rps261	1.24E-07	0.83449	1	0.958	0.002506	4 L1 _	Rps26
Lacc1	1.11E-07	0.403572	0.471	0.091	0.002226	4 L1 _	Lacc1
Col1a12	1.00E-07	1.802513	1	0.672	0.002023	4 L1 _	Col1a1
Daglb1	9.78E-08	0.70984	0.471	0.095	0.00197	4 L1 _	Daglb
Gpsm2	9.40E-08	0.357994	0.353	0.054	0.001893	4 L1 _	Gpsm2
Col1a22	9.37E-08	1.690726	1	0.749	0.001887	4 L1 _	Col1a2
Vgll31	9.37E-08	1.013289	0.824	0.31	0.001887	4 L1 _	Vgll3
Ogn2	9.28E-08	1.448634	1	0.544	0.001869	4 L1 _	Ogn
Sod32	8.61E-08	1.062786	0.824	0.295	0.001734	4 L1 _	Sod3
Eef22	8.59E-08	0.825579	1	0.898	0.001729	4 L1 _	Eef2
Sulf11	8.20E-08	0.826682	0.941	0.346	0.001651	4 L1 _	Sulf1
Ssc5d1	8.15E-08	0.606653	0.588	0.145	0.001642	4 L1 _	Ssc5d
Glce1	8.14E-08	0.687527	0.529	0.116	0.00164	4 L1 _	Glce
Nmt2	7.99E-08	0.572633	0.765	0.229	0.00161	4 L1 _	Nmt2
Zfp976	7.74E-08	0.261417	0.235	0.025	0.001558	4 L1 _	Zfp976
Cacnb4	7.24E-08	0.267096	0.294	0.037	0.001458	4 L1 _	Cacnb4
Vasn1	6.79E-08	1.138175	0.824	0.297	0.001367	4 L1 _	Vasn
Pdxk1	6.63E-08	0.451866	0.529	0.107	0.001336	4 L1 _	Pdxk
Has2	6.10E-08	0.293058	0.294	0.038	0.001228	4 L1 _	Has2
Pdpn2	6.02E-08	1.225702	0.882	0.377	0.001213	4 L1 _	Pdpn
Ndst2	5.93E-08	0.385828	0.412	0.07	0.001195	4 L1 _	Ndst2
Nqo22	5.78E-08	0.516991	0.588	0.137	0.001164	4 L1 _	Nqo2
Meg32	5.40E-08	1.584149	1	0.648	0.001088	4 L1 _	Meg3
Gm45187	5.23E-08	0.316938	0.176	0.014	0.001054	4 L1 _	Gm45187
Prcp	4.46E-08	0.468886	0.588	0.13	0.000899	4 L1 _	Prcp
Ttc39c1	4.24E-08	0.294473	0.412	0.066	0.000854	4 L1 _	Ttc39c
Tmem1581	4.24E-08	0.835027	0.824	0.263	0.000854	4 L1 _	Tmem158
Rps151	4.12E-08	0.954493	1	0.951	0.000831	4 L1 _	Rps15
Plac82	3.96E-08	0.854616	1	0.361	0.000797	4 L1 _	Plac8
Hdac73	3.83E-08	1.147702	1	0.448	0.000771	4 L1 _	Hdac7

Rbpj1	3.32E-08	1.024413	1	0.442	0.00067	4 L1 _	Rbpj
Plpp33	2.89E-08	1.383127	1	0.729	0.000581	4 L1 _	Plpp3
Agpat4	2.59E-08	0.995757	0.765	0.241	0.000522	4 L1 _	Agpat4
Ptprj	2.55E-08	0.38856	0.471	0.083	0.000513	4 L1 _	Ptprj
Sh3pxd2b2	2.32E-08	0.807654	0.941	0.324	0.000467	4 L1 _	Sh3pxd2b
B3galnt11	2.11E-08	0.393667	0.471	0.082	0.000425	4 L1 _	B3galnt1
Arhgap422	2.05E-08	0.662543	0.765	0.199	0.000413	4 L1 _	Arhgap42
Ahnak21	2.05E-08	1.342545	0.882	0.35	0.000413	4 L1 _	Ahnak2
Six1	1.61E-08	0.293356	0.235	0.023	0.000323	4 L1 _	Six1
Gga2	1.51E-08	0.538568	0.647	0.149	0.000304	4 L1 _	Gga2
Rps121	1.49E-08	0.896084	1	0.974	0.0003	4 L1 _	Rps12
Ahnak4	1.37E-08	1.046011	1	0.894	0.000275	4 L1 _	Ahnak
Notch21	1.28E-08	0.658108	0.765	0.21	0.000257	4 L1 _	Notch2
Kank11	1.22E-08	0.543485	0.588	0.122	0.000246	4 L1 _	Kank1
9130008F23Rik	1.17E-08	0.381259	0.353	0.048	0.000236	4 L1 _	9130008F23Rik
Alcam	1.14E-08	0.286299	0.294	0.034	0.000231	4 L1 _	Alcam
Disp1	1.05E-08	0.536434	0.471	0.083	0.000212	4 L1 _	Disp1
Fbln52	9.15E-09	0.997408	1	0.386	0.000184	4 L1 _	Fbln5
Col5a13	8.84E-09	1.329154	0.941	0.512	0.000178	4 L1 _	Col5a1
Col3a12	8.56E-09	2.08133	1	0.756	0.000172	4 L1 _	Col3a1
Dkk2	8.55E-09	0.502965	0.118	0.006	0.000172	4 L1 _	Dkk2
S100a111	8.37E-09	1.250021	1	0.863	0.000169	4 L1 _	S100a11
Fyn1	8.37E-09	1.254978	0.882	0.398	0.000169	4 L1 _	Fyn
Igfbp52	8.29E-09	2.292937	0.941	0.515	0.000167	4 L1 _	Igfbp5
Adamts53	7.10E-09	1.472048	1	0.523	0.000143	4 L1 _	Adamts5
Ly6a2	6.82E-09	1.628061	1	0.731	0.000137	4 L1 _	Ly6a
Adamtsl51	5.46E-09	0.414452	0.647	0.132	0.00011	4 L1 _	Adamtsl5
Plekho2	5.26E-09	0.911535	0.706	0.181	0.000106	4 L1 _	Plekho2
Fam102b2	5.18E-09	1.027984	0.882	0.313	0.000104	4 L1 _	Fam102b
Gpnmb1	4.30E-09	0.415003	0.529	0.093	8.66E-05	4 L1 _	Gpnmb
Slco3a12	4.28E-09	0.872541	0.941	0.319	8.63E-05	4 L1 _	Slco3a1
Loxl22	4.14E-09	1.036245	0.941	0.326	8.34E-05	4 L1 _	Loxl2
Ablim13	4.05E-09	1.21337	1	0.395	8.16E-05	4 L1 _	Ablim1
Ly6c12	4.05E-09	1.34386	1	0.423	8.15E-05	4 L1 _	Ly6c1
Cavin13	3.60E-09	1.265676	1	0.48	7.25E-05	4 L1 _	Cavin1
Lama41	3.48E-09	1.501146	1	0.517	7.00E-05	4 L1 _	Lama4
Ifi2052	3.02E-09	0.950907	0.941	0.294	6.08E-05	4 L1 _	Ifi205
Ugp2	2.42E-09	1.025285	0.882	0.314	4.87E-05	4 L1 _	Ugp2
Serpinb6a3	2.31E-09	1.315362	1	0.712	4.66E-05	4 L1 _	Serpinb6a
Itsn11	2.24E-09	0.68963	0.824	0.214	4.51E-05	4 L1 _	Itsn1
Tbx181	1.99E-09	0.395186	0.412	0.058	4.02E-05	4 L1 _	Tbx18

Arhgap202	1.97E-09	0.987245	0.824	0.241	3.96E-05	4 L1 _	Arhgap20
Mfap52	1.86E-09	2.348267	0.941	0.535	3.75E-05	4 L1 _	Mfap5
Il1rl2	1.58E-09	1.030387	0.588	0.129	3.19E-05	4 L1 _	Il1rl2
Sntb22	1.47E-09	1.105992	1	0.549	2.96E-05	4 L1 _	Sntb2
Pde8a3	1.31E-09	0.874405	0.765	0.199	2.64E-05	4 L1 _	Pde8a
Lims22	1.16E-09	0.735812	0.706	0.152	2.34E-05	4 L1 _	Lims2
Marcks2	1.10E-09	1.470108	1	0.823	2.21E-05	4 L1 _	Marcks
S100a102	1.01E-09	1.656086	1	0.69	2.03E-05	4 L1 _	S100a10
Gngt22	9.43E-10	1.06836	0.882	0.257	1.90E-05	4 L1 _	Gngt2
St3gal2	8.36E-10	0.828949	0.882	0.266	1.68E-05	4 L1 _	St3gal2
Scara53	7.81E-10	1.593532	1	0.405	1.57E-05	4 L1 _	Scara5
Mmp22	7.73E-10	1.721493	1	0.56	1.56E-05	4 L1 _	Mmp2
Gm101151	6.34E-10	0.801989	0.588	0.115	1.28E-05	4 L1 _	Gm10115
Uap12	5.25E-10	1.003202	0.882	0.263	1.06E-05	4 L1 _	Uap1
Axl3	4.91E-10	1.411504	1	0.577	9.89E-06	4 L1 _	Axl
Pla1a1	4.57E-10	2.011282	0.882	0.414	9.20E-06	4 L1 _	Pla1a
Lurap1l2	3.13E-10	0.516089	0.471	0.068	6.31E-06	4 L1 _	Lurap1l
Ppp1r14b1	3.00E-10	1.869972	1	0.483	6.05E-06	4 L1 _	Ppp1r14b
Mrgprf	2.74E-10	0.395954	0.588	0.098	5.51E-06	4 L1 _	Mrgprf
Bmp13	2.38E-10	1.566358	0.941	0.439	4.78E-06	4 L1 _	Bmp1
Lvrn	2.19E-10	0.596819	0.471	0.071	4.41E-06	4 L1 _	Lvrn
Basp13	2.19E-10	1.42166	0.941	0.373	4.41E-06	4 L1 _	Basp1
Aebp11	1.91E-10	1.80296	1	0.629	3.84E-06	4 L1 _	Aebp1
Klhl132	1.05E-10	0.704378	0.706	0.148	2.12E-06	4 L1 _	Klhl13
Dcbld2	8.11E-11	0.812964	0.882	0.227	1.63E-06	4 L1 _	Dcbld2
Nt5dc3	6.61E-11	0.258187	0.353	0.037	1.33E-06	4 L1 _	Nt5dc3
Hspb81	5.50E-11	0.585444	0.647	0.118	1.11E-06	4 L1 _	Hspb8
Lmnb1	5.02E-11	0.469036	0.471	0.066	1.01E-06	4 L1 _	Lmnb1
Kctd9	4.88E-11	0.537135	0.529	0.082	9.83E-07	4 L1 _	Kctd9
Tgfbr23	4.38E-11	1.700508	1	0.651	8.83E-07	4 L1 _	Tgfbr2
Poglut31	3.97E-11	0.991028	0.882	0.245	8.00E-07	4 L1 _	Poglut3
Dnase2a	3.85E-11	0.618937	0.647	0.12	7.75E-07	4 L1 _	Dnase2a
Gan	3.78E-11	0.703689	0.647	0.122	7.62E-07	4 L1 _	Gan
Ecm12	2.78E-11	2.344265	0.941	0.48	5.60E-07	4 L1 _	Ecm1
Sema3d	2.41E-11	0.645867	0.529	0.082	4.86E-07	4 L1 _	Sema3d
Gxylt21	2.36E-11	0.913674	0.765	0.173	4.75E-07	4 L1 _	Gxylt2
Anxa13	1.69E-11	1.994467	1	0.571	3.40E-07	4 L1 _	Anxa1
Ttyh2	1.54E-11	0.558197	0.588	0.094	3.09E-07	4 L1 _	Ttyh2
Nid13	1.25E-11	2.022071	1	0.733	2.51E-07	4 L1 _	Nid1
Zyx2	1.11E-11	1.421526	1	0.433	2.23E-07	4 L1 _	Zyx
C33	9.27E-12	2.684961	1	0.395	1.87E-07	4 L1 _	C3

Gnpnat1	8.87E-12	0.894615	0.824	0.197	1.79E-07	4 L1 _	Gnpnat1
Epdr1	8.10E-12	0.577854	0.471	0.062	1.63E-07	4 L1 _	Epdr1
Dclk12	7.47E-12	1.068478	0.882	0.216	1.51E-07	4 L1 _	Dclk1
Eya4	6.69E-12	0.329875	0.235	0.016	1.35E-07	4 L1 _	Eya4
Fstl12	5.44E-12	2.087933	1	0.698	1.10E-07	4 L1 _	Fstl1
Grb102	5.25E-12	1.238077	1	0.353	1.06E-07	4 L1 _	Grb10
Sulf22	5.10E-12	0.834476	0.882	0.2	1.03E-07	4 L1 _	Sulf2
Rian	4.77E-12	1.533597	0.765	0.177	9.60E-08	4 L1 _	Rian
Gfpt23	2.90E-12	1.471783	1	0.317	5.84E-08	4 L1 _	Gfpt2
Sec22c	2.74E-12	0.465725	0.412	0.046	5.52E-08	4 L1 _	Sec22c
Cd344	2.72E-12	2.141541	1	0.445	5.47E-08	4 L1 _	Cd34
Plxna11	1.98E-12	0.753371	0.882	0.199	3.98E-08	4 L1 _	Plxna1
Tmem1004	1.73E-12	1.752872	0.941	0.282	3.48E-08	4 L1 _	Tmem100
Cd276	1.22E-12	0.389117	0.412	0.045	2.46E-08	4 L1 _	Cd276
Dbn11	1.12E-12	1.468456	0.882	0.258	2.25E-08	4 L1 _	Dbn1
Itpr32	1.10E-12	0.601959	0.588	0.087	2.22E-08	4 L1 _	Itpr3
Timp23	1.07E-12	2.853402	1	0.704	2.15E-08	4 L1 _	Timp2
Adam331	8.53E-13	0.572745	0.412	0.044	1.72E-08	4 L1 _	Adam33
B830012L14Rik	7.87E-13	0.465476	0.412	0.044	1.58E-08	4 L1 _	B830012L14Rik
Igfbp62	6.59E-13	2.748685	1	0.486	1.33E-08	4 L1 _	Igfbp6
Medag3	5.18E-13	1.643665	1	0.329	1.04E-08	4 L1 _	Medag
Npr13	4.85E-13	0.826511	0.706	0.12	9.76E-09	4 L1 _	Npr1
Ace5	3.50E-13	1.719893	1	0.394	7.05E-09	4 L1 _	Ace
Fbn13	3.34E-13	3.033151	1	0.62	6.73E-09	4 L1 _	Fbn1
Sdk11	3.19E-13	0.330933	0.412	0.041	6.42E-09	4 L1 _	Sdk1
Heg13	2.55E-13	1.58578	1	0.335	5.13E-09	4 L1 _	Heg1
Atp1b12	2.49E-13	0.939554	0.706	0.122	5.02E-09	4 L1 _	Atp1b1
Xpnpep21	2.07E-13	0.489422	0.529	0.066	4.17E-09	4 L1 _	Xpnpep2
Bmp61	1.79E-13	0.50086	0.412	0.043	3.60E-09	4 L1 _	Bmp6
Ddr22	1.46E-13	1.861772	1	0.44	2.94E-09	4 L1 _	Ddr2
Tnfaip61	1.10E-13	0.938984	0.765	0.147	2.22E-09	4 L1 _	Tnfaip6
Col14a13	5.19E-14	2.73025	1	0.456	1.04E-09	4 L1 _	Col14a1
Adamts23	4.45E-14	1.80584	0.941	0.305	8.97E-10	4 L1 _	Adamts2
Galnt163	3.67E-14	0.788785	0.765	0.126	7.39E-10	4 L1 _	Galnt16
Gm48617	3.21E-14	0.260424	0.235	0.013	6.46E-10	4 L1 _	Gm48617
Has2os	2.97E-14	0.345562	0.353	0.029	5.98E-10	4 L1 _	Has2os
Tek3	2.29E-14	0.981087	0.882	0.161	4.61E-10	4 L1 _	Tek
Cul9	2.28E-14	0.623022	0.588	0.078	4.60E-10	4 L1 _	Cul9
Car83	1.34E-14	1.023029	0.529	0.065	2.70E-10	4 L1 _	Car8
Irak3	1.20E-14	0.845708	0.882	0.168	2.42E-10	4 L1 _	Irak3
Gm9780	1.17E-14	0.280988	0.353	0.028	2.35E-10	4 L1 _	Gm9780

Sirpa1	9.25E-15	0.695273	0.588	0.079	1.86E-10	4 L1 _	Sirpa
Clec3b3	7.53E-15	3.315716	1	0.433	1.52E-10	4 L1 _	Clec3b
Klf43	5.51E-15	2.339315	1	0.311	1.11E-10	4 L1 _	Klf4
Cd2482	5.25E-15	2.939691	1	0.421	1.06E-10	4 L1 _	Cd248
Efemp13	4.33E-15	2.665256	1	0.353	8.73E-11	4 L1 _	Efemp1
Ebf23	4.14E-15	1.495473	0.882	0.174	8.33E-11	4 L1 _	Ebf2
Igdcc4	4.10E-15	0.420777	0.471	0.048	8.25E-11	4 L1 _	Igdcc4
Glipr22	4.05E-15	0.886252	0.824	0.154	8.15E-11	4 L1 _	Glipr2
Mmp271	3.44E-15	0.288127	0.235	0.012	6.93E-11	4 L1 _	Mmp27
Gas72	3.17E-15	2.525652	1	0.351	6.37E-11	4 L1 _	Gas7
Mmp232	2.68E-15	1.491312	0.882	0.204	5.40E-11	4 L1 _	Mmp23
Rhou2	1.07E-15	0.825292	0.824	0.138	2.16E-11	4 L1 _	Rhou
Procr2	7.93E-16	1.191487	0.765	0.123	1.60E-11	4 L1 _	Procr
Antxr12	7.77E-16	1.963724	1	0.302	1.57E-11	4 L1 _	Antxr1
Dact11	6.79E-16	1.141566	0.941	0.204	1.37E-11	4 L1 _	Dact1
Man1a2	6.40E-16	1.819727	1	0.333	1.29E-11	4 L1 _	Man1a
Tubb2b1	6.38E-16	0.964885	0.765	0.122	1.28E-11	4 L1 _	Tubb2b
Csrp21	5.08E-16	2.131391	1	0.286	1.02E-11	4 L1 _	Csrp2
Cdon2	3.92E-16	1.234215	0.706	0.106	7.89E-12	4 L1 _	Cdon
Tubb4a2	2.13E-16	0.414017	0.353	0.025	4.30E-12	4 L1 _	Tubb4a
Gprc5b1	1.62E-16	0.765337	0.588	0.07	3.27E-12	4 L1 _	Gprc5b
AW551984	1.60E-16	0.77146	0.529	0.057	3.23E-12	4 L1 _	AW551984
Ugdh2	1.46E-16	2.602996	0.941	0.24	2.94E-12	4 L1 _	Ugdh
Zfpm2	1.22E-16	0.767278	0.647	0.086	2.45E-12	4 L1 _	Zfpm2
C1qtnf12	1.16E-16	1.125706	0.882	0.162	2.33E-12	4 L1 _	C1qtnf1
Bdh21	9.99E-17	0.504387	0.529	0.053	2.01E-12	4 L1 _	Bdh2
Sytl23	9.97E-17	0.426518	0.647	0.075	2.01E-12	4 L1 _	Sytl2
Rras2	6.99E-17	0.853575	0.706	0.097	1.41E-12	4 L1 _	Rras2
Fez12	6.51E-17	0.513539	0.588	0.065	1.31E-12	4 L1 _	Fez1
Anxa34	6.03E-17	2.871307	1	0.311	1.22E-12	4 L1 _	Anxa3
Scara31	3.18E-17	1.887871	0.941	0.232	6.40E-13	4 L1 _	Scara3
Hebp2	2.43E-17	0.451512	0.588	0.063	4.89E-13	4 L1 _	Hebp2
Metrnl	7.61E-18	1.789003	1	0.26	1.53E-13	4 L1 _	Metrnl
Zfp385a1	5.81E-18	1.33047	0.765	0.121	1.17E-13	4 L1 _	Zfp385a
Pi162	2.95E-18	3.569863	1	0.263	5.95E-14	4 L1 _	Pi16
Thbs31	1.95E-18	1.250653	0.882	0.153	3.92E-14	4 L1 _	Thbs3
Osr12	2.31E-19	1.96781	0.941	0.188	4.64E-15	4 L1 _	Osr1
Srgap12	2.54E-20	0.927014	0.647	0.069	5.11E-16	4 L1 _	Srgap1
Olfml2b3	2.33E-20	1.219085	0.882	0.126	4.69E-16	4 L1 _	Olfml2b
Tnxb2	1.59E-20	3.241293	1	0.235	3.20E-16	4 L1 _	Tnxb
Flnc2	6.99E-21	0.954711	0.824	0.116	1.41E-16	4 L1 _	Flnc

Lgi21	6.55E-21	1.555193	0.824	0.12	1.32E-16	4 L1 _	Lgi2
C4b2	4.01E-21	2.122394	0.706	0.084	8.08E-17	4 L1 _	C4b
Tppp32	3.99E-21	1.860873	1	0.187	8.04E-17	4 L1 _	Tppp3
Twist2	3.60E-21	0.593676	0.529	0.044	7.26E-17	4 L1 _	Twist2
5730507C01Rik	3.46E-21	0.320508	0.235	0.008	6.97E-17	4 L1 _	5730507C01Rik
Lamc21	3.13E-21	0.439028	0.235	0.008	6.31E-17	4 L1 _	Lamc2
Nhsl11	1.96E-21	0.989859	0.765	0.095	3.95E-17	4 L1 _	Nhsl1
Mustn13	5.57E-22	1.224018	0.882	0.116	1.12E-17	4 L1 _	Mustn1
Nox41	5.26E-22	0.93541	0.706	0.075	1.06E-17	4 L1 _	Nox4
Kif21a1	3.32E-22	0.502649	0.412	0.025	6.68E-18	4 L1 _	Kif21a
Gm12122	1.06E-22	0.402596	0.471	0.031	2.14E-18	4 L1 _	Gm12122
Arl4d2	4.78E-23	0.710691	0.588	0.049	9.62E-19	4 L1 _	Arl4d
Megf91	3.49E-23	0.730176	0.706	0.07	7.04E-19	4 L1 _	Megf9
Pi15	3.28E-23	1.572167	0.765	0.088	6.61E-19	4 L1 _	Pi15
Il6ra1	2.48E-23	0.528055	0.471	0.031	4.99E-19	4 L1 _	Il6ra
Ildr21	1.48E-23	1.625999	0.765	0.091	2.98E-19	4 L1 _	Ildr2
Ndrg41	8.75E-24	0.493173	0.471	0.03	1.76E-19	4 L1 _	Ndrg4
Wnt10b	8.36E-24	0.429525	0.412	0.023	1.68E-19	4 L1 _	Wnt10b
Arsi1	3.79E-24	0.906493	0.588	0.049	7.63E-20	4 L1 _	Arsi
Rab32	2.16E-24	0.672663	0.529	0.038	4.35E-20	4 L1 _	Rab32
Qpct1	3.58E-25	1.536949	0.882	0.119	7.21E-21	4 L1 _	Qpct
Gpc32	2.41E-25	1.54556	0.765	0.08	4.86E-21	4 L1 _	Gpc3
Sel1l3	2.33E-25	0.375309	0.176	0.003	4.68E-21	4 L1 _	Sel1l3
Pygl1	2.07E-25	0.915745	0.706	0.066	4.18E-21	4 L1 _	Pygl
Wnt21	1.69E-25	1.156696	0.647	0.056	3.39E-21	4 L1 _	Wnt2
Prdm81	1.42E-25	0.622075	0.412	0.022	2.86E-21	4 L1 _	Prdm8
Emilin22	2.30E-26	2.544684	1	0.169	4.63E-22	4 L1 _	Emilin2
Itga111	9.99E-27	1.058857	0.765	0.075	2.01E-22	4 L1 _	Itga11
Dpt2	2.97E-27	4.387907	1	0.165	5.98E-23	4 L1 _	Dpt
Fn12	7.88E-28	4.737653	1	0.159	1.59E-23	4 L1 _	Fn1
Cadm32	1.07E-28	0.941645	0.706	0.056	2.15E-24	4 L1 _	Cadm3
Stxbp61	1.83E-30	0.531314	0.588	0.035	3.69E-26	4 L1 _	Stxbp6
Gda3	1.71E-30	1.514098	0.824	0.078	3.45E-26	4 L1 _	Gda
Ackr33	7.08E-32	2.375167	1	0.12	1.43E-27	4 L1 _	Ackr3
Slc4a41	3.48E-32	1.050363	0.647	0.044	7.01E-28	4 L1 _	Slc4a4
Limch12	3.09E-32	1.265872	0.882	0.08	6.22E-28	4 L1 _	Limch1
5033421B08Rik1	1.76E-32	0.335146	0.235	0.005	3.55E-28	4 L1 _	5033421B08Rik
Pamr11	9.29E-33	1.798363	0.882	0.088	1.87E-28	4 L1 _	Pamr1
Tnfrsf11b	2.69E-34	0.355425	0.412	0.015	5.42E-30	4 L1 _	Tnfrsf11b
Stmn41	1.13E-34	0.335737	0.235	0.004	2.28E-30	4 L1 _	Stmn4
Sema3c3	4.81E-35	3.228118	1	0.116	9.69E-31	4 L1 _	Sema3c

Cd552	1.35E-35	2.641034	1	0.114	2.72E-31	4 L1 _	Cd55
Fhl13	9.78E-36	1.094054	0.882	0.07	1.97E-31	4 L1 _	Fhl1
Hoxb31	3.13E-37	0.280678	0.235	0.004	6.30E-33	4 L1 _	Hoxb3
Itgb3	4.04E-38	0.880249	0.882	0.069	8.15E-34	4 L1 _	Itgb3
Fndc11	3.32E-38	3.527999	1	0.106	6.69E-34	4 L1 _	Fndc1
Pcdh20	2.24E-38	0.598472	0.353	0.009	4.51E-34	4 L1 _	Pcdh20
Dmkn1	1.84E-39	1.154061	0.176	0.002	3.70E-35	4 L1 _	Dmkn
Adra1b1	2.47E-40	0.27629	0.235	0.003	4.97E-36	4 L1 _	Adra1b
Krtdap	3.27E-41	1.569889	0.118	0	6.58E-37	4 L1 _	Krtdap
Vldlr2	2.04E-41	1.492355	0.882	0.064	4.11E-37	4 L1 _	Vldlr
Ackr11	1.01E-41	0.461857	0.471	0.016	2.04E-37	4 L1 _	Ackr1
Sfrp22	5.19E-43	3.42122	1	0.089	1.05E-38	4 L1 _	Sfrp2
Kcnk51	3.49E-43	0.634727	0.588	0.025	7.02E-39	4 L1 _	Kcnk5
Chrdl11	1.01E-44	0.717095	0.353	0.008	2.02E-40	4 L1 _	Chrdl1
Vegfd1	3.61E-45	0.717319	0.588	0.024	7.28E-41	4 L1 _	Vegfd
Tbx5	7.86E-46	0.308476	0.176	0.001	1.58E-41	4 L1 _	Tbx5
Fzd10	7.86E-46	0.262312	0.176	0.001	1.58E-41	4 L1 _	Fzd10
Ano3	6.59E-46	0.319128	0.176	0.001	1.33E-41	4 L1 _	Ano3
Pcsk62	2.06E-46	2.2421	0.882	0.059	4.15E-42	4 L1 _	Pcsk6
Ackr21	5.29E-47	0.574672	0.353	0.007	1.07E-42	4 L1 _	Ackr2
Clip41	4.61E-48	1.241983	0.824	0.047	9.29E-44	4 L1 _	Clip4
Rnf165	1.51E-49	0.622857	0.529	0.017	3.03E-45	4 L1 _	Rnf165
Cmah2	1.42E-52	1.581066	0.882	0.049	2.86E-48	4 L1 _	Cmah
Gm267711	5.65E-53	0.766776	0.588	0.02	1.14E-48	4 L1 _	Gm26771
Sema3b	1.22E-53	0.919961	0.529	0.016	2.45E-49	4 L1 _	Sema3b
Gpr11	6.13E-54	0.417401	0.294	0.004	1.23E-49	4 L1 _	Gpr1
Adamts16	6.81E-55	0.284603	0.176	0.001	1.37E-50	4 L1 _	Adamts16
Ston21	3.98E-55	0.983861	0.588	0.019	8.02E-51	4 L1 _	Ston2
Lrrc172	2.21E-55	1.813236	1	0.063	4.44E-51	4 L1 _	Lrrc17
Sbsn1	2.83E-56	2.853742	0.941	0.055	5.70E-52	4 L1 _	Sbsn
Lrrn4cl1	1.46E-57	1.712321	0.824	0.039	2.95E-53	4 L1 _	Lrrn4cl
Dact21	1.50E-59	1.36252	0.706	0.026	3.02E-55	4 L1 _	Dact2
Smpd31	1.17E-59	2.334909	0.471	0.01	2.35E-55	4 L1 _	Smpd3
Gm156751	7.50E-60	0.325077	0.353	0.005	1.51E-55	4 L1 _	Gm15675
Sfrp41	1.23E-61	1.701221	0.588	0.016	2.49E-57	4 L1 _	Sfrp4
Ccn3	5.12E-62	1.303772	0.588	0.016	1.03E-57	4 L1 _	Ccn3
Cthrc1	1.74E-62	1.52729	0.588	0.016	3.50E-58	4 L1 _	Cthrc1
Creb51	1.24E-62	2.845729	1	0.056	2.50E-58	4 L1 _	Creb5
En11	6.50E-63	0.573903	0.294	0.003	1.31E-58	4 L1 _	En1
Gap43	3.55E-63	0.765104	0.471	0.009	7.14E-59	4 L1 _	Gap43
Efhd13	1.01E-65	1.5986	0.882	0.037	2.03E-61	4 L1 _	Efhd1

Gm15902	3.08E-67	0.402922	0.353	0.004	6.21E-63	4 L1 _	Gm15902
Rorb1	1.28E-67	0.760623	0.353	0.004	2.58E-63	4 L1 _	Rorb
1700019D03Rik1	4.00E-74	1.289143	0.765	0.024	8.06E-70	4 L1 _	1700019D03Rik
Efna51	2.24E-74	0.805183	0.588	0.013	4.52E-70	4 L1 _	Efna5
Adgrg21	2.15E-75	0.413009	0.294	0.002	4.32E-71	4 L1 _	Adgrg2
Dmrt2	1.80E-75	0.440102	0.294	0.002	3.62E-71	4 L1 _	Dmrt2
Edn12	5.20E-76	1.267375	0.824	0.026	1.05E-71	4 L1 _	Edn1
Adamtsl41	4.51E-83	1.377788	0.941	0.033	9.09E-79	4 L1 _	Adamtsl4
Tmeff21	4.04E-83	1.259145	0.824	0.024	8.14E-79	4 L1 _	Tmeff2
Adgrd11	3.95E-83	1.868371	1	0.039	7.95E-79	4 L1 _	Adgrd1
Il181	1.44E-84	0.898147	0.765	0.02	2.91E-80	4 L1 _	Il18
Aldh1a31	3.66E-87	1.272624	0.765	0.019	7.37E-83	4 L1 _	Aldh1a3
Fzd10os	3.43E-92	0.567243	0.412	0.004	6.91E-88	4 L1 _	Fzd10os
Aif1l1	3.78E-94	0.952993	0.765	0.017	7.60E-90	4 L1 _	Aif1l
Sema3e	3.89E-95	0.663828	0.471	0.005	7.83E-91	4 L1 _	Sema3e
Dpp41	4.95E-104	2.708736	1	0.03	9.98E-100	4 L1 _	Dpp4
Nova11	9.33E-111	1.5914	0.824	0.017	1.88E-106	4 L1 _	Nova1
Opcml1	5.46E-114	1.975968	0.941	0.022	1.10E-109	4 L1 _	Opcml
Gabra2	8.32E-121	0.343529	0.235	0	1.68E-116	4 L1 _	Gabra2
Krt19	1.22E-133	1.006143	0.412	0.002	2.45E-129	4 L1 _	Krt19
Duoxa11	3.41E-135	0.917152	0.529	0.004	6.87E-131	4 L1 _	Duoxa1
Pcolce21	6.45E-142	3.118156	1	0.019	1.30E-137	4 L1 _	Pcolce2
Mrgprg	1.41E-150	0.713379	0.294	0	2.84E-146	4 L1 _	Mrgprg
Dhrs9	1.25E-154	0.573111	0.353	0	2.53E-150	4 L1 _	Dhrs9
Lgi3	6.10E-160	0.533451	0.471	0.002	1.23E-155	4 L1 _	Lgi3
Bmp31	1.61E-172	0.999039	0.647	0.004	3.24E-168	4 L1 _	Bmp3
Gm43050	2.29E-180	0.431297	0.353	0	4.62E-176	4 L1 _	Gm43050
Krt36	2.29E-180	0.369443	0.353	0	4.62E-176	4 L1 _	Krt36
Duox11	2.10E-186	0.746668	0.529	0.002	4.24E-182	4 L1 _	Duox1
Islr21	1.65E-187	1.146162	0.765	0.006	3.32E-183	4 L1 _	Islr2
Akr1c18	0	3.021516	0.824	0.001	0	4 L1 _	Akr1c18
Itgb71	2.23E-06	0.68378	0.5	0.084	0.044978	4 M1 _	Itgb7
Basp11	2.15E-06	1.010263	1	0.375	0.043358	4 M1 _	Basp1
Col5a11	2.15E-06	1.500699	1	0.513	0.043209	4 M1 _	Col5a1
Gstm12	2.14E-06	1.747277	1	0.665	0.043076	4 M1 _	Gstm1
Fam126a1	2.07E-06	0.789595	0.7	0.164	0.041731	4 M1 _	Fam126a
Antxr21	2.00E-06	1.73798	0.9	0.394	0.040324	4 M1 _	Antxr2
Lrrc8b1	2.00E-06	0.514652	0.4	0.053	0.040219	4 M1 _	Lrrc8b
Egfr1	1.98E-06	1.012504	0.9	0.311	0.039818	4 M1 _	Egfr
Sytl21	1.90E-06	0.383055	0.5	0.077	0.038242	4 M1 _	Sytl2
Ndrg4	1.88E-06	0.414759	0.3	0.032	0.037854	4 M1 _	Ndrg4

Adamts51	1.84E-06	1.812988	1	0.524	0.037043	4 M1 _	Adamts5
Bdh2	1.83E-06	0.557715	0.4	0.055	0.036763	4 M1 _	Bdh2
Zyx	1.81E-06	1.364988	1	0.435	0.036421	4 M1 _	Zyx
Glce	1.71E-06	0.71429	0.6	0.117	0.034352	4 M1 _	Glce
Ccdc174	1.62E-06	0.911141	1	0.334	0.032645	4 M1 _	Ccdc174
Il17ra	1.47E-06	0.825026	0.6	0.12	0.029562	4 M1 _	Il17ra
2810403A07Rik1	1.40E-06	0.30716	0.3	0.031	0.028147	4 M1 _	2810403A07Rik
Rnase41	1.35E-06	1.512482	1	0.601	0.027158	4 M1 _	Rnase4
Cep295nl	1.29E-06	0.282322	0.2	0.014	0.025896	4 M1 _	Cep295nl
B3galnt1	1.22E-06	0.607004	0.5	0.083	0.024534	4 M1 _	B3galnt1
Glipr21	1.21E-06	0.719146	0.7	0.156	0.024362	4 M1 _	Glipr2
Chst11	1.20E-06	0.706921	0.6	0.113	0.024121	4 M1 _	Chst1
1700020I14Rik3	1.09E-06	0.772789	0.6	0.107	0.022048	4 M1 _	1700020I14Rik
Mmp23	1.04E-06	0.968367	0.8	0.207	0.020911	4 M1 _	Mmp23
Mgst11	1.01E-06	1.641805	1	0.5	0.020301	4 M1 _	Mgst1
Serpinb6a1	9.45E-07	1.724218	1	0.712	0.019033	4 M1 _	Serpinb6a
1810022K09Rik4	8.61E-07	0.631056	0.6	0.102	0.017346	4 M1 _	1810022K09Rik
Mfap5	8.08E-07	2.238491	1	0.536	0.016264	4 M1 _	Mfap5
Il1r2	7.21E-07	0.87028	0.4	0.055	0.014515	4 M1 _	Il1r2
Oxsm	6.95E-07	0.403949	0.4	0.051	0.013996	4 M1 _	Oxsm
Lgi41	6.65E-07	0.858864	0.7	0.151	0.013402	4 M1 _	Lgi4
Sod31	6.28E-07	0.820639	1	0.295	0.012655	4 M1 _	Sod3
Efs1	6.23E-07	0.53611	0.6	0.11	0.012543	4 M1 _	Efs
Minos14	6.09E-07	0.609778	0.7	0.126	0.012269	4 M1 _	Minos1
Hdac71	5.91E-07	1.513884	1	0.45	0.011908	4 M1 _	Hdac7
Nid11	5.89E-07	1.922222	1	0.734	0.011857	4 M1 _	Nid1
Ppp1r14b	5.53E-07	1.432392	1	0.485	0.011147	4 M1 _	Ppp1r14b
Zcchc114	5.40E-07	0.494541	0.5	0.072	0.010883	4 M1 _	Zcchc11
Coro1b3	5.27E-07	0.535392	0.6	0.103	0.010612	4 M1 _	Coro1b
Tmem55b3	5.25E-07	0.432078	0.4	0.049	0.010567	4 M1 _	Tmem55b
Sulf2	5.03E-07	1.088807	0.8	0.202	0.010137	4 M1 _	Sulf2
Anxa12	5.01E-07	2.09083	1	0.573	0.01008	4 M1 _	Anxa1
Adamtsl5	4.81E-07	0.484078	0.7	0.134	0.009696	4 M1 _	Adamtsl5
Bmp11	3.67E-07	1.578271	1	0.441	0.007386	4 M1 _	Bmp1
Snhg11	3.27E-07	0.477017	0.2	0.013	0.006584	4 M1 _	Snhg11
Adgra3	2.93E-07	0.748793	0.7	0.143	0.005902	4 M1 _	Adgra3
D630033O11Rik	2.88E-07	0.732351	0.6	0.104	0.005802	4 M1 _	D630033O11Rik
Atp5o.14	2.79E-07	0.639437	0.7	0.123	0.005626	4 M1 _	Atp5o.1
Tmod2	2.44E-07	0.592553	0.7	0.138	0.004905	4 M1 _	Tmod2
Scrn1	2.36E-07	0.556286	0.5	0.074	0.004747	4 M1 _	Scrn1
Trim30d	2.28E-07	1.026382	0.6	0.111	0.004592	4 M1 _	Trim30d

Col14a11	2.03E-07	2.063766	1	0.458	0.004095	4 M1 _	Col14a1
Fbn11	1.96E-07	2.760873	1	0.621	0.003941	4 M1 _	Fbn1
Mt21	1.49E-07	1.19708	1	0.342	0.003004	4 M1 _	Mt2
Add31	1.22E-07	1.534489	1	0.507	0.002464	4 M1 _	Add3
Ddr21	1.09E-07	2.046759	1	0.442	0.002196	4 M1 _	Ddr2
Bmp6	9.71E-08	0.505647	0.4	0.044	0.001956	4 M1 _	Bmp6
Gng7	9.34E-08	0.29079	0.3	0.025	0.001882	4 M1 _	Gng7
Heg11	8.80E-08	1.781271	1	0.337	0.001773	4 M1 _	Heg1
Nqo21	8.57E-08	0.90846	0.7	0.138	0.001726	4 M1 _	Nqo2
Tgfbr21	8.21E-08	1.862673	1	0.652	0.001654	4 M1 _	Tgfbr2
Rhou1	7.87E-08	0.976563	0.7	0.14	0.001584	4 M1 _	Rhou
Axl1	7.67E-08	2.006641	1	0.578	0.001545	4 M1 _	Axl
Carhsp1	7.44E-08	0.967522	1	0.26	0.001499	4 M1 _	Carhsp1
Fam103a14	7.25E-08	0.623649	0.5	0.066	0.001461	4 M1 _	Fam103a1
Bicd1	6.95E-08	0.57863	0.4	0.044	0.001399	4 M1 _	Bicd1
Fam208a4	6.65E-08	0.388482	0.6	0.085	0.00134	4 M1 _	Fam208a
C31	6.15E-08	3.002608	1	0.397	0.001238	4 M1 _	C3
Ebf21	5.99E-08	1.245827	0.8	0.177	0.001205	4 M1 _	Ebf2
Cd342	5.36E-08	2.135385	1	0.447	0.00108	4 M1 _	Cd34
Scara51	5.33E-08	2.00899	1	0.407	0.001074	4 M1 _	Scara5
Timp21	4.44E-08	2.721025	1	0.705	0.000895	4 M1 _	Timp2
Exd2	4.34E-08	0.438099	0.5	0.065	0.000873	4 M1 _	Exd2
Ace3	2.92E-08	2.061176	1	0.396	0.000588	4 M1 _	Ace
Prkca	2.84E-08	0.990026	0.9	0.213	0.000573	4 M1 _	Prkca
Srd5a1	2.18E-08	0.372556	0.2	0.011	0.000439	4 M1 _	Srd5a1
Galnt161	2.03E-08	1.21939	0.7	0.128	0.00041	4 M1 _	Galnt16
Dbn1	1.91E-08	1.504961	0.9	0.26	0.000386	4 M1 _	Dbn1
Rtn1	1.88E-08	0.542594	0.4	0.043	0.00038	4 M1 _	Rtn1
Medag1	1.86E-08	1.397662	1	0.331	0.000375	4 M1 _	Medag
Folh1	1.80E-08	0.277392	0.1	0.003	0.000362	4 M1 _	Folh1
Adamts21	1.77E-08	1.781133	1	0.307	0.000357	4 M1 _	Adamts2
Igfbp6	1.64E-08	2.678025	1	0.488	0.00033	4 M1 _	Igfbp6
Uap11	1.60E-08	1.671771	0.9	0.265	0.000323	4 M1 _	Uap1
Galnt15	1.52E-08	1.087915	0.7	0.132	0.000307	4 M1 _	Galnt15
Agap1	1.52E-08	1.106691	0.8	0.165	0.000307	4 M1 _	Agap1
Efemp11	1.45E-08	2.551018	1	0.355	0.000293	4 M1 _	Efemp1
Dmpk2	1.22E-08	0.571566	0.7	0.113	0.000246	4 M1 _	Dmpk
Wnt2	8.87E-09	0.412859	0.5	0.059	0.000179	4 M1 _	Wnt2
Vasn	8.80E-09	1.719349	1	0.298	0.000177	4 M1 _	Vasn
Gfpt21	8.41E-09	1.926354	1	0.319	0.000169	4 M1 _	Gfpt2
Taf6l2	7.84E-09	0.724437	0.6	0.089	0.000158	4 M1 _	Taf6l

Dact1	7.52E-09	1.433616	0.9	0.206	0.000151	4 M1 _	Dact1
Gas7	7.06E-09	1.928091	1	0.353	0.000142	4 M1 _	Gas7
D17Wsu92e4	6.29E-09	0.649565	0.5	0.06	0.000127	4 M1 _	D17Wsu92e
Clec3b1	6.07E-09	2.615731	1	0.435	0.000122	4 M1 _	Clec3b
Tbx18	2.96E-09	0.734627	0.5	0.059	5.97E-05	4 M1 _	Tbx18
Klf41	2.56E-09	2.246031	1	0.313	5.15E-05	4 M1 _	Klf4
Cd2481	2.47E-09	2.63618	1	0.423	4.96E-05	4 M1 _	Cd248
Gm10073	2.29E-09	0.406864	0.2	0.009	4.61E-05	4 M1 _	Gm10073
Galnt17	2.25E-09	0.692291	0.6	0.085	4.54E-05	4 M1 _	Galnt17
Tek1	1.70E-09	0.963359	0.9	0.163	3.43E-05	4 M1 _	Tek
Angptl41	1.57E-09	0.96097	0.6	0.08	3.16E-05	4 M1 _	Angptl4
Klhl131	1.52E-09	0.911853	0.8	0.15	3.07E-05	4 M1 _	Klhl13
Mst1r	1.42E-09	0.257497	0.3	0.02	2.85E-05	4 M1 _	Mst1r
Fam208b3	1.39E-09	0.583556	0.4	0.036	2.79E-05	4 M1 _	Fam208b
Hoxc4	1.07E-09	0.25568	0.1	0.002	2.15E-05	4 M1 _	Hoxc4
Ifi2051	6.52E-10	2.529903	1	0.295	1.31E-05	4 M1 _	Ifi205
Cdon1	5.66E-10	1.058046	0.7	0.107	1.14E-05	4 M1 _	Cdon
Man1a1	5.64E-10	1.939153	1	0.335	1.13E-05	4 M1 _	Man1a
Coro2b1	5.06E-10	0.699439	0.6	0.081	1.02E-05	4 M1 _	Coro2b
Uchl1	3.63E-10	0.544116	0.4	0.035	7.31E-06	4 M1 _	Uchl1
Aaed13	3.02E-10	0.713282	0.6	0.072	6.09E-06	4 M1 _	Aaed1
Osr11	2.84E-10	1.47383	0.9	0.19	5.72E-06	4 M1 _	Osr1
Anxa32	2.78E-10	2.62631	1	0.313	5.60E-06	4 M1 _	Anxa3
Sgms2	2.27E-10	0.392591	0.4	0.033	4.57E-06	4 M1 _	Sgms2
Nox4	2.04E-10	1.113638	0.6	0.078	4.10E-06	4 M1 _	Nox4
2410015M20Rik4	1.66E-10	0.766106	0.8	0.116	3.35E-06	4 M1 _	2410015M20Rik
Srgap11	1.37E-10	0.947902	0.6	0.071	2.75E-06	4 M1 _	Srgap1
Ildr2	1.16E-10	0.69903	0.7	0.093	2.33E-06	4 M1 _	Ildr2
Lurap1l1	1.04E-10	0.760607	0.6	0.069	2.09E-06	4 M1 _	Lurap1l
Arl4d1	9.96E-11	0.795171	0.5	0.051	2.01E-06	4 M1 _	Arl4d
Nhsl1	9.86E-11	0.9139	0.7	0.098	1.99E-06	4 M1 _	Nhsl1
Tmem1002	8.52E-11	2.408157	1	0.284	1.72E-06	4 M1 _	Tmem100
Emb	8.23E-11	0.718564	0.5	0.049	1.66E-06	4 M1 _	Emb
Npr11	8.16E-11	0.971925	0.8	0.121	1.64E-06	4 M1 _	Npr1
Il6ra	6.43E-11	0.648446	0.4	0.032	1.29E-06	4 M1 _	Il6ra
Arhgap201	5.33E-11	1.544576	1	0.242	1.07E-06	4 M1 _	Arhgap20
Mustn11	4.67E-11	1.035519	0.8	0.119	9.40E-07	4 M1 _	Mustn1
Gm26870	1.92E-11	0.276736	0.1	0.002	3.87E-07	4 M1 _	Gm26870
Daglb	1.57E-11	1.132388	0.7	0.095	3.17E-07	4 M1 _	Daglb
Fez11	1.38E-11	0.695542	0.6	0.067	2.77E-07	4 M1 _	Fez1
Smtnl2	1.00E-11	0.319179	0.2	0.007	2.02E-07	4 M1 _	Smtnl2

Dscr31	9.43E-12	0.409747	0.4	0.029	1.90E-07	4 M1 _	Dscr3
Hspb8	9.19E-12	0.912944	0.8	0.119	1.85E-07	4 M1 _	Hspb8
Ugdh1	7.48E-12	1.757183	1	0.242	1.51E-07	4 M1 _	Ugdh
Atp6v0e2	4.68E-12	0.470129	0.4	0.028	9.43E-08	4 M1 _	Atp6v0e2
Pde8a1	4.29E-12	1.21762	1	0.199	8.64E-08	4 M1 _	Pde8a
Pla2g163	3.41E-12	0.640368	0.5	0.043	6.88E-08	4 M1 _	Pla2g16
Pi16	3.25E-12	4.679027	1	0.265	6.54E-08	4 M1 _	Pi16
Qpct	3.01E-12	1.251781	0.8	0.121	6.05E-08	4 M1 _	Qpct
Tnxb1	2.82E-12	3.020776	1	0.238	5.69E-08	4 M1 _	Tnxb
Gria4	2.67E-12	0.391534	0.2	0.007	5.38E-08	4 M1 _	Gria4
Adh7	2.61E-12	0.331264	0.2	0.007	5.27E-08	4 M1 _	Adh7
Flnc1	2.44E-12	0.969709	0.8	0.118	4.91E-08	4 M1 _	Flnc
Kcnk5	2.24E-12	0.486537	0.4	0.028	4.50E-08	4 M1 _	Kcnk5
Fam220a3	1.03E-12	0.74497	0.6	0.057	2.08E-08	4 M1 _	Fam220a
Sirpa	5.97E-13	0.699126	0.7	0.08	1.20E-08	4 M1 _	Sirpa
Kif21a	4.81E-13	0.636532	0.4	0.026	9.69E-09	4 M1 _	Kif21a
Mkl11	4.25E-13	0.397218	0.4	0.025	8.56E-09	4 M1 _	Mkl1
Mkl22	4.02E-13	0.464695	0.6	0.053	8.09E-09	4 M1 _	Mkl2
Gpc31	2.81E-13	1.53287	0.7	0.083	5.67E-09	4 M1 _	Gpc3
Tppp31	2.64E-13	1.953479	1	0.19	5.31E-09	4 M1 _	Tppp3
Ints5.1	1.85E-13	0.365104	0.3	0.014	3.72E-09	4 M1 _	Ints5.1
Gda1	7.26E-14	1.668077	0.7	0.08	1.46E-09	4 M1 _	Gda
2810474O19Rik4	3.46E-14	1.569514	0.7	0.077	6.97E-10	4 M1 _	2810474O19Rik
Il18	2.55E-14	0.627418	0.4	0.024	5.14E-10	4 M1 _	Il18
Nat8f3	1.50E-14	0.281438	0.2	0.006	3.03E-10	4 M1 _	Nat8f3
5033421B08Rik	1.22E-14	0.338036	0.2	0.006	2.45E-10	4 M1 _	5033421B08Rik
Aes4	8.68E-15	1.439021	1	0.135	1.75E-10	4 M1 _	Aes
Gm26771	8.00E-15	0.51266	0.4	0.022	1.61E-10	4 M1 _	Gm26771
Arsi	6.21E-15	0.751605	0.6	0.051	1.25E-10	4 M1 _	Arsi
Gprc5b	4.75E-15	0.905622	0.7	0.071	9.57E-11	4 M1 _	Gprc5b
Fbxo182	4.62E-15	0.642641	0.6	0.049	9.30E-11	4 M1 _	Fbxo18
Sfrp21	3.66E-15	2.10719	0.8	0.093	7.37E-11	4 M1 _	Sfrp2
2010107E04Rik4	1.39E-15	1.344128	1	0.127	2.80E-11	4 M1 _	2010107E04Rik
Sdccag33	1.07E-15	0.554225	0.6	0.046	2.16E-11	4 M1 _	Sdccag3
Adora2b	1.06E-15	0.596945	0.5	0.033	2.14E-11	4 M1 _	Adora2b
Lrrc171	6.47E-16	1.407871	0.7	0.067	1.30E-11	4 M1 _	Lrrc17
Pamr1	5.10E-16	1.921924	0.8	0.091	1.03E-11	4 M1 _	Pamr1
Dpt1	4.79E-16	3.549243	1	0.167	9.65E-12	4 M1 _	Dpt
Ism1	3.84E-16	1.102832	0.5	0.034	7.73E-12	4 M1 _	Ism1
Zfp385a	3.75E-16	2.069098	0.9	0.123	7.55E-12	4 M1 _	Zfp385a
Emilin21	2.94E-16	2.628175	1	0.171	5.92E-12	4 M1 _	Emilin2

C4b1	2.69E-16	1.705626	0.8	0.086	5.42E-12	4 M1 _	C4b
Ackr31	1.71E-16	2.516615	0.9	0.123	3.45E-12	4 M1 _	Ackr3
Fn11	1.26E-16	3.909252	1	0.162	2.53E-12	4 M1 _	Fn1
Hoxb3	1.24E-16	0.272015	0.2	0.005	2.50E-12	4 M1 _	Hoxb3
Gm15564	9.70E-17	0.317656	0.2	0.005	1.95E-12	4 M1 _	Gm15564
AI464131	4.65E-17	0.350128	0.3	0.011	9.36E-13	4 M1 _	AI464131
Fam213b1	1.78E-17	0.630504	0.4	0.019	3.58E-13	4 M1 _	Fam213b
Sfrp4	1.66E-17	1.477214	0.4	0.019	3.35E-13	4 M1 _	Sfrp4
Zbtb163	1.52E-17	1.252849	0.9	0.097	3.07E-13	4 M1 _	Zbtb16
Edn11	1.35E-17	2.202648	0.5	0.03	2.71E-13	4 M1 _	Edn1
Adra1b	6.52E-18	0.311806	0.2	0.004	1.31E-13	4 M1 _	Adra1b
En1	5.90E-18	0.272633	0.2	0.004	1.19E-13	4 M1 _	En1
Sdk1	4.87E-18	0.780388	0.6	0.042	9.82E-14	4 M1 _	Sdk1
Sbsn	1.50E-18	2.080147	0.7	0.059	3.03E-14	4 M1 _	Sbsn
Megf9	1.46E-18	0.832358	0.8	0.071	2.94E-14	4 M1 _	Megf9
Fhl12	1.05E-18	1.590903	0.8	0.073	2.12E-14	4 M1 _	Fhl1
Fam46a2	9.28E-19	1.268693	0.7	0.054	1.87E-14	4 M1 _	Fam46a
Pygl	1.05E-19	1.003157	0.8	0.068	2.11E-15	4 M1 _	Pygl
Gm16894	7.56E-20	0.38213	0.3	0.009	1.52E-15	4 M1 _	Gm16894
Tubb4a1	5.81E-20	0.601649	0.5	0.025	1.17E-15	4 M1 _	Tubb4a
Car81	4.57E-20	1.058159	0.8	0.065	9.19E-16	4 M1 _	Car8
Vldlr1	2.64E-20	1.298497	0.8	0.067	5.32E-16	4 M1 _	Vldlr
Lamc2	8.28E-21	0.593	0.3	0.009	1.67E-16	4 M1 _	Lamc2
Efna5	4.49E-21	0.623257	0.4	0.015	9.04E-17	4 M1 _	Efna5
Sema3c1	3.82E-21	3.120442	1	0.118	7.70E-17	4 M1 _	Sema3c
Cd551	3.57E-21	2.560169	1	0.117	7.19E-17	4 M1 _	Cd55
Fndc1	4.50E-23	3.525262	1	0.108	9.07E-19	4 M1 _	Fndc1
Irf4	9.06E-24	0.441536	0.2	0.003	1.82E-19	4 M1 _	Irf4
Kdelc23	4.39E-24	1.369485	0.9	0.071	8.83E-20	4 M1 _	Kdelc2
CT025619.11	2.14E-24	0.912207	0.6	0.03	4.30E-20	4 M1 _	CT025619.1
1-Nov	2.87E-25	1.063457	0.4	0.012	5.79E-21	4 M1 _	Nov
Dact2	4.49E-26	1.381105	0.6	0.029	9.03E-22	4 M1 _	Dact2
Gm15675	2.87E-26	0.368541	0.3	0.006	5.79E-22	4 M1 _	Gm15675
Spsb4	1.24E-26	0.498472	0.2	0.003	2.49E-22	4 M1 _	Spsb4
Opcml	2.97E-27	1.535823	0.6	0.027	5.98E-23	4 M1 _	Opcml
5830416I19Rik	3.04E-28	0.378803	0.4	0.011	6.13E-24	4 M1 _	5830416I19Rik
Vegfd	3.09E-29	1.568998	0.6	0.025	6.22E-25	4 M1 _	Vegfd
Dmkn	3.64E-30	0.285188	0.2	0.002	7.32E-26	4 M1 _	Dmkn
Slc4a4	3.55E-30	1.749685	0.8	0.045	7.15E-26	4 M1 _	Slc4a4
Creb5	3.40E-30	2.523531	0.9	0.06	6.84E-26	4 M1 _	Creb5
Adam33	2.76E-31	1.54425	0.8	0.044	5.57E-27	4 M1 _	Adam33

Ppp2r2b	8.98E-32	0.615706	0.4	0.009	1.81E-27	4 M1 _	Ppp2r2b
Efhd11	1.60E-32	1.431097	0.8	0.04	3.21E-28	4 M1 _	Efhd1
Aldh1a3	1.44E-32	1.361196	0.6	0.022	2.89E-28	4 M1 _	Aldh1a3
Prkg2	2.88E-33	0.658658	0.4	0.009	5.79E-29	4 M1 _	Prkg2
Stmn4	1.54E-33	0.466519	0.3	0.005	3.10E-29	4 M1 _	Stmn4
Ston2	1.89E-34	0.98446	0.6	0.021	3.80E-30	4 M1 _	Ston2
Fam46b	4.83E-35	0.334542	0.2	0.002	9.73E-31	4 M1 _	Fam46b
Clip4	4.05E-35	1.572447	0.9	0.049	8.16E-31	4 M1 _	Clip4
Stxbp6	5.92E-36	1.201559	0.8	0.036	1.19E-31	4 M1 _	Stxbp6
Cadm31	1.52E-36	1.494623	1	0.057	3.07E-32	4 M1 _	Cadm3
Adamtsl4	1.50E-36	1.551281	0.8	0.036	3.01E-32	4 M1 _	Adamtsl4
Pcsk61	5.96E-37	3.003431	1	0.061	1.20E-32	4 M1 _	Pcsk6
Adgrd1	2.10E-40	1.867996	0.9	0.042	4.23E-36	4 M1 _	Adgrd1
Cmah	8.56E-41	1.871298	1	0.051	1.72E-36	4 M1 _	Cmah
Ackr1	3.62E-41	0.83389	0.6	0.017	7.28E-37	4 M1 _	Ackr1
Serpina3c	6.23E-42	0.438436	0.2	0.001	1.25E-37	4 M1 _	Serpina3c
Ptx3	7.49E-44	0.758436	0.4	0.006	1.51E-39	4 M1 _	Ptx3
Prdm8	3.71E-44	1.165419	0.7	0.021	7.48E-40	4 M1 _	Prdm8
Sntg1	5.12E-46	0.684606	0.4	0.006	1.03E-41	4 M1 _	Sntg1
Adgrg2	8.25E-47	0.507858	0.3	0.003	1.66E-42	4 M1 _	Adgrg2
Tmeff2	2.03E-47	1.504091	0.8	0.027	4.08E-43	4 M1 _	Tmeff2
Aif1l	1.38E-47	1.146094	0.7	0.02	2.77E-43	4 M1 _	Aif1l
1700019D03Rik	6.74E-48	1.201507	0.8	0.026	1.36E-43	4 M1 _	1700019D03Rik
Rorb	1.12E-51	0.798668	0.4	0.005	2.25E-47	4 M1 _	Rorb
Lrrn4cl	7.47E-52	2.024279	1	0.04	1.50E-47	4 M1 _	Lrrn4cl
Miat	3.61E-52	0.399217	0.2	0.001	7.26E-48	4 M1 _	Miat
Gm36888	1.79E-52	0.260133	0.1	0	3.60E-48	4 M1 _	Gm36888
Chrdl1	1.49E-53	1.424684	0.5	0.008	3.01E-49	4 M1 _	Chrdl1
Gpr1	4.47E-59	0.65698	0.4	0.004	9.01E-55	4 M1 _	Gpr1
Bmp3	1.19E-60	0.653715	0.5	0.007	2.39E-56	4 M1 _	Bmp3
Dpp4	5.56E-62	2.645448	1	0.033	1.12E-57	4 M1 _	Dpp4
Duox1	1.88E-63	0.660672	0.4	0.004	3.78E-59	4 M1 _	Duox1
Eppk1	3.50E-66	0.314584	0.3	0.002	7.05E-62	4 M1 _	Eppk1
Islr2	2.69E-68	0.79063	0.6	0.009	5.41E-64	4 M1 _	Islr2
Mmp27	2.47E-78	0.88424	0.7	0.011	4.98E-74	4 M1 _	Mmp27
Ackr2	8.78E-81	1.24855	0.6	0.007	1.77E-76	4 M1 _	Ackr2
Pcolce2	2.81E-84	3.036075	1	0.022	5.66E-80	4 M1 _	Pcolce2
Htr2a	1.48E-92	0.451531	0.3	0.001	2.99E-88	4 M1 _	Htr2a
Nova1	1.60E-98	2.103567	1	0.018	3.21E-94	4 M1 _	Nova1
Smpd3	3.52E-103	3.563491	0.8	0.01	7.09E-99	4 M1 _	Smpd3
Duoxa1	1.87E-138	0.757994	0.7	0.005	3.77E-134	4 M1 _	Duoxa1

Supplementary Table 4. Differentially expressed matrisome genes in stromal subsetted mock and ligated cells

Differentially expressed matrisome-associated gene are shown for the stromal cell subset gene lists from Supplementary Table 3. Genes colored red are expressed in both ligated and mock cells within the cluster and those with no colors are unique to the condition.

Cluster 0		Cluster 1		Cluster 2		Cluster 3		Cluster 4	
**Ligated**	**Mock**	**Ligated**	**Mock**	**Ligated**	**Mock**	**Ligated**	**Mock**	**Ligated**	**Mock**
Abi3bp	Bmper	Fgl2	Srgn	Sparcl1	Crim1	Agrn	Crim1	Aebp1	Dpt
Aebp1	Crispld2	Lamb3		**Ecm2**	Ctgf	Fbln2	Ctgf	AW551984	Efemp1
Cilp	Ctgf	Srgn		**Emid1**	Cyr61	Gas6	Cyr61	Cthrc1	Emilin2
Creld2	Cyr61			**Lgi1**	Ecm2	Hmcn1	Ecm2	Ecm1	Fbn1
Crispld1	Dpt			**Mfge8**	Edil3	Igfbp3	Edil3	Fbln2	Fn1
Efemp2	Ecm1			**Tinagl1**	Emid1	Igfbp7	Emid1	Fbln5	Fndc1
Egflam	Efemp1			**Vtn**	Lamb2	Lama3	Lamb2	Igfbp5	Igfbp6
Eln	Emilin2			**Aspn**	Lgi1	Lama5	Lgi1	Lama4	Lamc2
Emilin1	Fbln1				Mfge8	Lrg1	Mfge8	Lgi2	Lgi4
Fbn2	Fbln2				Ntn4	Ltbp4	Ntn4	Lgi3	Mfap5
Fgl2	Fbln5				Postn	Mfap3	Postn	Thbs2	Nid1
Gas6	Fbln7				Sparc	Mmrn2	Sparc	Thbs3	Nov
Igfbp6	Fbn1				Thsd4	Vwa1	Thsd4	Tnfaip6	Pcolce2
Igfbp7	Fn1				Tinagl1	Col13a1	Tinagl1	Col1a1	Tnxb
Ltbp2	Igfbp4				Vtn	Esm1	Vtn	Col1a2	Col14a1
Mfap2	Igfbp5				Col4a1	Hspg2	Col4a1	Col3a1	Col5a1
Mfap4	Igsf10				Col4a2	Srgn	Col4a2	Ogn	
Mfap5	Lama2				Aspn	**Crim1**	Aspn	**Dpt**	
Mgp	Lama4					**Tinagl1**		**Efemp1**	
Postn	Lamb1					**Col4a1**		**Emilin2**	
Slit2	Lamc1					**Col4a2**		**Fbn1**	
Sparc	Lamc3							**Fn1**	
Spp1	Lgi2							**Fndc1**	
Svep1	Lgi4							**Igfbp6**	
Thbs2	Ltbp1							**Lamc2**	
Thbs4	Ltbp3							**Mfap5**	
Col11a1	Ltbp4							**Nid1**	
Col12a1	Nid1							**Pcolce2**	
Col16a1	Nid2							**Tnxb**	
Col28a1	Pcolce							**Col14a1**	
Col4a3	Pxdn							**Col5a1**	
Col4a4	Rspo1								
Col4a5	Rspo3								
Col8a1	Slit3								
Col8a2	Smoc2								
Bgn	Sned1								
Fmod	Spon1								
Omd	Spon2								
**Crispld2**	Srpx								
**Ecm1**	Srpx2								
**Efemp1**	Tgfbi								
**Fbln1**	Thbs1								
**Fbln5**	Thbs3								
**Fbln7**	Tnfaip6								

Fbn1	Tnxb
Igfbp4	Vit
Igfbp5	Vwa5a
Igsf10	Wisp1
Lama2	Col14a1
Lamb1	Col15a1
Lamc1	Col18a1
Lamc3	Col1a1
Ltbp1	Col1a2
Ltbp3	Col23a1
Nid1	Col27a1
Nid2	Col3a1
Pcolce	Col4a1
Rspo3	Col5a1
Slit3	Col5a2
Smoc2	Col5a3
Sned1	Col6a1
Spon1	Col6a2
Srpx	Col6a3
Srpx2	Col6a5
Tgfbi	Col6a6
Thbs1	Dcn
Col14a1	Hspg2
Col15a1	Lum
Col1a1	Ogn
Col1a2	Podn
Col27a1	Prelp
Col3a1	Prg4
Col5a1	Spock2
Col5a2	Vcan
Col5a3	
Col6a1	
Col6a2	
Col6a3	
Col6a6	
Dcn	
Hspg2	
Lum	
Ogn	
Podn	
Prelp	
Vcan	
